# Developing best practices for genotyping-by-sequencing analysis in the construction of linkage maps

**DOI:** 10.1093/gigascience/giad092

**Published:** 2023-10-27

**Authors:** Cristiane Hayumi Taniguti, Lucas Mitsuo Taniguti, Rodrigo Rampazo Amadeu, Jeekin Lau, Gabriel de Siqueira Gesteira, Thiago de Paula Oliveira, Getulio Caixeta Ferreira, Guilherme da Silva Pereira, David Byrne, Marcelo Mollinari, Oscar Riera-Lizarazu, Antonio Augusto Franco Garcia

**Affiliations:** Department of Genetics, University of São Paulo, São Paulo 13418-900, Brazil; Department of Horticultural Sciences, Texas A&M University, College Station, TX 77843-0001, USA; Department of Genetics, University of São Paulo, São Paulo 13418-900, Brazil; Mendelics Genomic Analysis, São Paulo 02511-000, Brazil; Department of Genetics, University of São Paulo, São Paulo 13418-900, Brazil; Department of Horticultural Sciences, Texas A&M University, College Station, TX 77843-0001, USA; Department of Genetics, University of São Paulo, São Paulo 13418-900, Brazil; Bioinformatics Research Center, Department of Horticultural Sciences, North Carolina State University, Raleigh, NC 27695-7566, USA; Roslin Institute, University of Edinburgh, Edinburgh EH25 9RG, Scotland; Department of Genetics, University of São Paulo, São Paulo 13418-900, Brazil; Department of Agronomy, Federal University of Viçosa, 36570-900, Brazil; Department of Horticultural Sciences, Texas A&M University, College Station, TX 77843-0001, USA; Bioinformatics Research Center, Department of Horticultural Sciences, North Carolina State University, Raleigh, NC 27695-7566, USA; Department of Horticultural Sciences, Texas A&M University, College Station, TX 77843-0001, USA; Department of Genetics, University of São Paulo, São Paulo 13418-900, Brazil

**Keywords:** genotyping error, haplotype, genetic maker, multiallelic

## Abstract

**Background:**

Genotyping-by-sequencing (GBS) provides affordable methods for genotyping hundreds of individuals using millions of markers. However, this challenges bioinformatic procedures that must overcome possible artifacts such as the bias generated by polymerase chain reaction duplicates and sequencing errors. Genotyping errors lead to data that deviate from what is expected from regular meiosis. This, in turn, leads to difficulties in grouping and ordering markers, resulting in inflated and incorrect linkage maps. Therefore, genotyping errors can be easily detected by linkage map quality evaluations.

**Results:**

We developed and used the Reads2Map workflow to build linkage maps with simulated and empirical GBS data of diploid outcrossing populations. The workflows run GATK, Stacks, TASSEL, and Freebayes for single-nucleotide polymorphism calling and updog, polyRAD, and SuperMASSA for genotype calling, as well as OneMap and GUSMap to build linkage maps. Using simulated data, we observed which genotype call software fails in identifying common errors in GBS sequencing data and proposed specific filters to better handle them. We tested whether it is possible to overcome errors in a linkage map using genotype probabilities from each software or global error rates to estimate genetic distances with an updated version of OneMap. We also evaluated the impact of segregation distortion, contaminant samples, and haplotype-based multiallelic markers in the final linkage maps. Through our evaluations, we observed that some of the approaches produce different results depending on the dataset (dataset dependent) and others produce consistent advantageous results among them (dataset independent).

**Conclusions:**

We set as default in the Reads2Map workflows the approaches that showed to be dataset independent for GBS datasets according to our results. This reduces the number of required tests to identify optimal pipelines and parameters for other empirical datasets. Using Reads2Map, users can select the pipeline and parameters that best fit their data context. The Reads2MapApp shiny app provides a graphical representation of the results to facilitate their interpretation.

## Introduction

Advances in sequencing technologies and the development of different genome-reduced representation library protocols result in millions of genetic markers from hundreds of samples in a single sequencing run [1–4]. Increasing the number of markers and individuals genotyped can enhance the capacity of linkage maps to locate recombination events that occur, resulting in higher map resolution and better statistical power for the localization of QTL in further analysis. This large amount of data and genotyping errors common with genotyping-by-sequencing (GBS) approaches [5] increases the need for computational resources and multiple bioinformatic tools.

Genotyping errors are frequent when high-throughput sequencing technology is applied to reduced representation libraries. There are a variety of protocols to create these types of libraries [4], called restriction site–associated DNA sequencing (RADseq) or GBS [6, 7]. Generally, 1 or more restriction enzymes are used to digest the sample DNA. The resulting DNA fragments are filtered by size, connected to adaptors and barcodes, amplified by polymerase chain reaction (PCR), and sequenced. Consequently, most sequences obtained are PCR duplicates of the regions around the enzyme cut site. By relying on duplicates to increase sequencing depth, such methods introduce errors and a sequencing bias toward one of the alleles due to variabilities in the PCR amplification. These errors are hard to detect by bioinformatic tools [8, 9].

To overcome genotyping errors coming from GBS methods, genotype calling software models sequencing error, allelic bias, overdispersion, outlying observations, and the population Mendelian expected segregation [10]. Building a genetic map with genotypes obtained using these methods can be a powerful tool to validate their efficiency. Wrong decisions or inefficient methods in all steps before linkage map building can be identified in the resulting map as errors that dissociate the map properties from biological processes. For example, genotyping errors generate inflated map sizes that show an excessive number of recombination breakpoints during meiosis [11]. The first genetic map studies by Sturtevant [12] discovered that crossing-overs are unlikely to happen too close to each other, a phenomenon named interference. Later studies describing the meiotic molecular mechanisms confirmed the low expected number of recombination breaks in a single event [13].

Recently developed approaches to build linkage maps [14–16] were implemented in the OneMap [17] 3.0 package. They use quantitative genotype probability measurements rather than the traditional qualitative genotypic information from single-nucleotide polymorphism (SNP) and genotype calling methods to account for genotyping errors and provide higher-quality genetic maps. These probabilities can be applied in different ways: using the probability of each possible genotype (PL field in VCF format), using an error probability associated with the called genotype (GQ field in VCF format), or using a global error rate that will be applied to all genotypes. Nevertheless, even using these approaches, building a linkage map will succeed only if the upstream software can identify the errors and provide reliable genotypes or their probabilities.

The biallelic codominant nature of SNPs is another characteristic of high-throughput markers that can affect linkage map building of outcrossing species. Although biallelic markers can distinguish only 2 haplotypes, the mapping population of outcrossing diploid species inherits 2 haplotypes with combinations of 4 different parental haplotypes. With biallelic markers, the observed parental genotypes are limited to types *ab* × *ab, ab* × *aa*, and *aa* × *ab*. When one of the parents is homozygous (*ab* × *aa* and *aa* × *ab*), it is impossible to observe the crossing-over change for this uninformative parent. So this is taken as missing information (nonmeasurable crossing-overs) for linkage map building if only 2-point information is considered. Therefore, building a linkage map with only biallelic markers requires a multipoint approach that uses loci information with both parents heterozygous (*ab* × *ab*) to estimate the recombination of loci where 1 parent is homozygous, and the recombination information is missing for closely linked loci. The multipoint approach applies likelihood computations involving several loci and has been successfully used since the seminal publication of Lander and Green [18]. The approach makes it possible to identify the 4 different parental haplotypes by phasing the biallelic information so that the SNPs can be used to identify all the allelic diversity.

Other approaches to overcome the low informativeness of biallelic markers involve combining adjacent biallelic markers in the same disequilibrium block (high LD) into a single multiallelic haplotype. These haplotype-based markers showed higher accuracy in association analysis than individual biallelic SNPs [19–25]. N’Diaye et al. [21] and Jiang et al. [25] pointed out several advantages of haplotype-based markers, including the higher capacity to identify epistatic interactions, the presence of more information to estimate identical-by-descent alleles, and the reduction of the number of statistical tests to perform.

Despite the availability of many software for estimating genotype probabilities [2, 10, 26–29] and haplotype-based multiallelic markers [26, 30], there are no recommendations about which combination and choice of parameters are the best for building linkage maps. Therefore, this work evaluates the consequences of building maps by applying genotype probabilities and haplotype-based markers from different software and parameters. To achieve these, we implemented new features in OneMap [17], a widely used software for building maps. We also developed the Reads2Map workflow, a tool to help users select a bioinformatic pipeline that provides the best-quality markers to build a linkage map for their dataset. Here, we performed tests with simulated and empirical data and were able to make recommendations to users to obtain better linkage maps in several situations, such as low- and high-depth sequencing, with and without segregation distortion, contaminant samples, and multiallelic markers, and using different software to perform the SNP and genotype calling.

## Material and Methods

We developed Reads2Map (RRID SCR_023593), a collection of bioinformatics workflows using Workflow Description Language (WDL) [31]. It enables sequence alignment, SNP and genotype calling analysis, and linkage map construction. With Reads2Map, researchers have the flexibility to explore various software options and parameter combinations, enhancing the construction of linkage maps. The workflows are available in GitHub [32] and in workflowhub.eu [33, 34].

**Figure 1:**
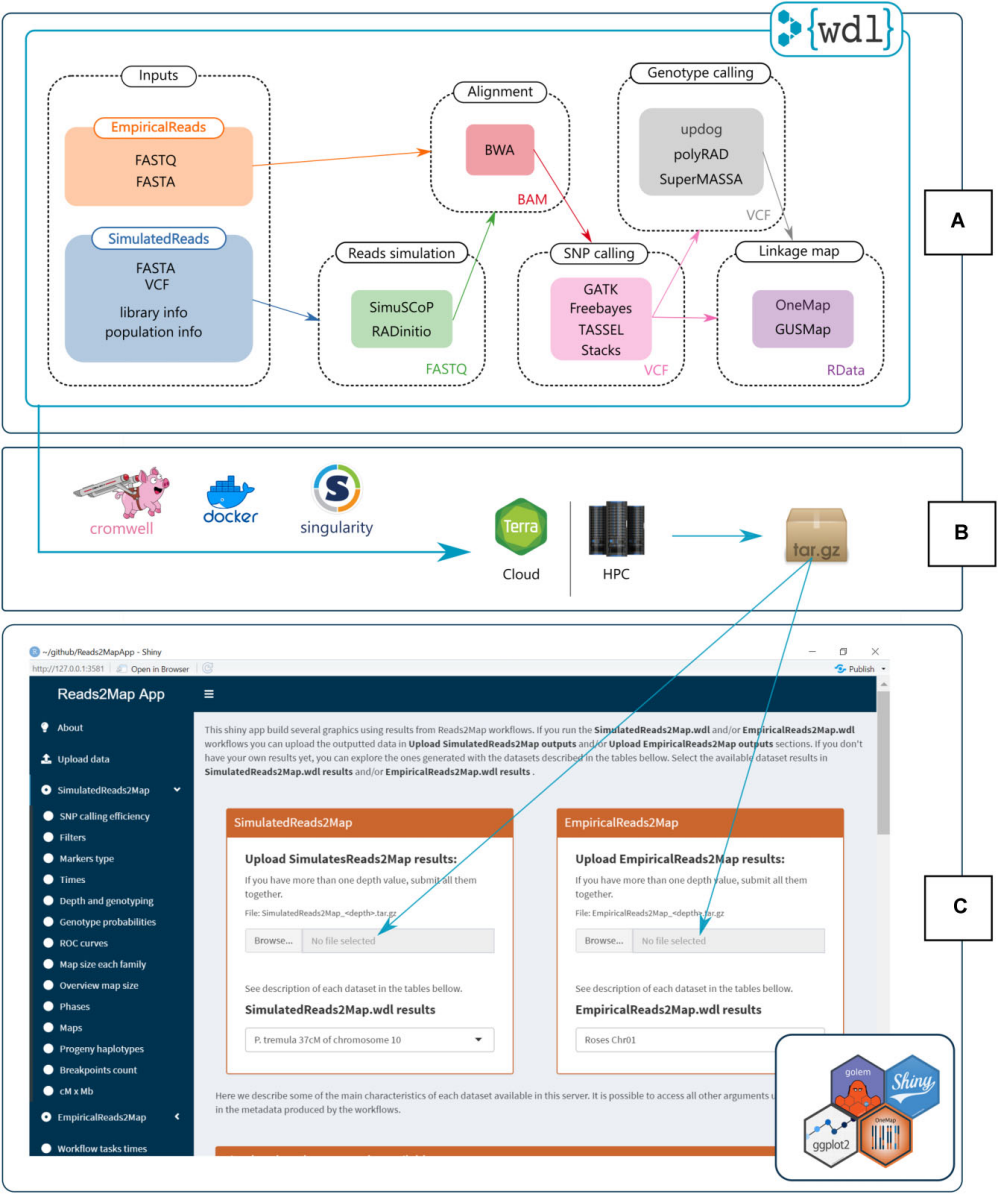
(A) Tasks of the 2 main Reads2Map workflows: EmpiricalReads2Map and SimulatedReads2Map. (B) Tools to run the workflows on the Cloud [35] or in high-performance computing (HPC) environments. (C) The Reads2Map shiny app has as input the outputs of the workflows. It builds several descriptive graphics to evaluate the best upstream software combination for linkage map construction.

The EmpiricalReads2Map workflow was designed to evaluate empirical (real) datasets, and the SimulatedReads2Map workflow was to simulate and evaluate datasets (Fig. 1). Both are composed of subworkflows that can be run independently, which increases usage flexibility. There are multiple options available for running WDL workflows. Some of them are Terra.bio platform [35] and Cromwell Execution Engine [31].

Each WDL task in Reads2Map is related to a Docker [36] or Singularity [37] container. Some of the container’s images used in Reads2Map are available in open repositories, and others were built using Dockerfiles stored in the Reads2Map repository and available in DockerHub. Check a list of all software and image versions used in Supplementary Table S1. We ran the analysis testing workflows on 2 high-performance computers (Texas A&M University HPRC, University of São Paulo Águia Cluster).

For building linkage maps, we implemented updates in OneMap package version 3.0 and used this version in the workflows. We also developed the Reads2MapTools [38] R package for support functions and Reads2MapApp shiny app [39], a visualization tool that receives as input the final workflow output and provides summary statistics about the resulting linkage maps, intermediary steps, and workflow performance.

### SNP calling

The first step of the workflows is the SNP calling. To start with GATK [27], Stacks [2], and Freebayes [26] approaches, the demultiplexed FASTQ sequences are first aligned to their respective reference genomes using BWA-MEM [40]. The workflow uses samtools [41] to merge the alignment of replicates, keeping the libraries’ identification on the BAM header and filtering out reads with MAPQ <10. After the alignment, BAM files for each sample are used as inputs for subworkflows with GATK, Stacks, and Freebayes tasks. The gatk_genotyping subworkflow reproduces GATK joint genotyping via HaplotypeCaller, GenomicsDBImport, and GenotypeGVCFs tools and applies the suggested hard-filtering procedures [8]. The freebayes_genotyping subworkflow runs Freebayes parallelized by reference genome intervals. The stacks_genotyping subworkflow includes the option to input the population file. If not included, all individuals are considered from the same population. It runs the gstacks and the populations plugins.

The TASSEL [1] SNP caller is implemented in the tassel_genotyping subworkflow. It first adds fake barcodes to the demultiplexed FASTQ sequences. After, it runs the plugins GBSSeqToTagDBPlugin and TagExportToFastqPlugin. The generated tags are aligned to the reference genome using BWA-MEM and the alignment files are input for the SAMToGBSdbPlugin plugin, which produces a database. The database is processed by the DiscoverySNPCallerPluginV2, SNPQualityProfilerPlugin, and ProductionSNPCallerPluginV2 plugins.

After obtaining the VCF file using 1 or more of the SNP calling methods, indel marker positions are left-aligned and normalized with BCFtools [42].

### Genotype calling

The VCF files with biallelic markers from Freebayes, TASSEL, Stacks, and GATK are the input for the genotype caller software polyRAD [28], SuperMASSA [29], and updog [10]. These 3 software are implemented in the subworkflows genotyping_empirical and genotyping_simulated.

To use the polyRAD approach, the VCF files are imported using VCF2RADdata without applying any filters or considering phase information. The polyRAD model is run with PipelineMapping2Parents default arguments, which assume an *F*_1_ biparental population. The function Export_MAPpoly is used to export the genotype probabilities. The vcfR package [43] and custom R (function polyRAD_genotype_vcf in Reads2MapTools package) code are used to store outputted genotypes and their probabilities in a new VCF file. We also adapted SuperMASSA scripts to output the genotype probabilities information. The modified version is available in the Reads2MapTools package. A wrapper function called supermassa_genotype, available in the package, can run the model in parallel and export the results to a new VCF file. The *F*_1_  SuperMASSA model is run with the parameter naive_posterior_reporting_threshold set to zero to not filter any genotype. The updog  *F*_1_ model is used in parallel using the function multidog through the Reads2MapTools wrapper function updog_genotype, which outputs the results in a new VCF file.

The software GUSMap performs the genotype calling and linkage map building with a single model. We use VCFtoRA function to convert the outputted VCF files from GATK, TASSEL, Stacks, and Freebayes approaches into GUSMap format. A pedigree of the population and a list of filters (MAF=0.05, MISS=0.25, BIN=0, DETPH=0, and PVALUE=0.05) are provided to the readRA function. The function makeFS is used to create the full-sib population information. Functions infer_OPGP_FS and rf_est_FS are used to estimate the phase and recombination fraction given the genomic order of the markers. In some situations, the function rf_est_FS outputs infinite values of the recombination fraction. In these situations, our pipeline removes the respective marker and runs the function again. This workaround code can increase the time required to run GUSMap.

### Updates in OneMap 3.0 for building linkage maps

OneMap is an open-source R package that has been serving the research community since its initial release in 2007. It offers a comprehensive suite of functions designed to facilitate marker filtering, grouping, ordering, and genetic distance estimation in both inbred and outbred populations. The genetic distance estimation is made using a hidden Markov model (HMM) multipoint approach. The forward–backward algorithm [44] is implemented to compute the HMM combined with the expectation–maximization (EM) algorithm.

The OneMap latest version (3.0) is implemented in Reads2Map workflows. In this new version, we have introduced a new feature to enhance the flexibility of the HMM in scenarios where genotyping errors are expected in the dataset. This update includes the create_probs function and modifications to the HMM algorithm. With this option, users can provide OneMap with prior information regarding the reliability of each input genotype, thereby increasing the HMM’s adaptability. The create_probs function allows users to input 3 types of values: a global error value (global_error), an error probability for each inferred genotype (genotypes_error), or genotype probabilities for each possible genotype in individuals (genotypes_probs). This flexibility empowers users to tailor the analysis to their specific dataset characteristics and improve the accuracy of the results. This update is described in detail in Supplementary File S1.

The OneMap software previous to version 3.0 considered the HMM error probability as a single value of 10^−5^ for every genotype. In version 3.0, this value is kept as default to keep the code reproducible. However, it is noteworthy that this probability can be unreliable in several situations when the genotypes are more prone to errors, especially for new genotyping technology (e.g., GBS data).

OneMap 3.0 updates also include the possibility to parallelize the HMM using the approach described by [45]. It parallelizes the procedure into a maximum of 4 cores. We used this new OneMap feature to estimate the genetic distances. We also implemented new functions for linkage maps quality diagnostics such as interactive plots for recombination fraction matrices, progeny haplotype representation, and counts of the recombination breakpoints in progeny.

Despite using the parallelized HMM, the genetic distance estimations in OneMap can take time to run with a high number of markers, chromosomes, and tested combinations of software. Therefore, the EmpiricalReads2Map workflow runs the HMM in just a subset of markers, which can be a single chromosome or a fragment of a chromosome. The alignment, the SNP, and genotype calling steps are performed with the entire dataset. After running the workflow and deciding the pipeline that provided the best results, the respective VCF output can be used to build the linkage map for all chromosomes in the R environment with OneMap functions.

The OneMap function onemap_read_vcfR is used to convert the VCFs to the OneMap R object format. The markers are filtered again by a maximum number of missing data of 25% because the VCF files include unexpected genotypes according to the segregation of a given locus (e.g., in a cross “aa × ab,” genotype “bb” cannot exist). OneMap makes this genotype call missing. Markers are also filtered if the segregation distortion is under a global significance level of 0.05 with Bonferroni correction and if they are redundant. Markers are ordered according to the reference genome position.

The Reads2Map workflows give flexibility to the user to define the probabilities to be used in the OneMap HMM for the estimation of the genetic distances. Users can provide more than 1 value to be tested as global errors (global_error input), can choose to use the upstream genotype caller error probability (genoprob_error input), and can provide global error values to be considered together with the software probabilities (genoprob_global_error input) according to the following: 1 − (1 − *global* *error*) *×* (1 − *software* *error* *probability*).

For GATK, TASSEL, Stacks, and Freebayes callers, the workflow uses in the HMM the Phred score genotype error (GQ FORMAT value) converted to probabilities. For the software polyRAD, SuperMASSA, and updog, it uses 1 − *output* *genotype* *probability* as a genotype error. For the latter, the population’s structure (*F*_1_) is used as *a priori* information to increase the accuracy of the estimated genotypes.

The simulations do not consider interference in the recombination events. Therefore, the Haldane map function was used to estimate the genetic distances in SimulatedReads2Map. Kosambi’s map function was applied to estimate the genetic distances in the EmpiricalReads2Map.

### Read2Map workflows app

The shiny app Reads2MapApp was built to display results from the workflow analysis. It includes graphics and statistics about SNP calling efficiency, the number of markers discarded by filtering steps, marker types, computer resources and time spent by each step of the workflow, allele depth by genotype, genotype probabilities, map size, map phases, recombination fraction matrix, progeny haplotypes, breakpoints count, and the correlation between linkage map and reference genome markers positions. Reads2MapApp is a modular R package using the golem framework [46] that can be rendered and displayed locally or on a server. It can be installed from its GitHub repository and run with a single command (run_app). Once the Reads2Map output file is uploaded into the app, all graphics will be automatically generated.

### Empirical datasets

We used the structure of Reads2Map to test the effects in the linkage map built using different combinations of software and parameters in datasets with different characteristics. For our tests with empirical data, we used 2 datasets from previous works. They are GBS datasets from a biparental diploid *F*_1_ full-sib mapping populations of aspen (*Populus tremula L*.) [47] (BioProject PRJNA395596) and rose (*Rosa* spp.) [48]. The aspen dataset comes from an intraspecific cross of 2 *P. tremula* genotypes. The GBS libraries were built using *HindIII* and *NalIII* enzymes and sequenced as 150-bp single-end reads on an Illumina HiSeq2500. Eight library replicates were built and sequenced for the parents and only 1 for each of the 116 F1 offspring. The dataset includes 6 samples erroneously sequenced as part of the progeny and later identified as contaminants. An average read depth of approximately 6× for progeny and 58× for parental samples was observed from the sequencing process. The *Populus trichocarpa* genome version 3.0 [49] was used as a reference for the sequence’s alignment. It is about 397 Mb in size.

The diploid roses dataset comprises 138 individuals from the cross between a Texas A&M breeding line J06-20-14-3 (J14-3) and cultivar Papa Hemeray (PH). GBS libraries were built with *NgoMIV* enzyme and sequenced as a 113-bp single-end read on a HiSeq2500. The parent J14-3 was repeated twice and the PH sample 3 times. An average read depth of approximately 94× for progeny and 528× for parental samples was observed from the sequencing process. The *Rosa chinensis* v1.0 genome assembly [50] was used as a reference genome to align the sequences. It is about 527 Mb in size.

The sequencing reads of the 2 empirical datasets were filtered using the Stacks plugin process_radtags [2] to filter sequences by the presence of the restriction site and sequencing quality. The reads were discarded if the average quality score of 50% of its length was below the Phred score of 10 (or 90% probability of being correct). The software cutadapt [51] was used to remove adapters and filter by a minimum read length of 64 bp. The sequences were then evaluated in our EmpiricalReads2Map workflow.

### Simulated GBS data

The first step of the SimulatedReads2Map workflow is to perform simulations of a mapping population, GBS libraries, and sequences. The simulation is based on a given reference genome chromosome sequence. If a reference linkage map and a VCF file are provided, the workflow simulates the marker genetic distances and parental genotype frequencies based on them. A cubic spline interpolation with the Hyman method [52] is applied to simulate the centimorgan position for each marker’s physical position based on this same relation on the reference linkage map provided.

We based our simulation analysis on the first 37% of the chromosome 10 sequence of *P. trichocarpa* version 3.0, which includes a sequence with 8.426 Mb from a total chromosome size of about 23 Mb. This sequence comprises 38 cM (21%) of the linkage group 10 built using the aspen empirical data [47]. Due to the computational resources needed to build such a high number of maps, we used only a subset of the data to finish the analysis in a reasonable time. Chromosome 10 was randomly chosen.

We simulated markers with different expected segregation patterns according to parental genotypes in each locus. Table 1 shows the notation used by OneMap for each possible marker type in an outcrossing diploid population. The SimulatedReads2Map workflow simulates parental haplotypes using the same proportion of marker types identified in the empirical VCF file. This approach overcomes the missing data present in the empirical dataset. The final VCF file used as a reference to the simulations contains 810 markers (126 B3.7, 263 D1.10, 278 D2.15, and 143 noninformative markers with both parents homozygous), which results from the aspen empirical data GATK SNP calling, filtered by a maximum of 25% of missing data and minor allele frequency (MAF) of 5%.

**Table 1:** Marker types according to parental genotype combinations and progeny segregation. The letters “a,” “b,” “c,” and “d” represent different alleles and the letter “o” represents null alleles. Adapted from [53].

			Parents	Progeny	
Marker type			Cross	Observed genotypes	Expected segregation
A		1	ab × cd	ac,ad,bc,bd	1:1:1:1
		2	ab × ac	a,ac,ba,bc	1:1:1:1
		3	ab × co	ac,a,bc,b	1:1:1:1
		4	ao × bo	ab,a,b,o	1:1:1:1
B	*B*_1_	5	ab × ao	ab,2a,b	1:2:1
	*B*_2_	6	ao × ab	ab,2a,b	1:2:1
	*B*_3_	7	ab × ab	a,2ab,b	1:2:1
C		8	ao × ao	3a,o	3:1
D	*D*_1_	9	ab × cc	ac,bc	1:1
		10	ab × aa	a,ab	1:1
		11	ab × oo	a,b	1:1
		12	bo × aa	ab,a	1:1
		13	ao × oo	a,o	1:1
	*D*_2_	14	cc × ab	ac,bc	1:1
		15	aa × ab	a,ab	1:1
		16	oo × ab	a,b	1:1
		17	aa × bo	ab,a	1:1
		18	oo × ao	a,o	1:1

PedigreeSim v2.1 software [54] is implemented in the workflow to simulate the meiosis events and generate an *F*_1_ progeny based on the provided genetic map and simulated parental haplotypes. We did not consider interference in meiotic events (Haldane [55] mapping function). PedigreeSim output files were converted to VCF files using Reads2MapTools R package function pedsim2vcf.

While converting the files, the pedsim2vcf function can also simulate segregation distortion by applying a selection strength. For that, a high number of individuals in the progeny have to be simulated with the PedigreeSim software and 1 or more loci to be under a given selection intensity. In our study, we targeted a final population size of 200 individuals. For that, we simulated 50 × 200 individuals and applied a selection intensity of 50% in the 30th marker, eliminating 50% of the genotypes containing one of the alleles. Then, 200 individuals of the resulting population are randomly selected to compose the mapping population. We used this feature to compare software performance with segregation distortion.

The VCF file output by pedsim2vcf and the reference genome file are inputs for the RADinitio [9] software. RADinitio adds the VCF polymorphisms in the reference genome sequence and simulates the GBS sequences. It uses the inherited efficiency model [56] to simulate a PCR-amplified pool of molecules. The model includes the heterogeneity of the PCR amplification and the polymerase substitution errors. Next, RADinitio applies the user-defined ratio between DNA original molecules to be sequenced and PCR duplicates to create a distribution that will define the number of times the pool of loci is sampled, the number of duplicate molecules that are generated from a RAD locus template, and the distribution of PCR errors in the resulting reads. We defined the default parameter with a proportion of 4:1. Besides the PCR errors inserted during the pool sampling, the software also includes a commonly observed error pattern, where the 3′ end of the read accumulates more errors than the 5′ [57]. We tested different values of PCR cycles (5, 9, and 14) and mean depth (5, 10, and 20) to simulate the FASTA files. We set the other RADinitio simulation parameters to obtain 150 bases of read length, sequence size of 350 (parameter “–insert-mean”), and restriction enzymes *HindIII* and *NalIII*. The mean read depth parameter for the parental samples was 8 times higher than the progeny. The combination of RADinitio parameters that produced results closer to those observed in empirical data was selected to perform simulations with and without segregation distortion, 5 repetitions (5 families), and 2 average sequencing depths (10 and 20) and 5 PCR cycles.

RADinitio does not output the sequence quality scores, so we converted the FASTA file format to FASTQ format, including a Phred score of 40 for every base simulated using seqtk [58] software. After obtaining the FASTQ files, the SimulatedReads2Map workflow followed the same tasks as the EmpiricalReads2Map, with alignment, SNP and genotype calling, and linkage map build. The SimulatedReads2Map workflow makes comparisons between real and estimated results within each step. The comparisons made during the workflow can be visualized in the shiny app Reads2MapApp.

### Tested scenarios

We ran all implemented software for SNP calling and genotype calling (GATK, Freebayes, TASSEL, Stacks, updog, SuperMASSA, and polyRAD) on the empirical and simulated datasets. In addition, we explored the substitution of VCF allele counts with counts from the alignment (BAM) files to mitigate potential biases introduced by SNP caller software when analyzing low-coverage sequence data. GATK inserts the bias when reads are filtered in the local reassembly step to avoid sequencing errors [59]. BCFtools is used to find the read depths information for each allele in BAM files and update the allele depths information in the AD (allele depth) field of the VCF file. For the aspen dataset, we also executed the workflows for every scenario in the presence of the contaminant samples.

The markers identified by the SNP callers (GATK, TASSEL, Stacks, Freebayes) were filtered by MAF of 5% and maximum missing data allowed of 25% before proceeding to the genotype callers (updog, polyRAD, and SuperMASSA). At this step, we also tested 2 other filters. One of them was removing noninformative markers from the VCF file. We considered noninformative markers homozygous in both parents or if at least one of the parental genotypes was missing. The second filter was to replace the AD field in the VCF file format by missing data when the genotype was missing. This avoids that updog, polyRAD, and SuperMASSA use the allele depth when GATK filtered out the genotype due to bad quality.

After the genotype call, we reduced the analysis to a subset of markers (the first 8.426 Mb or 37%) of *P. trichocarpa* chromosome 10 and the first 25 Mb ( 37%) of *R. chinensis* chromosome 1 reference genomes. This made it possible to build maps for all tests in a feasible time. The markers were filtered by the maximum missing data allowed of 25%, redundancy, and segregation distortion. In addition, we tested filtering the genotypes by a minimum genotype probability of 0.8.

We tested the consequences of building maps applying different genotype probabilities in the OneMap 3.0 HMM coming from 7 different genotype caller software: GATK, Freebayes, TASSEL, Stacks, polyRAD [28], SuperMASSA [29], and updog [10], with a global error rate of 0.01, 0.05, 0.1, and the OneMap 2.0 default value of 10^−5^. We also tested the combination of the 2 distributions. We compared OneMap 3.0 capacity of estimating accurate genetic distances with the GUSMap package [14] estimations since it also uses an HMM to account for errors present in sequencing data.

We also tested the consequences of the presence and absence of the Stacks haplotype-based multiallelic markers in the linkage map. To test the influence of the presence of the multiallelic markers in the ordering procedure, we built a map for the entire chromosomes 1 and 10 from the roses and aspen datasets, respectively, using the selected pipeline. We ordered the markers using MDSMap [60] (wrapper function implemented in OneMap 3.0) ordering algorithm with and without multiallelic markers.

In the testing of scenarios in which we considered multiallelic markers, the VCFs containing them were merged into the VCF files from polyRAD, SuperMASSA, and updog. The merged VCF is the input for linkage map building in OneMap version 3.0.

Table 2 shows an overview of the notations used to refer to each evaluated scenario.

**Table 2:** Notation used to refer to each evaluation scenario in empirical and simulated datasets

Workflow step	Notation	Description
Reads simulations	Depth 10	Mean read depth used
	Depth 20	to simulate the dataset
	Segregation distortion	Dataset simulated with
		segregation distortion
SNP calling	Freebayes	Software used to
	GATK	identify the variants
	TASSEL	
	Stacks	
Counts source	BAM	Source files of allele depth information
	VCF	
Filters	Only informative markers	Filter noninformative markers
		(both parents homozygous
		or at least 1 missing)
	Missing replaced	Replace AD field for missing
		data when GT is missing
Genotype calling	polyRAD	Software used to perform the
	SuperMASSA	estimation of genotype for a
	updog	given allele depth information
	SNPCaller	Software used for genotype calling is
		the same as that which performed the SNP calling
Filters	Genotype prob >0.8	Filter by minimum genotype
		probabilities of 0.8
Marker type	Biallelics	Keep only biallelic markers
	Biallelics + multiallelics	Keep biallelic and multiallelic markers
Map building	<Genotype caller name>	Maps built with genotype
		probabilities from
		<Genotype caller name>
	<Genotype caller name>	Map built with genotypes from
	(<global error rate>%)	<Genotype caller name> and
		global error of <global error probability>
	<Genotype caller name> ×	Map build with genotype probabilities
	(<global error rate>%)	from <Genotype caller name> and global
		error of <global error probability>

### Performance comparison

We conducted performance comparisons of each tested dataset and scenario based on the built linkage map quality. To consider good quality, we evaluated the following linkage map characteristics:

- Marker type:
  In outcrossing populations, it is important to have markers that have recombination information for both parents. We avoid approaches that provide only ab × aa (D1.10) or aa × ab (D2.15) in a single chromosome. The Reads2MapApp “Marker type” section describes the amount of each marker type in the linkage maps built by Reads2Map workflows.
- Marker coverage:
  It refers to how equally distributed markers are in the genome. We avoid approaches that do not detect markers in a large portion of the genomic selected area. The graphics in the Reads2MapApp section “cM × Mb” section correlate the linkage map position with the genomic positions. This is an excellent tool to evaluate marker coverage.
- Marker density:
  It refers to how equally distributed markers are on the linkage map. We avoid big gaps (higher than about 10 cM) in the linkage maps. Some of the gaps observed in the maps were due to outlier markers (a single marker with gaps in both edges). Outlier markers can be removed manually in further steps. We search for approaches that provide fewer outlier markers, which would require less manipulation later. The linkage map draw and graphics about the genetic distances among markers present in the section “Map size” of Reads2MapApp are good tools to evaluate marker density.
- Marker order:
  The efficiency of ordering algorithms can be significantly influenced by the presence of marker types that provide recombination information for both parents. In the Reads2Map workflows, to ensure accurate comparisons and to distinguish if linkage map inflation is due to different orders or genotyping errors, we have standardized the marker order across the workflow comparisons. Therefore, the order of the markers is always based on the reference genome. This means that it is crucial to carefully select, for the workflows, tests chromosome regions in the datasets that do not exhibit inversions or translocations when compared to the reference genome.
  However, in order to assess the impact of highly informative haplotype-based multiallelic markers, we conduct separate experiments outside of the workflows. In these experiments, we exclude outlier markers and evaluate the efficiency of the MDS ordering algorithms with and without the inclusion of multiallelic markers. This allows us to investigate these markers’ influence on the algorithm’s performance. We evaluate the orders provided by the different ordering algorithms by computing the absolute value of Spearman’s rank correlation between orders.
- Marker quality:
  In cases where all markers are correctly ordered (following the standardization in Reads2Map comparisons), and there is sufficient coverage and density, an inflated size of the linkage map can be attributed to a high error rate in the genotypes. Our objective is to find an approach that minimizes this inflation and brings the linkage map size closer to the expected value (e.g., 38 cM in our tested subsets).
  To identify the causes of inflated maps, the linkage map draw and recombination fraction matrix heatmap generated by Reads2MapApp prove valuable. It enables us to distinguish whether the inflation is a result of outlier markers creating gaps or due to genotyping errors.
- Estimated haplotypes:
  Together with the linkage map, the OneMap HMM multipoint approach also estimates the parents and progeny haplotypes. In a scenario without contaminant samples, we expect a low (around 1 or 2) and equally distributed number of recombination breaks across all samples. In scenarios where there are contaminant samples, we expect that their haplotypes contain a high number of estimated breaks because wrong assumptions were made, leading to the wrong estimated number for these samples. Reads2MapApp contains a section for visualizing the progeny haplotypes and also for counting the estimated number of recombination breaks.

## Results and Discussion

We use the structure of the Reads2Map workflows and the simulated and empirical datasets to test each software and some different parameters and markers filters. Our goal was to identify the approach that provides the best-quality linkage map.

We have categorized the approaches used in our analysis into 2 groups: dataset independent and dataset dependent. The dataset-independent approaches consistently produce reliable results across all datasets, while the dataset-dependent approaches exhibit varying efficiency depending on the dataset characteristics. To streamline the user experience, we have selected the dataset-independent approaches that improve linkage map quality as the default options in the Reads2Map workflows (Table 3). This simplifies the process for users by reducing the number of tests required, as these default approaches consistently yield favorable results across different datasets.

**Table 3:** Reads2Map workflows default option set based on tests with empirical and simulated data

Process	Workflow options	Default
SNP calling	Run GATK	TRUE
	Run Freebayes	FALSE
	Run Stacks	TRUE
	Run TASSEL	FALSE
	Remove duplicates	FALSE
	Replace AD by BAM counts	FALSE
	GATK hard filters	TRUE
genotype calling	Replace AD by missing when GT is missing	TRUE
	Probability threshold	0.8
	Run updog	TRUE
	Run polyRAD	TRUE
	Run SuperMASSA	FALSE
	Run GUSMap	FALSE
linkage map	Filter noninformative	TRUE
	Add multiallelics	TRUE (if available)
	Global errors	0.05
	Genotype caller probabilities	FALSE
	Genotype caller probabilities + global errors	0.05

We focused our tests and set the default options based on *F*_1_ diploid populations and GBS markers. However, because the Reads2Map workflow is modularized, the EmpiricalSNPCalling subworkflow can be used separately and applied to other population structures, ploidy, and sequencing libraries. In the case of working with sequencing libraries other than RADseq, such as whole-genome sequencing (WGS) or exome sequencing, it is important to set the option “remove duplicates” to TRUE. The PCR duplicates in RADseq data constitute the majority of the data, and they are included in the allele count while calling the genotypes, but in other types of libraries, they are considered artifacts and are removed to avoid errors [61].

**Figure 2:**
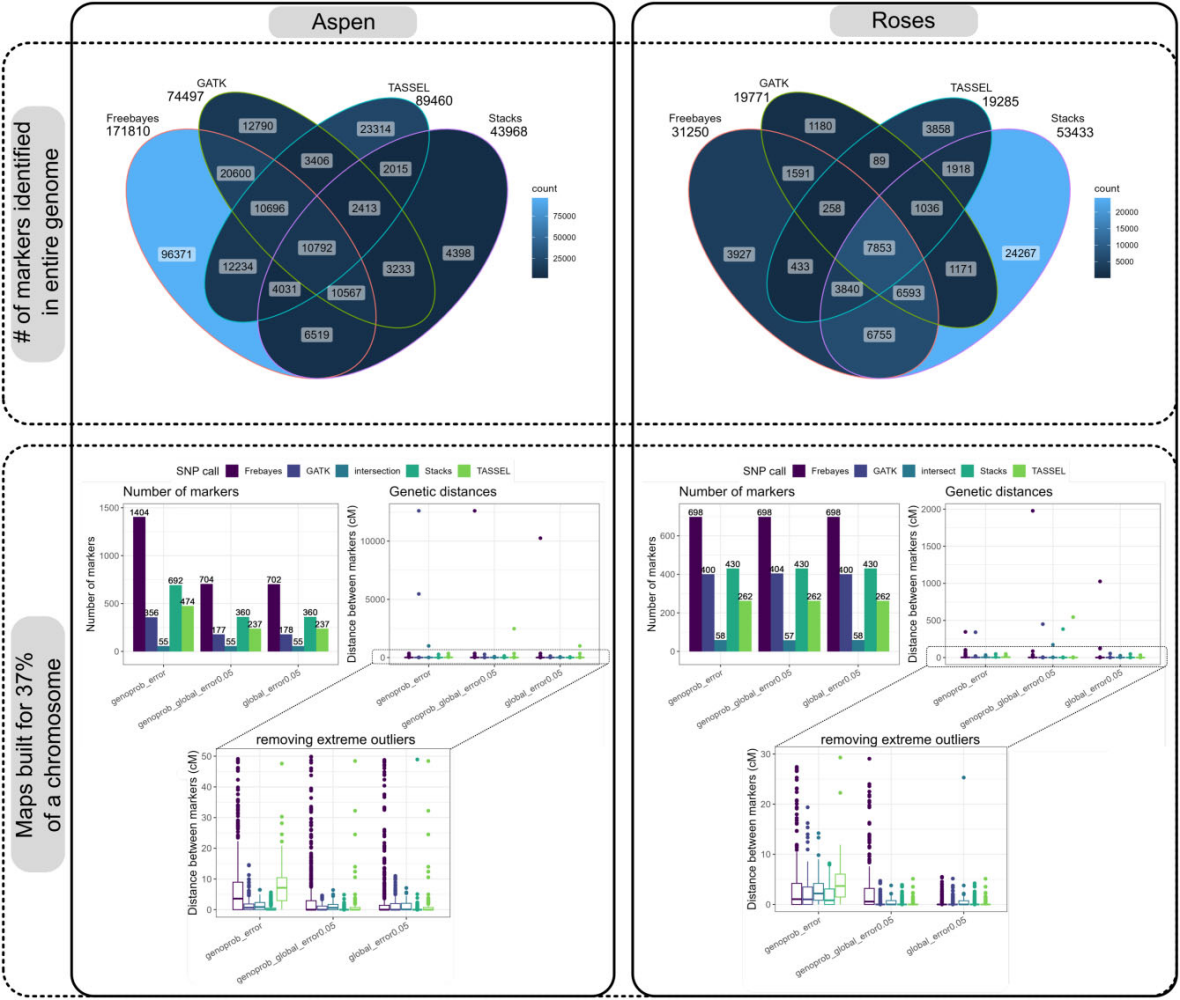
The top 2 figures show the number of markers identified by each SNP call software (number above each software name) and Venn diagrams showing the number of markers with common positions among all software results for the aspen and roses complete datasets. The markers were previously filtered by maximum missing data of 25% and MAF of 5%. The compatibility of positions among markers from different software was only possible after using “BCFtools norm” to left-align the indels positions. The bottom 2 figures show the number of markers (bar plot) and distances between markers (boxplot) after building the linkage maps for a subset of 37% of chromosome 10 in the aspen dataset and 1 in the roses dataset with the markers from Freebayes, GATK, TASSEL, and Stacks. It was considered in the OneMap HMM the genotypes and a global error of 5% (global_error0.05), genotypes probabilities (genoprob_error), and the combination of genotype probabilities and a global error of 5% (genoprob_global_error0.0.5). These figures can be generated for user-defined empirical datasets in the Reads2MapApp sections “SNP calling efficiency” and “Map size” after running the EmpiricalMaps workflow.

The genotype call and linkage map building in the EmpiricalMap subworkflow have the *F*_1_ population structure as an assumption. In this current version, they can be applied to another type of sequencing library but not to another type of population structure. For these steps, it is just important that the VCF file format is standardized and can be processed by BCFtools. They do not need to be necessarily from the SNP call software implemented. They can be also a combination of VCFs from different software such as the common markers between the implemented SNP call software results (“intersect” in Fig. 2).

We had to perform extra manipulations in TASSEL VCF output to be able to run the downstream analysis because they presented missing header information. Also, processing Freebayes showed the consumption of an unexpectedly high amount of RAM memory in some situations, which made it impossible to automatize the amount of memory required from the HPC and Cloud by the workflow task.

The number of markers identified by each software is related to the species, library preparation, and sequencing aspects such as genome size, restriction enzyme used, and sequencing depth. In Fig. 2, we can observe that more markers were identified in the aspen dataset compared to the roses dataset due to the higher frequency of enzyme cut sites. There is no consistency between the 2 datasets about which software identifies the higher number of markers.

After all the filtering steps and linkage map building, it is consistent that Freebayes keeps more markers. However, the resulting maps built with Freebayes markers, genotypes, and genotypes probabilities presented higher genetic distances inflation compared to the other approaches. Using TASSEL software markers also resulted in higher inflation in aspen dataset maps, which have a lower sequencing depth (∼6×) compared to the roses dataset (∼94×). The other approaches also presented outlier markers that inflate the total map size, but because they are individual markers, they can be easily removed in further steps. The maps built with only common markers among all 4 software (intersection in Fig. 2) contained fewer markers and had markers distances similar to GATK and Stacks results.

Evaluating the results of our simulations for GATK, we identified a format characteristic of VCFs from this software that leads to genotyping errors in estimations by updog, polyRAD, and SuperMASSA. In such cases, the genotype is considered missing in the GATK output VCF GT format field, while the total read depth is always reported in the reference allele field of the AD format field (e.g., Estimated = GT:AD ./.;22,0 | True = GT:AD 1/1;0,22).

**Figure 3:**
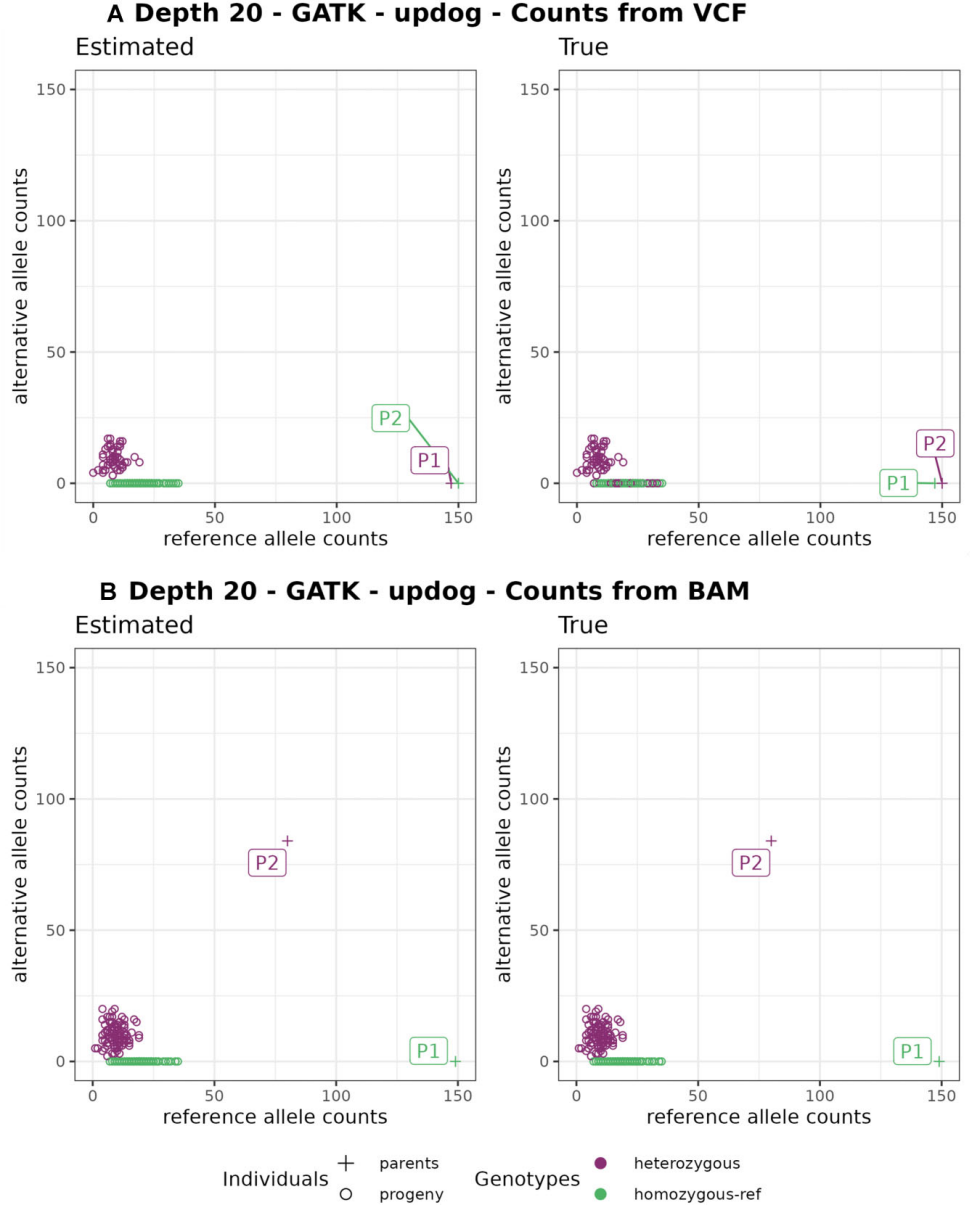
Example of error (Est: homozygous | True: heterozygous and Est: heterozygous | True: homozygous) in parental genotypes leading to a wrong marker type (Est: D1.10 | True: D2.15). Estimated reference (x-axis) and alternative (y-axis) allele count. Graphics on the left have colors according to estimated genotypes and on the right to the true genotypes. (A) Counts from the GATK VCF file and (B) from the BAM file. In the VCF file outputted by GATK, the P1 genotype is missing (GT./.) because the reads did not pass the quality filters, but it reports the counts in the reference AD field (149,0). The updog software use progeny segregation (1:1) to estimate the parents, but it makes a mistake identifying which one is heterozygous. Using counts from the BAM file (B) fixes this issue despite losing the GATK quality filters that can be important in other situations.

**Figure 4:**
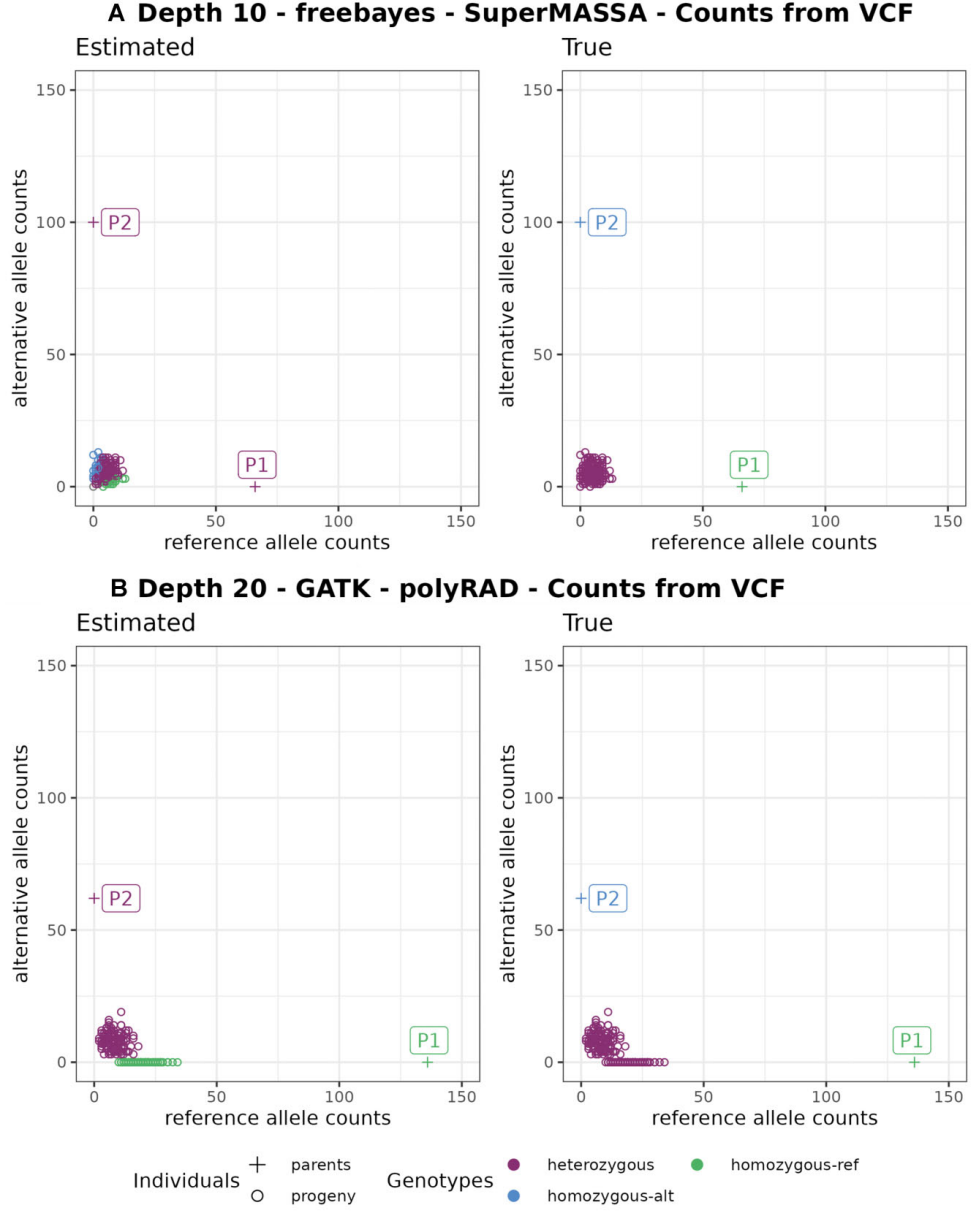
Example of error (Est: homozygous | True: heterozygous) in progeny genotypes leading to wrong marker types in (A) Est: B3.7 | True: noninformative and in (B) Est: D1.10 | True: noninformative. Graphics on the left have colors according to estimated genotypes and on the right to the true genotypes.

We present examples of the consequences of this format in genotypes called by updog, polyRAD, and SuperMASSA in Figs. 3 and 4. In Fig. 3A, allele dropouts are observed in the genotype of parent P2 and some of the progeny individuals. In empirical data, allele dropout can occur due to various reasons, such as polymorphisms in the cut site or the nonamplification of 1 allele during the PCR step [9]. Our simulations also consider allele dropout, but in the observed scenario, the source of allele dropout is due to the format characteristic of the GATK VCF file.

The occurrence of genotyping errors while using GATK VCF allele counts was previously observed by [59], who suggested using counts from BAM alignment files to address the issue (Fig. 3B). However, when testing the usage of BAM allele counts, we lose the advantage of the robust filtering applied by the GATK pipeline to retain only high-quality read counts in its VCF allele depth field. To maintain the accuracy of the GATK allele depth while overcoming the common error observed when the genotype is missing, we replaced the VCF allele count (AD and DP fields) with zero when the genotype information is missing before utilizing it for genotyping with polyRAD, SuperMASSA, and updog. This more precise way of solving the issue was only possible due to our simulation studies once they provide a clear comparison between simulated (true) and estimated data that highlighted the sources of the genotyping errors.

We also observed situations in updog, polyRAD, and SuperMASSA results where the parental genotypes are wrongly estimated because of the low quality of the progeny genotypes that distort the expected segregation. These genotype call software consider the expected segregation in their models, and therefore errors in the progeny lead to errors in the parents. Figure 4 shows examples where the marker would be considered noninformative for an outcrossing population, as both parents are homozygous. However, due to genotyping errors in the population, SuperMASSA and polyRAD incorrectly estimate the parents as heterozygous. To tackle this problem, we implement a filtering step to exclude noninformative markers before applying the genotype callers.

Solving these issues was particularly important because erroneous parent genotypes have a higher impact on linkage map quality than progeny genotype errors. OneMap 3.0 does not consider the parental genotype probabilities in its HMM multipoint approach. Thus, it is important to plan the sequencing experiment with high-quality parental genotypes because, if there are errors, they will not be corrected in downstream processing, and it will cause distortions in the resulting distances and haplotypes. To avoid map size inflation, erroneous parental genotypes must be removed before the linkage map analysis.

In general, the evaluation of the RADinitio simulations profile shows that we can expect fewer markers and genotyping errors in the simulated compared to the empirical data (Supplementary Fig. S7). A smaller number of markers should not reduce the built linkage map quality because the analysis was made in *F*_1_ populations, which have large disequilibrium blocks. However, the smaller number of genotyping errors overestimates the SNP and genotype calling software efficiency. This overestimation is commonly observed in simulation results once the data cannot capture all biases and errors in the empirical data. Thus, we used the simulations to understand specific software limitations and error sources but not ultimately define the best performance [62].

We observed the same or improved quality of linkage maps in the empirical dataset evaluations (Supplementary Fig. S8) when we applied these 2 described filtering steps: removing noninformative data before genotype calling and replacing allele counts with missing data when the genotype is missing in the GATK calls. After the genotype calling, we applied a threshold of 0.8 to filter low-quality genotypes, which also was beneficial in all scenarios. It is important to notice that these filters are applied before the segregation test filter, which reduces the number of tests and increases the permissibility of the threshold corrected by multiple tests (Bonferroni correction). Thus, the built map can have more markers in some scenarios even if more filters are applied.

The simulations were also useful to validate all code developed for the analysis and to measure the effects of segregation distortion. The results showed that the segregation distortion does not affect the frequency of correct estimated genotypes in most scenarios, despite affecting the reliability of the genotype probabilities provided by updog, SuperMASSA, and polyRAD (Supplementary Figs. S9 and S10). This can be one of the reasons why using genotype probabilities in the HMM did not present consistent results across tested datasets.

Despite considering the HMM error rate dataset-dependent values, we identified that some of the possible values can be discarded. Using the OneMap default value of 10^−5^, global error rate produced bad-quality maps in all situations. The same happened while using all the genotype call software relative error. Using higher values of global error rate and genotypes from GATK, Freebayes, TASSEL, Stacks, updog, and polyRAD, or the combination of the genotype probability and a global error rate from software GATK, updog, Stacks, and polyRAD, produced the most reliable linkage maps, with linkage map sizes closer to the expected.

**Figure 5:**
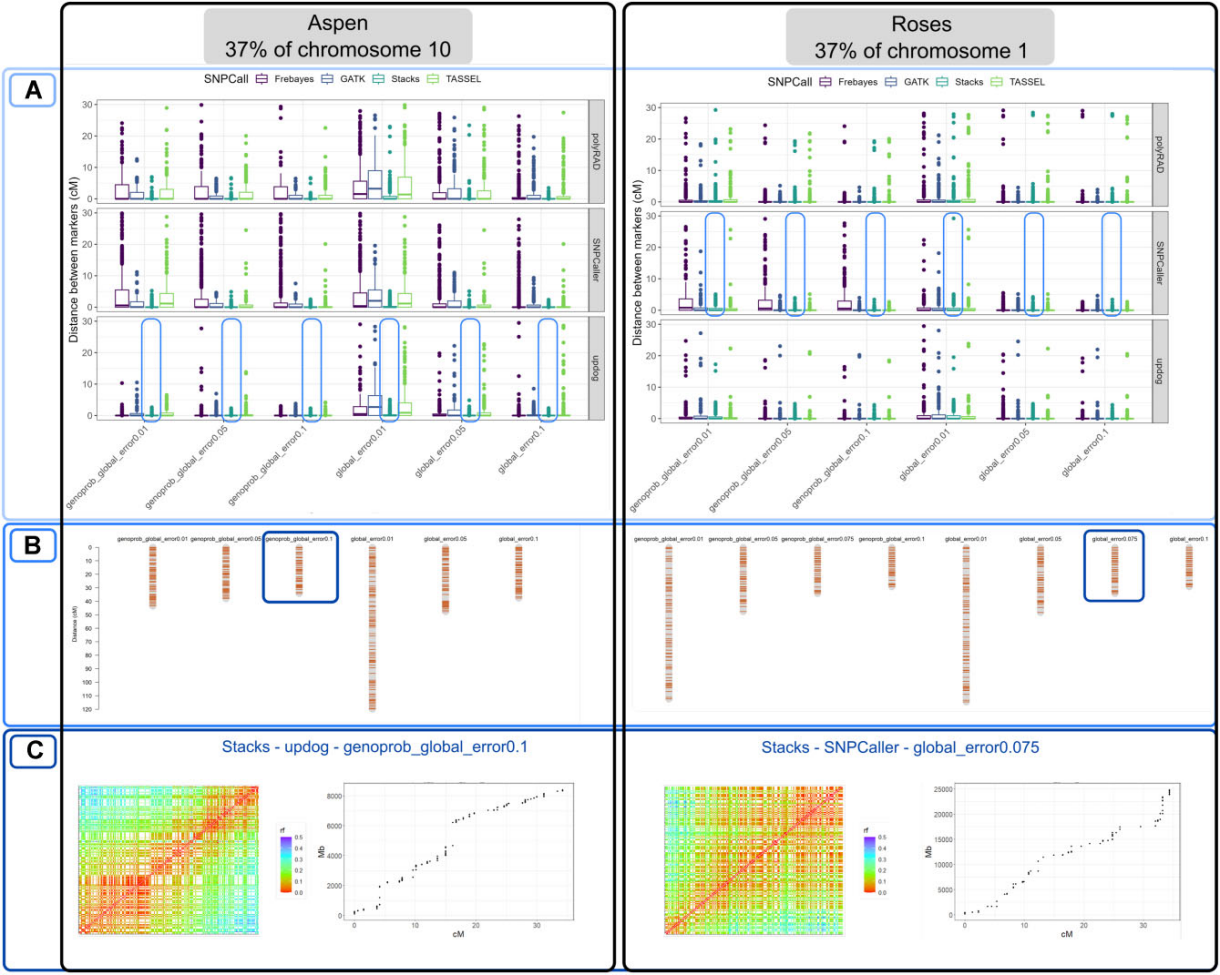
Process of selecting the best pipeline. (A) Comparing the effect of different error probabilities in the OneMap 3.0 HMM in the distances between adjacent markers. (B) Comparing the effect of different error probabilities in the linkage maps total size built with a single SNP call software. (C) Checking the recombination fraction (rf) heatmap and markers coverage in the genome using the selected pipeline. These figures were extracted from Reads2MapApp.

As observed in Fig. 5, many of the approaches produced linkage maps with distances between all adjacent markers smaller than 10 cM. We chose the method that results in less inflated linkage maps and outlier markers even when applying the small values of the global error rate (0.01). Once the method was selected, we tried an intermediary global error rate (0.075) for the roses dataset values to adjust to the expected total size. We also checked the recombination fraction heatmap, the markers coverage, density, and the number of estimated recombination breakpoints in progeny through Reads2MapApp figures (see the app interface demonstration in Supplementary File S2).

**Figure 6:**
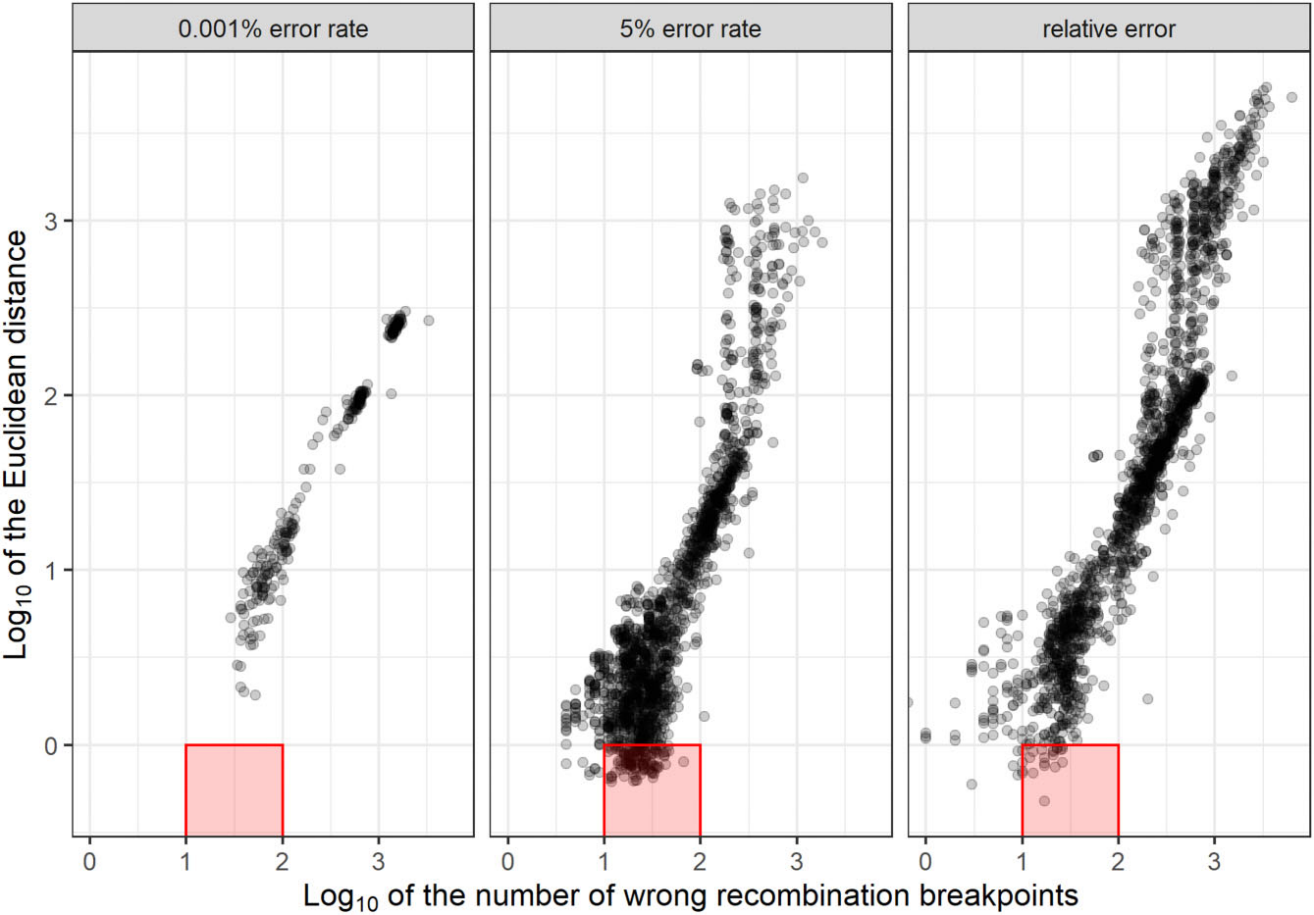
Relation between Euclidean distance (y-axis) and the number of recombination breakpoints (x-axis) in maps built with global error rates (0.001% and 5%) and with probabilities outputted by the genotype call software (relative error). Each dot represents a map built with simulated data based on the first 37% of aspen chromosome 10. The red squares highlight maps that do not present inflated size (1 or less Euclidean distance) but have from 10 to 100 wrong recombination breakpoints.

Before using the map size as a metric for map quality, we checked if a map with the expected size always means good quality. A map can have the expected size but a poor quality if the number of overestimated and underestimated recombination breakpoints in the progeny haplotypes is the same—in other words, if they cancel out. To test if this happens in our simulated dataset, we compared the Euclidean relation of estimated and true genetic distances with the total number of wrong (overestimated + underestimated) recombination breakpoints in the progeny haplotypes (Fig. 6). For identifying a break as overestimated or underestimated, we do not consider the expected break position but the total breaks expected for the evaluated haplotype. For example, if 1 haplotype for a specific progeny was simulated with 1 break and estimated with zero, then we count it as 1 underestimated break.

The comparison shows that overestimated breakpoints are generally more frequent than underestimated ones. We observe that when a map is inflated, it also has many wrong recombination breakpoints. However, in some cases, the map has the expected map size but a high number of wrong haplotypes due to both overestimated and underestimated breaks. A high number of underestimated breaks can be observed in situations where the Euclidean distance is close to or less than 1 (*log*_10_0) and the number of wrong recombination events is between 10 and 100 (*log*_10_1 and *log*_10_2). These situations are more frequent when a global error rate of 5% is used.

In the empirical data results, we observed maps with expected size and excess recombination breakpoints in just a few individuals in the progeny. This variation can be related to contaminant samples. The study by Zhigunov et al. [47] identified 6 contaminants in the aspen dataset. When we ran the workflows, including the contaminant samples, the maps built with Freebayes markers and updog, SuperMASSA, and polyRAD were smaller in size than without the contaminant (Supplementary Fig. S11). This would (wrongly) suggest better quality if map size is the only metric used. Nevertheless, the maps presented higher differences in the number of recombination breakpoints among individuals when using the genotype probabilities relative to each genotype call software. Some contaminant samples presented more estimated recombination events than the rest of the progeny. Using higher values of global error reduces this difference and can mask the presence of contamination.

These results show that it is important to exclude contaminant samples before the linkage map building once the multipoint HMM approach tends to fix the genotypes according to the biological assumption that they are all *F*_1_ individuals. There are several methods available for identifying contaminant samples in previous steps. The ADMIXTURE [63] software analysis as made by Zhigunov et al. [47] is one possibility. Another is to calculate a marker-based relationship matrix using the R package AGHmatrix [64].

So far, all the evaluations we have discussed have focused exclusively on biallelic markers. We also evaluate the impact on the genetic distances when haplotype-based multiallelic markers are included. In most of the tested scenarios, incorporating these markers leads to map inflation. This is primarily due to the fact that inaccurately estimated multiallelic markers or genotyping errors associated with them can significantly affect the quality of the linkage map. The impact is particularly pronounced because multiallelic markers provide richer information, including recombination and phase information for both parents, compared to biallelic markers. However, the advantages of including the multiallelic markers appear in the marker ordering step.

Algorithms that use 2-point recombination fractions estimations have issues ordering only biallelic markers because of the missing linkage information between markers D1 and D2 (homozygous × heterozygous or vice versa). These markers can only be related to each other in the presence of more informative markers, such as B3.7 (heterozygous × heterozygous) or multiallelic states. Yet, having few B3.7 markers compared to D1 and D2 can still be an issue for linkage map building. In fact, this characteristic was the reason behind the initial development of separate maps for each parent in the first methods used for building genetic maps in such populations [65]. These nonintegrated genetic maps subsequently limited further analysis of multiallelic traits in terms of QTL mapping [66].

The markers ordering efficiency is not considered by Reads2Map workflows once it uses the genomic order to position the markers in the linkage maps. The reference genome is a required input by the workflows to standardize the positions of the markers across all tested methods. This avoids the confounding interpretation of bad-quality linkage maps due to wrong ordering and not genotyping errors.

**Figure 7:**
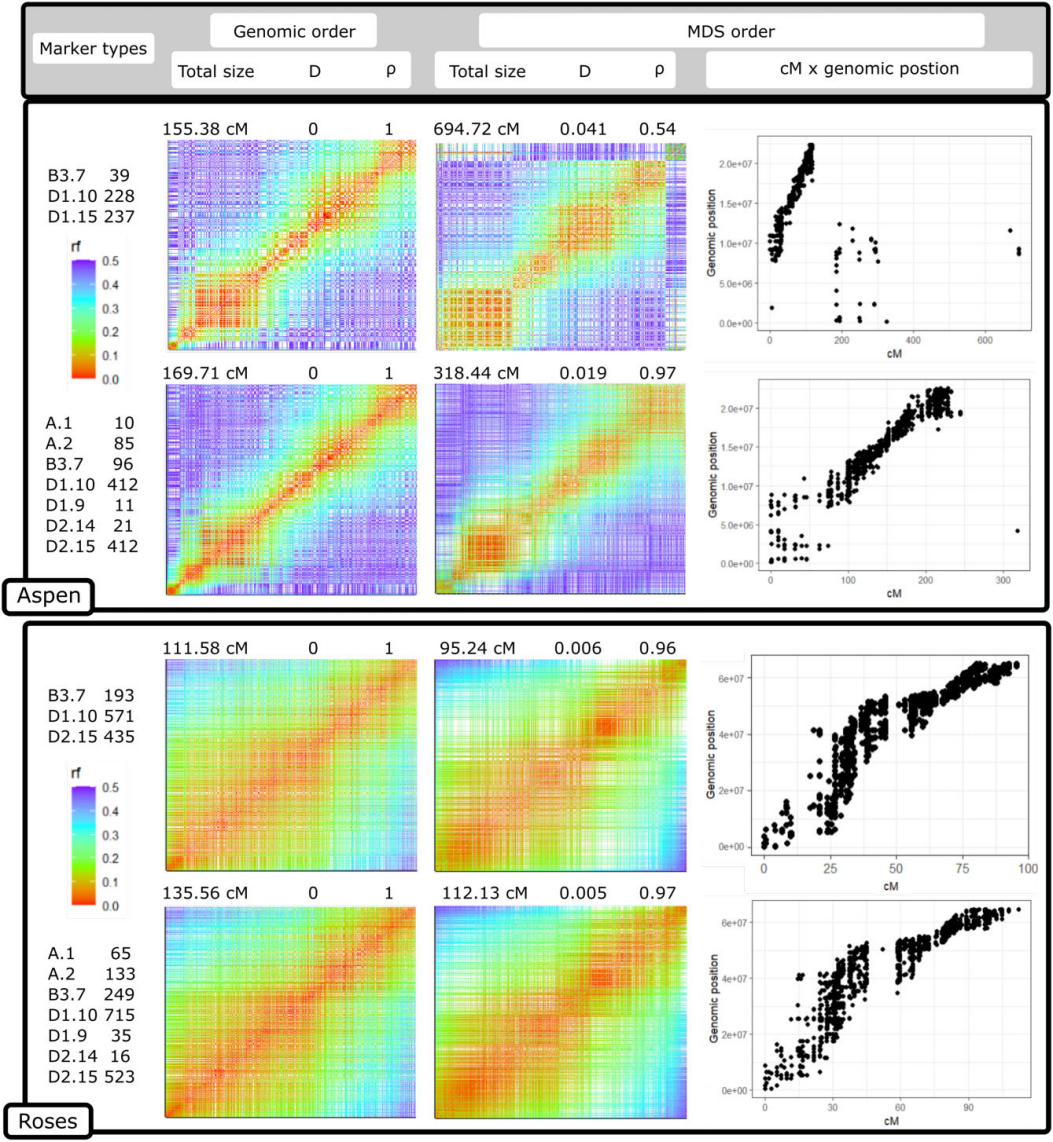
Comparison between MDS ordering algorithm performance in the aspen and roses dataset entire linkage group 10 and 1, respectively, with only biallelic markers and with biallelic and haplotype-based multiallelic markers estimated by Stacks. The heatmaps represent the recombination fraction (rf) matrix between markers positioned at both axes. In well-ordered linkage groups, we expect a gradient from hot colors in the diagonal (adjacent markers) to cold colors in the upper left and lower right corners. The figure also presents the Spearman rank correlation (ρ) and the Euclidean distances (D) between the estimated map using MDS and the map built with markers ordered by the genomic positions (used as reference). The dot plots relate the positions of markers estimated by MDS with the genomic position.

To test the effect of multiallelic markers in the ordering, we built a linkage map for the entire chromosomes 1 and 10 of the roses and aspen datasets, respectively, using the selected methods and adding the haplotype-based multiallelic markers provided by the Stacks population plugin. We used the OneMap wrapper function mds_onemap to order the markers with MDS [60]. The genetic distances were estimated by the HMM multipoint approach. Figure 7 shows the effects of including the multiallelic markers in the 2-point-based MDS algorithm.

The impact of multiallelic markers differed between the aspen and roses datasets. In the aspen dataset, characterized by a lower depth and a higher rate of genotyping errors in the markers, most of the B3.7 biallelic markers were filtered out during previous steps, resulting in an unsatisfactory performance of the MDS algorithm in ordering the markers. However, incorporating the multiallelic markers, although slightly inflating the genetic distances, significantly improved the ordering accuracy using MDS. It should be noted that MDS itself can contribute to genetic distance inflation as it may erroneously invert markers in close proximity. In scenarios where a reference genome is unavailable, the inclusion of multiallelic markers can prove valuable for effective marker ordering in these types of datasets.

The roses dataset is characterized by higher-quality markers, and the genomic ordering can be almost entirely reproduced using only biallelic markers. In this scenario, the inclusion of multiallelic markers also leads to a slight inflation of the map size while improving the ordering accuracy through MDS. Unlike the aspen dataset, the MDS algorithm in the roses dataset tends to reduce the genetic distances, resulting in an underestimation of recombination breakpoints. However, considering that there are no significant inversions or translocations (see dot plots in Fig. 7), we can have more confidence in the genomic order, even if the map is larger. Any discrepancies between the MDS-based order and the genomic order are likely attributed to local changes, which are likely to be errors introduced by MDS.

## Final Considerations

The Reads2Map workflows have a robust structure to generate production-level results with simple inputs and optimized usage of computational resources. The structure allowed us to test the quality of genetic maps built with the following scenarios: (i) using different SNP calling software (GATK, TASSEL, Stacks, and Freebayes), (ii) using different genotype calling software (GATK, Freebayes, TASSEL, Stacks, updog, polyRAD, SuperMASSA), (iii) using different linkage map-building software (OneMap 3.0 and GUSMap), (iv) establishing different error probabilities (relative to genotype call software; 10%, 1%, 5%, and 0.001% global error; and the combination of the global error rate with the genotype call probabilities), (v) applying different marker filtering (vi) with or without multiallelic markers, (vi) in empirical and simulated data, (vii) with and without segregation distortion, (viii) with and without contaminant samples, (ix) with different GBS library preparation aspects, and (x) with different sequencing depths. These scenarios are commonly found by researchers trying to produce high-quality linkage maps using sequencing technologies. The Reads2Map and Reads2MapApp are the first tools to guide best practices for building linkage maps with sequencing data pointing software, parameters, and marker filters to be used in diverse scenarios.

We elaborated and limited the scenarios explored according to our experiences as developers of OneMap. OneMap first version was released in 2007, and since then, it has been used to build linkage maps in a diversity of species. Its strategies and structure also served as a base for more complex software such as MAPpoly [15] for building linkage maps in polyploid species. With time, new methods for genetic marker identification using sequencing data emerged, changing the context where OneMap was used. We included updates in this version 3.0 to resolve issues with inflated genetic maps and marker ordering. Two major changes allow users to read and build genetic maps with the genotype probabilities and haplotype-based multiallelic markers information from the input files (OneMap format or VCF file). However, the success of genetic map building will be proportional to the quality of the information provided by upstream procedures such as library preparation, SNP and genotype calling, genotype probabilities estimation, and the combination of SNPs into haplotype-based markers. With Reads2Map and Reads2MapApp, we provide users tools to select the best approaches before using OneMap 3.0 to guarantee that it will result in the best-quality genetic map possible with the data available.

It is important to highlight that we did not design the workflows to be a tool to build a final linkage map but to select the bioinformatic pipeline that provides the best-quality genetic markers. Once the pipeline is selected, the respective VCF file and OneMap functions can be used in the R environment to build the final map. Building the complete linkage map will require evaluations and edits that are highly specific and cannot be fully automated within the workflows. These tasks include addressing the presence of translocations and inversions, identifying outlier markers, and linkage between markers located in different chromosomes.

The diversity in the results of the pipeline suggested for both empirical datasets highlights that pipelines perform differently with datasets with different properties. This means that the pipelines presented here as the best cannot be considered the best for every dataset. We could reduce the number of required tests by users identifying the dataset-independent approaches and setting them as default in Reads2Map. However, we suggest users reproduce the tests presented here for the dataset-dependent approaches using the Reads2Map workflows with their empirical dataset and select the best pipelines for their specific conditions.

The workflows were built using WDL and containers to ensure high reproducibility. This guarantees that different results running different datasets are due to the dataset’s properties and not to bioinformatic pipeline changes. Also, updates can be easily made in the workflows as the software implemented are improved once the versions are controlled by Docker images. This makes Reads2Map also a useful tool for software developers to validate updates because it facilitates checking the consequences of the changes in the quality of the markers by easily controlling versions, rerunning datasets, and checking the map quality.

Every Reads2Map workflow run returns a large amount of information. Every step of the workflow, from the reads’ alignment to the completed linkage map, provides quality measurements for users to evaluate each scenario. The Reads2MapApp shiny app receives all this information compressed in a single workflow output file and converts it into comprehensive interactive graphics. Through the app interface, users can evaluate the performance of each combination of software and parameters in each step. If results show issues in any of them, users can rerun the workflow with adapted parameters or include new filters that make sense in their context. Once the upstream steps are established based on the app graphics for the built linkage map subset, users can reproduce it for the complete dataset, inputting the VCF files from Reads2Map into OneMap.

## Availability of Source Code and Requirements

- Project name: Reads2Map
- Project homepage: [32]
- Main workflows: EmpiricalReads2Map [33] and SimulatedReads2Map [34]
- Operating system(s): Platform independent
- Programming language: WDL
- Other requirements: Docker or singularity
- License: MIT
- RRID: SCR_023593
- biotoolsID: reads2map

## Supplementary Material

giad092_GIGA-D-22-00323_Original_Submission

giad092_GIGA-D-22-00323_Revision_1

giad092_GIGA-D-22-00323_Revision_2

giad092_GIGA-D-22-00323_Revision_3

giad092_Response_to_Reviewer_Comments_Original_Submission

giad092_Response_to_Reviewer_Comments_Revision_1

giad092_Response_to_Reviewer_Comments_Revision_2

giad092_Reviewer_1_Report_Original_Submission Peter M. Bourke, Ph.D. -- 3/23/2023 Reviewed

giad092_Reviewer_1_Report_Revision_1 Peter M. Bourke, Ph.D -- 8/4/2023 Reviewed

giad092_Reviewer_2_Report_Original_Submission Ramil Mauleon -- 3/23/2023 Reviewed

giad092_Reviewer_2_Report_Revision_1 Ramil Mauleon -- 8/9/2023 Reviewed

giad092_Reviewer_3_Report_Original_Submission Zhenbin Hu -- 3/27/2023 Reviewed

giad092_Reviewer_3_Report_Revision_1 Zhenbin Hu -- 7/2/2023 Reviewed

giad092_Supplemental_File

## Data Availability

An archival copy of the code and supporting data is available via the *GigaScience* repository, GigaDB [67].Workflows are also archived at workflowhub.eu [33, 34].

## Additional Files

**Supplementary File S1**. Emission function for outcrossing.

**Supplementary File S2**. Reads2MapApp interface demonstration.

**Supplementary Fig. S1**. Reads2MapApp about page. The red arrow indicates the menu icon to access the app’s other pages.

**Supplementary Fig. S2**. Reads2MapApp upload page. The red arrow indicates the button to upload the EmpiricalMaps workflow results.

**Supplementary Fig. S3**. Example of the “SNP calling efficiency” section. Venn diagrams are built to show the number of markers identified in the pipelines defined in the options and the common markers between them.

**Supplementary Fig. S4**. Example of the “Map size” section of Reads2MapApp. The graphic shows the number of markers (left) and the distances between adjacent markers (right) for each method.

**Supplementary Fig. S5**. Example of the “Breakpoint count” section of Reads2MapApp. The graphic shows the number of estimated breakpoints in each progeny haplotype according to the method selected in the options.

**Supplementary Fig. S6**. Example of the “Progeny haplotypes” section of Reads2MapApp. The graphic shows the estimated haplotype for the individuals selected in the “Individuals from progeny” options according to the method selected in the options.

**Supplementary Fig. S7**. Venn diagrams show the number of markers identified by freebayes, GATK, and simulated (true). The intersection between the datasets represents markers with the same position in the reference genome *Populus trichocarpa* version 3.0. The empirical datasets include markers spread across the entire reference genome. The simulations only include markers in the first 8.426 Mb of chromosome 10 (2.1% of the genome). The mean and standard deviation of number markers are shown for the simulated dataset once the simulation and SNP calling are repeated 60 times. Markers were filtered by 25% maximum missing data and MAF 5% in empirical and simulated data. *Number of markers common to all 60 repetitions.

**Supplementary Fig. S8**. The relation between filters applied (x-axis), the map size (A, y-axis), and the number of markers (B, y-axis) for genotype calling software used in the empirical datasets. The datasets shown in the figure contain only biallelic markers. The horizontal red line indicates the expected map size (38 cM) for the subset of the genomes used.

**Supplementary Fig. S9**. ROC curves with the true and estimated genotypes from the 5 families simulated with mean depth 10 and 20 and with the first 8.426 Mb of chromosome 10 (37% or 38 cM). Here, only biallelic markers are considered. The specificity and sensitivity profiles consider different thresholds in the genotype probabilities for each scenario. The higher the area under the curve, the higher the genotype’s probability reliability. Genotype probability thresholds closer to the left superior corner have a higher capacity to differentiate right and wrong genotypes.

**Supplementary Fig. S10**. See Supplementary Fig. S9 description.

**Supplementary Fig. S11**. Effect of contaminant samples in the map size (A) and in the number of estimated recombination breakpoints range (B) among progeny individuals. The empirical aspen datasets presented in this figure contain multiallelic markers and the allele counts from the VCF file, and they are filtered by genotype probability higher than 0.8 to keep only informative markers.

**Supplementary Table S1**. Emission function values according to marker types. The error rate is represented by *e*, unphased genotypes as “a,” “b,” “c,” “d,” and their combination. The estimated phased genotypes are represented by “AA,” “AB,” “BA,” and “BB.” The “o” represents null alleles. Marker types follow the segregation pattern as described in Wu et al. [53].

**Supplementary Table S2**. Continued from Supplementary Table S1.

**Supplementary Table S3**. Continued from Supplementary Table S1.

**Supplementary Table S4**. Continued from Supplementary Table S1.

**Supplementary Table S5**. Continued from Supplementary Table S1.

**Supplementary Table S6**. Continued from Supplementary Table S1.

**Supplementary Table S7**. Continued from Supplementary Table S1.

**Supplementary Table S8**. List of third-party software and images versions used.

## Abbreviations

CPU: central processing unit; DNA sequencing; EM: expectation–maximization; GBS: genotyping-by-sequencing; GT: genotype; GQ: genotyping quality; GWAS: genome-wide association; HMM: hidden Markov model; HPRC: high-performance research computing; LD: linkage disequilibrium; MAF: minor allele frequency; NGS: next-generation sequencing. PCR: polymerase chain reaction; QTL: quantitative trait loci; RADSeq: restriction site associated; SNP: single-nucleotide polymorphism; VCF: variant call format; WDL: Workflow Description Language.
