# Supplementary material for: Developing best practices for genotyping-by-sequencing analysis in the construction of linkage maps
Source: Gigascience. 2023 Oct 27;12:giad092. doi: 10.1093/gigascience/giad092 (PMC10603770; doi:10.1093/gigascience/giad092)
Supplement: giad092_GIGA-D-22-00323_Original_Submission [file giad092_giga-d-22-00323_original_submission.pdf]

GigaScience

Developing best practices for genotyping-by-sequencing analysis using linkage maps as benchmarks

--Manuscript Draft--

**Manuscript Number:**	GIGA-D-22-00323
**Full Title:**	Developing best practices for genotyping-by-sequencing analysis using linkage maps as benchmarks
**Article Type:**	Technical Note
**Funding Information:**	National Institute of Food and Agriculture (2020-51181-32156) — Dr David Byrne<br>Bill and Melinda Gates Foundation (OPP1213329) — Dr Marcelo Mollinari<br>HORIZON EUROPE Marie Sklodowska-Curie Actions (801215) — Dr Thiago de Paula Oliveira<br>Conselho Nacional de Desenvolvimento Científico e Tecnológico (313269/2021-1) — Dr Antonio Augusto Franco Garcia

Funder	Recipient
National Institute of Food and Agriculture (2020-51181-32156)	Dr David Byrne
Bill and Melinda Gates Foundation (OPP1213329)	Dr Marcelo Mollinari
HORIZON EUROPE Marie Sklodowska-Curie Actions (801215)	Dr Thiago de Paula Oliveira
Conselho Nacional de Desenvolvimento Científico e Tecnológico (313269/2021-1)	Dr Antonio Augusto Franco Garcia

Abstract:

Background: Genotyping-by-Sequencing (GBS) provides affordable methods for genotyping hundreds of individuals using millions of markers. However, this challenges bioinformatic procedures that must overcome possible artifacts such as the bias generated by PCR duplicates and sequencing errors. Genotyping errors lead to data that deviate from what is expected from regular meiosis. This, in turn, leads to difficulties in grouping and ordering markers resulting in inflated and incorrect linkage maps. Therefore, genotyping errors can be easily detected by linkage map quality evaluations.

Results: We developed and used the Reads2Map workflow to build linkage maps with simulated and empirical GBS data of diploid outcrossing populations. The workflows run GATK and freebayes for SNP calling and updog, polyRAD, and SuperMASSA for genotype calling, and OneMap and GUSMap to build linkage maps. Using simulated data, we observed which genotype call software fails in identifying common errors in GBS sequencing data and proposed specific filters to better handle them. We tested whether it is possible to overcome errors in a linkage map using genotype probabilities from each software or global error rates to estimate genetic distances with an updated version of OneMap. We also evaluated the impact of segregation distortion, contaminant samples, and haplotype-based multiallelic markers in the final linkage maps. The results showed a low impact of segregation distortion in the linkage map quality, improvements in ordering markers with haplotype-based multiallelic markers, and improved maps with expected size using reliable genotype probabilities or a global error rate of 5%.

Conclusions: The pipelines results in each scenario changed according to the data set used, indicating that optimal pipelines and parameters are dataset-dependent and cannot be generalized to all GBS data sets. The Reads2Map workflow can reproduce the analysis in other GBS empirical data sets where users can select the pipeline and parameters adapted to their data context. The Reads2MapApp shiny app provides a graphical representation of the results to facilitate their interpretation.

**Corresponding Author:**	Cristiane Hayumi Taniguti<br>Texas A&M University<br>College Station, Texas UNITED STATES
**Corresponding Author Secondary Information:**	
**Corresponding Author's Institution:**	Texas A&M University
**Corresponding Author's Secondary Institution:**	
**First Author:**	Cristiane Hayumi Taniguti
**First Author Secondary Information:**	

Powered by Editorial Manager® and ProduXion Manager® from Aries Systems Corporation

**Order of Authors:**	Cristiane Hayumi Taniguti
	Lucas Mitsuo Taniguti
	Rodrigo Rampazo Amadeu
	Jeekin Lau
	Gabriel de Siqueira Gesteira
	Thiago de Paula Oliveira
	Getulio Caixeta Ferreira
	Guilherme da Silva Pereira
	David Byrne
	Marcelo Mollinari
	Oscar Riera-Lizarazu
	Antonio Augusto Franco Garcia
**Order of Authors Secondary Information:**	
**Additional Information:**	
**Question**	**Response**
Are you submitting this manuscript to a special series or article collection?	No
**Experimental design and statistics**<br><br>Full details of the experimental design and statistical methods used should be given in the Methods section, as detailed in our Minimum Standards Reporting Checklist. Information essential to interpreting the data presented should be made available in the figure legends.<br><br>Have you included all the information requested in your manuscript?	Yes
**Resources**<br><br>A description of all resources used, including antibodies, cell lines, animals and software tools, with enough information to allow them to be uniquely identified, should be included in the Methods section. Authors are strongly encouraged to cite Research Resource Identifiers (RRIDs) for antibodies, model organisms and tools, where possible.	Yes

Have you included the information requested as detailed in our Minimum Standards Reporting Checklist?	
**Availability of data and materials**<br><br>All datasets and code on which the conclusions of the paper rely must be either included in your submission or deposited in publicly available repositories (where available and ethically appropriate), referencing such data using a unique identifier in the references and in the "Availability of Data and Materials" section of your manuscript.<br><br>Have you have met the above requirement as detailed in our Minimum Standards Reporting Checklist?	Yes

Powered by Editorial Manager® and ProduXion Manager® from Aries Systems Corporation

Main manuscript

Click here to access/download;LaTeX - Main Document (TeX file);main.tex

OXFORD (GIGA)n SCIENCE

GigaScience, 2017, 1–24

doi: xx.xxxx/xxxx
Manuscript in Preparation

Developing best practices for genotyping-by-sequencing analysis using linkage maps as benchmarks

Cristiane Hayumi Taniguti[1,2,*], Lucas Mitsuo Taniguti[1,3], Rodrigo Rampazo Amadeu[1], Jeekin Lau[2], Gabriel de Siqueira Gesteira[1,4], Thiago de Paula Oliveira[5], Getulio Caixeta Ferreira[1], Guilherme da Silva Pereira[7], David Byrne[2], Marcelo Mollinari[4], Oscar Riera-Lizarazu[2] and Antonio Augusto Franco Garcia[1,*]

[1]Department of Genetics, University of São Paulo, Brazil and [2]Department of Horticultural Sciences, Texas A&M University, College Station, TX, USA and [3]Mendelics Genomic Analysis, São Paulo, Brazil and [4]Bioinformatics Research Center, Department of Horticultural Sciences, North Carolina State University, Raleigh, NC, USA and [5]Roslin Institute, University of Edinburgh, Scotland and [7]Department of Agronomy, Federal University of Viçosa, Brazil

[*]chtaniguti@tamu.edu; augusto.garcia@usp.br

Abstract

Background Genotyping-by-Sequencing (GBS) provides affordable methods for genotyping hundreds of individuals using millions of markers. However, this challenges bioinformatic procedures that must overcome possible artifacts such as the bias generated by PCR duplicates and sequencing errors. Genotyping errors lead to data that deviate from what is expected from regular meiosis. This, in turn, leads to difficulties in grouping and ordering markers resulting in inflated and incorrect linkage maps. Therefore, genotyping errors can be easily detected by linkage map quality evaluations.
Results We developed and used the Reads2Map workflow to build linkage maps with simulated and empirical GBS data of diploid outcrossing populations. The workflows run `GATK` and `freebayes` for SNP calling and `updog`, `polyRAD`, and `SuperMASSA` for genotype calling, and `OneMap` and `GUSMap` to build linkage maps. Using simulated data, we observed which genotype call software fails in identifying common errors in GBS sequencing data and proposed specific filters to better handle them. We tested whether it is possible to overcome errors in a linkage map using genotype probabilities from each software or global error rates to estimate genetic distances with an updated version of `OneMap`. We also evaluated the impact of segregation distortion, contaminant samples, and haplotype-based multiallelic markers in the final linkage maps. The results showed a low impact of segregation distortion in the linkage map quality, improvements in ordering markers with haplotype-based multiallelic markers, and improved maps with expected size using reliable genotype probabilities or a global error rate of 5%.
Conclusions The pipelines results in each scenario changed according to the data set used, indicating that optimal pipelines and parameters are dataset-dependent and cannot be generalized to all GBS data sets. The Reads2Map workflow can reproduce the analysis in other GBS empirical data sets where users can select the pipeline and parameters adapted to their data context. The Reads2MapApp shiny app provides a graphical representation of the results to facilitate their interpretation.

Key words: genotyping error; haplotype; genetic maker; multiallelic

Compiled on: November 29, 2022.
Draft manuscript prepared by the author.

Introduction

Advances in sequencing technologies and the development of different genome-reduced representation library protocols result in millions of genetic markers from hundreds of samples in a single sequencing run [1, 2, 3, 4]. Increasing the number of markers and individuals genotyped can enhance the capacity of linkage maps to locate recombination events that occur, resulting in higher map resolution and better statistical power for the localization of QTL in further analysis. This large amount of data and genotyping errors common with genotyping-by-sequencing approaches [5] increases the need for computational resources and multiple bioinformatic tools.

Genotyping errors are frequent when high-throughput sequencing technology is applied to reduced representation libraries. There are a variety of protocols to create these types of libraries [4], called Restriction-site Associated DNA sequencing (RADseq) or genotyping-by-sequencing (GBS) [6, 7]. Generally, one or more restriction enzymes are used to digest the sample DNA. The resulting DNA fragments are filtered by size, connected to adaptors and barcodes, amplified by PCR, and sequenced. Consequently, most sequences obtained are PCR duplicates of the regions around the enzyme cut site. By relying on duplicates to increase sequencing depth, such methods introduce errors and a sequencing bias towards one of the alleles due to variabilities in the PCR amplification. These errors are hard to detect by bioinformatic tools [8, 9].

To overcome genotyping errors coming from GBS methods, genotype calling software model sequencing error, allelic bias, overdispersion, outlying observations, and the population Mendelian expected segregation [10]. Building a genetic map with genotypes obtained using these methods can be a powerful tool to validate their efficiency. Wrong decisions or inefficient methods in all steps before linkage map building can be identified in the resulting map as errors that dissociate the map properties from biological processes. For example, genotyping errors generate inflated map sizes that show an excessive number of recombination breakpoints during meiosis [11]. The first genetic map studies by Morgan and Sturtevant [12] discovered that crossing-overs are unlikely to happen too close to each other, a phenomenon named interference. Later studies describing the meiotic molecular mechanisms confirmed the low expected number of recombination breaks in a single event [13].

Recently developed approaches to build linkage maps [14, 15, 16] were implemented in `OneMap` [17] 3.0 package. They use quantitative genotype probability measurements rather than the traditional qualitative genotypic information from SNP and genotype calling methods to account for genotyping errors and provide higher-quality genetic maps. These probabilities can be applied in different ways: using the probability of each possible genotype (PL field in VCF format); using an error probability associated with the called genotype (GQ field in VCF format); or using a global error rate that will be applied to all genotypes. Nevertheless, even using these approaches, building a linkage map will succeed only if the upstream software can identify the errors and provide reliable genotypes or their probabilities.

The biallelic codominant nature of SNPs is another characteristic of high-throughput markers that can affect linkage map building of outcrossing species. Although biallelic markers can distinguish only two haplotypes, the mapping population of outcrossing diploid species inherits two haplotypes with combinations of four different parental haplotypes. With biallelic markers, the observed parental genotypes are limited to types $ab \times ab$, $ab \times aa$, and $aa \times ab$. When one of the parents is homozygous ($ab \times aa$ and $aa \times ab$), it is impossible to observe the crossing-over change for this uninformative parent. So this is taken as missing information (non-measurable crossing-overs) for linkage map building if only two-point information is considered. Therefore, building a linkage map with only biallelic markers requires a multi-point approach that uses loci information with both parents heterozygous ($ab \times ab$) to estimate the recombination of loci where one parent is homozygous, and the recombination information is missing for closely linked loci. The multi-point approach applies likelihood computations involving several loci and has been successfully used since the seminal publication of Lander and Green [18]. The approach makes it possible to identify the four different parental haplotypes by phasing the biallelic information so that the SNPs can be used to identify all the allelic diversity.

Other approaches to overcome the low informativeness of biallelic markers involve combining adjacent biallelic markers in the same disequilibrium block (high LD) into a single multiallelic haplotype. These haplotype-based markers showed higher accuracy in association analysis than individual biallelic SNPs [19, 20, 21, 22, 23, 24, 25]. N'Diaye et al. [21] and Jiang et al. [25] pointed out several advantages of haplotype-based markers, including the higher capacity to identify epistatic interactions, the presence of more information to estimate identical-by-descent alleles and the reduction of the number of statistical tests to perform.

Despite many software available for estimating genotype probabilities [26, 2, 27, 26, 28, 29, 10] and haplotype-based multiallelic markers [26, 30], there are no recommendations yet about which combination and choice of parameters are the best for building linkage maps. Therefore, this work evaluates the consequences of building maps by applying genotype probabilities and haplotype-based markers from different software and parameters. To achieve these, we implemented new features in `OneMap` [17], a widely-used software for building maps, and developed the `Reads2Map` workflow. We were able to make recommendations to users to obtain better linkage maps in several situations, such as low and high-depth sequencing, with and without segregation distortion, contaminant samples, and multiallelic markers, and using different bioinformatic software to perform the SNP and genotype calling.

Material and Methods

We built two workflows using Workflow Description Language (WDL) [31] to perform sequence alignment, SNP and genotype calling, and linkage map building: EmpiricalReads2Map, for evaluating empirical (real) data sets; and SimulatedReads2Map, to evaluate simulated data sets (figure 1). Both share the same sub-workflows for most of the steps, allowing users to evaluate software and parameters in an organized and efficient way. WDL workflows can be executed using Cromwell Execution Engine [31], Docker [32], and Singularity [33] containers. We ran the analysis testing workflows on two high-performance computers (Texas A&M University HPRC, University of São Paulo Águia Cluster). The CPU and memory amount utilized by each workflow task in the Texas A&M HPRC is shown in Supplementary figures 1-4. The workflows are available at https://github.com/Cristianetaniguti/Reads2Map. For the linkage map building step, we implemented updates in `OneMap` package version 3.0 (https://CRAN.R-project.org/package=onemap) and used this version in the workflows. We also developed the Reads2MapApp shiny app (https://github.com/Cristianetaniguti/Reads2MapApp). We used it to upload the final workflow output and visualize summary statistics about the resulting linkage maps, intermediary steps, and workflow performance.

Genotype probabilities in `OneMap` 3.0 Hidden Markov Model

With a combination of a hidden Markov model (HMM) and the expectation-maximization algorithm (EM) [18], `OneMap` [17] can perform multipoint estimation of map genetic distance for F2, backcross, RILs, and outcrossing populations. For the multipoint esti-

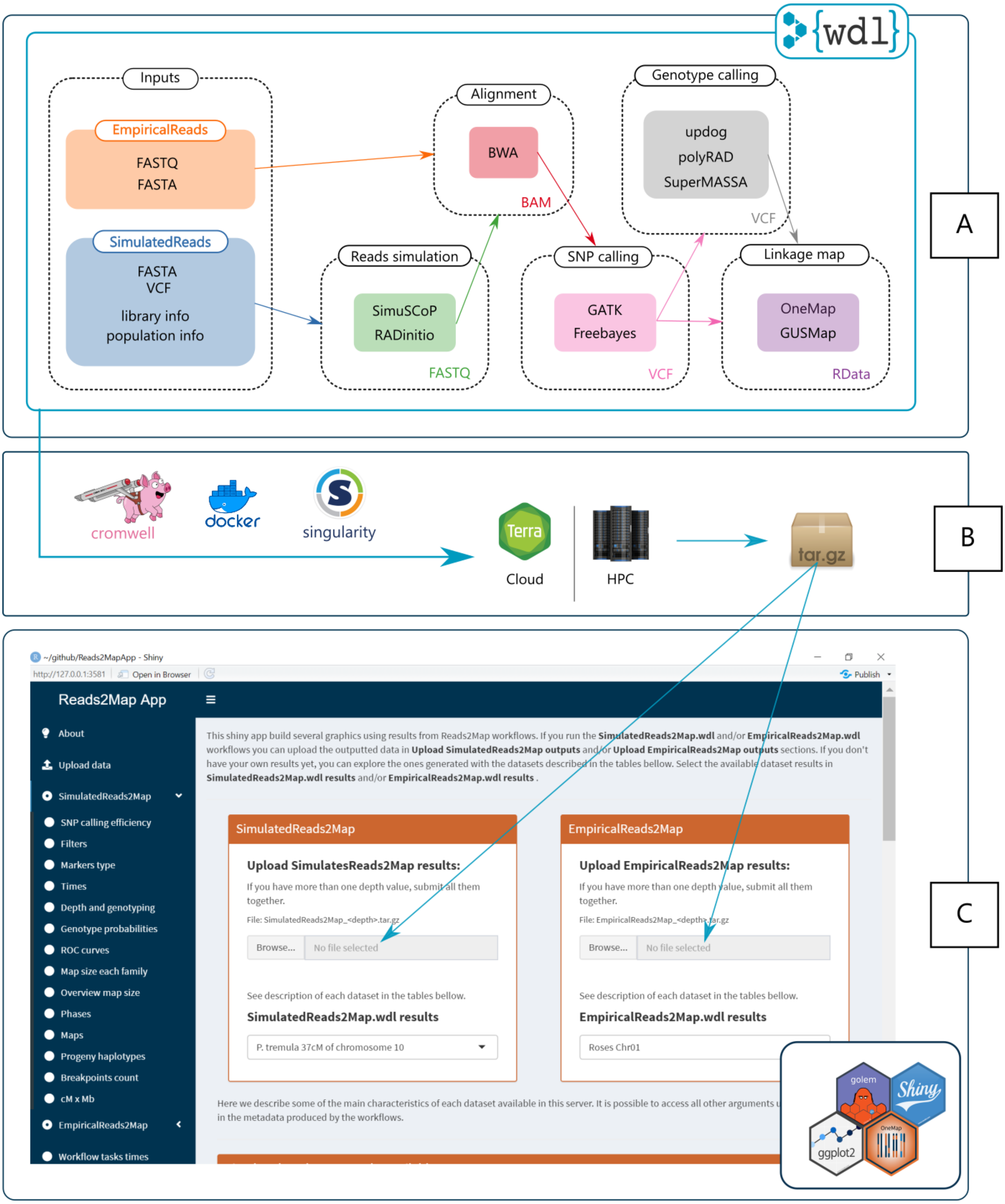

Figure 1. A: Tasks of the two main Reads2Map workflows: EmpiricalReads2Map.wdl and SimulatedReads2Map.wdl. B: Tools to run the workflows on the Cloud (`https://app.terra.bio/` platform) or in High-Performance Computing (HPC) environments. C: The Reads2Map shiny app has as input the outputs of the workflows. It builds several descriptive graphics to evaluate the best upstream software combination for linkage map construction.

mation, OneMap algorithms use code adapted from R/QTL package [34].

In short, the latent variable G_i, $i = 1, ..., n$, denotes the true underlying genotypes for the individual at a set of n ordered loci; O_i is the observed variable of the molecular phenotype (observed genotypes) for the locus i. The HMM can be represented as [35]:

$$P(O|G_i = g_i) = \sum_{g_1} ... \sum_{g_i-1} \sum_{g_i+1} ... \sum_{g_n} \pi(g_1) \prod_{j=1}^{n-1} t_j(g_j, g_{j+1}) \prod_{j=1}^{n} e(g_j, O_j) \quad (1)$$

The initial probability $\pi(g_1)$ is the probability of having a given genotype for the first locus (G_1), and its value depends on the cross-type. For example, for an outcrossing population, this value will be 0.25, assuming a uniform distribution of all four possible genotypes (AA, BA, AB, and BB). The same reasoning applies to backcross data, with probabilities of 0.5 since there are only two possible genotypes (AA and AB).

The transition probability $t_j(g_j, g_{j+1})$ is the probability of the genotype in a locus ($G_{j=i+1}$) changing to the next locus genotype (G_{j+1}). The initial value for this probability is based on the phase, and recombination fraction estimated by a two-point approach using maximum likelihood estimators [36], and is updated after iterations of the EM algorithm. The emission probability $e(g_j, O_j)$ is the probability of the observed variable given the genotype. This probability is defined by an associated genotyping error (see Supplementary file 1). The OneMap software previous to version 3.0 considered this error probability as a single value of 10^{-5} for every genotype. In version 3.0, this value is kept as default to maintain the code reproducibility. But it is noteworthy that this probability can be unreliable in several situations when the genotypes are more prone to errors, especially for new genotyping technology (e.g. GBS data). OneMap 3.0 allow users to provide individual values of error probabilities in the emission probability of the HMM for each genotype or marker, having a potential impact on the results. Using the *create_probs* function, users can provide three types of values: one global value, which was the previous default (*global_error*); an error probability for each inferred genotype (*genotypes_error*); or genotype probabilities for each possible genotype in individuals (*genotypes_probs*). We tested the consequences of building maps applying different genotype probabilities coming from five different genotype caller software, a global error rate of 0.05, and the old default value of 10^{-5}.

Here we used GATK [27], freebayes [26], polyRAD [28], SuperMASSA [29] and updog [10] to estimate the genotypes and genotypes probabilities. For GATK and freebayes caller, we used the Phred score genotype error (GQ FORMAT value) converted to probabilities. The software polyRAD, SuperMASSA and updog use the known population's structure (in our case F_1) as *a priori* information to increase the accuracy of the estimated genotypes.

OneMap uses the forward-backward algorithm [37] to compute the HMM combined with the expectation-maximization algorithm (EM). Since version 3.0, OneMap presents the possibility to parallelize the HMM using the approach described in [38]. It parallelizes the procedure into a maximum of four cores. We used this new OneMap feature to estimate the genetic distances. We also implemented new functions for linkage maps quality diagnostics such as interactive plots for recombination fraction matrices, progeny haplotypes representation, and counts of the recombination breakpoints in progeny. We compared OneMap 3.0 capacity of estimating accurate genetic distances with the GUSMap package estimations since it also uses an HMM to account for errors present in sequencing data.

Empirical data analysis

We ran EmpiricalReads2Map workflow using two empirical data sets that already have linkage maps built. They are GBS data sets from a bi-parental diploid F_1 full-sib mapping populations of aspen (*Populus tremula L.*) [39] (BioProject PRJNA395596), and rose (*Rosa spp.*) [40]. The aspen data set comes from an intraspecific cross of two *Populus tremula* genotypes. The GBS libraries were built using *HindIII* and *NalI* enzymes and sequenced as 150 base pair single-end reads on an Illumina HiSeq2500. Eight library replicates were built and sequenced for the parents and only one for each of the 116 F1 offspring. The data set includes six samples erroneously sequenced as part of the progeny and later identified as contaminants. An average read depth of approximately 6x for progeny and 58x for parental samples were observed from the sequencing process. The *Populus trichocarpa* genome version 3.0 [41] was used as a reference for the sequence's alignment.

The diploid roses data set comprises 138 individuals from the cross between a Texas A&M breeding line J06-20-14-3 (J14-3) and cultivar Papa Hemeray (PH). GBS libraries were built with *NgoMIV* enzyme and sequenced as a 113 base pair single-end read on a HiSeq2500. The parent J14-3 was repeated twice, and the PH sample three times. An average read depth of approximately 94x for progeny and 528x for parental samples was observed from the sequencing process. The *Rosa chinensis* v1.0 genome assembly [42] was used as a reference genome to align the sequences.

The sequencing reads of the two empirical data sets were filtered using the Stacks plugin process_radtags [2] to filter sequences by the presence of the restriction site and sequencing quality. The reads were discarded if the average quality score of 50% of its length was below the Phred score of 10 (or 90% probability of being correct). The software cutadapt [43] was used to remove adapters and filter by a minimum read length of 64 bp. The sequences were then evaluated in our EmpiricalReads2Map workflow.

Each time the EmpiricalReads2Map workflow is executed, it considers all the pipeline combinations generating 34 maps with combinations of SNP caller (GATK and freebayes), genotype caller (GATK/freebayes, polyRAD, updog, SuperMASSA), source of the reads counts (VCF and BAM files), and map builder packages (OneMap and GUSMap). The output provides maps built with genotype call software genotype probabilities, with 5% and 0.001% of global error rate in the HMM chain.

We executed the EmpiricalReads2Map workflows in the presence and absence of haplotype-based multiallelic markers and applied four different marker filtering methods. For the aspen data set, we also executed the workflows for every scenario in the presence of the contaminant samples. Therefore, the experiment has a total of 3 (data sets: rose, aspen and aspen with contaminants) × 2 (presence/absence of multiallelic markers) × 4 (filter methods - see details below) × 34 = 816 maps built for the first 8.426 Mb of chromosome 10 of *Populus trichocarpa* genome and the first 25 Mb (37%) of chromosome 1 *Rosa chinensis* reference genome. Table 1 shows an overview of the notations used to refer to each evaluated scenario. It is important to mention that this represents what users will find in building maps for the whole genome; a sample was required to reduce the computation burden.

GBS data simulation

The first step of the SimulatedReads2Map workflow is to perform simulations of a mapping population, GBS libraries, and sequences. The simulation is based on a given reference genome chromosome sequence. If a reference linkage map and a VCF file are provided, the workflow simulates the marker genetic distances and parental genotype frequencies based on them. A cubic spline interpolation with the Hyman method [44] is applied to simulate the centimorgan position for each marker's physical position based on this same relation on the reference linkage map provided.

We based our simulation analysis on the first 37% of the chromosome 10 sequence of *Populus trichocarpa* version 3.0, which comprehends a sequence with 8.426 Mb from a total chromosome size

Table 1. Notation used to refer to each evaluation scenario in empirical and simulated data sets.

Step	Notation	Description
Reads simulations	depth 10	Mean read depth used to simulate the data set
	depth 20	
SNP calling	`freebayes`	Software used to identify the variants
	`GATK`	
	BAM	Source files of allele depth information
	VCF	
Genotype calling	`polyRAD`	Software used to perform the estimation of genotype for a given allele depth information
	`SuperMASSA`	
	`updog`	
	`freebayes`/ `GATK`	Software used to genotype calling is the same that performed the SNP calling
Map building	`polyRAD`	Maps built with genotypes probabilities from `polyRAD`
	`SuperMASSA`	Maps built with genotypes probabilities from `SuperMASSA`
	`updog`	Maps built with genotypes probabilities from `updog`
	`freebayes`/ `GATK`	Maps built with genotype probabilities from `freebayes` if `freebayes` was used for SNP calling or `GATK` if `GATK` was.
	`polyRAD` (5%)	Maps built with genotypes from `polyRAD` and global error of 0.05
	`SuperMASSA` (5%)	Maps built with genotypes from `SuperMASSA` and global error of 0.05
	`updog` (5%)	Maps built with genotypes from `updog` and global error of 0.05
	`freebayes`/ `GATK` (5%)	Maps built with genotypes from `freebayes` or `GATK` and global error of 0.05
	`freebayes`/ `GATK` (0.001%)	Maps built with genotypes from `freebayes` or `GATK` and global error of 0.00001

of about 23 Mb. This sequence comprises 38 cM (21%) of the linkage group 10 reference linkage map built using the aspen empirical data [39]. Due to the computational resources needed to build such a high number of maps, we used only a subset of the data to finish the analysis in a reasonable time. Chromosome 10 was randomly chosen.

We simulated markers with different expected segregation patterns according to parental genotypes in each locus. Table 2 shows the notation for each possible marker type in an outcrossing diploid population. The SimulatedReads2Map workflow simulates parental haplotypes using the same proportion of marker types identified in the empirical VCF file. This approach overcomes the missing data present in the empirical data set. The final VCF file used as a reference to the simulations contains 810 markers (126 B3.7, 263 D1.10, 278 D2.15, and 143 non-informative markers with both parents homozygous), which results from the aspen empirical data `GATK` SNP calling, filtered by a maximum of 25% of missing data and MAF of 5%.

Table 2. Marker types according to parental genotype combinations and progeny segregation. The letters "a", "b", "c" and "d" represent different alleles and the letter "o" represents null alleles. Adapted from [45].

			Parents	Progeny	
Marker type			Cross	Observed genotypes	Expected segregation
A		1	ab x cd	ac,ad,bc,bd	1:1:1:1
		2	ab x ac	a,ac,ba,bc	1:1:1:1
		3	ab x co	ac,a,bc,b	1:1:1:1
		4	ao x bo	ab,a,b,o	1:1:1:1
B	B_1	5	ab x ao	ab,2a,b	1:2:1
	B_2	6	ao x ab	ab,2a,b	1:2:1
	B_3	7	ab x ab	a,2ab,b	1:2:1
C		8	ao x ao	3a,o	3:1
D	D_1	9	ab x cc	ac,bc	1:1
		10	ab x aa	a,ab	1:1
		11	ab x oo	a,b	1:1
		12	bo x aa	ab,a	1:1
		13	ao x oo	a,o	1:1
	D_2	14	cc x ab	ac,bc	1:1
		15	aa x ab	a,ab	1:1
		16	oo x ab	a,b	1:1
		17	aa x bo	ab,a	1:1
		18	oo x ao	a,o	1:1

`PedigreeSim` v2.1 software [46] is implemented in the workflow to simulate the meiosis events and generate an F_1 progeny based on the provided genetic map and simulated parental haplotypes. We did not consider the interference in meiotic events (Haldane [47] mapping function). `PedigreeSim` output files were converted to VCF files using `Reads2MapTools` (available at `https://github.com/Cristianetaniguti/Reads2MapTools`) R package function `pedsim2vcf`.

While converting the files, the `pedsim2vcf` function can also simulate segregation distortion by applying a selection strength. For that, a high number of individuals in the progeny have to be simulated with the `PedigreeSim` software and one or more loci to be under a given selection intensity. In our study, we targeted a final population size of 200 individuals. For that, we simulated 50 $\times$ 200 individuals and applied a selection intensity of 50% in the 30th marker, eliminating 50% of the genotypes containing one of the alleles. Then, 200 individuals of the resulting population are randomly selected to compose the mapping population. We used this feature to compare software performance in segregation distortion.

The VCF file output by pedsim2vcf is used as input in `RADinitio` software together with the reference genome sequence. `RADinitio` adds the VCF polymorphisms in the reference genome sequence and simulates the GBS sequences. It uses the inherited efficiency model [48] to simulate a PCR-amplified pool of molecules. The model includes the heterogeneity of the PCR amplification and the polymerase substitution errors. Next, `RADinitio` applies the user-defined ratio between DNA original molecules to be sequenced and PCR duplicates to create a distribution that will define the number of times the pool of loci is sampled, the number of duplicate molecules that are generated from a RAD locus template, and the distribution of PCR errors in the resulting reads. We defined the default parameter with a proportion of 4:1. Besides the PCR errors inserted during the pool sampling, the software also includes a commonly observed error pattern, where the 3' end of the read accumulates more errors than the 5' [49]. We tested different values of PCR cycles (5, 9, and 14) and mean depth (5, 10, and 20) to simulate the

FASTA files. We set the other simulation parameters to obtain 150 bases of read length, sequence size of 350, and restriction enzymes *HindIII* and *NalIII*. The mean read depth parameter for the parental samples was eight times higher than the progeny. The combination of `RADinitio` parameters that produced results closer to those observed in empirical data was selected to perform simulations with and without segregation distortion, five repetitions (five families), and two average sequencing depths (10 and 20) and 5 PCR cycles.

`RADinitio` does not output the sequence quality scores, so we converted the FASTA file format to FASTQ format, including a Phred score of 40 for every base simulated using `seqtk` [50] software. After obtaining the FASTQ files, the SimulatedReads2Map workflow followed the same tasks as the EmpiricalReads2Map, with alignment, SNP and genotype calling, and linkage map build. The SimulatedReads2Map workflow makes comparisons between real and estimated results within each step. The comparisons made during the workflow can be visualized in the shiny app `Reads2MapApp`.

Similarly to the EmpiricalReads2Map, the `SimulatedReads2Map` workflow generates maps for each combination of SNP and genotype call and linkage map building software. However, the total number of maps generated is multiplied by two because the workflows build maps with and without loci that were wrongly identified as polymorphic due to sequencing errors (false-positive markers). We also execute the `SimulatedReads2Map` workflow in the presence and absence of haplotype-based multiallelic markers, segregation distortion, and four methods for marker filtering. Therefore, the experiment has a total of 5 (repetitions) $\times$ 2 (average depths) $\times$ 2 (presence/absence of multiallelic markers) $\times$ 2 (with and without segregation distortion) $\times$ 4 (filters method - see details below) $\times$ 68 = 10,880 maps built for the first 8.426 Mb of chromosome 10 of *Populus trichocarpa* genome. Table 1 shows an overview of the notations used to refer to each evaluated scenario.

SNP calling

First, the FASTQ sequences are aligned with `BWA-MEM` [51] to their respective reference genomes. The workflow uses `samtools` [52] to merge the alignment of the same samples BAM files, keeping the libraries identification on the BAM header and filtering out reads with MAPQ < 10. After the alignment, BAM files for each sample are used as inputs for sub-workflows with `GATK` and `freebayes` approaches. One of the sub-workflow reproduces `GATK` joint genotyping via `HaplotypeCaller`, `GenomicsDBImport`, and `GenotypeGVCFs` tools and applies the suggested hard-filtering procedures [8]. The other sub-workflow runs `freebayes` parallelized by reference genome intervals. After obtaining the VCF files, indels marker positions are left-aligned and normalized with `BCFtools`, and multiallelic markers are separated into a new VCF file.

`GATK` and `freebayes` may introduce bias towards the reference allele when used to process low-coverage sequence data. `GATK` inserts the bias when reads are filtered in the local re-assembly step to avoid sequencing errors [53]. To overcome the bias during the genotype calling, the workflow applies two measures of allele depth, one from VCF and the other from BAM files. `BCFtools` is used to find the read depths information for each allele in BAM files and update the allele depths information in the AD (allele depth) field of the VCF file. Therefore, each SNP calling method results in three VCFs: i) biallelic markers with read counts outputted by the SNP callers, ii) biallelic markers with counts from BAM files, iii) multiallelic markers.

Genotype calling

For the empirical data sets, the alignment and SNP calling steps were performed with entire data sets, but for the next steps, we selected just a subset of markers (the first 8.426 Mb or 37%) of *Populus trichocarpa* chromosome 10 and the first 25 Mb (37%) of *Rosa chinensis* chromosome 1 reference genomes. The markers were filtered by minor allele frequency (MAF) of 5%, and maximum missing data allowed of 25%. The VCF files with biallelic markers from `freebayes` and `GATK`, and with read counts source from VCF and BAM files were the input for the genotype caller software `polyRAD`, `SuperMASSA`, and `updog`.

To use the `polyRAD` approach, the VCF files were imported using `VCF2RADdata` without applying any filters or considering phase information. The `polyRAD` model was run with `PipelineMapping2Parents` default arguments which assume an F_1 bi-parental population. The function `Export_MAPpoly` was used to export the genotype probabilities. The `vcfR` package [54] and custom R (function `polyRAD_genotype_vcf` in Reads2MapTools package) code were used to store outputted genotypes and their probabilities in a new VCF file. We also adapted `SuperMASSA` scripts to output the genotype probabilities information. The modified version is available in `Reads2MapTools` package. A wrapper function called `supermassa_genotype`, available in the package, can run the model in parallel and export the results to a new VCF file. The F_1 `SuperMASSA` model was run with parameter `naive_porterior_reporting_threshold` set to zero to not filter any genotype. The `updog` F_1 model was used in parallel using the function `multidog` through the `Reads2MapTools` wrapper function `updog_genotype` which outputs the results in a new VCF file. In the testing of scenarios in which we considered multiallelic markers, the VCF containing them are merged into the VCF files from `polyRAD`, `SuperMASSA`, and `updog`. The merged VCF is the input for linkage map building in `OneMap` version 3.0.

The software `GUSMap` performs the genotype calling and linkage map building with a single model. We used `VCFtoRA` function to convert the outputted VCF files from `GATK` and `freebayes` approaches into `GUSMap` format. A pedigree of the population and a list of filters (MAF = 0.05, MISS=0.25, BIN=0, DETPH=0 and PVALUE=0.05) was provided to the `readRA` function. The function `makeFS` was used to create the full-sib population information. Functions `infer_OPGP_FS` and `rf_est_FS` were used to estimate the phase and recombination fraction giving the genomic order of the markers. In some situations, function `rf_est_FS` outputs infinite values of the recombination fraction. In these situations, our pipeline removes the respective marker and runs the function again. This workaround code increased the time required to run `GUSMap`.

Linkage maps

Once imported to `OneMap`, markers were filtered again by maximum missing data of 25%. Because the VCF files include unexpected genotypes according to the loci segregation (e.g. in a cross "AA x AB", genotype "BB" cannot exist), `OneMap` makes these genotype calls missing. We also filtered markers with segregation distortion under a global significance level of 0.05 with Bonferroni correction and removed redundant markers. Markers were ordered according to the reference genome position. The genetic distances were estimated by the parallelized HMM multipoint [17, 38] approach using as emission probability a global error rate of 10^{-5} (default in `OneMap` version < 3.0, here referred to as "freebayes/GATK (0.001%)"), a global error rate of 0.05, and the genotypes probabilities estimated by each genotype caller.

In SimulatedReads2Map, the Haldane map function was used; in EmpiricalReads2Map, we used Kosambi's map function. To test the influence of the presence of the multiallelic markers in the ordering procedure, we used the built map for the chromosome 10 linkage group of aspen and ordered its markers using MDSMap [55] (wrapper function implemented in `OneMap` 3.0) and order_seq ordering algorithms with and without multiallelic markers.

Performance comparison

We conducted performance comparisons for each combination of SNP caller, genotype caller, and source of read counts, after which they were filtered by sequencing quality, MAF, segregation distortion, redundancy, and missing data. Outlier markers breaking the pattern of the recombination fraction matrix were removed only for the ordering test with and without haplotype-based multiallelic markers in the empirical data set. We evaluated the estimated progeny genotype concordance by comparing the agreement between real and estimated heterozygous, reference allele homozygous (homozygous-ref), and alternative allele homozygous (homozygous-alt) states. For that, we count the number of genotypes estimated as one type given that the true type was another, i.e., Est: homozygous | True: heterozygous. The methods are the combination of each SNP caller, genotype caller, and read count source. We expected that a good method would result in high probabilities for the same estimated and real genotypes (i.e. Est: homozygous | True: homozygous) and low probabilities when they are different (i.e. Est: homozygous | True: heterozygous). These were summarized using receiver operating characteristic (ROC) curves by plotting the *sensitivity* ($\frac{\textit{true positives}}{\textit{true positives+false negatives}}$) in the vertical axis versus 1 − *specificity* ($\frac{\textit{false positives}}{\textit{false positives+true negatives}}$) on the horizontal axis for all possible thresholds in a logistic regression [56].

To test the capabilities of software correctly estimating the parental genotypes, we used the same conditional frequency, but instead of measuring the similarities between individuals' genotypes, we tested the combination of both parental genotypes. To do that, we calculate the conditional frequency analysis between the marker types (e.g. Est=B3.7 | True=B3.7). Based on Mollinari et al. [57], we compared the centiMorgan distances of markers in the maps estimated by each method and the real map using the Euclidean distance (D):

$$D = [(m-1)^{-1}(\hat{\mathbf{d}} - \mathbf{d})'(\hat{\mathbf{d}} - \mathbf{d})]^{1/2}$$

where m is the number of markers evaluated, $\hat{\mathbf{d}}$ is the vector of estimated distances,$\mathbf{d}$ is the vector of real distances, and $'$ indicates vector transposition. A value of $D = 1$ means that the estimated map differs by an average of 1 cM from the built map regarding all genomic positions. We also evaluated the orders provided by the different ordering algorithms by computing the absolute value of Spearman's rank correlation between orders.

Read2Map Workflows App

The shiny app Reads2MapApp was built to display results from the workflow analysis. It includes graphics and statistics about SNP calling efficiency, the number of markers discarded by filtering steps, marker types, computer resources and time spent by each step of the workflow, allele depth by genotype, genotype probabilities, ROC curves, map size, map phases, recombination fraction matrix, progeny haplotypes, breakpoints count, and the correlation between linkage map and reference genome markers positions. Reads2MapApp is a modularized R package using the golem framework [58] that can be rendered and displayed locally or on a server. It can be installed from its GitHub repository and run with a single command (run_app). Once uploaded the Reads2Map output file in the upload section of the app, all graphics will be automatically generated.

Results and Discussion

RADinitio reads simulations

Allelic bias has been observed frequently in GBS data [10, 9]. The primary source of bias in GBS data is related to the PCR amplification step during library preparation [8, 9]. Duplicates can be generated from the library preparation using the PCR or from erroneous detection of a single amplification cluster as if multiplied by the optical sensor of the sequencing instrument [59]. For Whole Genome Sequence (WGS) and exome sequencing data, it is recommended that duplicated sequences are filtered out because of their redundant information and the bias that they can bring to the statistical analysis. In this context, we expect that most of the sequences have partial overlap. Therefore, it is possible to identify the duplicates as the ones that completely overlap with each other and have a lower quality score of the sequence base. But, with GBS data, duplicated sequences are expected to be common because all sequences have the same starting point: the restriction enzyme cut site. Filtering duplicates, in this case, would reduce the read depth per loci to only one read per allele and increase the uncertainties of genotype estimation in the presence of sequencing errors [60]. Duplicates in GBS present advantages to sequencing depth. However, they also bring more allelic bias and erroneous nucleotide substitutions from PCR.

With the `Reads2Map` workflows, we simulated the read sequences by testing several values of `RADinitio` parameters to try to be as similar as possible to the empirical data and real scenarios. We found that with low mean depths (5) and any of the number of PCR cycles tested (5, 9, and 14), almost all markers identified by `GATK` are filtered out in the segregation distortion test, and maps cannot be built. Setting the mean depth to 10 and a high number of PCR cycles (9 and 14) also kept a few markers in the `GATK` analysis. Therefore, we performed all the simulated scenarios using 5 PCR cycles with mean depths of 10 and 20.

The mean percentage of duplicated reads in the aspen empirical data set was 76% (SE 0.55%), while in the simulated data set with mean depths of 10 and 20 were, respectively, 88% (SE 0.00%) and 92% (SE 0.00%), according to the `Picard MarkDuplicates` tool [61] results. It shows that `RADinitio` simulates more duplicates per cycle than expected by the set proportion of 4:1 in the input parameters. Even with a lower number of PCR cycles (5), the simulated data presents more PCR duplicates than the empirical PCR performed to generate the aspen data set, which had 14 cycles [39]. The excessive number of PCR duplicates in the simulations may be why `GATK` identified a few false positives markers with a mean number of 0.49 for depth 10 and 0.48 for depth 20 (Figure 2).

Another difference between the simulated and empirical data set is the number of markers identified by `freebayes` and `GATK`. If the only filters applied to the identified markers are maximum missing data of 25% and MAF of 5%, `freebayes` identified 4.30x and 5.45x more markers than `GATK` in the rose and aspen data sets, respectively. This same proportion is not observed in the simulated data sets, in which `GATK` identifies a mean number of markers of 172.27 (SD 8.12) in depth 10 and 175.80 (SD 6.50) in depth 20, and `freebayes` identify a mean number of 160.39 (SD 2.10) in depth 10 and 157.33 (SD 2.47) in depth 20 (Figure 2). This shows that the simulations are biased towards `GATK` because its markers were used as references for the simulations.

In the simulated data, markers were close to the restriction enzyme cut sites identified in *P. tremula* empirical data. However, the simulations consider that the efficiency of the enzyme can vary across libraries which may explain the high number of false negatives (about 77% of the simulated data). Measuring the common markers across the simulated families, we observed a higher overlap of marker positions when estimated by `freebayes` than `GATK` (Figure 2).

Once the markers are identified, the genotypes can be estimated according to the read count at each locus. Ideally, in a diploid individual, the homozygous would receive the same allele from both

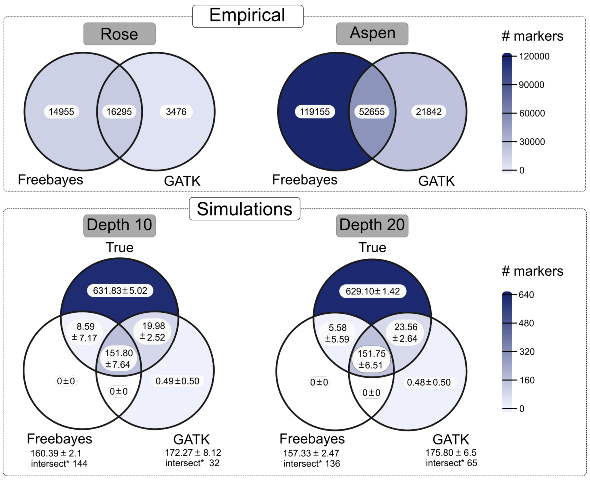

Figure 2. Venn diagrams show the number of markers identified by `freebayes`, `GATK`, and simulated (true). The intersection between the data sets represents markers with the same position in the reference genome *Populus trichocarpa* version 3.0. The Empirical data sets include markers spread across the entire reference genome. The simulations only include markers in the first 8.426 Mb of chromosome 10 (2.1% of the genome). The mean and standard deviation of number markers are shown for the simulated data set once the simulation and SNP calling are repeated 60 times. Markers were filtered by 25% maximum missing data and MAF 5% in empirical and simulated data. * Number of markers common to all 60 repetitions.

parents. The heterozygous would have half of the reads containing one allele and half a different one. However, we can observe the deviation of this ideal scenario in GBS empirical data (Supplementary Figures 5-6).

The `RADinitio` simulation results in alleles read counts distribution (Supplementary Figures 7-10) were similar to the observed in the progeny of the empirical data in terms of dispersion and allelic bias [9]. However, it could not simulate the low-depth counts for parents nor the outlier allele depth presented in the empirical data set. Thus, our simulations were not able to cover these two characteristics that can be found in empirical data sets.

In general, the evaluations of `RADinitio` simulations profile shows that we can expect fewer markers and genotyping errors in the simulated compared to the empirical data. A smaller number of markers should not reduce the built linkage map quality because the analysis was made in F_1 populations, which have large disequilibrium blocks. However, the smaller number of genotyping errors overestimates the SNP and genotype calling software efficiency. This overestimation is commonly observed in simulation results once the data cannot capture all biases and errors in the empirical data. If the software has low efficiency in simulated data, it will probably underperform with empirical data. Thus, the simulations can be used to understand specific software limitations but not ultimately define the best performance [62].

With simulated data results, it is possible to identify the source of the errors causing the low efficiency and elaborate methods to overcome them because simulated data provide a clear comparison between simulated (true) and estimated data. Therefore, the simulations were useful to optimize filters applied to identified markers and genotypes to obtain good quality linkage maps with simulated maps and improved maps with empirical data. We also used the simulations to measure the effects of segregation distortion in the linkage maps and to validate all code developed for the analysis.

Genotype calling efficiency

With the simulations, we could measure the number of wrongly estimated genotypes and the reliability of genotype probability provided by each software (Supplementary figure 7-12). We observed three types of errors: when the genotype is estimated as homozygous, but it is actually heterozygous (Est: homozygous | True: heterozygous); when the estimated genotype is heterozygous and the true genotype is homozygous (Est: heterozygous |True: homozygous); when the estimated genotype is alternative homozygous, and the true genotype is the reference or vice-versa (Est: homozygous-alt/ref | True: homozygous-ref/alt). The latter is only observed in genotypes estimated by `polyRAD`, `SuperMASSA`, and `updog` using `GATK` output VCF read counts (AD format) and had a maximum frequency of 0.74% of the genotypes in `SuperMASSA` estimations in simulations with mean depth 20. We observed that in these situations, the genotype is considered missing in the `GATK` output VCF GT format field, but it always reports the total read depth in the reference allele field of the AD format field (e.g. Estimated = GT:AD ./.;22,0 | True = GT:AD 1/1;0,22). This same issue can also cause errors of type Est: homozygous | True: heterozygous (Figure 3 and progeny genotypes in Figure 4) in `polyRAD`, `updog` and `SuperMASSA` genotypes generating an allele dropout scenario.

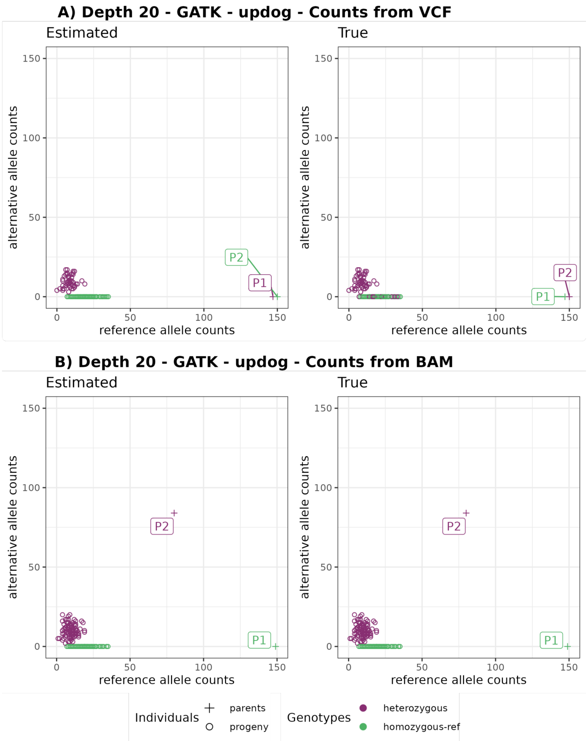

Figure 3. Example of error (Est: homozygous | True: heterozygous and Est: heterozygous | True: homozygous) in parental genotypes leading to a wrong marker type (Est: D1.10 | True: D2.15). Estimated reference (x-axis) and alternative (y-axis) allele count. Graphics on the left have colors according to estimated genotypes, and on the right to the true genotypes. A) show counts from `GATK` VCF file and B) from BAM file. In the VCF file outputted by `GATK` the P1 genotype is missing (GT ./.) because the reads did not pass the quality filters, but it reports the counts in the reference AD field (149,0). The `updog` software use progeny segregation (1:1) to estimate the parents, but it makes a mistake identifying which one is heterozygous. Using counts from BAM file (B) fix this issue despite losing the `GATK` quality filters that can be important in other situations.

Using the allele counts from the BAM alignment file, as suggested by [53], removes these types of errors in `polyRAD`, `SuperMASSA`, and `updog` genotype estimations with `GATK` markers. In contrast, by using the BAM counts, we lose the advantage of the robust filtering applied by `GATK` pipeline to maintain only the good quality read counts in its VCF allele depth field. To keep the `GATK` allele depth accurate but still overcome the common error observed when the genotype is missing, we replaced the VCF allele count (AD and DP fields) with zero when the genotype information is missing before using it for `polyRAD`, `SuperMASSA` and `updog` genotyping. In

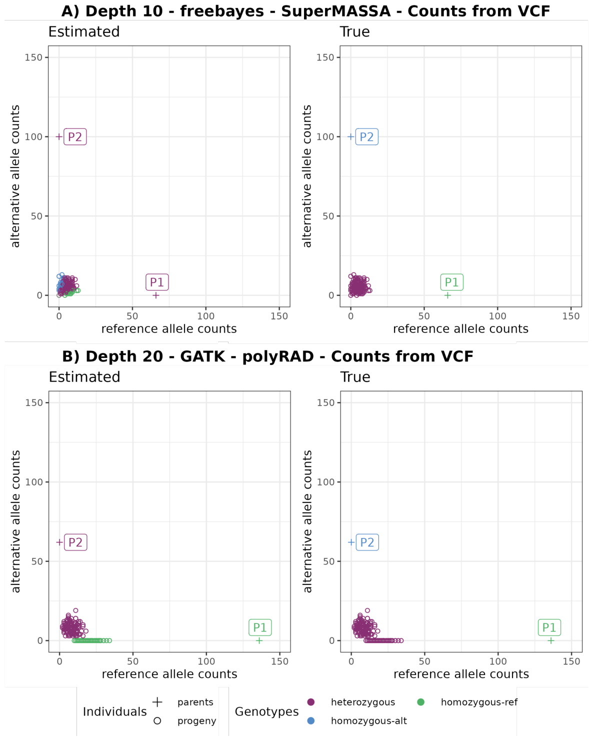

Figure 4. Example of error (Est: homozygous | True: heterozygous) in progeny genotypes leading to wrong marker types in A) Est: B3.7 | True: non-informative and in B) Est: D1.10 | True: non-informative. Graphics on the left have colors according to estimated genotypes, and on the right to the true genotypes.

empirical data, allele dropout can happen for other reasons, such as polymorphisms in the cut site or non-amplification of one of the alleles in the PCR step [9]. This requires another strategy to avoid wrong estimations.

For genotypes called by `polyRAD` and `updog`, the error (Est: homozygous | True: heterozygous) is more frequent than the error (Est: heterozygous | True: homozygous) in simulations with a mean depth of 10. The opposite is observed in some scenarios of the simulations with a mean depth of 20. This difference between simulations with mean depths 10 and 20 shows that `updog` and `polyRAD` are more susceptible to wrongly estimating homozygous genotypes in the presence of sequencing errors found more frequently at higher depths. All incorrectly called genotypes presented high differences in allele counts (e.g., 1 alternative allele: 23 reference alleles).

The scenarios with a higher number of correct genotypes were those called by `freebayes` and `GATK`, or by `updog` and `polyRAD` using markers from `freebayes` SNP calling, counts from VCF, and simulation mean of 20. The segregation distortion does not affect the frequency of correct genotypes in most scenarios (Supplementary figures 7-10), despite affecting the reliability of the genotype probabilities provided by `polyRAD` (Supplementary figures 11-12).

Marker types

Combining information from both parental genotypes defines the expected Mendelian segregation for each locus. The informative combinations for outcrossing species with biallelic codominant markers must have at least one heterozygous genotype in one of the parents, including the marker types B3.7, D1.10, and D2.15 (Supplementary figures 13-16). The haplotype-based multiallelic codominant markers can also present types A.1, A.2, D1.9, and D2.14. `OneMap` 3.0 does not consider the parental genotype probabilities in its HMM multi-point approach. Thus, it is important to plan the sequencing experiment with high-quality parental genotypes because, if there are errors, they will not be corrected in downstream processing, and it will cause distortions in the resulting distances and haplotypes. To avoid map size inflation, erroneous parental genotypes must be removed before the linkage map analysis.

Filtering the data set by segregation distortion is an efficient way of removing markers with wrong parental genotypes. The software `updog`, `polyRAD`, and `SuperMASSA` models consider the segregation pattern of the population to infer the genotypes, and, in some cases, they change the parental genotypes to fit in the observed population segregation pattern. If the progeny genotypes have low quality, it can lead to an erroneous estimation of the parental genotypes. We observed some cases in which non-informative markers are estimated as informative because of genotyping errors in progeny genotypes (Figure 4). In other cases, when alleles dropout in the heterozygous parent of a marker segregating 1:1, the models identify that one of the parents should be heterozygous, but the predictive models make mistakes in identifying which of them should be heterozygous (Figure 3).

We tested three other filters to overcome this in `updog`, `polyRAD`, and `SuperMASSA`. One of them was filtering the genotypes by the genotype probability. If the progeny genotype has a genotype probability lower than 0.8, the genotype is considered missing data. The marker is discarded if the frequency of missing data across all progeny is higher than 25%. The other filter tested was removing non-informative markers from the VCF file coming from `GATK` and `freebayes` before using it as input for `updog`, `polyRAD` and `SuperMASSA`. We considered non-informative markers homozygous in both parents or if at least one of the parental genotypes was missing. The third filter was to replace the allele depth (AD) field in the VCF file format by missing data when the genotype is missing. This avoids that `updog`, `polyRAD`, and `SuperMASSA` use the allele depth when `GATK` filtered out the genotype due to bad quality.

Removing the non-informative markers before the genotype calling by `updog`, `polyRAD`, and `SuperMASSA` reduced the number of wrongly identified marker types by that software, mainly in the simulated scenarios with a mean depth of 20 (Figure 5 and Supplementary figure 17).

We expect all multiallelic markers identified by `freebayes` to come from combinations of biallelic marker types (Figure 6 and Supplementary figure 18). The simulations showed the amount of B3.7, D1.10, D2.15, and non-informative markers converted to A.1, A.2, D1.9, and D2.14 markers. The D1.9 and D2.14 were converted from D1.10 and D2.15 SNP combinations, respectively. Also, the haplotyping approach could combine a few non-informative into A.1, D1.9, and D2.14 markers.

Relation between map size and correct haplotypes

Before using the map size as a metric for map quality, we checked if a map with the expected size always means good quality. A map can have the expected size but poor quality if the number of overestimated and underestimated recombination breakpoints in the progeny haplotypes is the same; in other words, if they cancel out. To test if this happens in our simulated data set, we compared the Euclidean relation of estimated and true genetic distances with the total number of wrong (overestimated + underestimated) recombination breakpoints in the progeny haplotypes (Figure 7 and Supplementary figures 19 and 20). For identifying a break as overestimated or underestimated, we do not consider the expected break position but the total breaks expected for the evaluated haplotype. For example, if one haplotype for a specific progeny was simulated with one break and estimated with zero, then we count it as one underestimated break.

The comparison shows that overestimated breakpoints are generally more frequent than underestimated ones. We observe that when a map is inflated, it also has many wrong recombination breakpoints. However, in some cases, the map has the expected

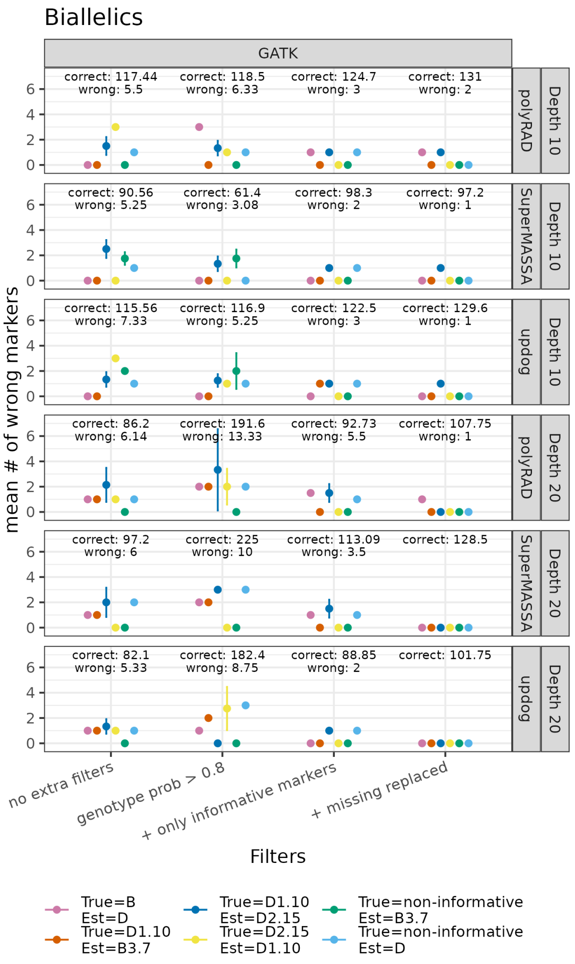

Figure 5. Mean number of wrongly identified biallelic markers in the simulated data set (y-axis) while applying filters by minimum genotypes probability of 0.8, by informativity and replacing AD and GQ VCF field by missing data when GT is missing (x-axis). The numbers on the top of each graphic show the mean total number of correct and wrong markers across the five repetitions. The markers presented here were obtained with GATK as SNP and updog, polyRAD, and SuperMASSA genotype calling, with mean depths 10 and 20, with segregation distortion, with allele depth count from VCF. The notation of marker types follows table 2 notation.

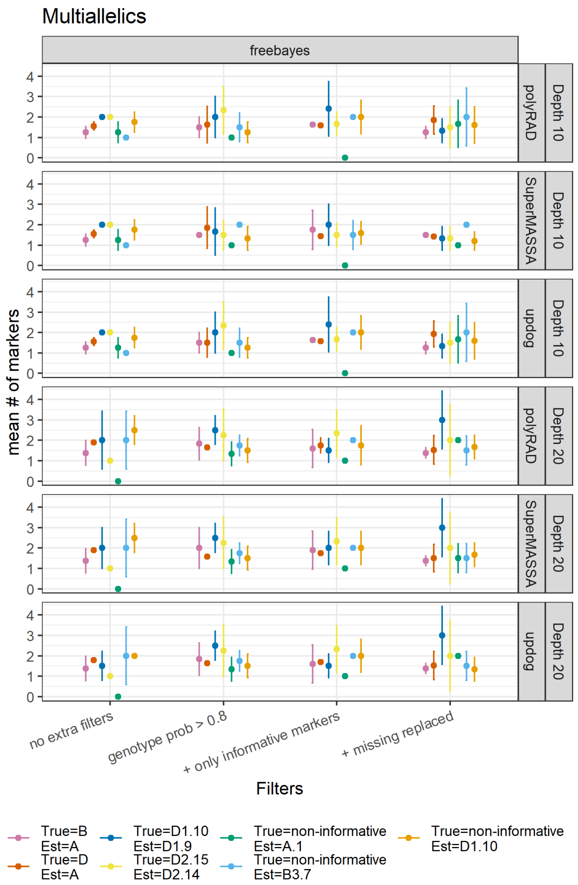

Figure 6. Mean number of multiallelic markers converted from biallelics (y-axis) and how many of them are kept after applying filters by minimum genotypes probability of 0.8, by informativity and replacing AD and GQ VCF field by missing data when GT is missing (y-axis). The markers presented here were obtained using simulated data, freebayes as SNP and updog, polyRAD and SuperMASSA genotype calling, with mean depths 10 and 20, with and without segregation distortion, with allele depth count from VCF. The notation of marker types follows table 2 notation.

map size, but a high number of wrong haplotypes due to both overestimated and underestimated breaks. A high number of underestimated breaks can be observed in situations where the Euclidean distance is close to, or less than 1 ($log_{10}0$) and the number of wrong recombination events is between 10 and 100 ($log_{10}1$–$log_{10}2$). These situations are more frequent when a global error rate of 5% is used.

Effects of contaminant samples

In the empirical data results, we observed maps with expected size and excess recombination breakpoints in just a few individuals in the progeny. This variation can be related to contaminant samples. The study of Zhigunov et al. [39] identified six contaminants in the aspen data set. When we ran the workflows, including the contaminant samples, the maps built with freebayes markers and updog, SuperMASSA, and polyRAD were smaller in size than without the contaminant. This would (wrongly) suggest better quality if map size is the only metric used (Figure 8A and Supplementary figure 21A). Nevertheless, the maps presented higher differences in the number of recombination breakpoints among individuals when using the genotype probabilities relative to each genotype call software (Figure 8B and Supplementary figure 21B). Some contaminant samples presented more recombination events than the rest of the progeny. Using 5% of global error reduces this difference and can mask the presence of contamination (Figures 9).

Effects of filters

Another important characteristic to consider in a good-quality map is the number of markers. The same data set will vary according to the SNP and genotype call software and filters used. We filtered all data sets by maximum missing data of 25%, segregation distortion, and redundancy. We tested the effects of three extra filters based on common errors observed in the simulated data set genotyping evaluations (Figures 3 and 4): minimum genotype probability of 0.8; removal of non-informative markers; replacing AD and GQ with missing data when GT is considered missing in the VCF file (Figure 10 and 11). These filters are applied before the segregation test filter, which reduces the number of tests and increases the permissibility of the threshold corrected by multiple tests (Bonferroni correction). Thus, the built map can have more markers in some scenarios even if more filters are applied.

Maps built with genotypes from GATK and a global error of 5% were smaller when filtering by a minimum genotype probability of 0.8 in higher depths of empirical and simulated data (Supplementary figures 22 and 23). The most significant effect of the filters can be observed in maps built with updog, SuperMASSA and polyRAD genotypes and genotypes probability (Figures 11 and 10). In both empirical and simulated data sets, higher-depth scenarios generate

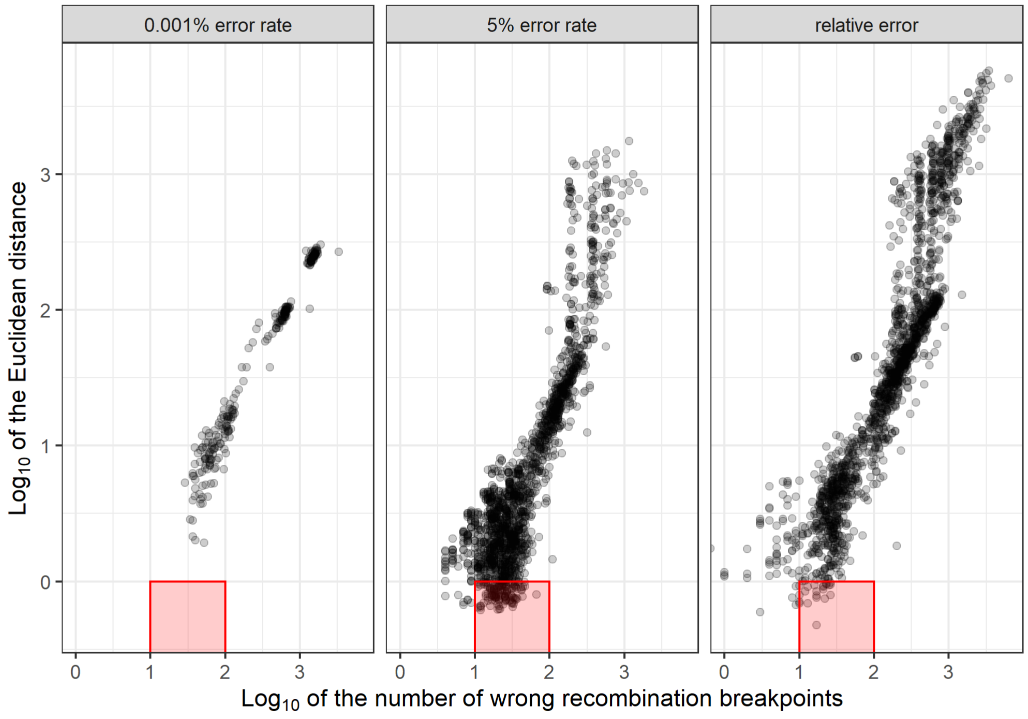

Figure 7. Relation between Euclidean distance (y-axis) and the number of recombination breakpoints (x-axis) in maps built with global error rates (0.001% and 5%), and with probabilities outputted by the genotype call software (relative error). Each dot represents a map built with simulated data based on the first 37% of aspen chromosome 10. The red squares highlight maps that do not present inflated size (1 or less Euclidean distance) but have from 10 to 100 wrong recombination breakpoints.

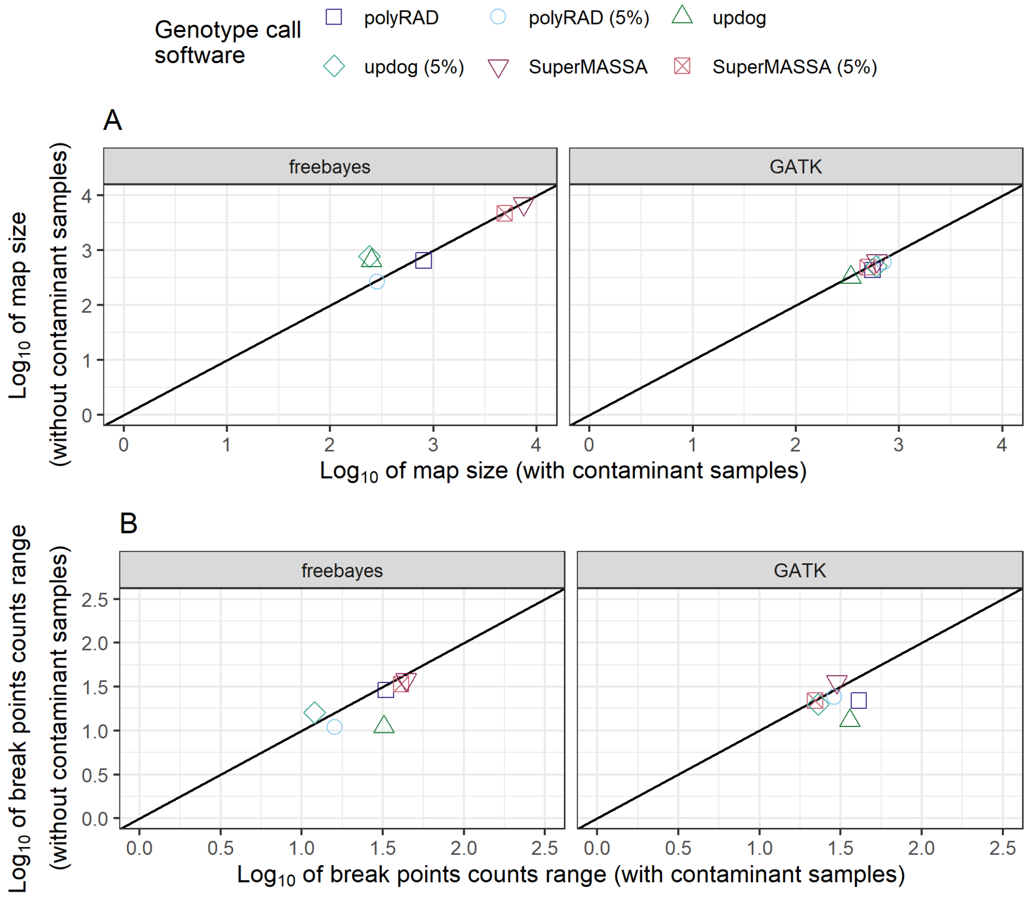

Figure 8. Effect of contaminant samples in the map size (A) and the number of estimated recombination breakpoints range among aspen empirical data set progeny individuals (B). The data sets presented in this figure contain multiallelic markers, the allele counts from the VCF file, and were filtered by genotype probability higher than 0.8 and contain only informative markers.

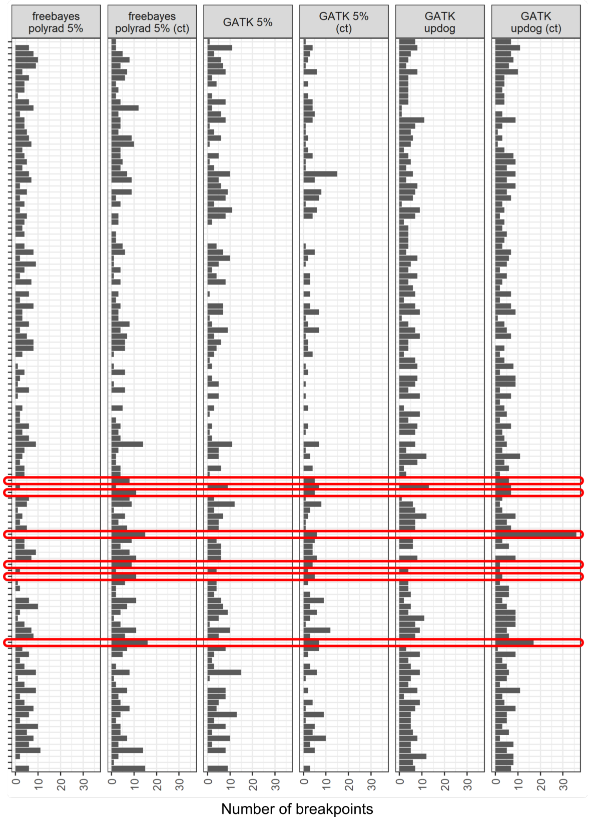

Figure 9. The total number of recombination breakpoints (x-axis) estimated for each progeny individual (y-axis) of the aspen full-sib population with and without contaminant samples (cont) with selected pipelines. The red ellipses indicate the contaminant samples.

linkage maps with sizes closer to the expected after the extra filters are applied.

Effects of segregation distortion

The segregation distortion in the data does not affect the number of wrong estimated genotypes by the genotype call software (Supplementary figures 7-10), but it can affect the reliability of `updog`, `SuperMASSA`, and `polyRAD` in outputting genotype probabilities in some scenarios (Supplementary figure 11 and 12). Consequently, the map size can be inflated using genotype probabilities from these software (Figure 12 and Supplementary figure 24).

Comparison with `GUSMap`

We compared all maps built with `OneMap` combined with upstream approaches with maps built with the GUSMap [14] software (Figures 13, 14 and Supplementary figures 25 and 26). We could not apply the extra filters to GUSMap genotypes as they are estimated internally in the software. In both simulated and empirical data, the maps generated by GUSMap presented greater map sizes.

Selected pipelines

The differences between simulated and empirical data discussed below also result in differences in the performances of software in these two data set types (Figure 15 and Supplementary figures 27-29). We focused on selecting the best pipelines only for the empirical data. For those, we consider as promising approaches the ones that resulted in linkage maps with a high number of markers, with no or few outlier markers distorting the total map length (Figures 15 and Supplementary figure 27), and with the number of recombination breakpoints identified in each progeny individual closer to what is expected for a 38 cM group according to meiotic properties (Figure 9 and Supplementary figure 30).

The rose data set presents higher sequencing depth; thus, the quality of the genetic map is generally better than the aspen data set. Using the filters by genotype probability and non-informative markers, it was possible to remove the majority of the outliers from the maps built and still keep a high number of markers by using `GATK` markers, `GATK` and `polyRAD` genotypes, and a global error rate of 5%. Despite presenting a higher number of markers, the approach using `freebayes` markers and genotypes with a global error rate of 5% resulted in a map with double the size (Figure 16). The number of recombination breakpoints profiles in these three cases shows that the individual 649-12 is a possible contaminant in this data set (Supplementary figure 30). The contaminant samples tend to have a higher number of breaks, as we saw in the comparison of aspen with and without contaminant samples.

In the aspen data set, the best approach was to build the map with `GATK` markers, `GATK` genotypes and a global error of 5%, or with `updog` genotype probabilities (Figure 17). Similar maps were also built using markers from `freebayes`, genotypes from `polyRAD` and a global error rate of 5%. All the maps built for the aspen data set still presented some outlier markers. Removing these outlier markers requires careful evaluation of diagnostic graphics, such as the heatmaps of the recombination fraction matrix (Supplementary Figures 31 and 32), which is not possible with the workflow's straightforward approach. It makes `Reads2Map` workflows a tool for selecting the SNP and genotyping calling and the genotype probability to build the map, but further revisions to remove the outliers are required to obtain a good quality genetic map.

Haplotype-based multiallelic markers

The previous evaluations show that multiallelic markers do not present a unique effect on the genetic distances (Figures 19 and 18 and Supplementary figures 33 and 34). Depending on the data set quality and combination of software used, it can decrease, increase, or even not affect the linkage map quality under these criteria. We target approaches that can reduce or not affect the genetic map size because the advantage of using multiallelic markers is not in the genetic map distance estimation but in the ordering step of the linkage map building. Algorithms that use two-point recombination fractions estimations to order only biallelic markers have difficulty missing linkage information between markers D1 and D2 (homozygous x heterozygous or vice-versa). These markers can only be related to each other in the presence of more informative markers, such as B3.7 (heterozygous x heterozygous) or the multiallelic. Yet, having few B7.3 markers compared to D1 and D2 can still be an issue for linkage map building. This characteristic was why the first methods for building genetic maps in this type of population resulted in separate maps for each parent [63]. The non-integrated genetic maps limit further QTL analysis of multiallelic traits [64].

The ordering step was not considered in the previous evaluations once the workflows used genomic order to build the maps. To test the effect of multiallelic markers in the ordering, we built a linkage map for the entire chromosome 10 of the aspen data set using markers called by `freebayes`, an error rate of 5%, and two of the `OneMap` order_seq and MDS algorithms to order the markers. The genetic distances were estimated by HMM multipoint approach. Figure 20 B shows the impact of including the multiallelic markers in the two-points-based MDS algorithm [55]. Multiallelic markers slightly increase the Pearson correlation and drastically reduce the Euclidean distance between the estimated ordering and the genomic order. The order_seq algorithm is a strategy developed to apply HMM in the ordering procedure. First, it estimates the order of the markers using a two-point approach (the default is

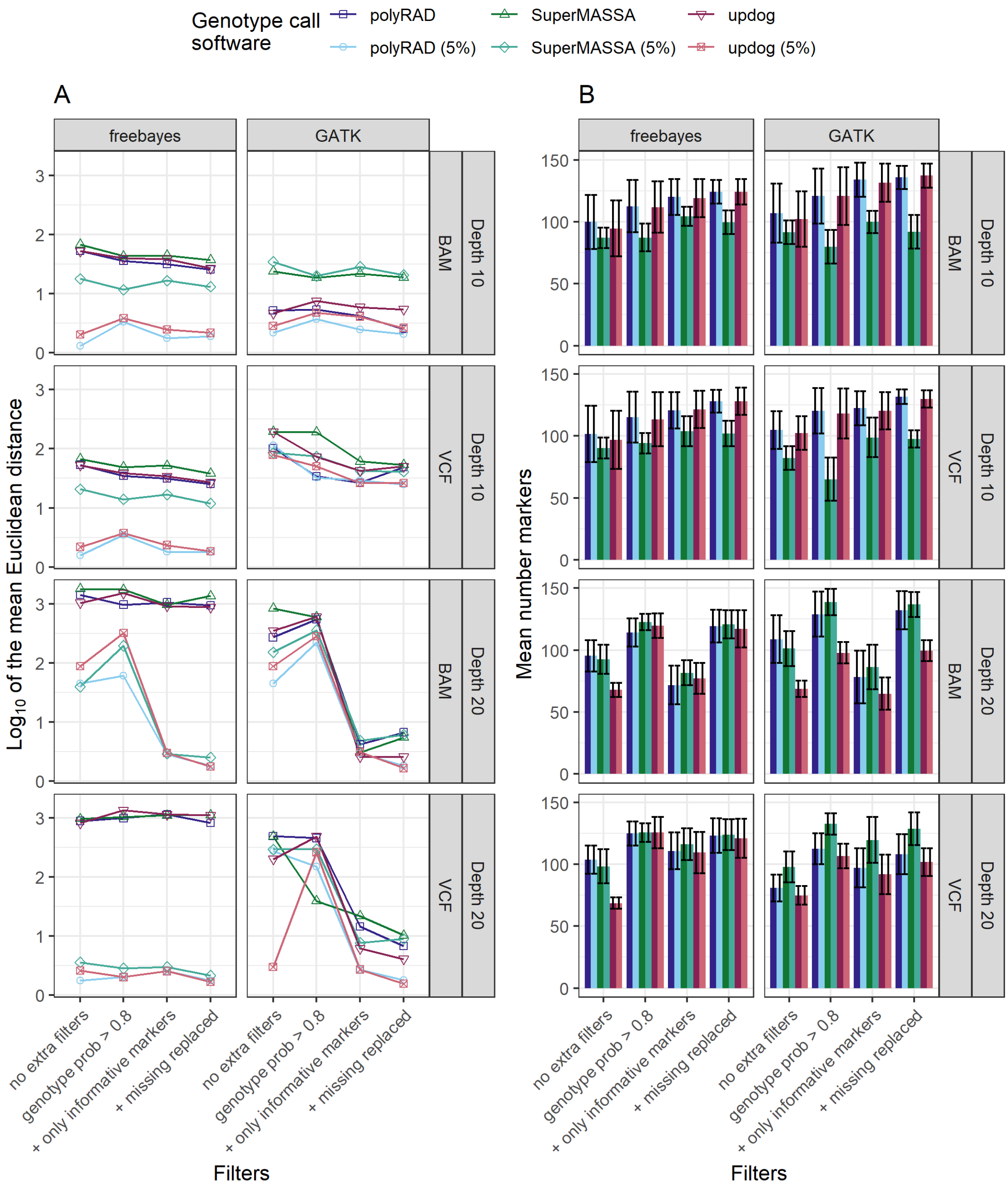

Figure 10. The relation between filters applied (x-axis) and the mean Euclidean genetic distances (A y-axis) and the number of markers (B y-axis) for genotype calling software. The data set shown in the figure contains multiallelic markers and segregation distortion.

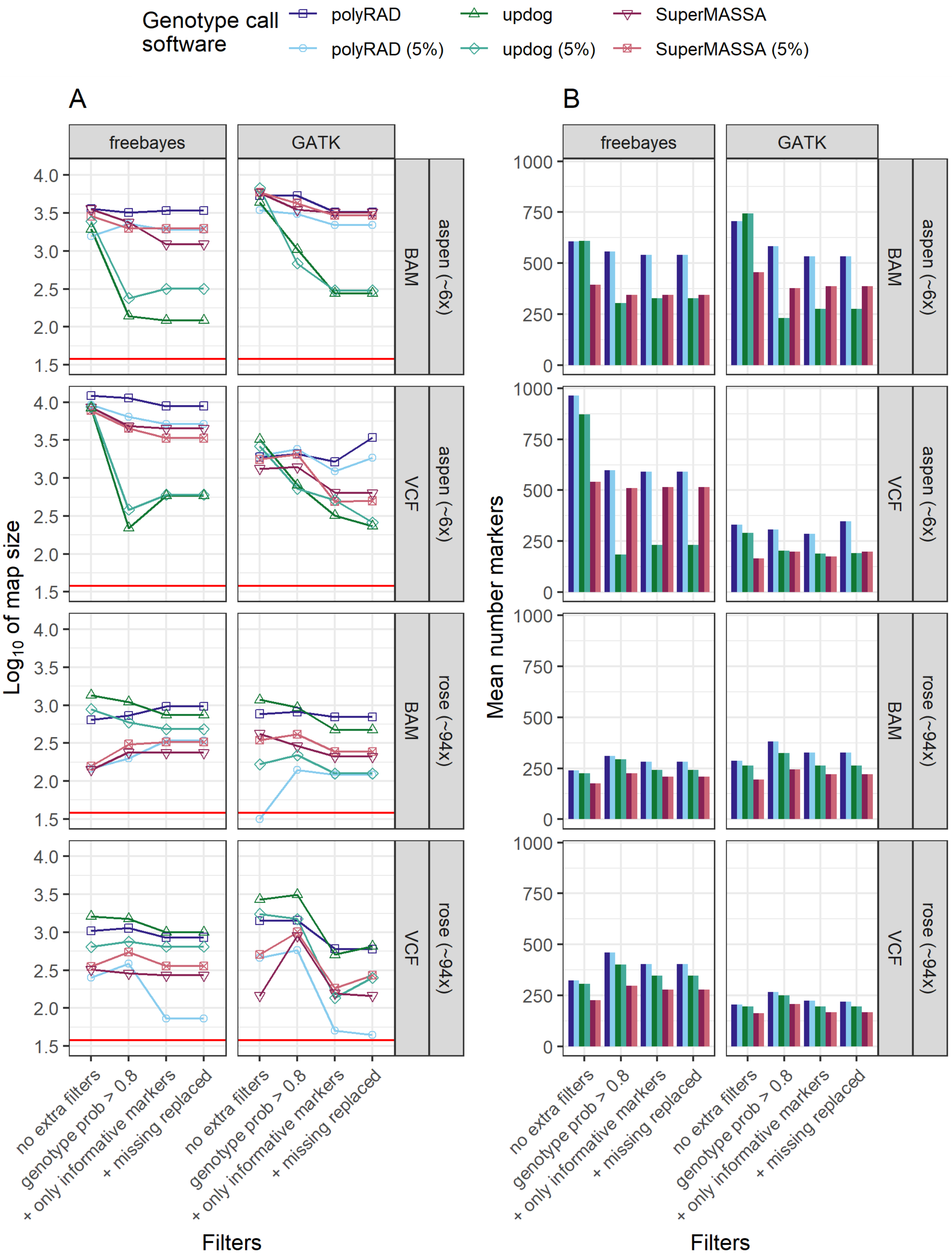

Figure 11. The relation between filters applied (x-axis), the map size (A y-axis), and the number of markers (B y-axis) for genotype calling software used in the empirical data sets. The data sets shown in the figure contain only biallelic markers. The horizontal red line indicates the expected map size (38 cM) for the subset of the genomes used.

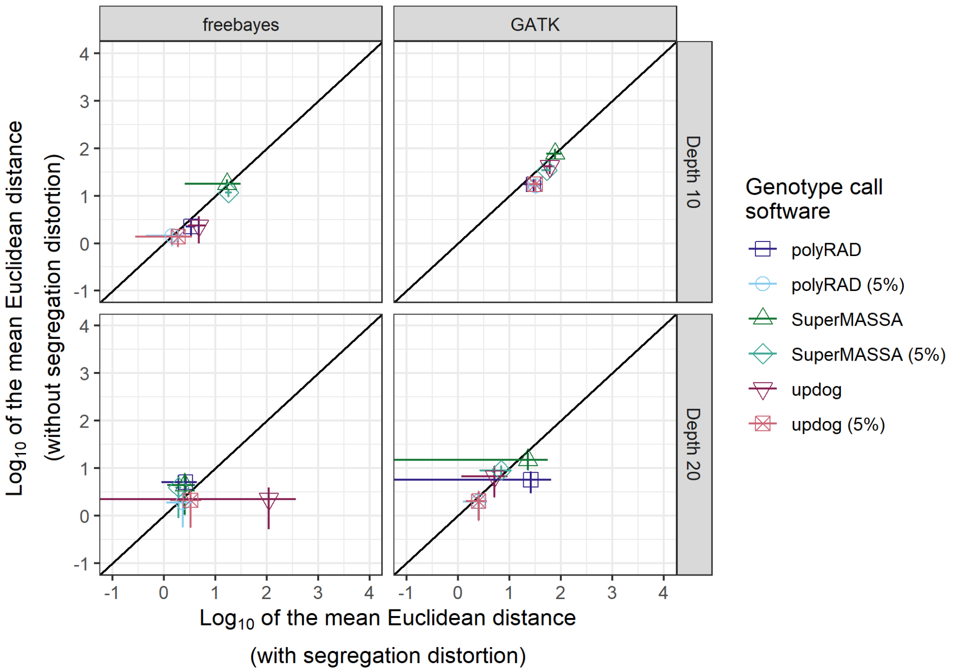

Figure 12. The effect of the simulated segregation distortion in the maps Euclidean distance by genotype calling software. The x-axis shows the mean of the Euclidean distance between estimated and simulated maps built for the data set with simulated segregation distortion, and the y-axis shows with data set simulated without the segregation distortion. The lines crossing the symbols indicate the standard deviation across the five repetitions. The data sets contain only biallelic markers and allele depth count from the VCF file. The markers were filtered by genotype probability higher than 0.8 and only informative markers.

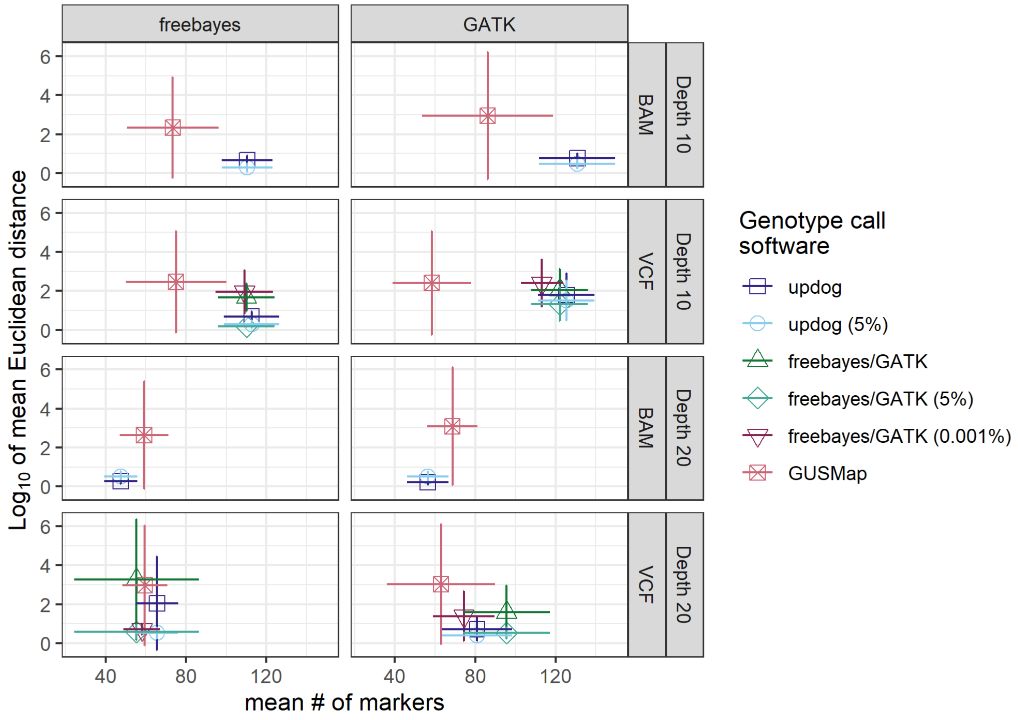

Figure 13. Comparison of Euclidean distance (y-axis) and the number of markers in maps built with `OneMap 3.0` and `GUSMap` for the simulated data. The lines crossing the symbols indicate the standard deviation across the five repetitions. The maps built with `OneMap` are represented by the name of the genotype calling software that provided the genotypes and their probabilities for `OneMap` multipoint approach of genetic distance estimation. The markers inputted in `OneMap` included multiallelic markers filtered by genotype probability higher than 0.8, included only informative markers, and the AD and GQ fields were replaced by missing data when GT is missing.

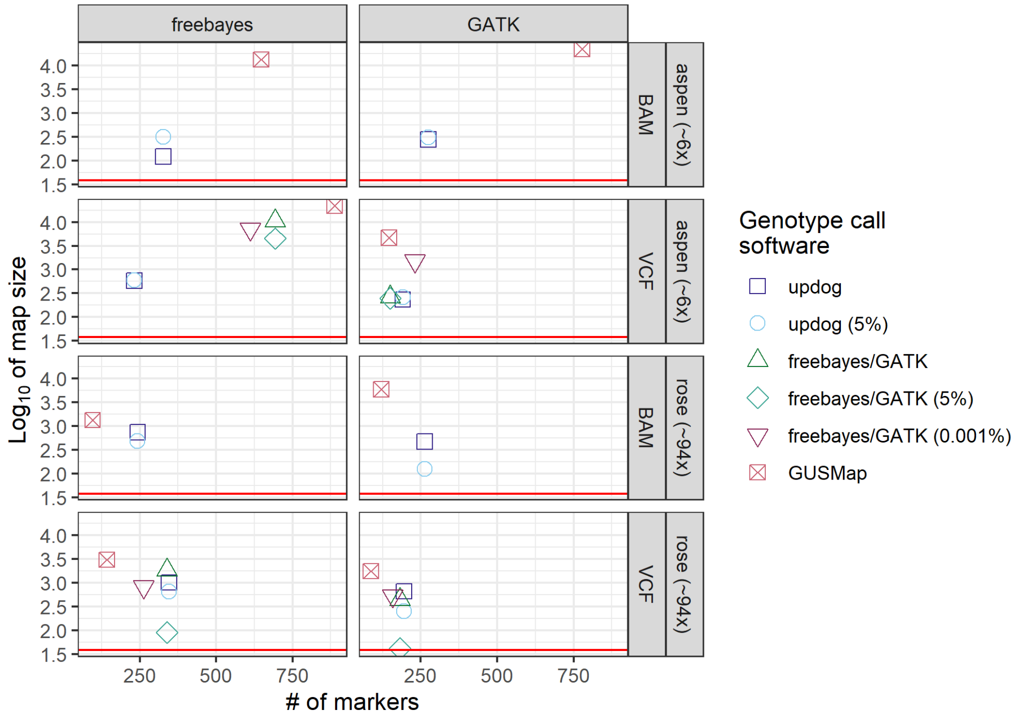

Figure 14. Comparison of map size (y-axis) and the number of markers in maps built with `OneMap` 3.0 and `GUSMap` for the empirical data. The maps built with `OneMap` are represented by the name of the genotype call software that provided the genotypes and their probabilities for `OneMap` multipoint approach of genetic distance estimation. The data sets shown here contain only biallelic. Markers inputted in `OneMap` had a genotype probability higher than 0.8, included only informative markers, and the AD and GQ fields were replaced by missing data when GT was missing. The horizontal red line indicates the expected map size (38 cM) for the subset of the genomes used.

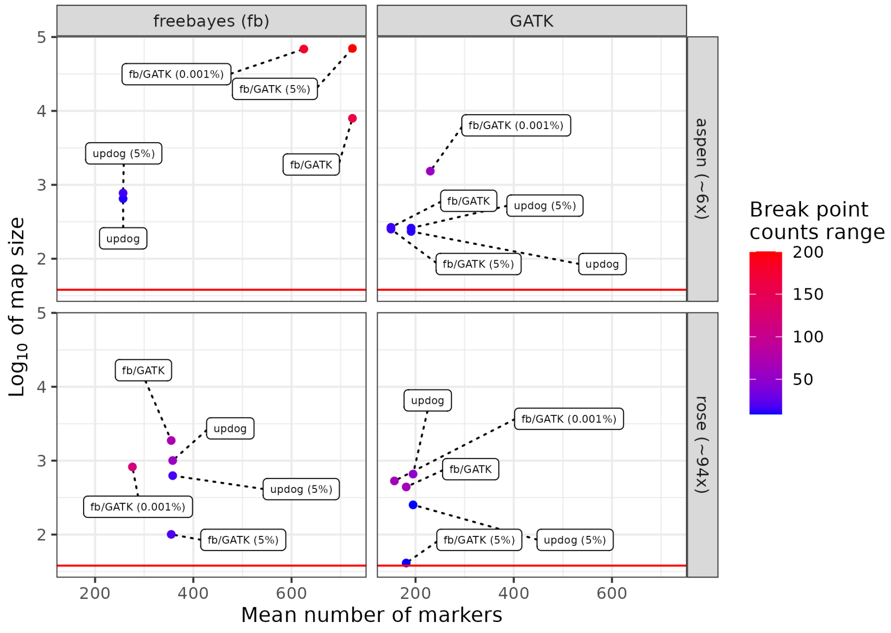

Figure 15. Comparison of map size (y-axis) and the number of markers in maps built with `OneMap` 3.0 using different upstream software for estimating genotypes and genotypes probabilities. Markers inputted in `OneMap` included multiallelic markers filtered by genotype probability higher than 0.8, included only informative markers, and the AD and GQ fields were replaced by missing data when GT is missing. The horizontal red line indicates the expected map size (38 cM) for the subset of the genomes used.

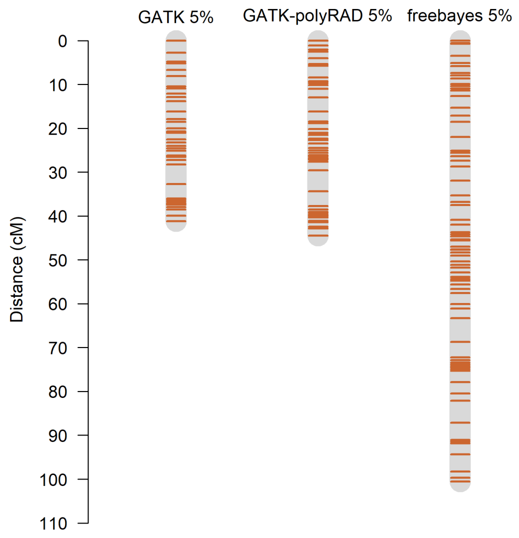

Figure 16. The figure shows the linkage maps built for 37% of rose chromosome 1 (38 cM) with selected pipelines.

the RECORD [65] algorithm). Based on the two-point ordering, a subset (default of five markers) of equally distributed markers is selected and ordered by exhaustive search (compare function). Next, the algorithm adds all the other markers sequentially, testing each possible position using the HMM multi-point approach in the already established sequence. The RECORD algorithm has steps where markers are randomized, which makes the result non-deterministic in the sense that each run can result in a (normally slightly) different order. This strategy used to be very accurate when dealing with a few informative markers (such as SSRs) but is more prone to errors if only biallelic markers are available. Results show that, with haplotype-based multiallelic markers, the strategy returns a high-quality order, reproducing almost entirely the genomic order and the correct pattern of the recombination fraction matrix (Figure 20 A).

Final considerations

The `Reads2Map` workflows have a robust structure to generate production-level results with simple inputs and optimized usage of computational resources. The structure allowed us to test the quality of genetic maps built with the following scenarios: i) using different SNP calling software (`GATK` and `freebayes`); ii) using different genotype calling software (`GATK`, `freebayes`, `updog`, `polyRAD`, `SuperMASSA`); iii) using different linkage map building software (`OneMap` 3.0 and `GUSMap`); iv) establishing different error probabilities (relative to genotype call software, 5%, and 0.001% global error); v) applying different marker filtering; vi) with or without multiallelic markers; vi) in empirical and simulated data; vii) with and without segregation distortion; viii) with and without contaminant samples; ix) with different library preparation; and x) with different sequencing depths. These scenarios are commonly found by researchers trying to produce high-quality linkage maps using sequencing technologies. The `Reads2Map` and `Reads2MapApp` are the first tools to guide best practices for building linkage maps with sequencing data pointing software, parameters and marker filters to be used in diverse scenarios.

We elaborated and limited the scenarios explored according to our experiences as developers of `OneMap`. `OneMap` first version was released in 2007, and since then it has been used to build linkage maps in a diversity of species. Its strategies and structure also served as a base for more complex software such as `MAPpoly` [15] for building linkage maps in polyploid species. With time, new methods for genetic marker identification using sequencing data emerged, changing the context where `OneMap` was used. We included updates in this version 3.0 to resolve issues with inflated genetic maps and marker ordering. Two major changes allow users to read and build genetic maps with the genotype probabilities and haplotype-based multiallelic markers information from the input files (`OneMap` format or VCF file). However, the success of genetic map building will be proportional to the quality of the information provided by upstream procedures such as library preparation, SNP and genotype calling, genotype probabilities estimation, and the combination of SNPs into haplotype-based markers. With `Reads2Map` and `Reads2MapApp`, we provide users tools to select the best approaches before using `OneMap` 3.0 to guarantee that it will result in the best quality genetic map possible with the data available.

For the rose data, the best pipelines filtered the markers using all extra filters (minimum 0.8 of genotype probability, removal of non-informative markers, and replacing AD and GQ field by missing if GT is missing in VCF file), and used the combinations: `GATK` as SNP and genotype calling with a global error of 5%; `GATK` as SNP calling and `polyRAD` as genotype calling with a global error rate of 5%; `freebayes` as SNP and genotype calling with multiallelic markers and a global error rate of 5%. The aspen had a lower sequencing depth. Thus, none of the methods could provide maps with the expected size. Even using the selected methods, further marker filtering was required to obtain a good-quality final map. For the aspen data set, we obtained the best pipelines by also filtering the markers with all extra filters and using the combinations: `GATK` as SNP and genotype calling with a global error rate of 5%; `GATK` as SNP calling and `updog` as genotype calling using `updog` genotype probabilities or `freebayes` as SNP and `polyRAD` as genotype calling using a global error rate of 5%.

Most of the selected pipelines for both empirical data sets used a global error of 5% to estimate the genetic distances because they gave map sizes closer to the expected. We also observed the same results when applying a 5% error rate in the simulated data. With those, we could relate the map size with the number of wrongly estimated haplotypes. The evaluation showed that inflated maps mostly reflect a high number of wrongly estimated haplotypes, but there were some cases where the map was estimated with the expected size but presented a high number of wrong haplotypes, mostly when a 5% global error rate was applied. Using a 5% error rate can also mask the presence of contaminant samples among the progenies. For these reasons, we intend to update `Reads2Map` with genotype calling software that adapt the genotype probabilities for this specific usage and result in map sizes closer to the expected.

The diversity in the pipelines suggested for both empirical data sets highlights that pipelines perform differently with data sets with different properties. We can see this diversity in the effects observed while testing filters, software, and conditions. This means that the pipelines presented here as the best cannot be considered the best for every data set. Thus, users should reproduce all tests presented here using the `Reads2Map` workflows with their empirical data set and select the best pipelines for their specific conditions. The workflows were built using WDL and containers to ensure high reproducibility. This guarantees that different results running different data sets is due to the data set's properties and not to bioinformatic pipeline changes. Also, as the upstream procedures for genotyping and identifying haplotype-based multiallelic markers are improved, updates can be easily made in the workflows.

Every `Reads2Map` workflow run returns a large amount of information. Every step of the workflow, from the reads' alignment to the completed linkage map, provides quality measurements for users to evaluate each scenario. The `Reads2MapApp` shiny app receives all this information compressed in a single workflow output file and converts it into comprehensive interactive graphics. Through the app interface, users can evaluate the performance of

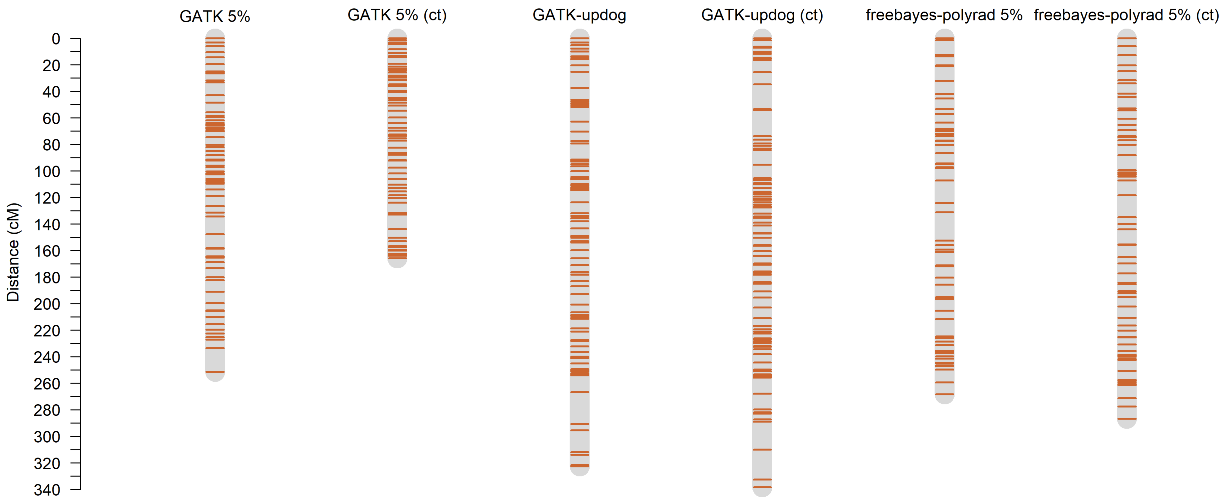

Figure 17. The figure shows the linkage maps built for 37% of aspen chromosome 10 (38 cM) with (ct) and without the presence of 6 contaminant samples and selected pipelines.

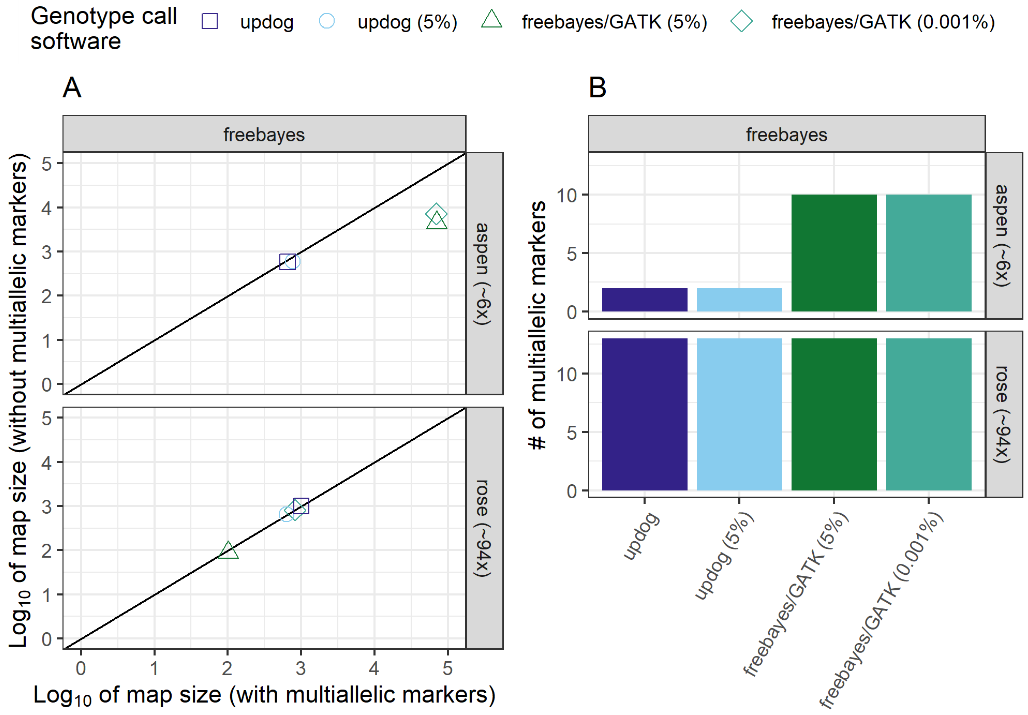

Figure 18. Comparison between empirical data sets with and without multiallelic markers. A: relationship of map size between data set with (x-axis) and without (y-axis) multiallelic markers. B: Number of multiallelic markers present in data sets represented in the x-axis of graphic A. The data sets shown in this figure have allele depth counts from the VCF file, were filtered by a minimum genotype probability of 0.8, included only informative markers and AD and GQ VCF fields were replaced by missing when GT was missing.

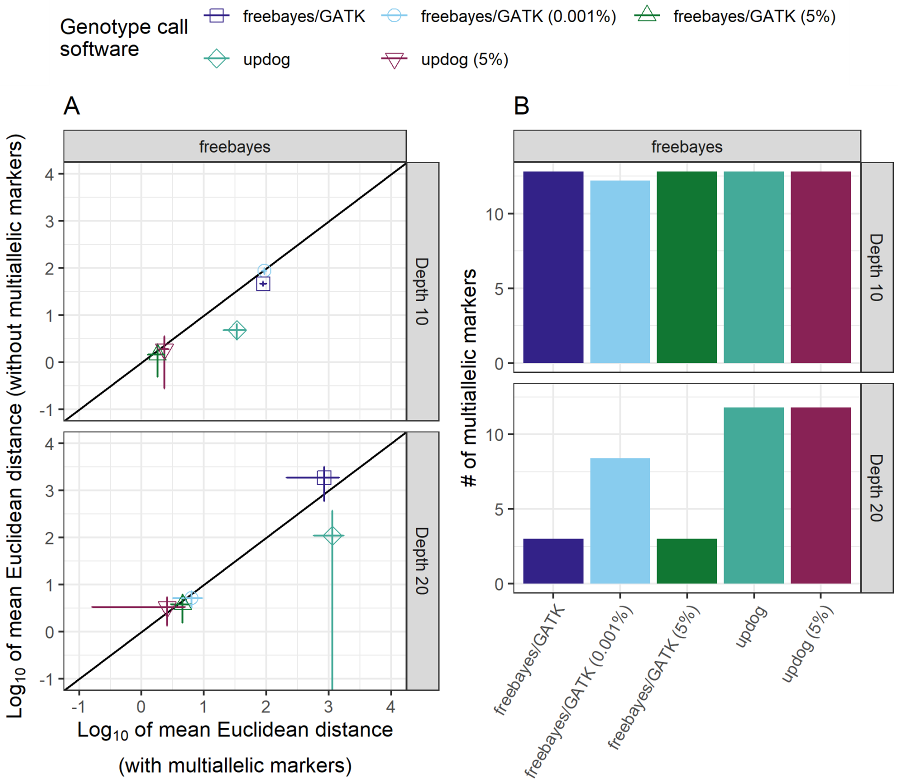

Figure 19. Comparison between simulated data sets with and without multiallelic markers. A: relationship of mean map size between data set with (x-axis) and without (y-axis) multiallelic markers. B: Number of multiallelic markers present in data sets represented in the x-axis of graphic A. The lines crossing the symbols indicate the standard deviation across the five repetitions. The data sets shown in this figure have allele depth counts from the VCF file, segregation distortion, were filtered by a minimum genotype probability of 0.8, and only informative markers.

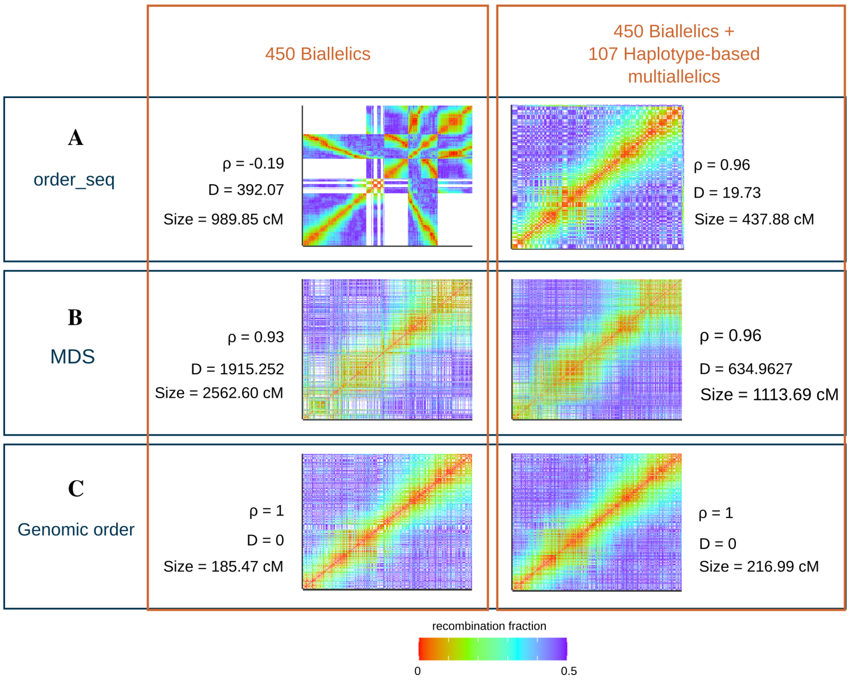

Figure 20. Comparison between ordering algorithms performance in the aspen data set entire linkage group 10 with only biallelic markers, and with biallelic and haplotype-based multiallelic markers. The heatmaps represent the recombination fraction matrix between markers positioned at both axes. In well-ordered linkage groups, we expect a gradient from hot colors in the diagonal (adjacent markers) to cold colors in the upper left and lower right corners. The figure also presents the Spearman rank correlation (ρ) and the Euclidean distances (D) between the estimated map and the map built with markers ordered by the genomic positions. The represented result from order_seq algorithm is only one of the possible results as the procedure is non-deterministic

each combination of software and parameters in each step. If results show issues in any of them, users can re-run the workflow with adapted parameters or include new filters that make sense in their context. Once established the upstream steps based on the app graphics for the built linkage map subset, users can reproduce it for the complete data set, inputting the VCF files from `Reads2Map` into `OneMap`.

Availability of source code and requirements

- Project name: Reads2Map
- Project home page: https://github.com/Cristianetaniguti/Reads2Map
- Main workflows: EmpiricalReads2Map [66] and SimulatedReads2Map [67]
- Operating system(s): Platform independent
- Programming language: WDL and R
- Other requirements: docker or singularity
- License: GNU GPL

Additional files

Supplementary File 1. Emission function for outcrossing.

Supplementary Figure S1. The log_{10} of the CPU time (blue) and the log_{10} of the amount of memory utilized (red) by each task of the Reads2Map workflows when running the simulations with a mean depth of 10. The CPU time is measured with the number of CPUs used times the wall-clock time used.

Supplementary Figure S2. The log_{10} of the CPU time (blue) and the log_{10} of the amount of memory utilized (red) by each task of the Reads2Map workflows when running the simulations with a mean depth of 20. The CPU time is measured with the number of CPUs used times the wall-clock time used.

Supplementary Figure S3. The log_{10} of the CPU time (blue) and the log_{10} of the amount of memory utilized (red) by each task of the Reads2Map workflows when running the aspen empirical data. The CPU time is measured with the number of CPUs used times the wall-clock time used. The filters and linkage map steps were made just with a subset of the data (37% of chromosome 10).

Supplementary Figure S4. The log_{10} of the CPU time (blue) and the log_{10} of the amount of memory utilized (red) by each task of the Reads2Map workflows when running the rose empirical data. The CPU time is measured with the number of CPUs used times the wall-clock time used. The filters and linkage map steps were made just with a subset of the data (37% of chromosome 1).

Supplementary Figure S5. Reference (x-axis) and alternative (y-axis) allele depth distribution for all progeny individuals and a subset of 5% of the markers in rose and aspen data considering the read counts from VCF and from BAM files. Colors represent the estimated genotype by the genotype calling methods. Percentages of each genotype in the entire data set are shown for progeny and parental genotypes in the top right of each graphic.

Supplementary Figure S6. Supplementary figure S5 continued.

Supplementary Figure S7. Reference (x-axis) and alternative (y-axis) allele depth distribution for all progeny individuals and a subset of 25% of the markers from a single simulated family data without segregation distortion, with mean depth of 10 and 20 and considering the read counts from VCF and from BAM files. Colors blue and green show genotypes called correctly by the genotype calling methods, and the colors yellow, orange, and red shows the ones that were called incorrectly. Percentages of correctly and incorrectly genotypes for the entire data set are shown for progeny and also parental genotypes at the top of each graphic.

Supplementary Figure S8. Supplementary Figure S7 continued.

Supplementary Figure S9. Reference (x-axis) and alternative (y-axis) allele depth distribution for all progeny individuals and a subset of 25% of the markers from a single simulated family data with segregation distortion, with mean depth of 10 and 20 and considering the read counts from VCF and from BAM files. Colors blue and green show genotypes called correctly by the genotype calling methods, and the colors yellow, orange, and red shows the ones that were called incorrectly. Percentages of correctly and incorrectly genotypes for the entire data set are shown for progeny and parental genotypes at the top of each graphic.

Supplementary Figure S10. Supplementary Figure S9 continued.

Supplementary Figure S11. ROC curves with the true and estimated genotypes from the five families simulated with mean depth 10 and 20 and the first 8.426 Mb of the chromosome 10 (37% or 38 cM). Here only biallelic markers are considered. The specificity and sensitivity profiles consider different thresholds in the genotype probabilities for each scenario. Higher is the area under the curve, the higher is the genotypes probability reliability. Genotype probabilities thresholds closer to the left superior corner have a higher capacity to differentiate right and wrong genotypes.

Supplementary Figure S12. Supplementary Figure S11 continued.

Supplementary Figure S13. Mean number of corrected identified biallelic by marker types (y-axis) while applying filters by minimum genotypes probability of 0.8, by informativity and replacing AD and GQ VCF field by missing data when GT is missing (x-axis). The markers presented here were obtained using simulated data, `GATK` as SNP and `updog`, `polyRAD`, and `SuperMASSA` genotype calling, with mean depths 10 and 20, with segregation distortion, with allele depth count from VCF. The notation of marker types follows table 2 notation.

Supplementary Figure S14. Supplementary Figure S13 continued. The same information is shown for `freebayes` and `GATK` as genotype call software.

Supplementary Figure S15. The number of markers (y-axis) identified in the first 37% of aspen chromosome 10 while applying filters by minimum genotypes probability of 0.8, by informativity and replacing AD and GQ VCF field by missing data when GT is missing (y-axis). Colors distinguish the marker types according to table 2.

Supplementary Figure S16. The number of markers (y-axis) identified in the first 37% of rose chromosome 1 while applying filters by minimum genotypes probability of 0.8, by informativity and replacing AD and GQ VCF field by missing data when GT is missing (y-axis). Colors distinguish the marker types according to table 2.

Supplementary Figure S17. Mean number of wrongly identified biallelic markers (y-axis) while applying filters by minimum genotypes probability of 0.8, by informativity and replacing AD and GQ VCF field by missing data when GT is missing (x-axis). The numbers on the top of each graphic show the mean total number of correct and wrong markers across the five repetitions. The markers presented here were obtained using simulated data, `GATK` as SNP and genotype calling, with mean depths 10 and 20, segregation distortion, and allele depth count from VCF. The notation of marker types follows table 2 notation.

Supplementary Figure S18. Mean number of multiallelic markers converted from biallelics (y-axis) and how many of them are kept after applying filters by minimum genotypes probability of 0.8, by informativity and replacing AD and GQ VCF field by missing data when GT is missing (y-axis). The markers presented here were obtained using simulated data, `freebayes` as SNP and genotype calling, with mean depths 10 and 20, with and without segregation distortion, with allele depth count from VCF. The notation of marker types follows table 2 notation.

Supplementary Figure S19. The base 10 logarithm of the mean of underestimated and overestimated recombination breakpoints identified in the progeny simulated with a mean depth of 10 and linkage maps built using genotypes and genotypes probabilities

coming from different approaches and filters applied. Colors distinguish the simulations with and without segregation distortion and multiallelic markers and the source of read counts by allele. The blue horizontal line cuts infinite values generated by the logarithmic of zero when there are no wrong breakpoints. The closer the triangles are to the blue line better the method could reproduce the recombination breakpoints number.

Supplementary Figure S20. The base 10 logarithm of the mean of underestimated and overestimated recombination breakpoints identified in the progeny simulated with a mean depth of 20 and linkage maps built using genotypes and genotypes probabilities coming from different approaches and filters applied. Colors distinguish the simulations with and without segregation distortion and multiallelic markers and the source of read counts by allele. The blue horizontal line cuts infinite values generated by the logarithmic of zero when there are no wrong breakpoints. The closer the triangles are to the blue line better the method could reproduce the recombination breakpoints number.

Supplementary Figure S21. Effect of contaminant samples in the map size and in the number of estimated recombination breakpoints range among progeny individuals. The empirical aspen data sets presented in this figure contain multiallelic markers, the allele counts from the VCF file and was filtered by genotype probability higher than 0.8 to keep only informative markers.

Supplementary Figure S22. The relation between filters applied (x-axis) and the mean Euclidean genetic distances (A y-axis) and the number of markers (B y-axis) of the built linkage maps. The simulated data set shown here contains multiallelic markers and segregation distortion.

Supplementary Figure S23. The relation between filters (x-axis) applied and the map size (A y-axis) and the number of markers (B y-axis) of the built linkage maps. The empirical data sets shown here contain multiallelic markers and segregation distortion. The horizontal red line indicates the expected map size (38 cM) for the subset of the genomes used.

Supplementary Figure S24. The effect of the simulated segregation distortion in the maps Euclidean distance. The x-axis shows the Euclidean distance between estimated and simulated maps built for the data sets with simulated segregation distortion. The y-axis shows data sets simulated without the segregation distortion. The lines crossing the symbols indicate the standard deviation across the five repetitions. The data sets contain only biallelic markers and allele depth count from the VCF file; the markers were filtered by genotype probability higher than 0.8 and only informative markers.

Supplementary Figure S25. Comparison of Euclidean distance (y-axis) and the number of markers in maps built with `OneMap` 3.0 and `GUSMap` for the simulated data. The lines crossing the symbols indicate the standard deviation across the five repetitions. The maps built with `OneMap` are represented by the name of the genotype call software that provided the genotypes and their probabilities for `OneMap` multipoint approach of genetic distance estimation. The markers inputted in `OneMap` included multiallelic markers, were filtered by genotype probability higher than 0.8 to keep only informative markers, and the AD and GQ fields were replaced by missing data when GT is missing.

Supplementary Figure S26. Comparison of map size (y-axis) and the number of markers in maps built with `OneMap` 3.0 and `GUSMap` for the empirical data. The maps built with `OneMap` are represented by the name of the genotype call software that provided the genotypes and their probabilities for `OneMap` multipoint approach of genetic distance estimation. The data sets shown here contain only biallelic. Markers inputted in `OneMap` were filtered by genotype probability higher than 0.8 to keep only informative markers. The AD and GQ fields were replaced by missing data when GT was missing. The horizontal red line indicates the expected map size (38 cM) for the subset of the genomes used.

Supplementary Figure S27. Comparison of map size (y-axis) and the number of markers in maps built with `OneMap` 3.0 using empirical data and different upstream software for estimating genotypes and genotypes probabilities. Markers inputted in `OneMap` included multiallelic markers, were filtered by genotype probability higher than 0.8, kept only informative markers, and the AD and GQ fields were replaced by missing data when GT was missing. The horizontal red line indicates the expected map size (38 cM) for the subset of the genomes used.

Supplementary Figure S28. Comparison of Euclidean distance (y-axis) and the number of markers in maps built with `OneMap` 3.0 using simulated data and different upstream software for estimating genotypes and genotypes probabilities. Markers inputted in `OneMap` included multiallelic markers and segregation distortion, were filtered by genotype probability higher than 0.8, kept only informative markers, and the AD and GQ fields were replaced by missing data when GT is missing.

Supplementary Figure S29. Comparison of Euclidean distance (y-axis) and the number of markers in maps built with `OneMap` 3.0 using simulated data and different upstream software for estimating genotypes and genotypes probabilities. Markers inputted in `OneMap` included multiallelic markers and segregation distortion, were filtered by genotype probability higher than 0.8, kept only informative markers, and the AD and GQ fields were replaced by missing data when GT is missing.

Supplementary Figure S30. The total number of recombination breakpoints estimated for each progeny individual of the rose full-sib population with selected pipelines.

Supplementary Figure S31. Recombination fraction matrix heat map obtained for 37% of chromosome 10 of aspen data set by selected pipelines. The heat maps represent the recombination fraction matrix between markers positioned at both axes.

Supplementary Figure S32. Recombination fraction matrix heat map obtained for 37% of chromosome 1 of rose data set by selected pipelines. The heat maps represent the recombination fraction matrix between markers positioned at both axes.

Supplementary Figure S33. A: relation of the Euclidean distance between simulated data set with (x-axis) and without (y-axis) multiallelic markers. B: Number of multiallelic markers present in data sets represented in the x-axis of graphic A. The lines crossing the symbols indicate the standard deviation across the five repetitions. The data sets shown in this figure have allele depth counts from the VCF file, segregation distortion, were filtered by a minimum genotype probability of 0.8, and only informative markers.

Supplementary Figure S34. A: relation of map size between empirical data set with (x-axis) and without (y-axis) multiallelic markers. B: Number of multiallelic markers present in data sets represented in the x-axis of graphic A. The data sets shown in this figure have allele depth counts from the VCF file, were filtered by a minimum genotype probability of 0.8, and only informative markers and AD and GQ VCF fields were replaced by missing when GT is missing.

Abbreviations

GBS: Genotyping-by-Sequencing; PCR: polymerase chain reaction; RADSeq: Restriction-site associated; DNA sequencing; VCF: variant call format; GQ: genotyping quality; GT: genotype; GWAS: genome-wide association; SNP: single nucleotide polymorphism; LD: linkage disequilibrium; QTL: quantitative trait loci; WDL: workflow description language; HPRC: high performance research computing; CPU: central processing unit; HMM: hidden Markov model; EM: expectation-maximization; MAF: minor allele frequency; NGS: Next Generation Sequencing.

Competing Interests

The authors declare that they have no competing interests.

Funding

This work was partially supported by the National Council for Scientific and Technological Development (CNPq - 313269/2021-1); by USDA, National Institute of Food and Agriculture (NIFA), Specialty Crop Research Initiative (SCRI) project "Tools for Genomics-Assisted Breeding in Polyploids: Development of a Community Resource" (Award No. 2020-51181-32156); and by the Bill and Melinda Gates Foundation (OPP1213329) project SweetGAINS. TPO acknowledges funding from the European Union's Horizon 2020 research and innovation program under the Marie Sklodowska-Curie grant agreement No. 801215 and the University of Edinburgh Data-Driven Innovation program part of the Edinburgh and South East Scotland City Region Deal.

Author's Contributions

CHT, MM, RRA, AAFG, GSP and GCF contributed to OneMap package updates. CHT, LMT, GSG, GSP, AAGF, MM, ORL and JL contributed with ideas to design Reads2Map. CHT and LMT developed and optimized the Reads2Map code. CHT developed Reads2MapApp. CHT, TPO, AAFG, ORL, DB, and RRA contributed to elaborate the tested scenarios. CHT, TPO and RRA contributed to analyze the results. CHT wrote the first version of the manuscript. All authors provided helpful discussions for the work and reviewed the manuscript.

Acknowledgements

Portions of this research were conducted with the advanced computing resources provided by Texas A&M High Performance Research Computing and by University of São Paulo Aguia High Performance Computing.

References

1. Glaubitz JC, Casstevens TM, Lu F, Harriman J, Elshire RJ, Sun Q, et al. TASSEL-GBS: a high capacity genotyping by sequencing analysis pipeline. PLoS ONE 2014 2;9:1–11.
2. Catchen J, Hohenlohe PA, Bassham S, Amores A, Cresko WA. Stacks: an analysis tool set for population genomics. Molecular Ecology 2013;22:3124–40.
3. Anderson CB, Franzmayr BK, Hong SW, Larking AC, Stijn TC, Tan R, et al. Protocol: A versatile, inexpensive, high-throughput plant genomic DNA extraction method suitable for genotyping-by-sequencing. Plant Methods 2018 8;14.
4. Andrews KR, Good JM, Miller MR, Luikart G, Hohenlohe PA. Harnessing the power of RADseq for ecological and evolutionary genomics. Nature Reviews Genetics 2016 1;17:81–92.
5. Bresadola L, Link V, Buerkle CA, Lexer C, Wegmann D. Estimating and accounting for genotyping errors in RAD-seq experiments. Molecular Ecology Resources 2020;20:856–870.
6. Baird NA, Etter PD, Atwood TS, Currey MC, Shiver AL, Lewis ZA, et al. Rapid SNP Discovery and Genetic Mapping Using Sequenced RAD Markers. PLoS ONE 2008;3:e3376.
7. Elshire RJ, Glaubitz JC, Sun Q, Poland JA, Kawamoto K, Buckler ES, et al. A robust, simple genotyping-by-sequencing (GBS) approach for high diversity species. PLoS ONE 2011 5;6:e19379.
8. der Auwera GV, O'Connor B. Genomics in the Cloud: Using Docker, GATK, and WDL in Terra. O'Reilly Media, Incorporated; 2020.
9. Rivera-Colón AG, Rochette NC, Catchen JM. Simulation with RADinitio improves RADseq experimental design and sheds light on sources of missing data. Molecular Ecology Resources 2020;p. 1–16.
10. Gerard D, Ferrão LFV, Garcia AAF, Stephens M. Genotyping Polyploids from Messy Sequencing Data. Genetics 2018 11;210:789–807.
11. a Hackett C, Broadfoot LB. Effects of genotyping errors, missing values and segregation distortion in molecular marker data on the construction of linkage maps. Heredity 2003 1;90:33–38.
12. Sturtevant AH. The behavior of the chromosomes as studied through linkage. Zeitschrift für induktive Abstammungs- und Vererbungslehre 1915;13:234–287.
13. Smith GR, Nambiar M. New Solutions to Old Problems: Molecular Mechanisms of Meiotic Crossover Control. Trends in Genetics 2020;36:337–346.
14. Bilton TP, Schofield MR, Black MA, Chagné D, Wilcox PL, Dodds KG. Accounting for Errors in Low Coverage High-Throughput Sequencing Data When Constructing Genetic Maps Using Biparental Outcrossed Populations. Genetics 2018 5;209:65–76.
15. Mollinari M, Garcia AAF. Linkage Analysis and Haplotype Phasing in Experimental Autopolyploid Populations with High Ploidy Level Using Hidden Markov Models. G3: Genes|Genomes|Genetics 2019 10;9:3297–3314.
16. Liao Y, Voorrips RE, Bourke PM, Tumino G, Arens P, Visser RGF, et al. Using probabilistic genotypes in linkage analysis of polyploids. Theoretical and Applied Genetics 2021 8;134:2443–2457.
17. Margarido GRA, Souza AP, Garcia AAF. OneMap: software for genetic mapping in outcrossing species. Hereditas 2007 7;144:78–9.
18. Lander ES, Green P. Construction of multilocus genetic linkage maps in humans. Proc Natl Acad Sci USA 1987;84:2363–2367.
19. Lorenz AJ, Hamblin MT, Jannink JL. Performance of single nucleotide polymorphisms versus haplotypes for genome-wide association analysis in barley. PLoS ONE 2010;5:1–11.
20. Gawenda I, Thorwarth P, Günther T, Ordon F, Schmid KJ. Genome-wide association studies in elite varieties of German winter barley using single-marker and haplotype-based methods. Plant Breeding 2015 2;134:28–39.
21. N'Diaye A, Haile JK, Fowler DB, Ammar K, Pozniak CJ. Effect of Co-segregating Markers on High-Density Genetic Maps and Prediction of Map Expansion Using Machine Learning Algorithms. Frontiers in Plant Science 2017 8;8.
22. Sehgal D, Dreisigacker S. Haplotypes-based genetic analysis: Benefits and challenges. Vavilovskii Zhurnal Genetiki i Selektsii 2019;23:803–808.
23. Abed A, Belzile F. Comparing Single-SNP, Multi-SNP, and Haplotype-Based Approaches in Association Studies for Major Traits in Barley. The Plant Genome 2019;12:190036.
24. Liu N, Zhang K, in Genetics Zhao HBTA. Haplotype-Association Analysis. Genetic Dissection of Complex Traits 2008;60:335–405.
25. Jiang Y, Schmidt RH, Reif JC. Haplotype-Based Genome-Wide Prediction Models Exploit Local Epistatic Interactions Among Markers. G3: Genes|Genomes|Genetics 2018;49:g3.300548.2017.
26. Garrison E, Marth G. Haplotype-based variant detection from short-read sequencing. ArXiv e-prints 2012;p. 9.
27. McKenna A, Hanna M, Banks E, Sivachenko A, Cibulskis K, Kernytsky A, et al. The Genome Analysis Toolkit: A MapReduce framework for analyzing next-generation DNA sequencing data. Genome Research 2010 9;20:1297–1303.
28. Clark LV, Lipka AE, Sacks EJ. polyRAD: Genotype Calling with Uncertainty from Sequencing Data in Polyploids and Diploids. G3: Genes|Genomes|Genetics 2019;9:g3.200913.2018.
29. Serang O, Mollinari M, Garcia AAF. Efficient exact maximum a posteriori computation for bayesian SNP genotyping in polyploids. PLoS ONE 2012;7:1–13.
30. Patterson M, Marschall T, Pisanti N, van Iersel L, Stougie L, Klau GW, et al. WhatsHap: Weighted Haplotype Assembly for Future-Generation Sequencing Reads. Journal of Computational Biology 2015;22:498–509.
31. Voss K, Gentry J, Auwera GVD. Full-stack genomics pipelining with GATK4+ WDL+ Cromwell [version 1; not peer reviewed]. F1000Research 2017;p. 4.
32. Merkel D. Docker : Lightweight Linux Containers for Consistent Development and Deployment Docker : a Little Background Under the Hood. Linux Journal 2014;2014:2–7.
33. Kurtzer GM, Sochat V, Bauer MW. Singularity: Scientific containers for mobility of compute. PLOS ONE 2017 5;12:e0177459.
34. Broman KW, Wu H, Sen S, Churchill GA. R/qtl: QTL mapping in experimental crosses. Bioinformatics 2003;19:889–890.
35. Broman KW, Śaunak Sen, Sen S. A Guide to QTL Mapping with R/qtl, vol. 66. Springer New York; 2009.
36. Maliepaard C, Jansen J, Ooijen JWV. Linkage analysis in a full-sib family of an outbreeding plant species : overview and consequences for applications. Genetical Research 1997;70:237–250.
37. Baum E, Petrie T, G S, N W. A Maximization Technique Occurring in the Statistical Analysis of Probabilistic Functions of Markov Chains. The Annals of Mathematical Statistics 1970;41:164–171.
38. Schiffthaler B, Bernhardsson C, Ingvarsson PK, Street NR. BatchMap: A parallel implementation of the OneMap R package for fast computation of F1 linkage maps in outcrossing species. PLoS ONE 2017;12:1–12.
39. Zhigunov AV, Ulianich PS, Lebedeva MV, Chang PL, Nuzhdin SV, Potokina EK. Development of F1 hybrid population and the high-density linkage map for European aspen (Populus tremula L.) using RADseq technology. BMC Plant Biology 2017;17.
40. Young EL, Lau J, Bentley NB, Rawandoozi Z, Collins S, Windham MT, et al. Identification of QTLs for Reduced Susceptibility to Rose Rosette Disease in Diploid Roses. Pathogens 2022 6;11:660.
41. Tuskan GA, DiFazio S, Jansson S, Bohlmann J, Grigoriev I, Hellsten U, et al. The Genome of Black Cottonwood, Populus trichocarpa. Science 2006 9;313:1596–1604.
42. Saint-Oyant LH, Ruttink T, Hamama L, Kirov I, Lakhwani D, Zhou NN, et al. A high-quality genome sequence of Rosa chinen-

sis to elucidate ornamental traits. Nature Plants 2018 7;4:473–484.
43. Martin M. Cutadapt removes adapter sequences from high-throughput sequencing reads. EMBnetjournal 2011 5;17:10.
44. Hyman JM. Accurate Monotonicity Preserving Cubic Interpolation. SIAM Journal on Scientific and Statistical Computing 1983 12;4:645–654.
45. Wu R, Ma CX, Wu SS, Zeng ZB. Linkage mapping of sex-specific differences. Genetical research 2002;79:85–96.
46. Voorrips RE, Maliepaard CA. The simulation of meiosis in diploid and tetraploid organisms using various genetic models. BMC Bioinformatics 2012 12;13:248.
47. Haldane JBS. The combination of linkage values, and the calculation of distance between linked factors. Journal of Genetics 1919;8:299–309.
48. Best K, Oakes T, Heather JM, Shawe-Taylor J, Chain B. Computational analysis of stochastic heterogeneity in PCR amplification efficiency revealed by single molecule barcoding. Scientific reports 2015 10;5:14629.
49. Glenn TC. Field guide to next-generation DNA sequencers. Molecular Ecology Resources 2011;11:759–769.
50. Li H. seqtk: Toolkit for processing sequences in FASTA/Q formats. seqtk GitHub repository 2020;https://github.com/lh3/seqtk.
51. Li H. Aligning sequence reads, clone sequences and assembly contigs with BWA-MEM. ArXiv 2013;1303.
52. Li H, Handsaker B, Wysoker A, Fennell T, Ruan J, Homer N, et al. The sequence alignment/map format and SAMtools. Bioinformatics 2009;25:2078–2079.
53. Ros-Freixedes R, Gonen S, Gorjanc G, Hickey JM. A method for allocating low-coverage sequencing resources by targeting haplotypes rather than individuals. Genetics Selection Evolution 2017;49:1–17.
54. Knaus BJ, Grünwald NJ. vcfR: a package to manipulate and visualize variant call format data in R. Molecular Ecology Resources 2017 1;17:44–53.
55. Preedy KF, Hackett CA. A rapid marker ordering approach for high-density genetic linkage maps in experimental autotetraploid populations using multidimensional scaling. Theoretical and Applied Genetics 2016;.
56. Berkson J. Application of the Logistic Function to Bio-Assay. Journal of the American Statistical Association 1944 3;39:357–365.
57. Mollinari M, Margarido GRA, Vencovsky R, Garcia AAF. Evaluation of algorithms used to order markers on genetic maps. Heredity 2009;103:494–502.
58. Guyader V, Fay C, Rochette S, Girard C. golem: A Framework for Robust Shiny Applications. Golem GitHub repository 2022;https://github.com/ThinkR-open/golem.
59. DePristo MA, Banks E, Poplin R, Garimella KV, Maguire JR, Hartl C, et al. A framework for variation discovery and genotyping using next-generation DNA sequencing data. Nature Genetics 2011;43:491–8.
60. Kagale S, Koh C, Clarke WE, Bollina V, Parkin IAP, Sharpe AG. Analysis of Genotyping-by-Sequencing (GBS) Data. Plant Bioinformatics 2016;p. 269–284.
61. Institute B. Picard Tools. Broad Institute, GitHub repository 2009;https://github.com/broadinstitute/picard.
62. Duncavage EJ, Coleman JF, de Baca ME, Kadri S, Leon A, Routbort M, et al. Recommendations for the Use of In silico Approaches for Next Generation Sequencing Bioinformatic Pipeline Validation: A Joint Report of the Association for Molecular Pathology, Association for Pathology Informatics, and College of American Pathologists. The Journal of molecular diagnostics : JMD 2022 10;.
63. Grattapaglia D, Sederoff R. Genetic linkage maps of Eucalyptus grandis and Eucalyptus urophylla using a pseudo-testcross: mapping strategy and RAPD markers. Genetics 1994 8;137:1121–37.
64. Gazaffi R, Margarido GRA, Pastina MM, Mollinari M, Garcia AAF. A model for quantitative trait loci mapping, linkage phase, and segregation pattern estimation for a full-sib progeny. Tree Genetics and Genomes 2014;10:791–801.
65. Os H, Stam P, Visser RGF, Eck HJ, Os HV, Stam P, et al. RECORD: a novel method for ordering loci on a genetic linkage map. Theoretical and Applied Genetics 2005;112:30–40.
66. Taniguti CH. EmpiricalReads2Map. WorkflowHub 2022;https://doi.org/10.48546/WORKFLOWHUB.WORKFLOW.409.1.
67. Taniguti CH. SimulatedReads2Map. WorkflowHub 2022;https://doi.org/10.48546/WORKFLOWHUB.WORKFLOW.410.1.

Reference PDF
Click here to access/download;Manuscript;sxjrggcvcwdtfwdjvxqtqvgtfdfvrkq

(GIGA)^n SCIENCE

GigaScience, 2017, 1–24

doi: xx.xxxx/xxxx
Manuscript in Preparation

Developing best practices for genotyping-by-sequencing analysis using linkage maps as benchmarks

Cristiane Hayumi Taniguti[1,2,*], Lucas Mitsuo Taniguti[1,3], Rodrigo Rampazo Amadeu[1], Jeekin Lau[2], Gabriel de Siqueira Gesteira[1,4], Thiago de Paula Oliveira[5], Getulio Caixeta Ferreira[1], Guilherme da Silva Pereira[7], David Byrne[2], Marcelo Mollinari[4], Oscar Riera-Lizarazu[2] and Antonio Augusto Franco Garcia[1,*]

[1]Department of Genetics, University of São Paulo, Brazil and [2]Department of Horticultural Sciences, Texas A&M University, College Station, TX, USA and [3]Mendelics Genomic Analysis, São Paulo, Brazil and [4]Bioinformatics Research Center, Department of Horticultural Sciences, North Carolina State University, Raleigh, NC, USA and [5]Roslin Institute, University of Edinburgh, Scotland and [7]Department of Agronomy, Federal University of Viçosa, Brazil

[*]chtaniguti@tamu.edu; augusto.garcia@usp.br

Abstract

Background Genotyping-by-Sequencing (GBS) provides affordable methods for genotyping hundreds of individuals using millions of markers. However, this challenges bioinformatic procedures that must overcome possible artifacts such as the bias generated by PCR duplicates and sequencing errors. Genotyping errors lead to data that deviate from what is expected from regular meiosis. This, in turn, leads to difficulties in grouping and ordering markers resulting in inflated and incorrect linkage maps. Therefore, genotyping errors can be easily detected by linkage map quality evaluations.
Results We developed and used the Reads2Map workflow to build linkage maps with simulated and empirical GBS data of diploid outcrossing populations. The workflows run `GATK` and `freebayes` for SNP calling and `updog`, `polyRAD`, and `SuperMASSA` for genotype calling, and `OneMap` and `GUSMap` to build linkage maps. Using simulated data, we observed which genotype call software fails in identifying common errors in GBS sequencing data and proposed specific filters to better handle them. We tested whether it is possible to overcome errors in a linkage map using genotype probabilities from each software or global error rates to estimate genetic distances with an updated version of `OneMap`. We also evaluated the impact of segregation distortion, contaminant samples, and haplotype-based multiallelic markers in the final linkage maps. The results showed a low impact of segregation distortion in the linkage map quality, improvements in ordering markers with haplotype-based multiallelic markers, and improved maps with expected size using reliable genotype probabilities or a global error rate of 5%.
Conclusions The pipelines results in each scenario changed according to the data set used, indicating that optimal pipelines and parameters are dataset-dependent and cannot be generalized to all GBS data sets. The Reads2Map workflow can reproduce the analysis in other GBS empirical data sets where users can select the pipeline and parameters adapted to their data context. The Reads2MapApp shiny app provides a graphical representation of the results to facilitate their interpretation.

Key words: genotyping error; haplotype; genetic maker; multiallelic

Compiled on: November 29, 2022.
Draft manuscript prepared by the author.

Introduction

Advances in sequencing technologies and the development of different genome-reduced representation library protocols result in millions of genetic markers from hundreds of samples in a single sequencing run [1, 2, 3, 4]. Increasing the number of markers and individuals genotyped can enhance the capacity of linkage maps to locate recombination events that occur, resulting in higher map resolution and better statistical power for the localization of QTL in further analysis. This large amount of data and genotyping errors common with genotyping-by-sequencing approaches [5] increases the need for computational resources and multiple bioinformatic tools.

Genotyping errors are frequent when high-throughput sequencing technology is applied to reduced representation libraries. There are a variety of protocols to create these types of libraries [4], called Restriction-site Associated DNA sequencing (RADseq) or genotyping-by-sequencing (GBS) [6, 7]. Generally, one or more restriction enzymes are used to digest the sample DNA. The resulting DNA fragments are filtered by size, connected to adaptors and barcodes, amplified by PCR, and sequenced. Consequently, most sequences obtained are PCR duplicates of the regions around the enzyme cut site. By relying on duplicates to increase sequencing depth, such methods introduce errors and a sequencing bias towards one of the alleles due to variabilities in the PCR amplification. These errors are hard to detect by bioinformatic tools [8, 9].

To overcome genotyping errors coming from GBS methods, genotype calling software model sequencing error, allelic bias, overdispersion, outlying observations, and the population Mendelian expected segregation [10]. Building a genetic map with genotypes obtained using these methods can be a powerful tool to validate their efficiency. Wrong decisions or inefficient methods in all steps before linkage map building can be identified in the resulting map as errors that dissociate the map properties from biological processes. For example, genotyping errors generate inflated map sizes that show an excessive number of recombination breakpoints during meiosis [11]. The first genetic map studies by Morgan and Sturtevant [12] discovered that crossing-overs are unlikely to happen too close to each other, a phenomenon named interference. Later studies describing the meiotic molecular mechanisms confirmed the low expected number of recombination breaks in a single event [13].

Recently developed approaches to build linkage maps [14, 15, 16] were implemented in `OneMap` [17] 3.0 package. They use quantitative genotype probability measurements rather than the traditional qualitative genotypic information from SNP and genotype calling methods to account for genotyping errors and provide higher-quality genetic maps. These probabilities can be applied in different ways: using the probability of each possible genotype (PL field in VCF format); using an error probability associated with the called genotype (GQ field in VCF format); or using a global error rate that will be applied to all genotypes. Nevertheless, even using these approaches, building a linkage map will succeed only if the upstream software can identify the errors and provide reliable genotypes or their probabilities.

The biallelic codominant nature of SNPs is another characteristic of high-throughput markers that can affect linkage map building of outcrossing species. Although biallelic markers can distinguish only two haplotypes, the mapping population of outcrossing diploid species inherits two haplotypes with combinations of four different parental haplotypes. With biallelic markers, the observed parental genotypes are limited to types $ab \times ab$, $ab \times aa$, and $aa \times ab$. When one of the parents is homozygous ($ab \times aa$ and $aa \times ab$), it is impossible to observe the crossing-over change for this uninformative parent. So this is taken as missing information (non-measurable crossing-overs) for linkage map building if only two-point information is considered. Therefore, building a linkage map with only biallelic markers requires a multi-point approach that uses loci information with both parents heterozygous ($ab \times ab$) to estimate the recombination of loci where one parent is homozygous, and the recombination information is missing for closely linked loci. The multi-point approach applies likelihood computations involving several loci and has been successfully used since the seminal publication of Lander and Green [18]. The approach makes it possible to identify the four different parental haplotypes by phasing the biallelic information so that the SNPs can be used to identify all the allelic diversity.

Other approaches to overcome the low informativeness of biallelic markers involve combining adjacent biallelic markers in the same disequilibrium block (high LD) into a single multiallelic haplotype. These haplotype-based markers showed higher accuracy in association analysis than individual biallelic SNPs [19, 20, 21, 22, 23, 24, 25]. N'Diaye et al. [21] and Jiang et al. [25] pointed out several advantages of haplotype-based markers, including the higher capacity to identify epistatic interactions, the presence of more information to estimate identical-by-descent alleles and the reduction of the number of statistical tests to perform.

Despite many software available for estimating genotype probabilities [26, 2, 27, 26, 28, 29, 10] and haplotype-based multiallelic markers [26, 30], there are no recommendations yet about which combination and choice of parameters are the best for building linkage maps. Therefore, this work evaluates the consequences of building maps by applying genotype probabilities and haplotype-based markers from different software and parameters. To achieve these, we implemented new features in `OneMap` [17], a widely-used software for building maps, and developed the `Reads2Map` workflow. We were able to make recommendations to users to obtain better linkage maps in several situations, such as low and high-depth sequencing, with and without segregation distortion, contaminant samples, and multiallelic markers, and using different bioinformatic software to perform the SNP and genotype calling.

Material and Methods

We built two workflows using Workflow Description Language (WDL) [31] to perform sequence alignment, SNP and genotype calling, and linkage map building: EmpiricalReads2Map, for evaluating empirical (real) data sets; and SimulatedReads2Map, to evaluate simulated data sets (figure 1). Both share the same sub-workflows for most of the steps, allowing users to evaluate software and parameters in an organized and efficient way. WDL workflows can be executed using Cromwell Execution Engine [31], Docker [32], and Singularity [33] containers. We ran the analysis testing workflows on two high-performance computers (Texas A&M University HPRC, University of São Paulo Águia Cluster). The CPU and memory amount utilized by each workflow task in the Texas A&M HPRC is shown in Supplementary figures 1-4. The workflows are available at https://github.com/Cristianetaniguti/Reads2Map. For the linkage map building step, we implemented updates in `OneMap` package version 3.0 (https://CRAN.R-project.org/package=onemap) and used this version in the workflows. We also developed the Reads2MapApp shiny app (https://github.com/Cristianetaniguti/Reads2MapApp). We used it to upload the final workflow output and visualize summary statistics about the resulting linkage maps, intermediary steps, and workflow performance.

Genotype probabilities in `OneMap` 3.0 Hidden Markov Model

With a combination of a hidden Markov model (HMM) and the expectation-maximization algorithm (EM) [18], `OneMap` [17] can perform multipoint estimation of map genetic distance for F2, backcross, RILs, and outcrossing populations. For the multipoint esti-

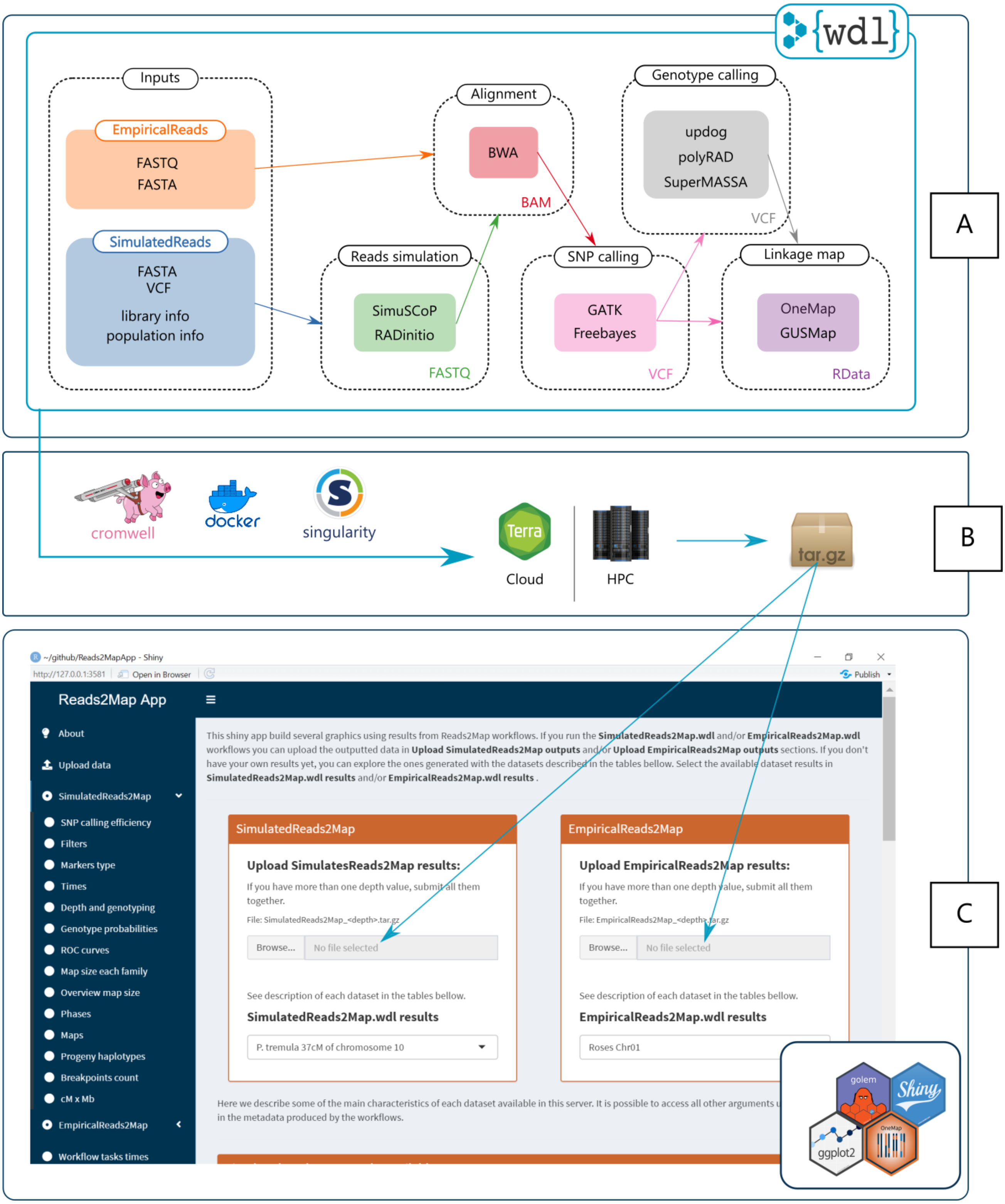

Figure 1. A: Tasks of the two main Reads2Map workflows: EmpiricalReads2Map.wdl and SimulatedReads2Map.wdl. B: Tools to run the workflows on the Cloud (`https://app.terra.bio/` platform) or in High-Performance Computing (HPC) environments. C: The Reads2Map shiny app has as input the outputs of the workflows. It builds several descriptive graphics to evaluate the best upstream software combination for linkage map construction.

mation, OneMap algorithms use code adapted from R/QTL package [34].

In short, the latent variable G_i, $i = 1, ..., n$, denotes the true underlying genotypes for the individual at a set of n ordered loci; O_i is the observed variable of the molecular phenotype (observed genotypes) for the locus i. The HMM can be represented as [35]:

$$P(O|G_i = g_i) = \sum_{g_1} ... \sum_{g_i-1} \sum_{g_i+1} ... \sum_{g_n} \pi(g_1) \prod_{j=1}^{n-1} t_j(g_j, g_{j+1}) \prod_{j=1}^{n} e(g_j, O_j) \quad (1)$$

The initial probability $\pi(g_1)$ is the probability of having a given genotype for the first locus (G_1), and its value depends on the cross-type. For example, for an outcrossing population, this value will be 0.25, assuming a uniform distribution of all four possible genotypes (AA, BA, AB, and BB). The same reasoning applies to backcross data, with probabilities of 0.5 since there are only two possible genotypes (AA and AB).

The transition probability $t_j(g_j, g_{j+1})$ is the probability of the genotype in a locus ($G_{j=i+1}$) changing to the next locus genotype (G_{j+1}). The initial value for this probability is based on the phase, and recombination fraction estimated by a two-point approach using maximum likelihood estimators [36], and is updated after iterations of the EM algorithm. The emission probability $e(g_j, O_j)$ is the probability of the observed variable given the genotype. This probability is defined by an associated genotyping error (see Supplementary file 1). The OneMap software previous to version 3.0 considered this error probability as a single value of 10^{-5} for every genotype. In version 3.0, this value is kept as default to maintain the code reproducibility. But it is noteworthy that this probability can be unreliable in several situations when the genotypes are more prone to errors, especially for new genotyping technology (e.g. GBS data). OneMap 3.0 allow users to provide individual values of error probabilities in the emission probability of the HMM for each genotype or marker, having a potential impact on the results. Using the *create_probs* function, users can provide three types of values: one global value, which was the previous default (*global_error*); an error probability for each inferred genotype (*genotypes_error*); or genotype probabilities for each possible genotype in individuals (*genotypes_probs*). We tested the consequences of building maps applying different genotype probabilities coming from five different genotype caller software, a global error rate of 0.05, and the old default value of 10^{-5}.

Here we used GATK [27], freebayes [26], polyRAD [28], SuperMASSA [29] and updog [10] to estimate the genotypes and genotypes probabilities. For GATK and freebayes caller, we used the Phred score genotype error (GQ FORMAT value) converted to probabilities. The software polyRAD, SuperMASSA and updog use the known population's structure (in our case F_1) as *a priori* information to increase the accuracy of the estimated genotypes.

OneMap uses the forward-backward algorithm [37] to compute the HMM combined with the expectation-maximization algorithm (EM). Since version 3.0, OneMap presents the possibility to parallelize the HMM using the approach described in [38]. It parallelizes the procedure into a maximum of four cores. We used this new OneMap feature to estimate the genetic distances. We also implemented new functions for linkage maps quality diagnostics such as interactive plots for recombination fraction matrices, progeny haplotypes representation, and counts of the recombination breakpoints in progeny. We compared OneMap 3.0 capacity of estimating accurate genetic distances with the GUSMap package estimations since it also uses an HMM to account for errors present in sequencing data.

Empirical data analysis

We ran EmpiricalReads2Map workflow using two empirical data sets that already have linkage maps built. They are GBS data sets from a bi-parental diploid F_1 full-sib mapping populations of aspen (*Populus tremula L.*) [39] (BioProject PRJNA395596), and rose (*Rosa spp.*) [40]. The aspen data set comes from an intraspecific cross of two *Populus tremula* genotypes. The GBS libraries were built using *HindIII* and *NalI* enzymes and sequenced as 150 base pair single-end reads on an Illumina HiSeq2500. Eight library replicates were built and sequenced for the parents and only one for each of the 116 F1 offspring. The data set includes six samples erroneously sequenced as part of the progeny and later identified as contaminants. An average read depth of approximately 6x for progeny and 58x for parental samples were observed from the sequencing process. The *Populus trichocarpa* genome version 3.0 [41] was used as a reference for the sequence's alignment.

The diploid roses data set comprises 138 individuals from the cross between a Texas A&M breeding line J06-20-14-3 (J14-3) and cultivar Papa Hemeray (PH). GBS libraries were built with *NgoMIV* enzyme and sequenced as a 113 base pair single-end read on a HiSeq2500. The parent J14-3 was repeated twice, and the PH sample three times. An average read depth of approximately 94x for progeny and 528x for parental samples was observed from the sequencing process. The *Rosa chinensis* v1.0 genome assembly [42] was used as a reference genome to align the sequences.

The sequencing reads of the two empirical data sets were filtered using the Stacks plugin process_radtags [2] to filter sequences by the presence of the restriction site and sequencing quality. The reads were discarded if the average quality score of 50% of its length was below the Phred score of 10 (or 90% probability of being correct). The software cutadapt [43] was used to remove adapters and filter by a minimum read length of 64 bp. The sequences were then evaluated in our EmpiricalReads2Map workflow.

Each time the EmpiricalReads2Map workflow is executed, it considers all the pipeline combinations generating 34 maps with combinations of SNP caller (GATK and freebayes), genotype caller (GATK/freebayes, polyRAD, updog, SuperMASSA), source of the reads counts (VCF and BAM files), and map builder packages (OneMap and GUSMap). The output provides maps built with genotype call software genotype probabilities, with 5% and 0.001% of global error rate in the HMM chain.

We executed the EmpiricalReads2Map workflows in the presence and absence of haplotype-based multiallelic markers and applied four different marker filtering methods. For the aspen data set, we also executed the workflows for every scenario in the presence of the contaminant samples. Therefore, the experiment has a total of 3 (data sets: rose, aspen and aspen with contaminants) × 2 (presence/absence of multiallelic markers) × 4 (filter methods - see details below) × 34 = 816 maps built for the first 8.426 Mb of chromosome 10 of *Populus trichocarpa* genome and the first 25 Mb (37%) of chromosome 1 *Rosa chinensis* reference genome. Table 1 shows an overview of the notations used to refer to each evaluated scenario. It is important to mention that this represents what users will find in building maps for the whole genome; a sample was required to reduce the computation burden.

GBS data simulation

The first step of the SimulatedReads2Map workflow is to perform simulations of a mapping population, GBS libraries, and sequences. The simulation is based on a given reference genome chromosome sequence. If a reference linkage map and a VCF file are provided, the workflow simulates the marker genetic distances and parental genotype frequencies based on them. A cubic spline interpolation with the Hyman method [44] is applied to simulate the centimorgan position for each marker's physical position based on this same relation on the reference linkage map provided.

We based our simulation analysis on the first 37% of the chromosome 10 sequence of *Populus trichocarpa* version 3.0, which comprehends a sequence with 8.426 Mb from a total chromosome size

Table 1. Notation used to refer to each evaluation scenario in empirical and simulated data sets.

Step	Notation	Description
Reads simulations	depth 10	Mean read depth used to simulate the data set
	depth 20	
SNP calling	freebayes	Software used to identify the variants
	GATK	
	BAM	Source files of allele depth information
	VCF	
Genotype calling	polyRAD	Software used to perform the estimation of genotype for a given allele depth information
	SuperMASSA	
	updog	
	freebayes/ GATK	Software used to genotype calling is the same that performed the SNP calling
Map building	polyRAD	Maps built with genotypes probabilities from polyRAD
	SuperMASSA	Maps built with genotypes probabilities from SuperMASSA
	updog	Maps built with genotypes probabilities from updog
	freebayes/ GATK	Maps built with genotype probabilities from freebayes if freebayes was used for SNP calling or GATK if GATK was.
	polyRAD (5%)	Maps built with genotypes from polyRAD and global error of 0.05
	SuperMASSA (5%)	Maps built with genotypes from SuperMASSA and global error of 0.05
	updog (5%)	Maps built with genotypes from updog and global error of 0.05
	freebayes/ GATK (5%)	Maps built with genotypes from freebayes or GATK and global error of 0.05
	freebayes/ GATK (0.001%)	Maps built with genotypes from freebayes or GATK and global error of 0.00001

of about 23 Mb. This sequence comprises 38 cM (21%) of the linkage group 10 reference linkage map built using the aspen empirical data [39]. Due to the computational resources needed to build such a high number of maps, we used only a subset of the data to finish the analysis in a reasonable time. Chromosome 10 was randomly chosen.

We simulated markers with different expected segregation patterns according to parental genotypes in each locus. Table 2 shows the notation for each possible marker type in an outcrossing diploid population. The SimulatedReads2Map workflow simulates parental haplotypes using the same proportion of marker types identified in the empirical VCF file. This approach overcomes the missing data present in the empirical data set. The final VCF file used as a reference to the simulations contains 810 markers (126 B3.7, 263 D1.10, 278 D2.15, and 143 non-informative markers with both parents homozygous), which results from the aspen empirical data GATK SNP calling, filtered by a maximum of 25% of missing data and MAF of 5%.

Table 2. Marker types according to parental genotype combinations and progeny segregation. The letters "a", "b", "c" and "d" represent different alleles and the letter "o" represents null alleles. Adapted from [45].

			Parents	Progeny	
Marker type			Cross	Observed genotypes	Expected segregation
A		1	ab x cd	ac,ad,bc,bd	1:1:1:1
		2	ab x ac	a,ac,ba,bc	1:1:1:1
		3	ab x co	ac,a,bc,b	1:1:1:1
		4	ao x bo	ab,a,b,o	1:1:1:1
B	B_1	5	ab x ao	ab,2a,b	1:2:1
	B_2	6	ao x ab	ab,2a,b	1:2:1
	B_3	7	ab x ab	a,2ab,b	1:2:1
C		8	ao x ao	3a,o	3:1
D	D_1	9	ab x cc	ac,bc	1:1
		10	ab x aa	a,ab	1:1
		11	ab x oo	a,b	1:1
		12	bo x aa	ab,a	1:1
		13	ao x oo	a,o	1:1
	D_2	14	cc x ab	ac,bc	1:1
		15	aa x ab	a,ab	1:1
		16	oo x ab	a,b	1:1
		17	aa x bo	ab,a	1:1
		18	oo x ao	a,o	1:1

PedigreeSim v2.1 software [46] is implemented in the workflow to simulate the meiosis events and generate an F_1 progeny based on the provided genetic map and simulated parental haplotypes. We did not consider the interference in meiotic events (Haldane [47] mapping function). PedigreeSim output files were converted to VCF files using Reads2MapTools (available at https://github.com/Cristianetaniguti/Reads2MapTools) R package function pedsim2vcf.

While converting the files, the pedsim2vcf function can also simulate segregation distortion by applying a selection strength. For that, a high number of individuals in the progeny have to be simulated with the PedigreeSim software and one or more loci to be under a given selection intensity. In our study, we targeted a final population size of 200 individuals. For that, we simulated 50 $\times$ 200 individuals and applied a selection intensity of 50% in the 30th marker, eliminating 50% of the genotypes containing one of the alleles. Then, 200 individuals of the resulting population are randomly selected to compose the mapping population. We used this feature to compare software performance in segregation distortion.

The VCF file output by pedsim2vcf is used as input in RADinitio software together with the reference genome sequence. RADinitio adds the VCF polymorphisms in the reference genome sequence and simulates the GBS sequences. It uses the inherited efficiency model [48] to simulate a PCR-amplified pool of molecules. The model includes the heterogeneity of the PCR amplification and the polymerase substitution errors. Next, RADinitio applies the user-defined ratio between DNA original molecules to be sequenced and PCR duplicates to create a distribution that will define the number of times the pool of loci is sampled, the number of duplicate molecules that are generated from a RAD locus template, and the distribution of PCR errors in the resulting reads. We defined the default parameter with a proportion of 4:1. Besides the PCR errors inserted during the pool sampling, the software also includes a commonly observed error pattern, where the 3' end of the read accumulates more errors than the 5' [49]. We tested different values of PCR cycles (5, 9, and 14) and mean depth (5, 10, and 20) to simulate the

FASTA files. We set the other simulation parameters to obtain 150 bases of read length, sequence size of 350, and restriction enzymes *HindIII* and *NalIII*. The mean read depth parameter for the parental samples was eight times higher than the progeny. The combination of RADinitio parameters that produced results closer to those observed in empirical data was selected to perform simulations with and without segregation distortion, five repetitions (five families), and two average sequencing depths (10 and 20) and 5 PCR cycles.

RADinitio does not output the sequence quality scores, so we converted the FASTA file format to FASTQ format, including a Phred score of 40 for every base simulated using seqtk [50] software. After obtaining the FASTQ files, the SimulatedReads2Map workflow followed the same tasks as the EmpiricalReads2Map, with alignment, SNP and genotype calling, and linkage map build. The SimulatedReads2Map workflow makes comparisons between real and estimated results within each step. The comparisons made during the workflow can be visualized in the shiny app Reads2MapApp.

Similarly to the EmpiricalReads2Map, the SimulatedReads2Map workflow generates maps for each combination of SNP and genotype call and linkage map building software. However, the total number of maps generated is multiplied by two because the workflows build maps with and without loci that were wrongly identified as polymorphic due to sequencing errors (false-positive markers). We also execute the SimulatedReads2Map workflow in the presence and absence of haplotype-based multiallelic markers, segregation distortion, and four methods for marker filtering. Therefore, the experiment has a total of 5 (repetitions) $\times$ 2 (average depths) $\times$ 2 (presence/absence of multiallelic markers) $\times$ 2 (with and without segregation distortion) $\times$ 4 (filters method - see details below) $\times$ 68 = 10,880 maps built for the first 8.426 Mb of chromosome 10 of *Populus trichocarpa* genome. Table 1 shows an overview of the notations used to refer to each evaluated scenario.

SNP calling

First, the FASTQ sequences are aligned with BWA-MEM [51] to their respective reference genomes. The workflow uses samtools [52] to merge the alignment of the same samples BAM files, keeping the libraries identification on the BAM header and filtering out reads with MAPQ < 10. After the alignment, BAM files for each sample are used as inputs for sub-workflows with GATK and freebayes approaches. One of the sub-workflow reproduces GATK joint genotyping via HaplotypeCaller, GenomicsDBImport, and GenotypeGVCFs tools and applies the suggested hard-filtering procedures [8]. The other sub-workflow runs freebayes parallelized by reference genome intervals. After obtaining the VCF files, indels marker positions are left-aligned and normalized with BCFtools, and multiallelic markers are separated into a new VCF file.

GATK and freebayes may introduce bias towards the reference allele when used to process low-coverage sequence data. GATK inserts the bias when reads are filtered in the local re-assembly step to avoid sequencing errors [53]. To overcome the bias during the genotype calling, the workflow applies two measures of allele depth, one from VCF and the other from BAM files. BCFtools is used to find the read depths information for each allele in BAM files and update the allele depths information in the AD (allele depth) field of the VCF file. Therefore, each SNP calling method results in three VCFs: i) biallelic markers with read counts outputted by the SNP callers, ii) biallelic markers with counts from BAM files, iii) multiallelic markers.

Genotype calling

For the empirical data sets, the alignment and SNP calling steps were performed with entire data sets, but for the next steps, we selected just a subset of markers (the first 8.426 Mb or 37%) of *Populus trichocarpa* chromosome 10 and the first 25 Mb (37%) of *Rosa chinensis* chromosome 1 reference genomes. The markers were filtered by minor allele frequency (MAF) of 5%, and maximum missing data allowed of 25%. The VCF files with biallelic markers from freebayes and GATK, and with read counts source from VCF and BAM files were the input for the genotype caller software polyRAD, SuperMASSA, and updog.

To use the polyRAD approach, the VCF files were imported using VCF2RADdata without applying any filters or considering phase information. The polyRAD model was run with PipelineMapping2Parents default arguments which assume an F_1 bi-parental population. The function Export_MAPpoly was used to export the genotype probabilities. The vcfR package [54] and custom R (function polyRAD_genotype_vcf in Reads2MapTools package) code were used to store outputted genotypes and their probabilities in a new VCF file. We also adapted SuperMASSA scripts to output the genotype probabilities information. The modified version is available in Reads2MapTools package. A wrapper function called supermassa_genotype, available in the package, can run the model in parallel and export the results to a new VCF file. The F_1 SuperMASSA model was run with parameter naive_porterior_reporting_threshold set to zero to not filter any genotype. The updog F_1 model was used in parallel using the function multidog through the Reads2MapTools wrapper function updog_genotype which outputs the results in a new VCF file. In the testing of scenarios in which we considered multiallelic markers, the VCF containing them are merged into the VCF files from polyRAD, SuperMASSA, and updog. The merged VCF is the input for linkage map building in OneMap version 3.0.

The software GUSMap performs the genotype calling and linkage map building with a single model. We used VCFtoRA function to convert the outputted VCF files from GATK and freebayes approaches into GUSMap format. A pedigree of the population and a list of filters (MAF = 0.05, MISS=0.25, BIN=0, DETPH=0 and PVALUE=0.05) was provided to the readRA function. The function makeFS was used to create the full-sib population information. Functions infer_OPGP_FS and rf_est_FS were used to estimate the phase and recombination fraction giving the genomic order of the markers. In some situations, function rf_est_FS outputs infinite values of the recombination fraction. In these situations, our pipeline removes the respective marker and runs the function again. This workaround code increased the time required to run GUSMap.

Linkage maps

Once imported to OneMap, markers were filtered again by maximum missing data of 25%. Because the VCF files include unexpected genotypes according to the loci segregation (e.g. in a cross “AA x AB”, genotype “BB” cannot exist), OneMap makes these genotype calls missing. We also filtered markers with segregation distortion under a global significance level of 0.05 with Bonferroni correction and removed redundant markers. Markers were ordered according to the reference genome position. The genetic distances were estimated by the parallelized HMM multipoint [17, 38] approach using as emission probability a global error rate of 10^{-5} (default in OneMap version < 3.0, here referred to as “freebayes/GATK (0.001%)”), a global error rate of 0.05, and the genotypes probabilities estimated by each genotype caller.

In SimulatedReads2Map, the Haldane map function was used; in EmpiricalReads2Map, we used Kosambi’s map function. To test the influence of the presence of the multiallelic markers in the ordering procedure, we used the built map for the chromosome 10 linkage group of aspen and ordered its markers using MDSMap [55] (wrapper function implemented in OneMap 3.0) and order_seq ordering algorithms with and without multiallelic markers.

Performance comparison

We conducted performance comparisons for each combination of SNP caller, genotype caller, and source of read counts, after which they were filtered by sequencing quality, MAF, segregation distortion, redundancy, and missing data. Outlier markers breaking the pattern of the recombination fraction matrix were removed only for the ordering test with and without haplotype-based multiallelic markers in the empirical data set. We evaluated the estimated progeny genotype concordance by comparing the agreement between real and estimated heterozygous, reference allele homozygous (homozygous-ref), and alternative allele homozygous (homozygous-alt) states. For that, we count the number of genotypes estimated as one type given that the true type was another, i.e., Est: homozygous | True: heterozygous. The methods are the combination of each SNP caller, genotype caller, and read count source. We expected that a good method would result in high probabilities for the same estimated and real genotypes (i.e. Est: homozygous | True: homozygous) and low probabilities when they are different (i.e. Est: homozygous | True: heterozygous). These were summarized using receiver operating characteristic (ROC) curves by plotting the *sensitivity* ($\frac{\textit{true positives}}{\textit{true positives+false negatives}}$) in the vertical axis versus 1 − *specificity* ($\frac{\textit{false positives}}{\textit{false positives+true negatives}}$) on the horizontal axis for all possible thresholds in a logistic regression [56].

To test the capabilities of software correctly estimating the parental genotypes, we used the same conditional frequency, but instead of measuring the similarities between individuals' genotypes, we tested the combination of both parental genotypes. To do that, we calculate the conditional frequency analysis between the marker types (e.g. Est=B3.7 | True=B3.7). Based on Mollinari et al. [57], we compared the centiMorgan distances of markers in the maps estimated by each method and the real map using the Euclidean distance (D):

$$D = [(m-1)^{-1}(\hat{\mathbf{d}}-\mathbf{d})'(\hat{\mathbf{d}}-\mathbf{d})]^{1/2}$$

where m is the number of markers evaluated, $\hat{\mathbf{d}}$ is the vector of estimated distances,$\mathbf{d}$ is the vector of real distances, and $'$ indicates vector transposition. A value of $D = 1$ means that the estimated map differs by an average of 1 cM from the built map regarding all genomic positions. We also evaluated the orders provided by the different ordering algorithms by computing the absolute value of Spearman's rank correlation between orders.

Read2Map Workflows App

The shiny app Reads2MapApp was built to display results from the workflow analysis. It includes graphics and statistics about SNP calling efficiency, the number of markers discarded by filtering steps, marker types, computer resources and time spent by each step of the workflow, allele depth by genotype, genotype probabilities, ROC curves, map size, map phases, recombination fraction matrix, progeny haplotypes, breakpoints count, and the correlation between linkage map and reference genome markers positions. Reads2MapApp is a modularized R package using the golem framework [58] that can be rendered and displayed locally or on a server. It can be installed from its GitHub repository and run with a single command (run_app). Once uploaded the Reads2Map output file in the upload section of the app, all graphics will be automatically generated.

Results and Discussion

RADinitio reads simulations

Allelic bias has been observed frequently in GBS data [10, 9]. The primary source of bias in GBS data is related to the PCR amplification step during library preparation [8, 9]. Duplicates can be generated from the library preparation using the PCR or from erroneous detection of a single amplification cluster as if multiplied by the optical sensor of the sequencing instrument [59]. For Whole Genome Sequence (WGS) and exome sequencing data, it is recommended that duplicated sequences are filtered out because of their redundant information and the bias that they can bring to the statistical analysis. In this context, we expect that most of the sequences have partial overlap. Therefore, it is possible to identify the duplicates as the ones that completely overlap with each other and have a lower quality score of the sequence base. But, with GBS data, duplicated sequences are expected to be common because all sequences have the same starting point: the restriction enzyme cut site. Filtering duplicates, in this case, would reduce the read depth per loci to only one read per allele and increase the uncertainties of genotype estimation in the presence of sequencing errors [60]. Duplicates in GBS present advantages to sequencing depth. However, they also bring more allelic bias and erroneous nucleotide substitutions from PCR.

With the `Reads2Map` workflows, we simulated the read sequences by testing several values of `RADinitio` parameters to try to be as similar as possible to the empirical data and real scenarios. We found that with low mean depths (5) and any of the number of PCR cycles tested (5, 9, and 14), almost all markers identified by `GATK` are filtered out in the segregation distortion test, and maps cannot be built. Setting the mean depth to 10 and a high number of PCR cycles (9 and 14) also kept a few markers in the `GATK` analysis. Therefore, we performed all the simulated scenarios using 5 PCR cycles with mean depths of 10 and 20.

The mean percentage of duplicated reads in the aspen empirical data set was 76% (SE 0.55%), while in the simulated data set with mean depths of 10 and 20 were, respectively, 88% (SE 0.00%) and 92% (SE 0.00%), according to the `Picard MarkDuplicates` tool [61] results. It shows that `RADinitio` simulates more duplicates per cycle than expected by the set proportion of 4:1 in the input parameters. Even with a lower number of PCR cycles (5), the simulated data presents more PCR duplicates than the empirical PCR performed to generate the aspen data set, which had 14 cycles [39]. The excessive number of PCR duplicates in the simulations may be why `GATK` identified a few false positives markers with a mean number of 0.49 for depth 10 and 0.48 for depth 20 (Figure 2).

Another difference between the simulated and empirical data set is the number of markers identified by `freebayes` and `GATK`. If the only filters applied to the identified markers are maximum missing data of 25% and MAF of 5%, `freebayes` identified 4.30x and 5.45x more markers than `GATK` in the rose and aspen data sets, respectively. This same proportion is not observed in the simulated data sets, in which `GATK` identifies a mean number of markers of 172.27 (SD 8.12) in depth 10 and 175.80 (SD 6.50) in depth 20, and `freebayes` identify a mean number of 160.39 (SD 2.10) in depth 10 and 157.33 (SD 2.47) in depth 20 (Figure 2). This shows that the simulations are biased towards `GATK` because its markers were used as references for the simulations.

In the simulated data, markers were close to the restriction enzyme cut sites identified in *P. tremula* empirical data. However, the simulations consider that the efficiency of the enzyme can vary across libraries which may explain the high number of false negatives (about 77% of the simulated data). Measuring the common markers across the simulated families, we observed a higher overlap of marker positions when estimated by `freebayes` than `GATK` (Figure 2).

Once the markers are identified, the genotypes can be estimated according to the read count at each locus. Ideally, in a diploid individual, the homozygous would receive the same allele from both

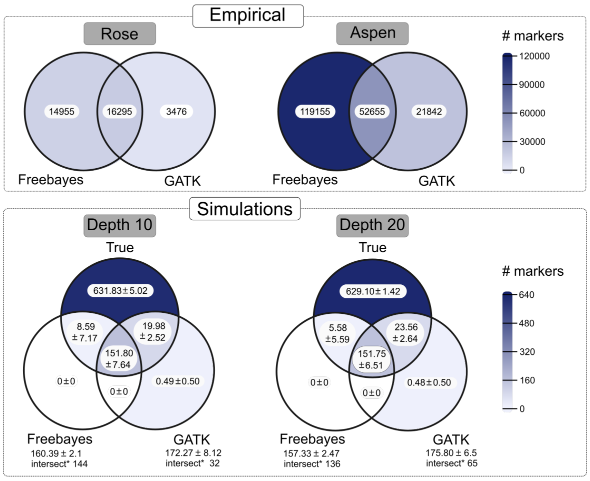

Figure 2. Venn diagrams show the number of markers identified by `freebayes`, `GATK`, and simulated (true). The intersection between the data sets represents markers with the same position in the reference genome *Populus trichocarpa* version 3.0. The Empirical data sets include markers spread across the entire reference genome. The simulations only include markers in the first 8.426 Mb of chromosome 10 (2.1% of the genome). The mean and standard deviation of number markers are shown for the simulated data set once the simulation and SNP calling are repeated 60 times. Markers were filtered by 25% maximum missing data and MAF 5% in empirical and simulated data. * Number of markers common to all 60 repetitions.

parents. The heterozygous would have half of the reads containing one allele and half a different one. However, we can observe the deviation of this ideal scenario in GBS empirical data (Supplementary Figures 5-6).

The `RADinitio` simulation results in alleles read counts distribution (Supplementary Figures 7-10) were similar to the observed in the progeny of the empirical data in terms of dispersion and allelic bias [9]. However, it could not simulate the low-depth counts for parents nor the outlier allele depth presented in the empirical data set. Thus, our simulations were not able to cover these two characteristics that can be found in empirical data sets.

In general, the evaluations of `RADinitio` simulations profile shows that we can expect fewer markers and genotyping errors in the simulated compared to the empirical data. A smaller number of markers should not reduce the built linkage map quality because the analysis was made in F_1 populations, which have large disequilibrium blocks. However, the smaller number of genotyping errors overestimates the SNP and genotype calling software efficiency. This overestimation is commonly observed in simulation results once the data cannot capture all biases and errors in the empirical data. If the software has low efficiency in simulated data, it will probably underperform with empirical data. Thus, the simulations can be used to understand specific software limitations but not ultimately define the best performance [62].

With simulated data results, it is possible to identify the source of the errors causing the low efficiency and elaborate methods to overcome them because simulated data provide a clear comparison between simulated (true) and estimated data. Therefore, the simulations were useful to optimize filters applied to identified markers and genotypes to obtain good quality linkage maps with simulated maps and improved maps with empirical data. We also used the simulations to measure the effects of segregation distortion in the linkage maps and to validate all code developed for the analysis.

Genotype calling efficiency

With the simulations, we could measure the number of wrongly estimated genotypes and the reliability of genotype probability provided by each software (Supplementary figure 7-12). We observed three types of errors: when the genotype is estimated as homozygous, but it is actually heterozygous (Est: homozygous | True: heterozygous); when the estimated genotype is heterozygous and the true genotype is homozygous (Est: heterozygous |True: homozygous); when the estimated genotype is alternative homozygous, and the true genotype is the reference or vice-versa (Est: homozygous-alt/ref | True: homozygous-ref/alt). The latter is only observed in genotypes estimated by `polyRAD`, `SuperMASSA`, and `updog` using `GATK` output VCF read counts (AD format) and had a maximum frequency of 0.74% of the genotypes in `SuperMASSA` estimations in simulations with mean depth 20. We observed that in these situations, the genotype is considered missing in the `GATK` output VCF GT format field, but it always reports the total read depth in the reference allele field of the AD format field (e.g. Estimated = GT:AD ./.;22,0 | True = GT:AD 1/1;0,22). This same issue can also cause errors of type Est: homozygous | True: heterozygous (Figure 3 and progeny genotypes in Figure 4) in `polyRAD`, `updog` and `SuperMASSA` genotypes generating an allele dropout scenario.

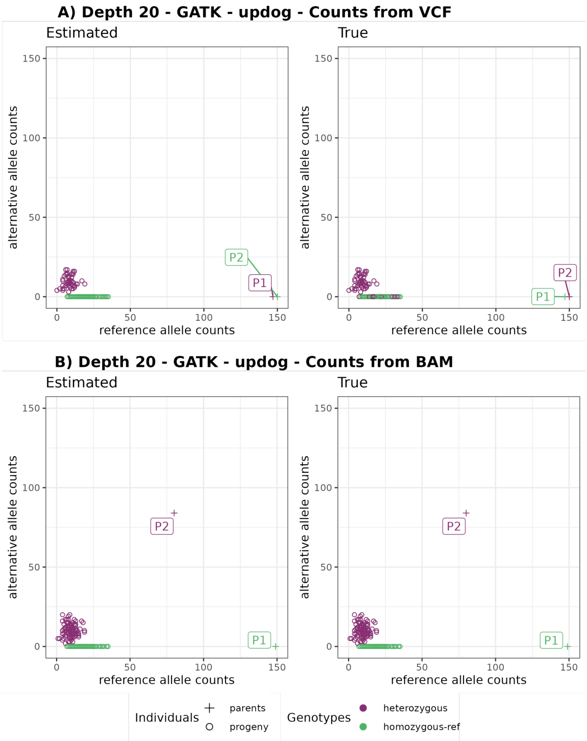

Figure 3. Example of error (Est: homozygous | True: heterozygous and Est: heterozygous | True: homozygous) in parental genotypes leading to a wrong marker type (Est: D1.10 | True: D2.15). Estimated reference (x-axis) and alternative (y-axis) allele count. Graphics on the left have colors according to estimated genotypes, and on the right to the true genotypes. A) show counts from `GATK` VCF file and B) from BAM file. In the VCF file outputted by `GATK` the P1 genotype is missing (GT ./.) because the reads did not pass the quality filters, but it reports the counts in the reference AD field (149,0). The `updog` software use progeny segregation (1:1) to estimate the parents, but it makes a mistake identifying which one is heterozygous. Using counts from BAM file (B) fix this issue despite losing the `GATK` quality filters that can be important in other situations.

Using the allele counts from the BAM alignment file, as suggested by [53], removes these types of errors in `polyRAD`, `SuperMASSA`, and `updog` genotype estimations with `GATK` markers. In contrast, by using the BAM counts, we lose the advantage of the robust filtering applied by `GATK` pipeline to maintain only the good quality read counts in its VCF allele depth field. To keep the `GATK` allele depth accurate but still overcome the common error observed when the genotype is missing, we replaced the VCF allele count (AD and DP fields) with zero when the genotype information is missing before using it for `polyRAD`, `SuperMASSA` and `updog` genotyping. In

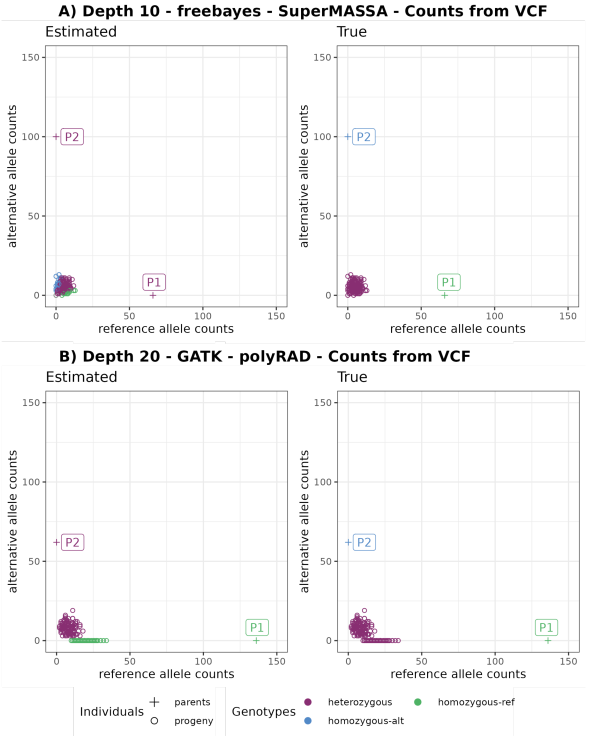

Figure 4. Example of error (Est: homozygous | True: heterozygous) in progeny genotypes leading to wrong marker types in A) Est: B3.7 | True: non-informative and in B) Est: D1.10 | True: non-informative. Graphics on the left have colors according to estimated genotypes, and on the right to the true genotypes.

empirical data, allele dropout can happen for other reasons, such as polymorphisms in the cut site or non-amplification of one of the alleles in the PCR step [9]. This requires another strategy to avoid wrong estimations.

For genotypes called by `polyRAD` and `updog`, the error (Est: homozygous | True: heterozygous) is more frequent than the error (Est: heterozygous | True: homozygous) in simulations with a mean depth of 10. The opposite is observed in some scenarios of the simulations with a mean depth of 20. This difference between simulations with mean depths 10 and 20 shows that `updog` and `polyRAD` are more susceptible to wrongly estimating homozygous genotypes in the presence of sequencing errors found more frequently at higher depths. All incorrectly called genotypes presented high differences in allele counts (e.g., 1 alternative allele: 23 reference alleles).

The scenarios with a higher number of correct genotypes were those called by `freebayes` and `GATK`, or by `updog` and `polyRAD` using markers from `freebayes` SNP calling, counts from VCF, and simulation mean of 20. The segregation distortion does not affect the frequency of correct genotypes in most scenarios (Supplementary figures 7-10), despite affecting the reliability of the genotype probabilities provided by `polyRAD` (Supplementary figures 11-12).

Marker types

Combining information from both parental genotypes defines the expected Mendelian segregation for each locus. The informative combinations for outcrossing species with biallelic codominant markers must have at least one heterozygous genotype in one of the parents, including the marker types B3.7, D1.10, and D2.15 (Supplementary figures 13-16). The haplotype-based multiallelic codominant markers can also present types A.1, A.2, D1.9, and D2.14. `OneMap` 3.0 does not consider the parental genotype probabilities in its HMM multi-point approach. Thus, it is important to plan the sequencing experiment with high-quality parental genotypes because, if there are errors, they will not be corrected in downstream processing, and it will cause distortions in the resulting distances and haplotypes. To avoid map size inflation, erroneous parental genotypes must be removed before the linkage map analysis.

Filtering the data set by segregation distortion is an efficient way of removing markers with wrong parental genotypes. The software `updog`, `polyRAD`, and `SuperMASSA` models consider the segregation pattern of the population to infer the genotypes, and, in some cases, they change the parental genotypes to fit in the observed population segregation pattern. If the progeny genotypes have low quality, it can lead to an erroneous estimation of the parental genotypes. We observed some cases in which non-informative markers are estimated as informative because of genotyping errors in progeny genotypes (Figure 4). In other cases, when alleles dropout in the heterozygous parent of a marker segregating 1:1, the models identify that one of the parents should be heterozygous, but the predictive models make mistakes in identifying which of them should be heterozygous (Figure 3).

We tested three other filters to overcome this in `updog`, `polyRAD`, and `SuperMASSA`. One of them was filtering the genotypes by the genotype probability. If the progeny genotype has a genotype probability lower than 0.8, the genotype is considered missing data. The marker is discarded if the frequency of missing data across all progeny is higher than 25%. The other filter tested was removing non-informative markers from the VCF file coming from `GATK` and `freebayes` before using it as input for `updog`, `polyRAD` and `SuperMASSA`. We considered non-informative markers homozygous in both parents or if at least one of the parental genotypes was missing. The third filter was to replace the allele depth (AD) field in the VCF file format by missing data when the genotype is missing. This avoids that `updog`, `polyRAD`, and `SuperMASSA` use the allele depth when `GATK` filtered out the genotype due to bad quality.

Removing the non-informative markers before the genotype calling by `updog`, `polyRAD`, and `SuperMASSA` reduced the number of wrongly identified marker types by that software, mainly in the simulated scenarios with a mean depth of 20 (Figure 5 and Supplementary figure 17).

We expect all multiallelic markers identified by `freebayes` to come from combinations of biallelic marker types (Figure 6 and Supplementary figure 18). The simulations showed the amount of B3.7, D1.10, D2.15, and non-informative markers converted to A.1, A.2, D1.9, and D2.14 markers. The D1.9 and D2.14 were converted from D1.10 and D2.15 SNP combinations, respectively. Also, the haplotyping approach could combine a few non-informative into A.1, D1.9, and D2.14 markers.

Relation between map size and correct haplotypes

Before using the map size as a metric for map quality, we checked if a map with the expected size always means good quality. A map can have the expected size but poor quality if the number of overestimated and underestimated recombination breakpoints in the progeny haplotypes is the same; in other words, if they cancel out. To test if this happens in our simulated data set, we compared the Euclidean relation of estimated and true genetic distances with the total number of wrong (overestimated + underestimated) recombination breakpoints in the progeny haplotypes (Figure 7 and Supplementary figures 19 and 20). For identifying a break as overestimated or underestimated, we do not consider the expected break position but the total breaks expected for the evaluated haplotype. For example, if one haplotype for a specific progeny was simulated with one break and estimated with zero, then we count it as one underestimated break.

The comparison shows that overestimated breakpoints are generally more frequent than underestimated ones. We observe that when a map is inflated, it also has many wrong recombination breakpoints. However, in some cases, the map has the expected

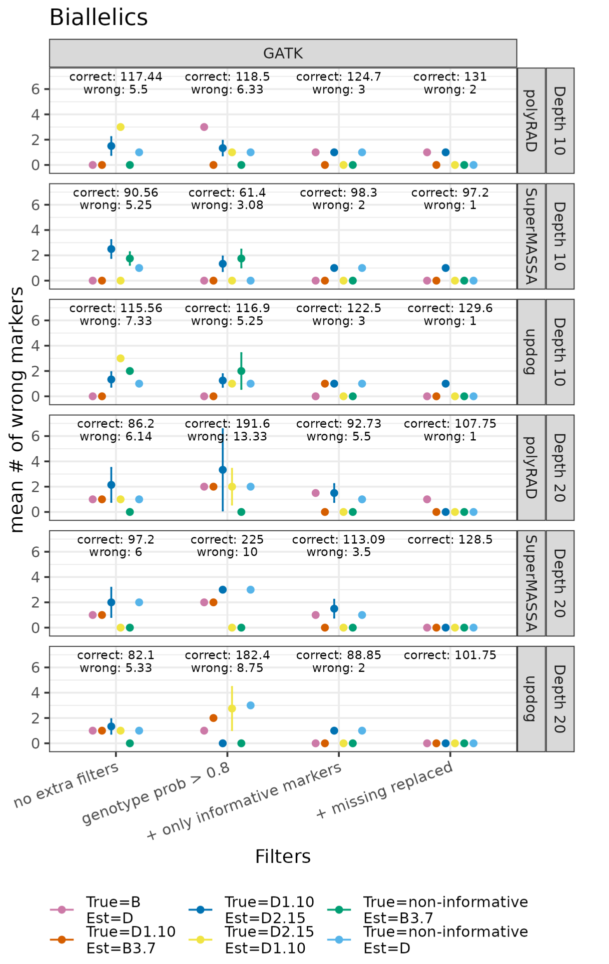

Figure 5. Mean number of wrongly identified biallelic markers in the simulated data set (y-axis) while applying filters by minimum genotypes probability of 0.8, by informativity and replacing AD and GQ VCF field by missing data when GT is missing (x-axis). The numbers on the top of each graphic show the mean total number of correct and wrong markers across the five repetitions. The markers presented here were obtained with `GATK` as SNP and `updog`, `polyRAD`, and `SuperMASSA` genotype calling, with mean depths 10 and 20, with segregation distortion, with allele depth count from VCF. The notation of marker types follows table 2 notation.

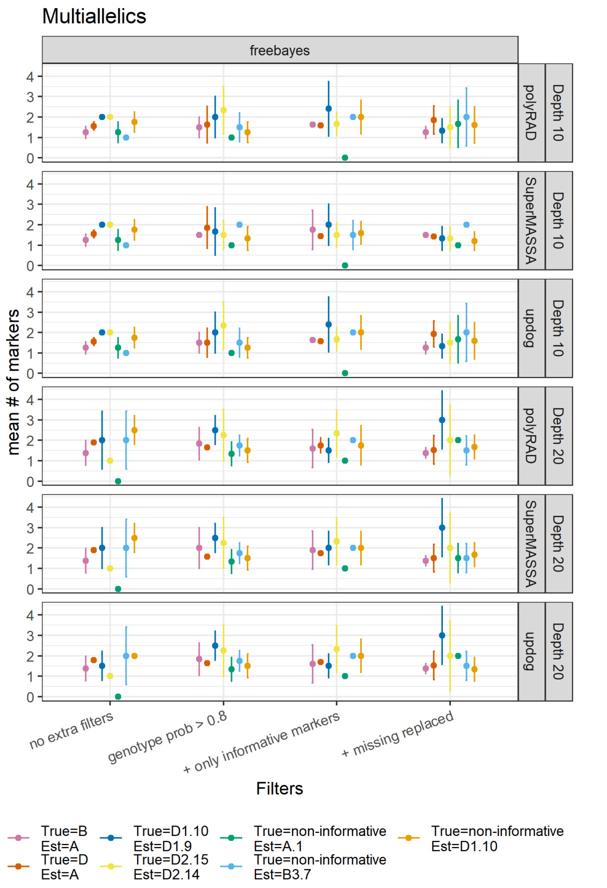

Figure 6. Mean number of multiallelic markers converted from biallelics (y-axis) and how many of them are kept after applying filters by minimum genotypes probability of 0.8, by informativity and replacing AD and GQ VCF field by missing data when GT is missing (y-axis). The markers presented here were obtained using simulated data, `freebayes` as SNP and `updog`, `polyRAD` and `SuperMASSA` genotype calling, with mean depths 10 and 20, with and without segregation distortion, with allele depth count from VCF. The notation of marker types follows table 2 notation.

map size, but a high number of wrong haplotypes due to both overestimated and underestimated breaks. A high number of underestimated breaks can be observed in situations where the Euclidean distance is close to, or less than 1 ($log_{10}0$) and the number of wrong recombination events is between 10 and 100 ($log_{10}1$–$log_{10}2$). These situations are more frequent when a global error rate of 5% is used.

Effects of contaminant samples

In the empirical data results, we observed maps with expected size and excess recombination breakpoints in just a few individuals in the progeny. This variation can be related to contaminant samples. The study of Zhigunov et al. [39] identified six contaminants in the aspen data set. When we ran the workflows, including the contaminant samples, the maps built with `freebayes` markers and `updog`, `SuperMASSA`, and `polyRAD` were smaller in size than without the contaminant. This would (wrongly) suggest better quality if map size is the only metric used (Figure 8A and Supplementary figure 21A). Nevertheless, the maps presented higher differences in the number of recombination breakpoints among individuals when using the genotype probabilities relative to each genotype call software (Figure 8B and Supplementary figure 21B). Some contaminant samples presented more recombination events than the rest of the progeny. Using 5% of global error reduces this difference and can mask the presence of contamination (Figures 9).

Effects of filters

Another important characteristic to consider in a good-quality map is the number of markers. The same data set will vary according to the SNP and genotype call software and filters used. We filtered all data sets by maximum missing data of 25%, segregation distortion, and redundancy. We tested the effects of three extra filters based on common errors observed in the simulated data set genotyping evaluations (Figures 3 and 4): minimum genotype probability of 0.8; removal of non-informative markers; replacing AD and GQ with missing data when GT is considered missing in the VCF file (Figure 10 and 11). These filters are applied before the segregation test filter, which reduces the number of tests and increases the permissibility of the threshold corrected by multiple tests (Bonferroni correction). Thus, the built map can have more markers in some scenarios even if more filters are applied.

Maps built with genotypes from `GATK` and a global error of 5% were smaller when filtering by a minimum genotype probability of 0.8 in higher depths of empirical and simulated data (Supplementary figures 22 and 23). The most significant effect of the filters can be observed in maps built with `updog`, `SuperMASSA` and `polyRAD` genotypes and genotypes probability (Figures 11 and 10). In both empirical and simulated data sets, higher-depth scenarios generate

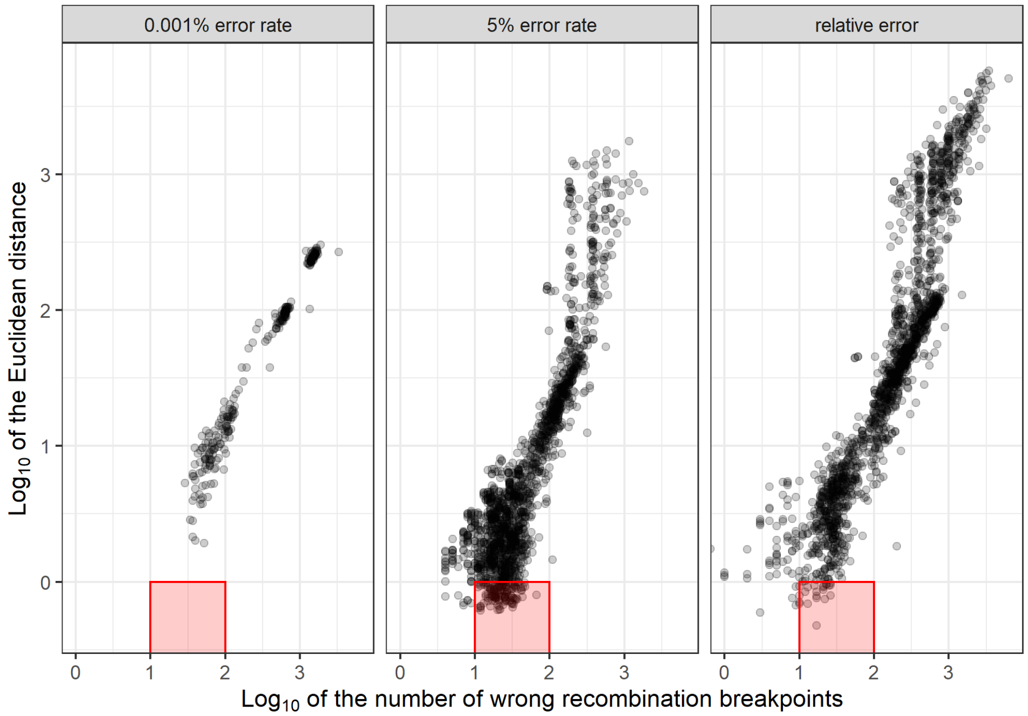

Figure 7. Relation between Euclidean distance (y-axis) and the number of recombination breakpoints (x-axis) in maps built with global error rates (0.001% and 5%), and with probabilities outputted by the genotype call software (relative error). Each dot represents a map built with simulated data based on the first 37% of aspen chromosome 10. The red squares highlight maps that do not present inflated size (1 or less Euclidean distance) but have from 10 to 100 wrong recombination breakpoints.

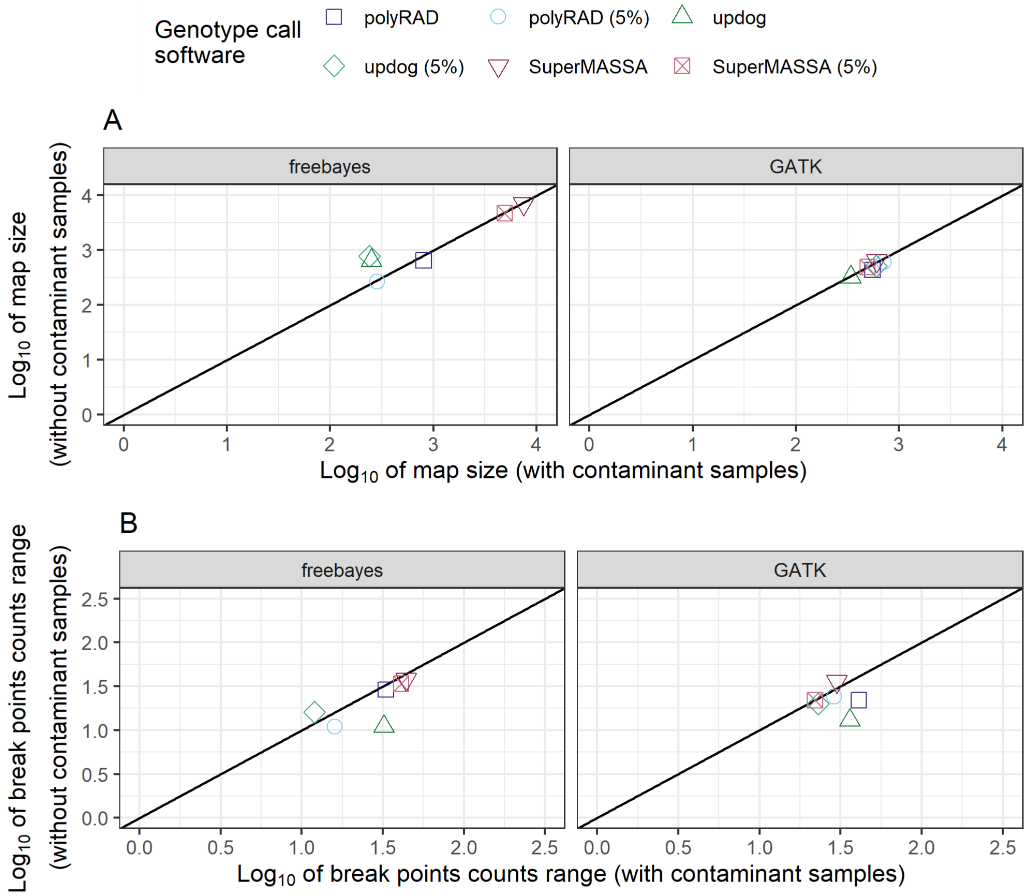

Figure 8. Effect of contaminant samples in the map size (A) and the number of estimated recombination breakpoints range among aspen empirical data set progeny individuals (B). The data sets presented in this figure contain multiallelic markers, the allele counts from the VCF file, and were filtered by genotype probability higher than 0.8 and contain only informative markers.

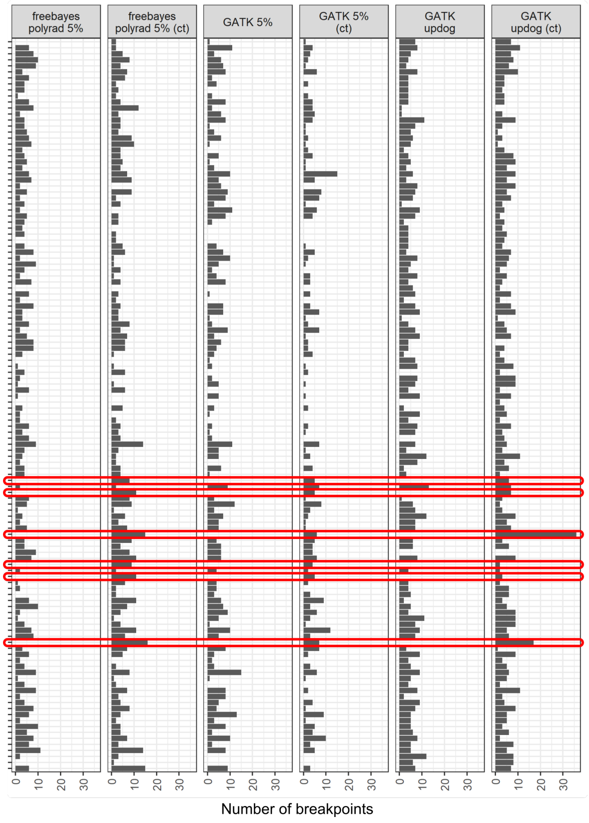

Figure 9. The total number of recombination breakpoints (x-axis) estimated for each progeny individual (y-axis) of the aspen full-sib population with and without contaminant samples (cont) with selected pipelines. The red ellipses indicate the contaminant samples.

linkage maps with sizes closer to the expected after the extra filters are applied.

Effects of segregation distortion

The segregation distortion in the data does not affect the number of wrong estimated genotypes by the genotype call software (Supplementary figures 7-10), but it can affect the reliability of `updog`, `SuperMASSA`, and `polyRAD` in outputting genotype probabilities in some scenarios (Supplementary figure 11 and 12). Consequently, the map size can be inflated using genotype probabilities from these software (Figure 12 and Supplementary figure 24).

Comparison with `GUSMap`

We compared all maps built with `OneMap` combined with upstream approaches with maps built with the GUSMap [14] software (Figures 13, 14 and Supplementary figures 25 and 26). We could not apply the extra filters to GUSMap genotypes as they are estimated internally in the software. In both simulated and empirical data, the maps generated by GUSMap presented greater map sizes.

Selected pipelines

The differences between simulated and empirical data discussed below also result in differences in the performances of software in these two data set types (Figure 15 and Supplementary figures 27-29). We focused on selecting the best pipelines only for the empirical data. For those, we consider as promising approaches the ones that resulted in linkage maps with a high number of markers, with no or few outlier markers distorting the total map length (Figures 15 and Supplementary figure 27), and with the number of recombination breakpoints identified in each progeny individual closer to what is expected for a 38 cM group according to meiotic properties (Figure 9 and Supplementary figure 30).

The rose data set presents higher sequencing depth; thus, the quality of the genetic map is generally better than the aspen data set. Using the filters by genotype probability and non-informative markers, it was possible to remove the majority of the outliers from the maps built and still keep a high number of markers by using `GATK` markers, `GATK` and `polyRAD` genotypes, and a global error rate of 5%. Despite presenting a higher number of markers, the approach using `freebayes` markers and genotypes with a global error rate of 5% resulted in a map with double the size (Figure 16). The number of recombination breakpoints profiles in these three cases shows that the individual 649-12 is a possible contaminant in this data set (Supplementary figure 30). The contaminant samples tend to have a higher number of breaks, as we saw in the comparison of aspen with and without contaminant samples.

In the aspen data set, the best approach was to build the map with `GATK` markers, `GATK` genotypes and a global error of 5%, or with `updog` genotype probabilities (Figure 17). Similar maps were also built using markers from `freebayes`, genotypes from `polyRAD` and a global error rate of 5%. All the maps built for the aspen data set still presented some outlier markers. Removing these outlier markers requires careful evaluation of diagnostic graphics, such as the heatmaps of the recombination fraction matrix (Supplementary Figures 31 and 32), which is not possible with the workflow's straightforward approach. It makes `Reads2Map` workflows a tool for selecting the SNP and genotyping calling and the genotype probability to build the map, but further revisions to remove the outliers are required to obtain a good quality genetic map.

Haplotype-based multiallelic markers

The previous evaluations show that multiallelic markers do not present a unique effect on the genetic distances (Figures 19 and 18 and Supplementary figures 33 and 34). Depending on the data set quality and combination of software used, it can decrease, increase, or even not affect the linkage map quality under these criteria. We target approaches that can reduce or not affect the genetic map size because the advantage of using multiallelic markers is not in the genetic map distance estimation but in the ordering step of the linkage map building. Algorithms that use two-point recombination fractions estimations to order only biallelic markers have difficulty missing linkage information between markers D1 and D2 (homozygous x heterozygous or vice-versa). These markers can only be related to each other in the presence of more informative markers, such as B3.7 (heterozygous x heterozygous) or the multiallelic. Yet, having few B7.3 markers compared to D1 and D2 can still be an issue for linkage map building. This characteristic was why the first methods for building genetic maps in this type of population resulted in separate maps for each parent [63]. The non-integrated genetic maps limit further QTL analysis of multiallelic traits [64].

The ordering step was not considered in the previous evaluations once the workflows used genomic order to build the maps. To test the effect of multiallelic markers in the ordering, we built a linkage map for the entire chromosome 10 of the aspen data set using markers called by `freebayes`, an error rate of 5%, and two of the `OneMap` order_seq and MDS algorithms to order the markers. The genetic distances were estimated by HMM multipoint approach. Figure 20 B shows the impact of including the multiallelic markers in the two-points-based MDS algorithm [55]. Multiallelic markers slightly increase the Pearson correlation and drastically reduce the Euclidean distance between the estimated ordering and the genomic order. The order_seq algorithm is a strategy developed to apply HMM in the ordering procedure. First, it estimates the order of the markers using a two-point approach (the default is

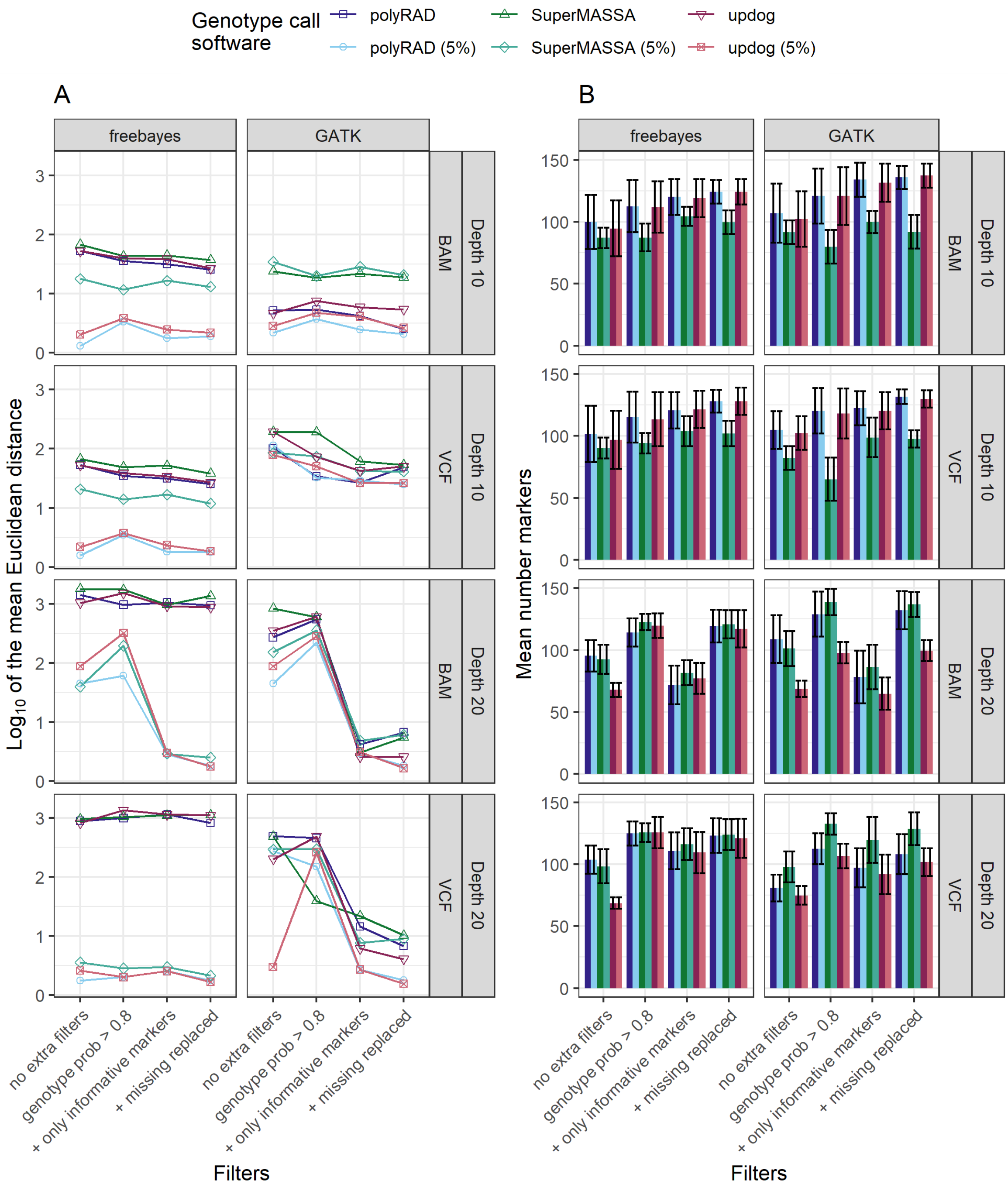

Figure 10. The relation between filters applied (x-axis) and the mean Euclidean genetic distances (A y-axis) and the number of markers (B y-axis) for genotype calling software. The data set shown in the figure contains multiallelic markers and segregation distortion.

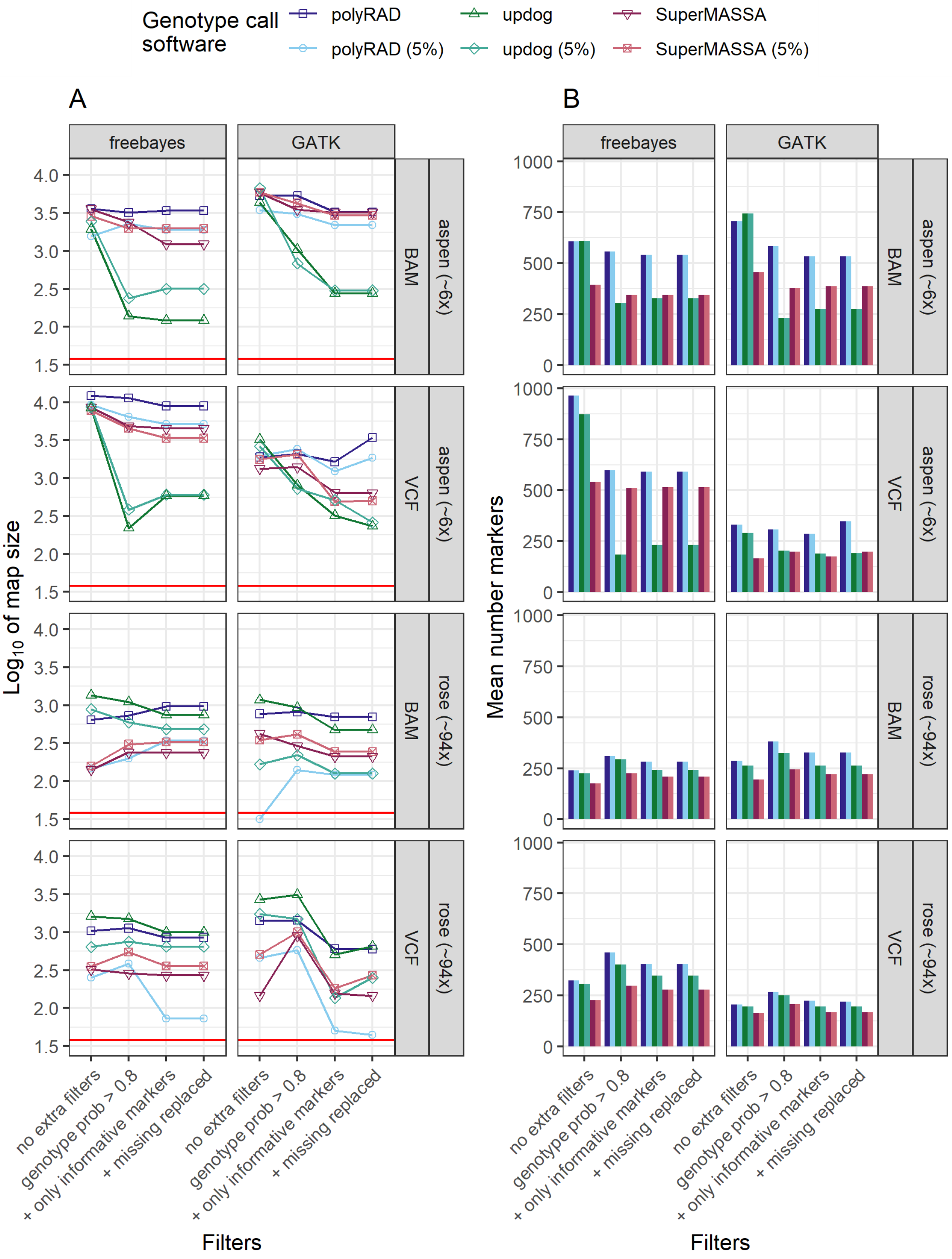

Figure 11. The relation between filters applied (x-axis), the map size (A y-axis), and the number of markers (B y-axis) for genotype calling software used in the empirical data sets. The data sets shown in the figure contain only biallelic markers. The horizontal red line indicates the expected map size (38 cM) for the subset of the genomes used.

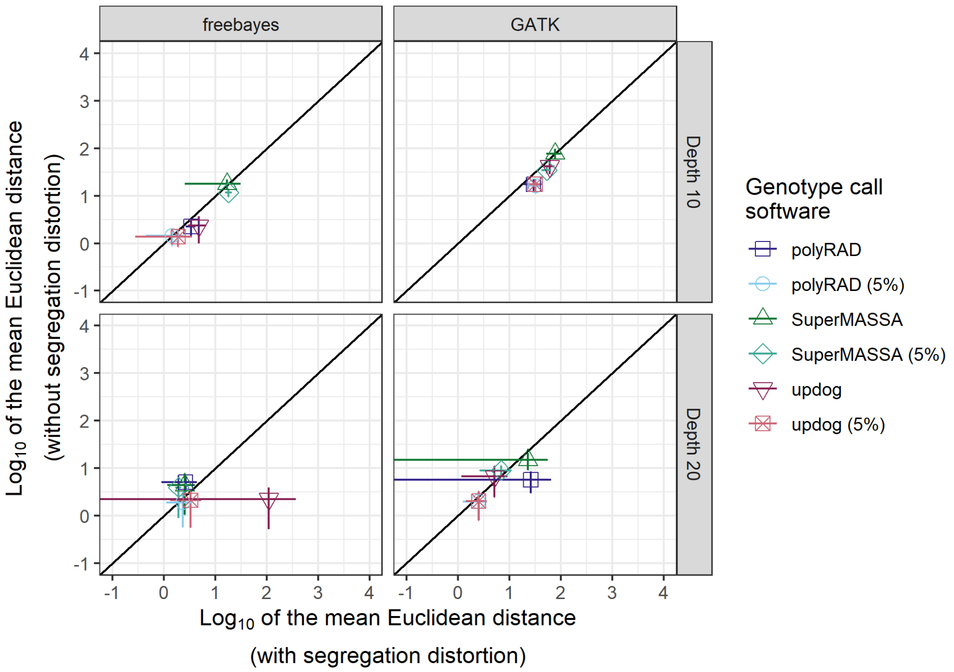

Figure 12. The effect of the simulated segregation distortion in the maps Euclidean distance by genotype calling software. The x-axis shows the mean of the Euclidean distance between estimated and simulated maps built for the data set with simulated segregation distortion, and the y-axis shows with data set simulated without the segregation distortion. The lines crossing the symbols indicate the standard deviation across the five repetitions. The data sets contain only biallelic markers and allele depth count from the VCF file. The markers were filtered by genotype probability higher than 0.8 and only informative markers.

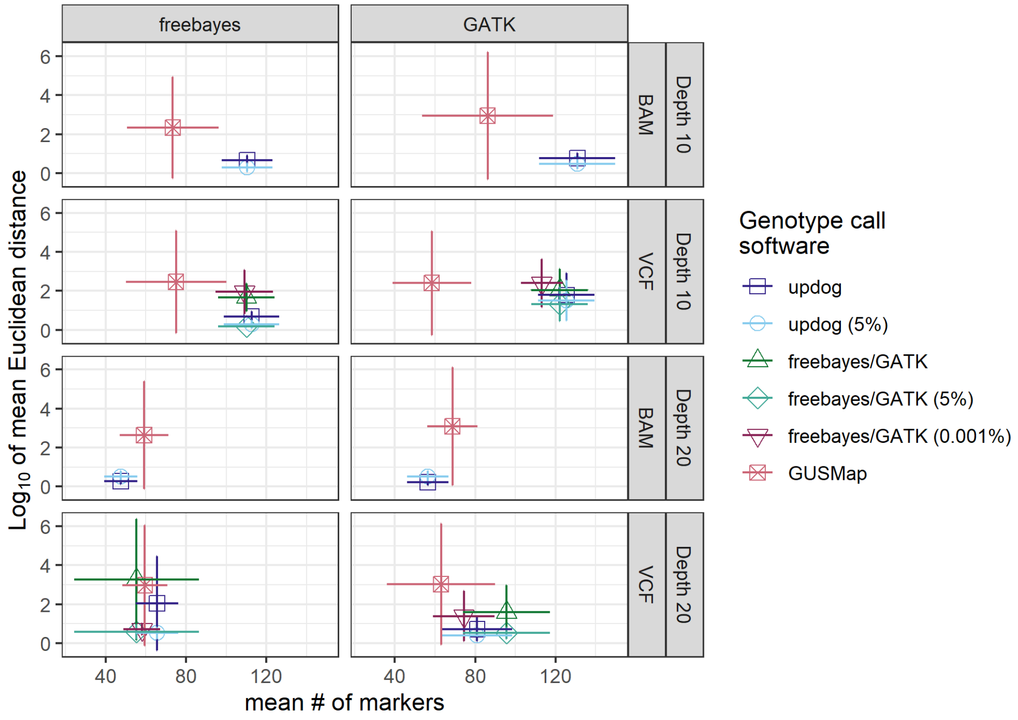

Figure 13. Comparison of Euclidean distance (y-axis) and the number of markers in maps built with `OneMap 3.0` and `GUSMap` for the simulated data. The lines crossing the symbols indicate the standard deviation across the five repetitions. The maps built with `OneMap` are represented by the name of the genotype calling software that provided the genotypes and their probabilities for `OneMap` multipoint approach of genetic distance estimation. The markers inputted in `OneMap` included multiallelic markers filtered by genotype probability higher than 0.8, included only informative markers, and the AD and GQ fields were replaced by missing data when GT is missing.

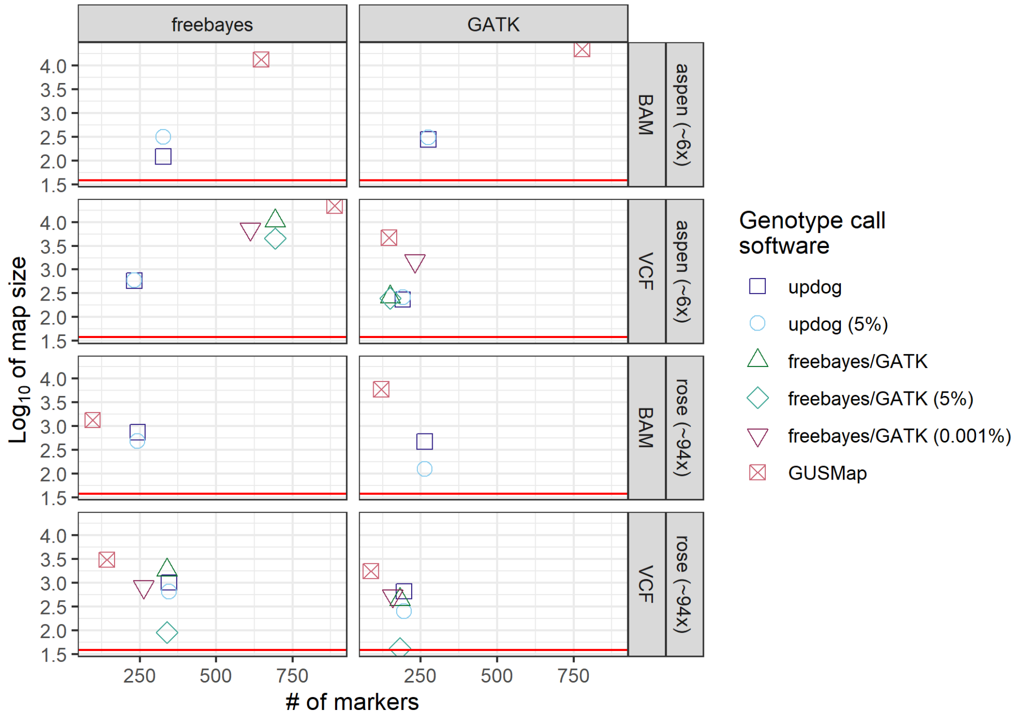

Figure 14. Comparison of map size (y-axis) and the number of markers in maps built with `OneMap` 3.0 and `GUSMap` for the empirical data. The maps built with `OneMap` are represented by the name of the genotype call software that provided the genotypes and their probabilities for `OneMap` multipoint approach of genetic distance estimation. The data sets shown here contain only biallelic. Markers inputted in `OneMap` had a genotype probability higher than 0.8, included only informative markers, and the AD and GQ fields were replaced by missing data when GT was missing. The horizontal red line indicates the expected map size (38 cM) for the subset of the genomes used.

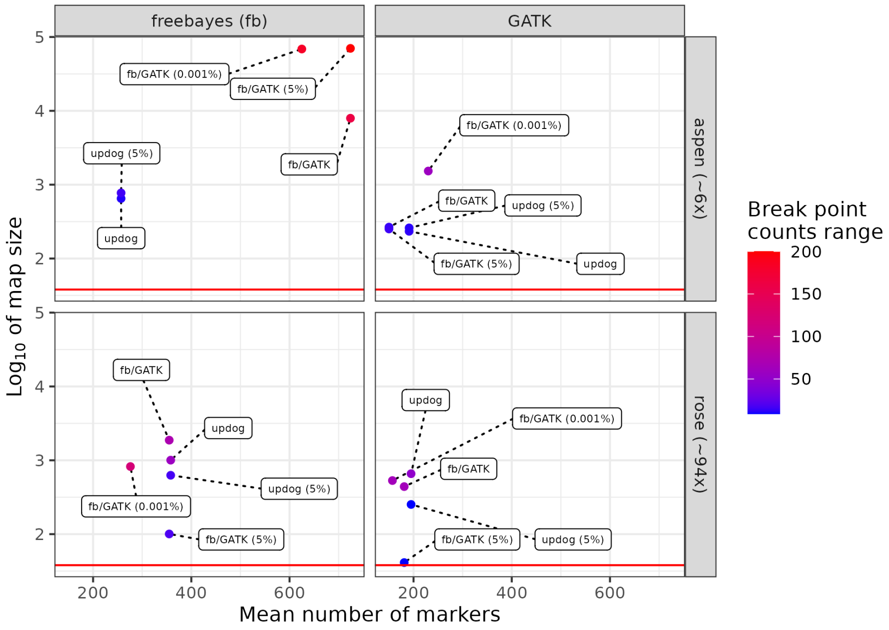

Figure 15. Comparison of map size (y-axis) and the number of markers in maps built with `OneMap` 3.0 using different upstream software for estimating genotypes and genotypes probabilities. Markers inputted in `OneMap` included multiallelic markers filtered by genotype probability higher than 0.8, included only informative markers, and the AD and GQ fields were replaced by missing data when GT is missing. The horizontal red line indicates the expected map size (38 cM) for the subset of the genomes used.

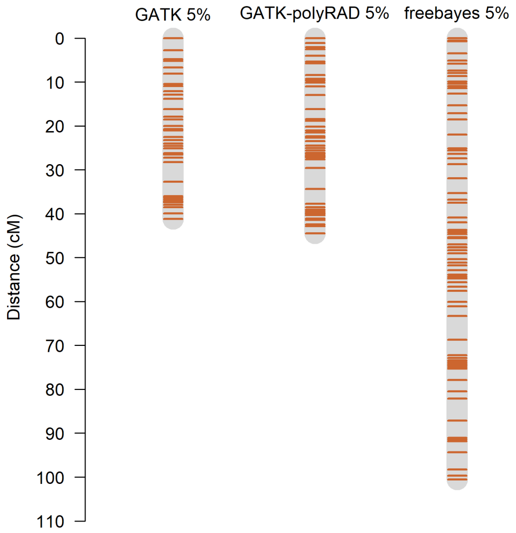

Figure 16. The figure shows the linkage maps built for 37% of rose chromosome 1 (38 cM) with selected pipelines.

the RECORD [65] algorithm). Based on the two-point ordering, a subset (default of five markers) of equally distributed markers is selected and ordered by exhaustive search (compare function). Next, the algorithm adds all the other markers sequentially, testing each possible position using the HMM multi-point approach in the already established sequence. The RECORD algorithm has steps where markers are randomized, which makes the result non-deterministic in the sense that each run can result in a (normally slightly) different order. This strategy used to be very accurate when dealing with a few informative markers (such as SSRs) but is more prone to errors if only biallelic markers are available. Results show that, with haplotype-based multiallelic markers, the strategy returns a high-quality order, reproducing almost entirely the genomic order and the correct pattern of the recombination fraction matrix (Figure 20 A).

Final considerations

The `Reads2Map` workflows have a robust structure to generate production-level results with simple inputs and optimized usage of computational resources. The structure allowed us to test the quality of genetic maps built with the following scenarios: i) using different SNP calling software (`GATK` and `freebayes`); ii) using different genotype calling software (`GATK`, `freebayes`, `updog`, `polyRAD`, `SuperMASSA`); iii) using different linkage map building software (`OneMap` 3.0 and `GUSMap`); iv) establishing different error probabilities (relative to genotype call software, 5%, and 0.001% global error); v) applying different marker filtering; vi) with or without multiallelic markers; vi) in empirical and simulated data; vii) with and without segregation distortion; viii) with and without contaminant samples; ix) with different library preparation; and x) with different sequencing depths. These scenarios are commonly found by researchers trying to produce high-quality linkage maps using sequencing technologies. The `Reads2Map` and `Reads2MapApp` are the first tools to guide best practices for building linkage maps with sequencing data pointing software, parameters and marker filters to be used in diverse scenarios.

We elaborated and limited the scenarios explored according to our experiences as developers of `OneMap`. `OneMap` first version was released in 2007, and since then it has been used to build linkage maps in a diversity of species. Its strategies and structure also served as a base for more complex software such as `MAPpoly` [15] for building linkage maps in polyploid species. With time, new methods for genetic marker identification using sequencing data emerged, changing the context where `OneMap` was used. We included updates in this version 3.0 to resolve issues with inflated genetic maps and marker ordering. Two major changes allow users to read and build genetic maps with the genotype probabilities and haplotype-based multiallelic markers information from the input files (`OneMap` format or VCF file). However, the success of genetic map building will be proportional to the quality of the information provided by upstream procedures such as library preparation, SNP and genotype calling, genotype probabilities estimation, and the combination of SNPs into haplotype-based markers. With `Reads2Map` and `Reads2MapApp`, we provide users tools to select the best approaches before using `OneMap` 3.0 to guarantee that it will result in the best quality genetic map possible with the data available.

For the rose data, the best pipelines filtered the markers using all extra filters (minimum 0.8 of genotype probability, removal of non-informative markers, and replacing AD and GQ field by missing if GT is missing in VCF file), and used the combinations: `GATK` as SNP and genotype calling with a global error of 5%; `GATK` as SNP calling and `polyRAD` as genotype calling with a global error rate of 5%; `freebayes` as SNP and genotype calling with multiallelic markers and a global error rate of 5%. The aspen had a lower sequencing depth. Thus, none of the methods could provide maps with the expected size. Even using the selected methods, further marker filtering was required to obtain a good-quality final map. For the aspen data set, we obtained the best pipelines by also filtering the markers with all extra filters and using the combinations: `GATK` as SNP and genotype calling with a global error rate of 5%; `GATK` as SNP calling and `updog` as genotype calling using `updog` genotype probabilities or `freebayes` as SNP and `polyRAD` as genotype calling using a global error rate of 5%.

Most of the selected pipelines for both empirical data sets used a global error of 5% to estimate the genetic distances because they gave map sizes closer to the expected. We also observed the same results when applying a 5% error rate in the simulated data. With those, we could relate the map size with the number of wrongly estimated haplotypes. The evaluation showed that inflated maps mostly reflect a high number of wrongly estimated haplotypes, but there were some cases where the map was estimated with the expected size but presented a high number of wrong haplotypes, mostly when a 5% global error rate was applied. Using a 5% error rate can also mask the presence of contaminant samples among the progenies. For these reasons, we intend to update `Reads2Map` with genotype calling software that adapt the genotype probabilities for this specific usage and result in map sizes closer to the expected.

The diversity in the pipelines suggested for both empirical data sets highlights that pipelines perform differently with data sets with different properties. We can see this diversity in the effects observed while testing filters, software, and conditions. This means that the pipelines presented here as the best cannot be considered the best for every data set. Thus, users should reproduce all tests presented here using the `Reads2Map` workflows with their empirical data set and select the best pipelines for their specific conditions. The workflows were built using WDL and containers to ensure high reproducibility. This guarantees that different results running different data sets is due to the data set's properties and not to bioinformatic pipeline changes. Also, as the upstream procedures for genotyping and identifying haplotype-based multiallelic markers are improved, updates can be easily made in the workflows.

Every `Reads2Map` workflow run returns a large amount of information. Every step of the workflow, from the reads' alignment to the completed linkage map, provides quality measurements for users to evaluate each scenario. The `Reads2MapApp` shiny app receives all this information compressed in a single workflow output file and converts it into comprehensive interactive graphics. Through the app interface, users can evaluate the performance of

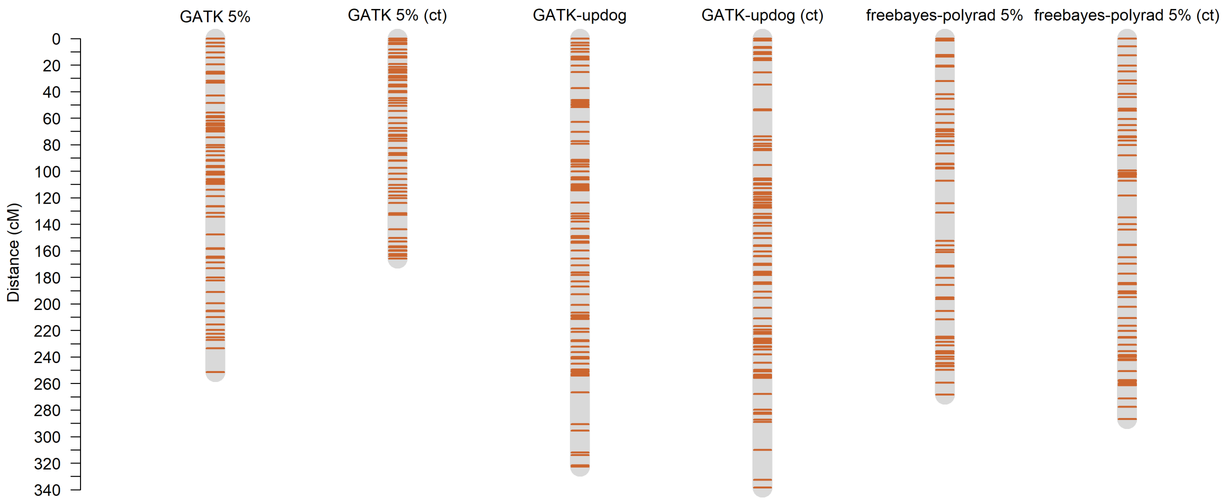

Figure 17. The figure shows the linkage maps built for 37% of aspen chromosome 10 (38 cM) with (ct) and without the presence of 6 contaminant samples and selected pipelines.

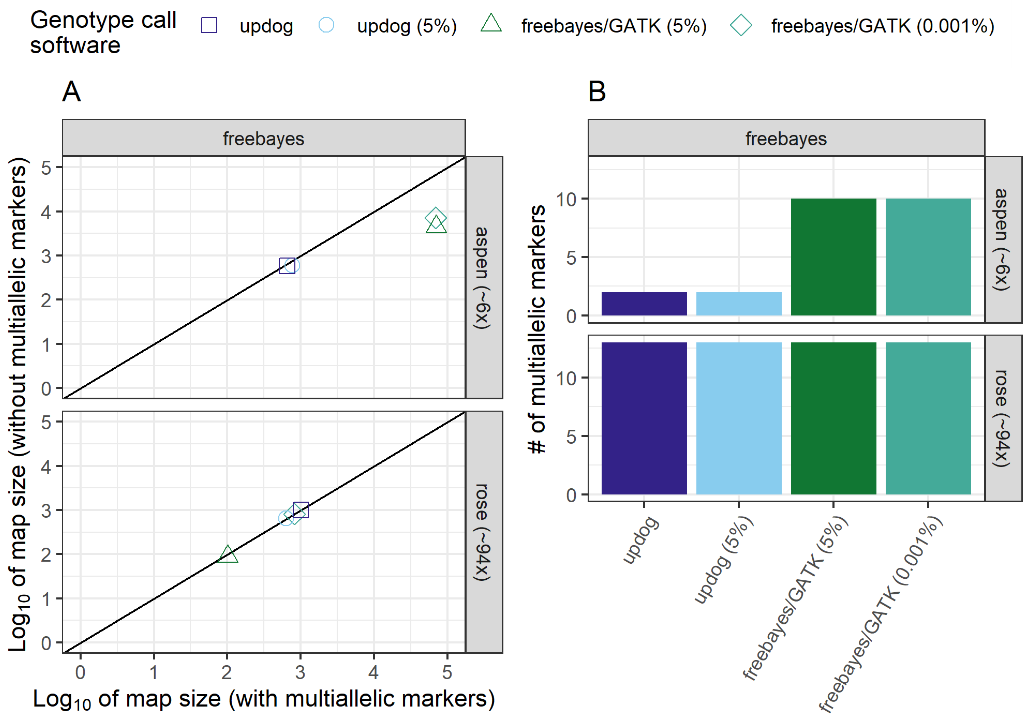

Figure 18. Comparison between empirical data sets with and without multiallelic markers. A: relationship of map size between data set with (x-axis) and without (y-axis) multiallelic markers. B: Number of multiallelic markers present in data sets represented in the x-axis of graphic A. The data sets shown in this figure have allele depth counts from the VCF file, were filtered by a minimum genotype probability of 0.8, included only informative markers and AD and GQ VCF fields were replaced by missing when GT was missing.

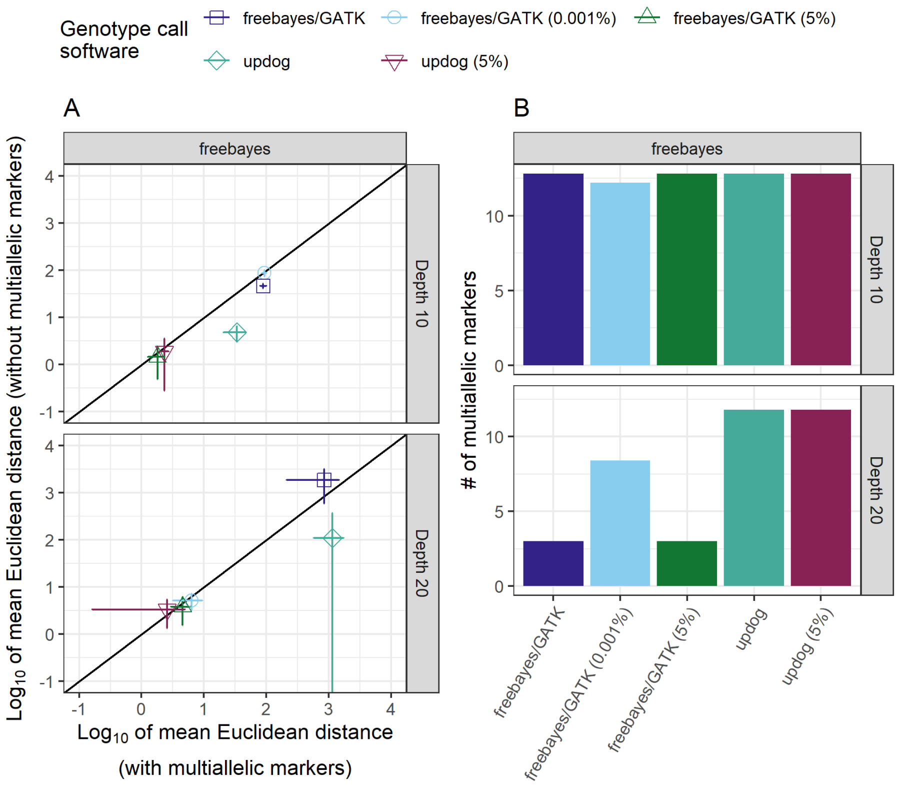

Figure 19. Comparison between simulated data sets with and without multiallelic markers. A: relationship of mean map size between data set with (x-axis) and without (y-axis) multiallelic markers. B: Number of multiallelic markers present in data sets represented in the x-axis of graphic A. The lines crossing the symbols indicate the standard deviation across the five repetitions. The data sets shown in this figure have allele depth counts from the VCF file, segregation distortion, were filtered by a minimum genotype probability of 0.8, and only informative markers.

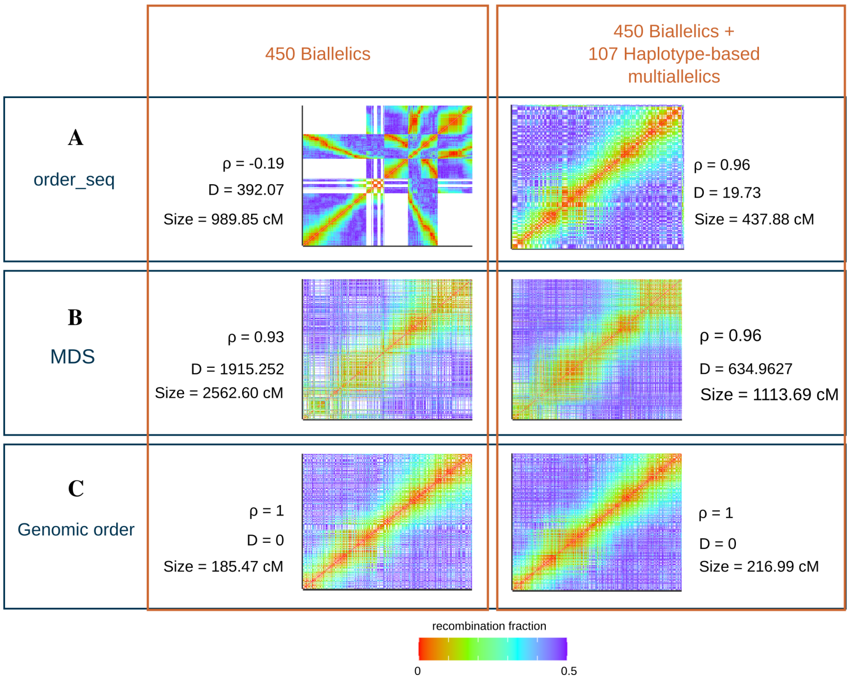

Figure 20. Comparison between ordering algorithms performance in the aspen data set entire linkage group 10 with only biallelic markers, and with biallelic and haplotype-based multiallelic markers. The heatmaps represent the recombination fraction matrix between markers positioned at both axes. In well-ordered linkage groups, we expect a gradient from hot colors in the diagonal (adjacent markers) to cold colors in the upper left and lower right corners. The figure also presents the Spearman rank correlation (ρ) and the Euclidean distances (D) between the estimated map and the map built with markers ordered by the genomic positions. The represented result from order_seq algorithm is only one of the possible results as the procedure is non-deterministic

each combination of software and parameters in each step. If results show issues in any of them, users can re-run the workflow with adapted parameters or include new filters that make sense in their context. Once established the upstream steps based on the app graphics for the built linkage map subset, users can reproduce it for the complete data set, inputting the VCF files from `Reads2Map` into `OneMap`.

Availability of source code and requirements

- Project name: Reads2Map
- Project home page: https://github.com/Cristianetaniguti/Reads2Map
- Main workflows: EmpiricalReads2Map [66] and SimulatedReads2Map [67]
- Operating system(s): Platform independent
- Programming language: WDL and R
- Other requirements: docker or singularity
- License: GNU GPL

Additional files

Supplementary File 1. Emission function for outcrossing.

Supplementary Figure S1. The log_{10} of the CPU time (blue) and the log_{10} of the amount of memory utilized (red) by each task of the Reads2Map workflows when running the simulations with a mean depth of 10. The CPU time is measured with the number of CPUs used times the wall-clock time used.

Supplementary Figure S2. The log_{10} of the CPU time (blue) and the log_{10} of the amount of memory utilized (red) by each task of the Reads2Map workflows when running the simulations with a mean depth of 20. The CPU time is measured with the number of CPUs used times the wall-clock time used.

Supplementary Figure S3. The log_{10} of the CPU time (blue) and the log_{10} of the amount of memory utilized (red) by each task of the Reads2Map workflows when running the aspen empirical data. The CPU time is measured with the number of CPUs used times the wall-clock time used. The filters and linkage map steps were made just with a subset of the data (37% of chromosome 10).

Supplementary Figure S4. The log_{10} of the CPU time (blue) and the log_{10} of the amount of memory utilized (red) by each task of the Reads2Map workflows when running the rose empirical data. The CPU time is measured with the number of CPUs used times the wall-clock time used. The filters and linkage map steps were made just with a subset of the data (37% of chromosome 1).

Supplementary Figure S5. Reference (x-axis) and alternative (y-axis) allele depth distribution for all progeny individuals and a subset of 5% of the markers in rose and aspen data considering the read counts from VCF and from BAM files. Colors represent the estimated genotype by the genotype calling methods. Percentages of each genotype in the entire data set are shown for progeny and parental genotypes in the top right of each graphic.

Supplementary Figure S6. Supplementary figure S5 continued.

Supplementary Figure S7. Reference (x-axis) and alternative (y-axis) allele depth distribution for all progeny individuals and a subset of 25% of the markers from a single simulated family data without segregation distortion, with mean depth of 10 and 20 and considering the read counts from VCF and from BAM files. Colors blue and green show genotypes called correctly by the genotype calling methods, and the colors yellow, orange, and red shows the ones that were called incorrectly. Percentages of correctly and incorrectly genotypes for the entire data set are shown for progeny and also parental genotypes at the top of each graphic.

Supplementary Figure S8. Supplementary Figure S7 continued.

Supplementary Figure S9. Reference (x-axis) and alternative (y-axis) allele depth distribution for all progeny individuals and a subset of 25% of the markers from a single simulated family data with segregation distortion, with mean depth of 10 and 20 and considering the read counts from VCF and from BAM files. Colors blue and green show genotypes called correctly by the genotype calling methods, and the colors yellow, orange, and red shows the ones that were called incorrectly. Percentages of correctly and incorrectly genotypes for the entire data set are shown for progeny and parental genotypes at the top of each graphic.

Supplementary Figure S10. Supplementary Figure S9 continued.

Supplementary Figure S11. ROC curves with the true and estimated genotypes from the five families simulated with mean depth 10 and 20 and the first 8.426 Mb of the chromosome 10 (37% or 38 cM). Here only biallelic markers are considered. The specificity and sensitivity profiles consider different thresholds in the genotype probabilities for each scenario. Higher is the area under the curve, the higher is the genotypes probability reliability. Genotype probabilities thresholds closer to the left superior corner have a higher capacity to differentiate right and wrong genotypes.

Supplementary Figure S12. Supplementary Figure S11 continued.

Supplementary Figure S13. Mean number of corrected identified biallelic by marker types (y-axis) while applying filters by minimum genotypes probability of 0.8, by informativity and replacing AD and GQ VCF field by missing data when GT is missing (x-axis). The markers presented here were obtained using simulated data, `GATK` as SNP and `updog`, `polyRAD`, and `SuperMASSA` genotype calling, with mean depths 10 and 20, with segregation distortion, with allele depth count from VCF. The notation of marker types follows table 2 notation.

Supplementary Figure S14. Supplementary Figure S13 continued. The same information is shown for `freebayes` and `GATK` as genotype call software.

Supplementary Figure S15. The number of markers (y-axis) identified in the first 37% of aspen chromosome 10 while applying filters by minimum genotypes probability of 0.8, by informativity and replacing AD and GQ VCF field by missing data when GT is missing (y-axis). Colors distinguish the marker types according to table 2.

Supplementary Figure S16. The number of markers (y-axis) identified in the first 37% of rose chromosome 1 while applying filters by minimum genotypes probability of 0.8, by informativity and replacing AD and GQ VCF field by missing data when GT is missing (y-axis). Colors distinguish the marker types according to table 2.

Supplementary Figure S17. Mean number of wrongly identified biallelic markers (y-axis) while applying filters by minimum genotypes probability of 0.8, by informativity and replacing AD and GQ VCF field by missing data when GT is missing (x-axis). The numbers on the top of each graphic show the mean total number of correct and wrong markers across the five repetitions. The markers presented here were obtained using simulated data, `GATK` as SNP and genotype calling, with mean depths 10 and 20, segregation distortion, and allele depth count from VCF. The notation of marker types follows table 2 notation.

Supplementary Figure S18. Mean number of multiallelic markers converted from biallelics (y-axis) and how many of them are kept after applying filters by minimum genotypes probability of 0.8, by informativity and replacing AD and GQ VCF field by missing data when GT is missing (y-axis). The markers presented here were obtained using simulated data, `freebayes` as SNP and genotype calling, with mean depths 10 and 20, with and without segregation distortion, with allele depth count from VCF. The notation of marker types follows table 2 notation.

Supplementary Figure S19. The base 10 logarithm of the mean of underestimated and overestimated recombination breakpoints identified in the progeny simulated with a mean depth of 10 and linkage maps built using genotypes and genotypes probabilities

coming from different approaches and filters applied. Colors distinguish the simulations with and without segregation distortion and multiallelic markers and the source of read counts by allele. The blue horizontal line cuts infinite values generated by the logarithmic of zero when there are no wrong breakpoints. The closer the triangles are to the blue line better the method could reproduce the recombination breakpoints number.

Supplementary Figure S20. The base 10 logarithm of the mean of underestimated and overestimated recombination breakpoints identified in the progeny simulated with a mean depth of 20 and linkage maps built using genotypes and genotypes probabilities coming from different approaches and filters applied. Colors distinguish the simulations with and without segregation distortion and multiallelic markers and the source of read counts by allele. The blue horizontal line cuts infinite values generated by the logarithmic of zero when there are no wrong breakpoints. The closer the triangles are to the blue line better the method could reproduce the recombination breakpoints number.

Supplementary Figure S21. Effect of contaminant samples in the map size and in the number of estimated recombination breakpoints range among progeny individuals. The empirical aspen data sets presented in this figure contain multiallelic markers, the allele counts from the VCF file and was filtered by genotype probability higher than 0.8 to keep only informative markers.

Supplementary Figure S22. The relation between filters applied (x-axis) and the mean Euclidean genetic distances (A y-axis) and the number of markers (B y-axis) of the built linkage maps. The simulated data set shown here contains multiallelic markers and segregation distortion.

Supplementary Figure S23. The relation between filters (x-axis) applied and the map size (A y-axis) and the number of markers (B y-axis) of the built linkage maps. The empirical data sets shown here contain multiallelic markers and segregation distortion. The horizontal red line indicates the expected map size (38 cM) for the subset of the genomes used.

Supplementary Figure S24. The effect of the simulated segregation distortion in the maps Euclidean distance. The x-axis shows the Euclidean distance between estimated and simulated maps built for the data sets with simulated segregation distortion. The y-axis shows data sets simulated without the segregation distortion. The lines crossing the symbols indicate the standard deviation across the five repetitions. The data sets contain only biallelic markers and allele depth count from the VCF file; the markers were filtered by genotype probability higher than 0.8 and only informative markers.

Supplementary Figure S25. Comparison of Euclidean distance (y-axis) and the number of markers in maps built with `OneMap` 3.0 and `GUSMap` for the simulated data. The lines crossing the symbols indicate the standard deviation across the five repetitions. The maps built with `OneMap` are represented by the name of the genotype call software that provided the genotypes and their probabilities for `OneMap` multipoint approach of genetic distance estimation. The markers inputted in `OneMap` included multiallelic markers, were filtered by genotype probability higher than 0.8 to keep only informative markers, and the AD and GQ fields were replaced by missing data when GT is missing.

Supplementary Figure S26. Comparison of map size (y-axis) and the number of markers in maps built with `OneMap` 3.0 and `GUSMap` for the empirical data. The maps built with `OneMap` are represented by the name of the genotype call software that provided the genotypes and their probabilities for `OneMap` multipoint approach of genetic distance estimation. The data sets shown here contain only biallelic. Markers inputted in `OneMap` were filtered by genotype probability higher than 0.8 to keep only informative markers. The AD and GQ fields were replaced by missing data when GT was missing. The horizontal red line indicates the expected map size (38 cM) for the subset of the genomes used.

Supplementary Figure S27. Comparison of map size (y-axis) and the number of markers in maps built with `OneMap` 3.0 using empirical data and different upstream software for estimating genotypes and genotypes probabilities. Markers inputted in `OneMap` included multiallelic markers, were filtered by genotype probability higher than 0.8, kept only informative markers, and the AD and GQ fields were replaced by missing data when GT was missing. The horizontal red line indicates the expected map size (38 cM) for the subset of the genomes used.

Supplementary Figure S28. Comparison of Euclidean distance (y-axis) and the number of markers in maps built with `OneMap` 3.0 using simulated data and different upstream software for estimating genotypes and genotypes probabilities. Markers inputted in `OneMap` included multiallelic markers and segregation distortion, were filtered by genotype probability higher than 0.8, kept only informative markers, and the AD and GQ fields were replaced by missing data when GT is missing.

Supplementary Figure S29. Comparison of Euclidean distance (y-axis) and the number of markers in maps built with `OneMap` 3.0 using simulated data and different upstream software for estimating genotypes and genotypes probabilities. Markers inputted in `OneMap` included multiallelic markers and segregation distortion, were filtered by genotype probability higher than 0.8, kept only informative markers, and the AD and GQ fields were replaced by missing data when GT is missing.

Supplementary Figure S30. The total number of recombination breakpoints estimated for each progeny individual of the rose full-sib population with selected pipelines.

Supplementary Figure S31. Recombination fraction matrix heat map obtained for 37% of chromosome 10 of aspen data set by selected pipelines. The heat maps represent the recombination fraction matrix between markers positioned at both axes.

Supplementary Figure S32. Recombination fraction matrix heat map obtained for 37% of chromosome 1 of rose data set by selected pipelines. The heat maps represent the recombination fraction matrix between markers positioned at both axes.

Supplementary Figure S33. A: relation of the Euclidean distance between simulated data set with (x-axis) and without (y-axis) multiallelic markers. B: Number of multiallelic markers present in data sets represented in the x-axis of graphic A. The lines crossing the symbols indicate the standard deviation across the five repetitions. The data sets shown in this figure have allele depth counts from the VCF file, segregation distortion, were filtered by a minimum genotype probability of 0.8, and only informative markers.

Supplementary Figure S34. A: relation of map size between empirical data set with (x-axis) and without (y-axis) multiallelic markers. B: Number of multiallelic markers present in data sets represented in the x-axis of graphic A. The data sets shown in this figure have allele depth counts from the VCF file, were filtered by a minimum genotype probability of 0.8, and only informative markers and AD and GQ VCF fields were replaced by missing when GT is missing.

Abbreviations

GBS: Genotyping-by-Sequencing; PCR: polymerase chain reaction; RADSeq: Restriction-site associated; DNA sequencing; VCF: variant call format; GQ: genotyping quality; GT: genotype; GWAS: genome-wide association; SNP: single nucleotide polymorphism; LD: linkage disequilibrium; QTL: quantitative trait loci; WDL: workflow description language; HPRC: high performance research computing; CPU: central processing unit; HMM: hidden Markov model; EM: expectation-maximization; MAF: minor allele frequency; NGS: Next Generation Sequencing.

Competing Interests

The authors declare that they have no competing interests.

Funding

This work was partially supported by the National Council for Scientific and Technological Development (CNPq - 313269/2021-1); by USDA, National Institute of Food and Agriculture (NIFA), Specialty Crop Research Initiative (SCRI) project "Tools for Genomics-Assisted Breeding in Polyploids: Development of a Community Resource" (Award No. 2020-51181-32156); and by the Bill and Melinda Gates Foundation (OPP1213329) project SweetGAINS. TPO acknowledges funding from the European Union's Horizon 2020 research and innovation program under the Marie Sklodowska-Curie grant agreement No. 801215 and the University of Edinburgh Data-Driven Innovation program part of the Edinburgh and South East Scotland City Region Deal.

Author's Contributions

CHT, MM, RRA, AAFG, GSP and GCF contributed to OneMap package updates. CHT, LMT, GSG, GSP, AAGF, MM, ORL and JL contributed with ideas to design Reads2Map. CHT and LMT developed and optimized the Reads2Map code. CHT developed Reads2MapApp. CHT, TPO, AAFG, ORL, DB, and RRA contributed to elaborate the tested scenarios. CHT, TPO and RRA contributed to analyze the results. CHT wrote the first version of the manuscript. All authors provided helpful discussions for the work and reviewed the manuscript.

Acknowledgements

Portions of this research were conducted with the advanced computing resources provided by Texas A&M High Performance Research Computing and by University of São Paulo Aguia High Performance Computing.

References

1. Glaubitz JC, Casstevens TM, Lu F, Harriman J, Elshire RJ, Sun Q, et al. TASSEL-GBS: a high capacity genotyping by sequencing analysis pipeline. PLoS ONE 2014 2;9:1–11.
2. Catchen J, Hohenlohe PA, Bassham S, Amores A, Cresko WA. Stacks: an analysis tool set for population genomics. Molecular Ecology 2013;22:3124–40.
3. Anderson CB, Franzmayr BK, Hong SW, Larking AC, Stijn TC, Tan R, et al. Protocol: A versatile, inexpensive, high-throughput plant genomic DNA extraction method suitable for genotyping-by-sequencing. Plant Methods 2018 8;14.
4. Andrews KR, Good JM, Miller MR, Luikart G, Hohenlohe PA. Harnessing the power of RADseq for ecological and evolutionary genomics. Nature Reviews Genetics 2016 1;17:81–92.
5. Bresadola L, Link V, Buerkle CA, Lexer C, Wegmann D. Estimating and accounting for genotyping errors in RAD-seq experiments. Molecular Ecology Resources 2020;20:856–870.
6. Baird NA, Etter PD, Atwood TS, Currey MC, Shiver AL, Lewis ZA, et al. Rapid SNP Discovery and Genetic Mapping Using Sequenced RAD Markers. PLoS ONE 2008;3:e3376.
7. Elshire RJ, Glaubitz JC, Sun Q, Poland JA, Kawamoto K, Buckler ES, et al. A robust, simple genotyping-by-sequencing (GBS) approach for high diversity species. PLoS ONE 2011 5;6:e19379.
8. der Auwera GV, O'Connor B. Genomics in the Cloud: Using Docker, GATK, and WDL in Terra. O'Reilly Media, Incorporated; 2020.
9. Rivera-Colón AG, Rochette NC, Catchen JM. Simulation with RADinitio improves RADseq experimental design and sheds light on sources of missing data. Molecular Ecology Resources 2020;p. 1–16.
10. Gerard D, Ferrão LFV, Garcia AAF, Stephens M. Genotyping Polyploids from Messy Sequencing Data. Genetics 2018 11;210:789–807.
11. a Hackett C, Broadfoot LB. Effects of genotyping errors, missing values and segregation distortion in molecular marker data on the construction of linkage maps. Heredity 2003 1;90:33–38.
12. Sturtevant AH. The behavior of the chromosomes as studied through linkage. Zeitschrift für induktive Abstammungs- und Vererbungslehre 1915;13:234–287.
13. Smith GR, Nambiar M. New Solutions to Old Problems: Molecular Mechanisms of Meiotic Crossover Control. Trends in Genetics 2020;36:337–346.
14. Bilton TP, Schofield MR, Black MA, Chagné D, Wilcox PL, Dodds KG. Accounting for Errors in Low Coverage High-Throughput Sequencing Data When Constructing Genetic Maps Using Biparental Outcrossed Populations. Genetics 2018 5;209:65–76.
15. Mollinari M, Garcia AAF. Linkage Analysis and Haplotype Phasing in Experimental Autopolyploid Populations with High Ploidy Level Using Hidden Markov Models. G3: Genes|Genomes|Genetics 2019 10;9:3297–3314.
16. Liao Y, Voorrips RE, Bourke PM, Tumino G, Arens P, Visser RGF, et al. Using probabilistic genotypes in linkage analysis of polyploids. Theoretical and Applied Genetics 2021 8;134:2443–2457.
17. Margarido GRA, Souza AP, Garcia AAF. OneMap: software for genetic mapping in outcrossing species. Hereditas 2007 7;144:78–9.
18. Lander ES, Green P. Construction of multilocus genetic linkage maps in humans. Proc Natl Acad Sci USA 1987;84:2363–2367.
19. Lorenz AJ, Hamblin MT, Jannink JL. Performance of single nucleotide polymorphisms versus haplotypes for genome-wide association analysis in barley. PLoS ONE 2010;5:1–11.
20. Gawenda I, Thorwarth P, Günther T, Ordon F, Schmid KJ. Genome-wide association studies in elite varieties of German winter barley using single-marker and haplotype-based methods. Plant Breeding 2015 2;134:28–39.
21. N'Diaye A, Haile JK, Fowler DB, Ammar K, Pozniak CJ. Effect of Co-segregating Markers on High-Density Genetic Maps and Prediction of Map Expansion Using Machine Learning Algorithms. Frontiers in Plant Science 2017 8;8.
22. Sehgal D, Dreisigacker S. Haplotypes-based genetic analysis: Benefits and challenges. Vavilovskii Zhurnal Genetiki i Selektsii 2019;23:803–808.
23. Abed A, Belzile F. Comparing Single-SNP, Multi-SNP, and Haplotype-Based Approaches in Association Studies for Major Traits in Barley. The Plant Genome 2019;12:190036.
24. Liu N, Zhang K, in Genetics Zhao HBTA. Haplotype-Association Analysis. Genetic Dissection of Complex Traits 2008;60:335–405.
25. Jiang Y, Schmidt RH, Reif JC. Haplotype-Based Genome-Wide Prediction Models Exploit Local Epistatic Interactions Among Markers. G3: Genes|Genomes|Genetics 2018;49:g3.300548.2017.
26. Garrison E, Marth G. Haplotype-based variant detection from short-read sequencing. ArXiv e-prints 2012;p. 9.
27. McKenna A, Hanna M, Banks E, Sivachenko A, Cibulskis K, Kernytsky A, et al. The Genome Analysis Toolkit: A MapReduce framework for analyzing next-generation DNA sequencing data. Genome Research 2010 9;20:1297–1303.
28. Clark LV, Lipka AE, Sacks EJ. polyRAD: Genotype Calling with Uncertainty from Sequencing Data in Polyploids and Diploids. G3: Genes|Genomes|Genetics 2019;9:g3.200913.2018.
29. Serang O, Mollinari M, Garcia AAF. Efficient exact maximum a posteriori computation for bayesian SNP genotyping in polyploids. PLoS ONE 2012;7:1–13.
30. Patterson M, Marschall T, Pisanti N, van Iersel L, Stougie L, Klau GW, et al. WhatsHap: Weighted Haplotype Assembly for Future-Generation Sequencing Reads. Journal of Computational Biology 2015;22:498–509.
31. Voss K, Gentry J, Auwera GVD. Full-stack genomics pipelining with GATK4+ WDL+ Cromwell [version 1; not peer reviewed]. F1000Research 2017;p. 4.
32. Merkel D. Docker : Lightweight Linux Containers for Consistent Development and Deployment Docker : a Little Background Under the Hood. Linux Journal 2014;2014:2–7.
33. Kurtzer GM, Sochat V, Bauer MW. Singularity: Scientific containers for mobility of compute. PLOS ONE 2017 5;12:e0177459.
34. Broman KW, Wu H, Sen S, Churchill GA. R/qtl: QTL mapping in experimental crosses. Bioinformatics 2003;19:889–890.
35. Broman KW, Śaunak Sen, Sen S. A Guide to QTL Mapping with R/qtl, vol. 66. Springer New York; 2009.
36. Maliepaard C, Jansen J, Ooijen JWV. Linkage analysis in a full-sib family of an outbreeding plant species : overview and consequences for applications. Genetical Research 1997;70:237–250.
37. Baum E, Petrie T, G S, N W. A Maximization Technique Occurring in the Statistical Analysis of Probabilistic Functions of Markov Chains. The Annals of Mathematical Statistics 1970;41:164–171.
38. Schiffthaler B, Bernhardsson C, Ingvarsson PK, Street NR. BatchMap: A parallel implementation of the OneMap R package for fast computation of F1 linkage maps in outcrossing species. PLoS ONE 2017;12:1–12.
39. Zhigunov AV, Ulianich PS, Lebedeva MV, Chang PL, Nuzhdin SV, Potokina EK. Development of F1 hybrid population and the high-density linkage map for European aspen (Populus tremula L.) using RADseq technology. BMC Plant Biology 2017;17.
40. Young EL, Lau J, Bentley NB, Rawandoozi Z, Collins S, Windham MT, et al. Identification of QTLs for Reduced Susceptibility to Rose Rosette Disease in Diploid Roses. Pathogens 2022 6;11:660.
41. Tuskan GA, DiFazio S, Jansson S, Bohlmann J, Grigoriev I, Hellsten U, et al. The Genome of Black Cottonwood, Populus trichocarpa. Science 2006 9;313:1596–1604.
42. Saint-Oyant LH, Ruttink T, Hamama L, Kirov I, Lakhwani D, Zhou NN, et al. A high-quality genome sequence of Rosa chinen-

sis to elucidate ornamental traits. Nature Plants 2018 7;4:473–484.
43. Martin M. Cutadapt removes adapter sequences from high-throughput sequencing reads. EMBnetjournal 2011 5;17:10.
44. Hyman JM. Accurate Monotonicity Preserving Cubic Interpolation. SIAM Journal on Scientific and Statistical Computing 1983 12;4:645–654.
45. Wu R, Ma CX, Wu SS, Zeng ZB. Linkage mapping of sex-specific differences. Genetical research 2002;79:85–96.
46. Voorrips RE, Maliepaard CA. The simulation of meiosis in diploid and tetraploid organisms using various genetic models. BMC Bioinformatics 2012 12;13:248.
47. Haldane JBS. The combination of linkage values, and the calculation of distance between linked factors. Journal of Genetics 1919;8:299–309.
48. Best K, Oakes T, Heather JM, Shawe-Taylor J, Chain B. Computational analysis of stochastic heterogeneity in PCR amplification efficiency revealed by single molecule barcoding. Scientific reports 2015 10;5:14629.
49. Glenn TC. Field guide to next-generation DNA sequencers. Molecular Ecology Resources 2011;11:759–769.
50. Li H. seqtk: Toolkit for processing sequences in FASTA/Q formats. seqtk GitHub repository 2020;https://github.com/lh3/seqtk.
51. Li H. Aligning sequence reads, clone sequences and assembly contigs with BWA-MEM. ArXiv 2013;1303.
52. Li H, Handsaker B, Wysoker A, Fennell T, Ruan J, Homer N, et al. The sequence alignment/map format and SAMtools. Bioinformatics 2009;25:2078–2079.
53. Ros-Freixedes R, Gonen S, Gorjanc G, Hickey JM. A method for allocating low-coverage sequencing resources by targeting haplotypes rather than individuals. Genetics Selection Evolution 2017;49:1–17.
54. Knaus BJ, Grünwald NJ. vcfR: a package to manipulate and visualize variant call format data in R. Molecular Ecology Resources 2017 1;17:44–53.
55. Preedy KF, Hackett CA. A rapid marker ordering approach for high-density genetic linkage maps in experimental autotetraploid populations using multidimensional scaling. Theoretical and Applied Genetics 2016;.
56. Berkson J. Application of the Logistic Function to Bio-Assay. Journal of the American Statistical Association 1944 3;39:357–365.
57. Mollinari M, Margarido GRA, Vencovsky R, Garcia AAF. Evaluation of algorithms used to order markers on genetic maps. Heredity 2009;103:494–502.
58. Guyader V, Fay C, Rochette S, Girard C. golem: A Framework for Robust Shiny Applications. Golem GitHub repository 2022;https://github.com/ThinkR-open/golem.
59. DePristo MA, Banks E, Poplin R, Garimella KV, Maguire JR, Hartl C, et al. A framework for variation discovery and genotyping using next-generation DNA sequencing data. Nature Genetics 2011;43:491–8.
60. Kagale S, Koh C, Clarke WE, Bollina V, Parkin IAP, Sharpe AG. Analysis of Genotyping-by-Sequencing (GBS) Data. Plant Bioinformatics 2016;p. 269–284.
61. Institute B. Picard Tools. Broad Institute, GitHub repository 2009;https://github.com/broadinstitute/picard.
62. Duncavage EJ, Coleman JF, de Baca ME, Kadri S, Leon A, Routbort M, et al. Recommendations for the Use of In silico Approaches for Next Generation Sequencing Bioinformatic Pipeline Validation: A Joint Report of the Association for Molecular Pathology, Association for Pathology Informatics, and College of American Pathologists. The Journal of molecular diagnostics : JMD 2022 10;.
63. Grattapaglia D, Sederoff R. Genetic linkage maps of Eucalyptus grandis and Eucalyptus urophylla using a pseudo-testcross: mapping strategy and RAPD markers. Genetics 1994 8;137:1121–37.
64. Gazaffi R, Margarido GRA, Pastina MM, Mollinari M, Garcia AAF. A model for quantitative trait loci mapping, linkage phase, and segregation pattern estimation for a full-sib progeny. Tree Genetics and Genomes 2014;10:791–801.
65. Os H, Stam P, Visser RGF, Eck HJ, Os HV, Stam P, et al. RECORD: a novel method for ordering loci on a genetic linkage map. Theoretical and Applied Genetics 2005;112:30–40.
66. Taniguti CH. EmpiricalReads2Map. WorkflowHub 2022;https://doi.org/10.48546/WORKFLOWHUB.WORKFLOW.409.1.
67. Taniguti CH. SimulatedReads2Map. WorkflowHub 2022;https://doi.org/10.48546/WORKFLOWHUB.WORKFLOW.410.1.

Figure

Click here to access/download;Figure;depth_simu_joint2_edit.png

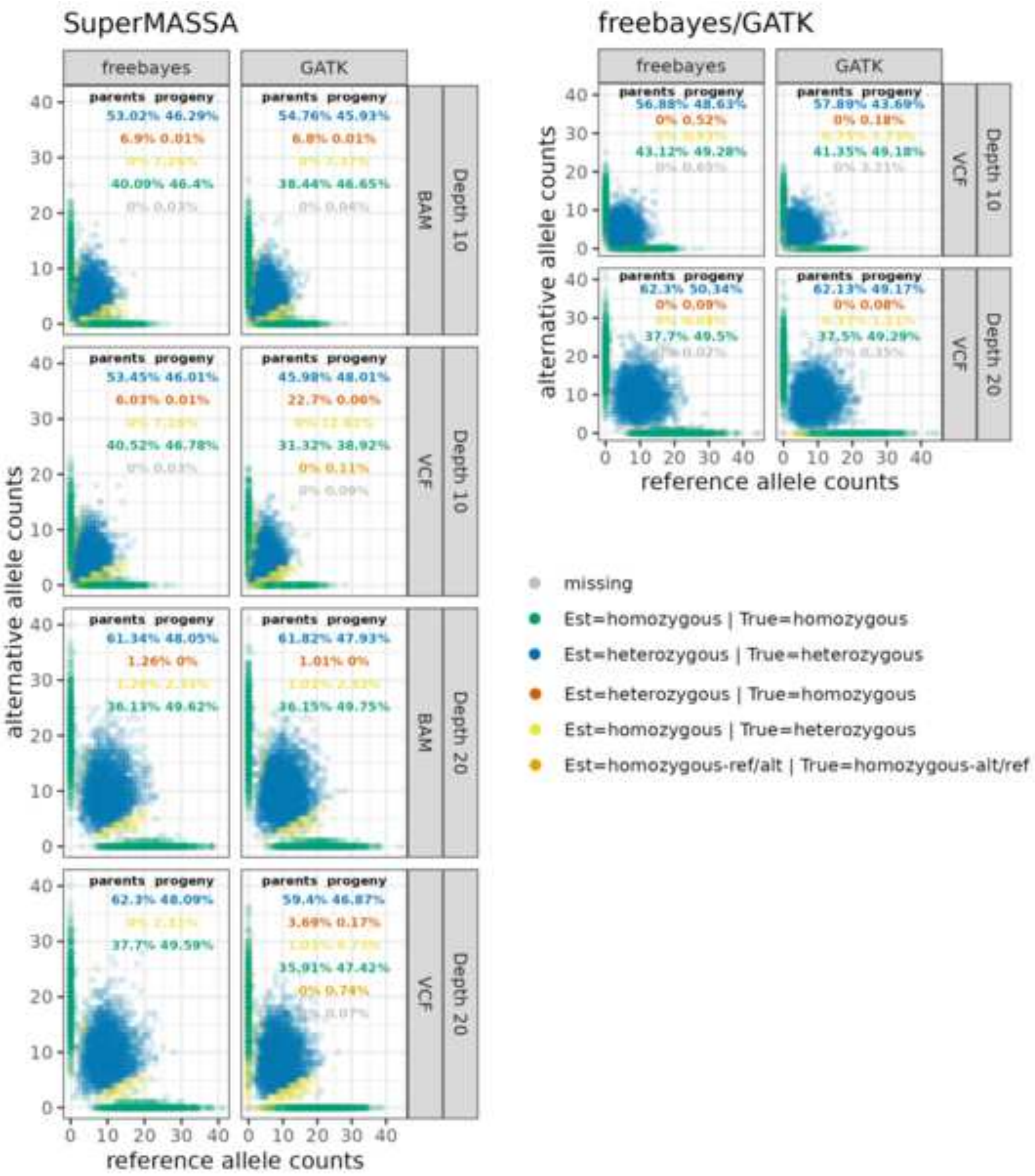

Figure

Click here to access/download;Figure;aspen_pipe2.png

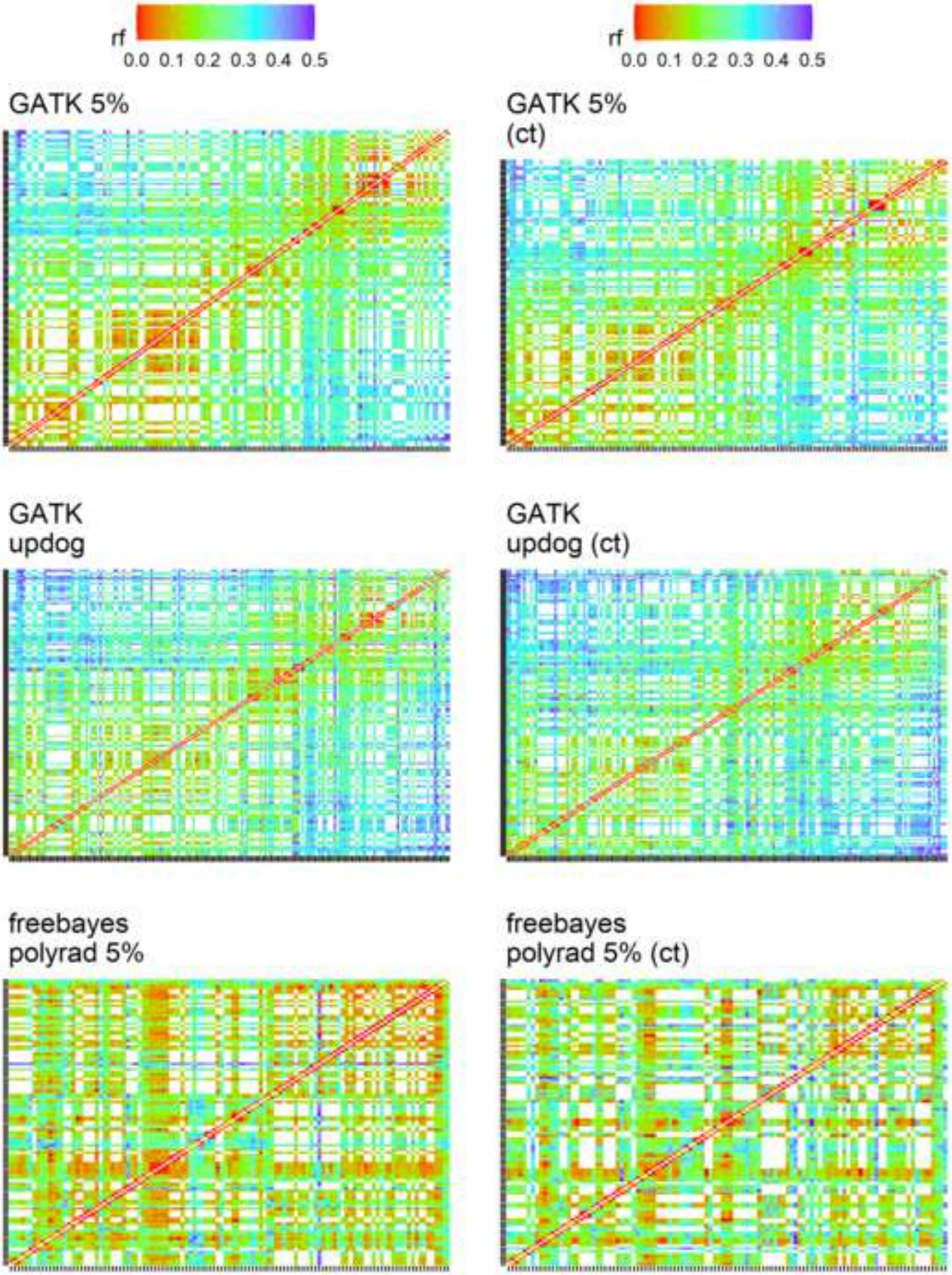

Click here to access/download;Figure;bixmulti_suppl.png

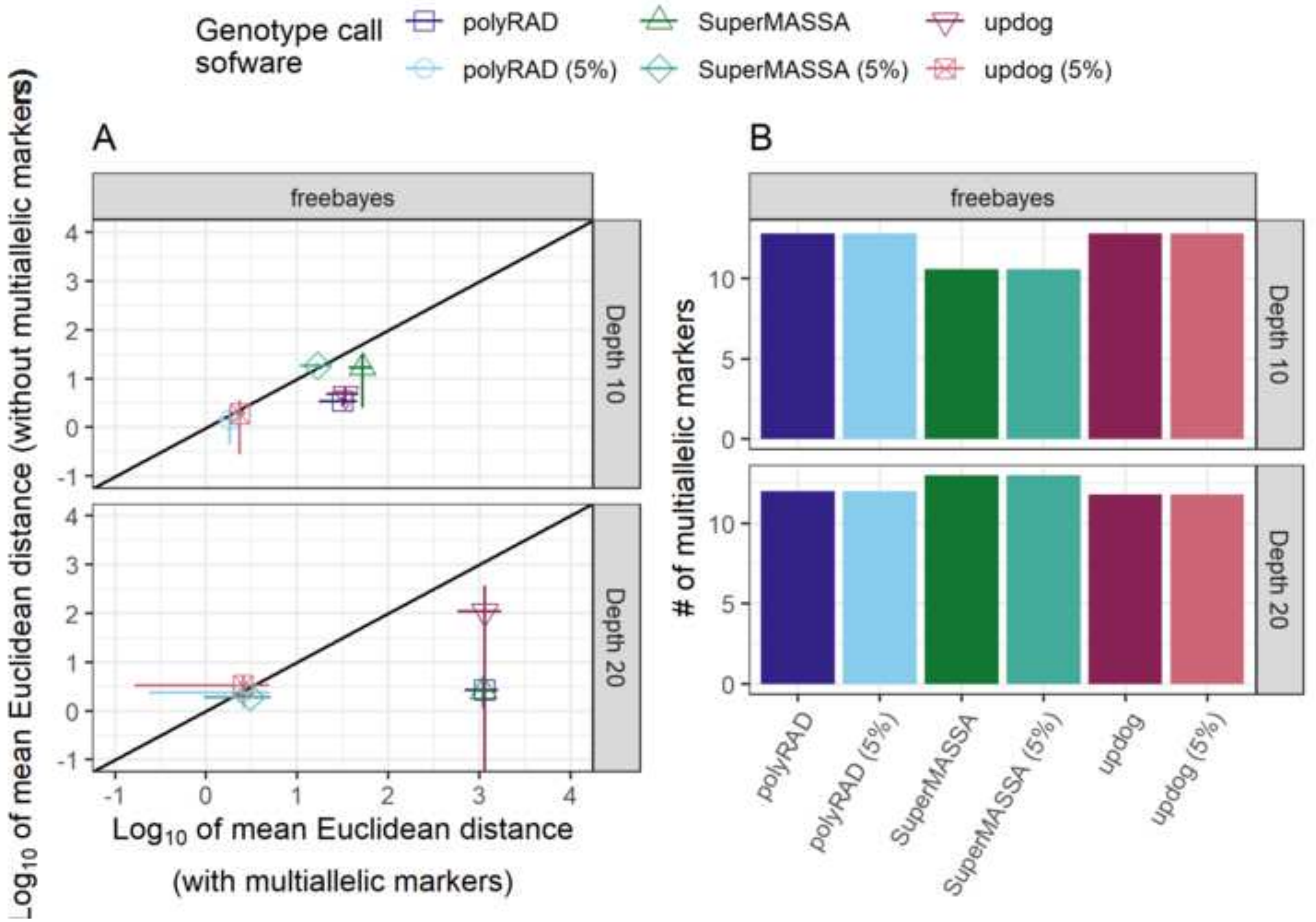

Click here to access/download;Figure;bixmulti_suppl_emp.png

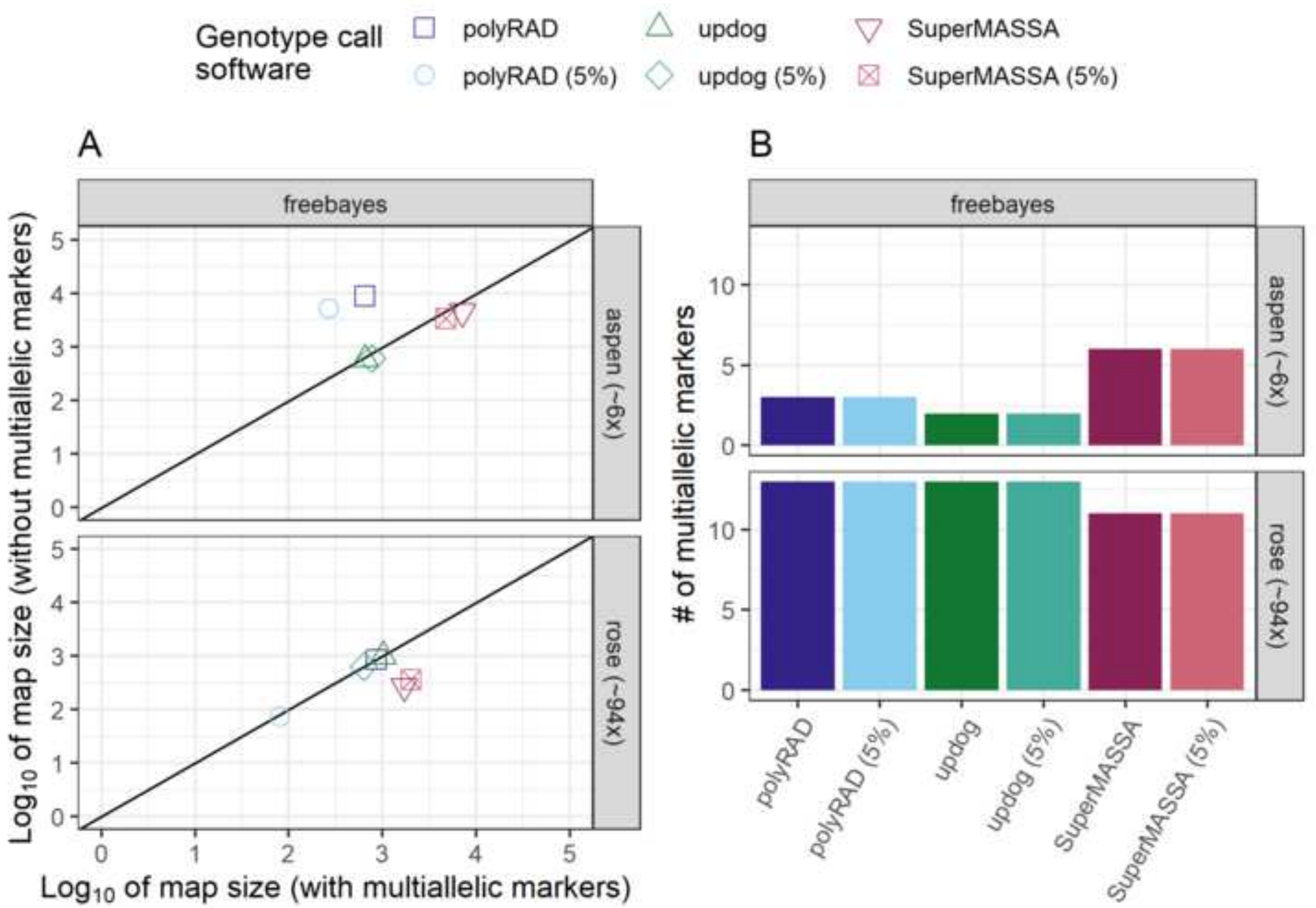

Figure Click here to access/download;Figure;depth_simu_joint1.png

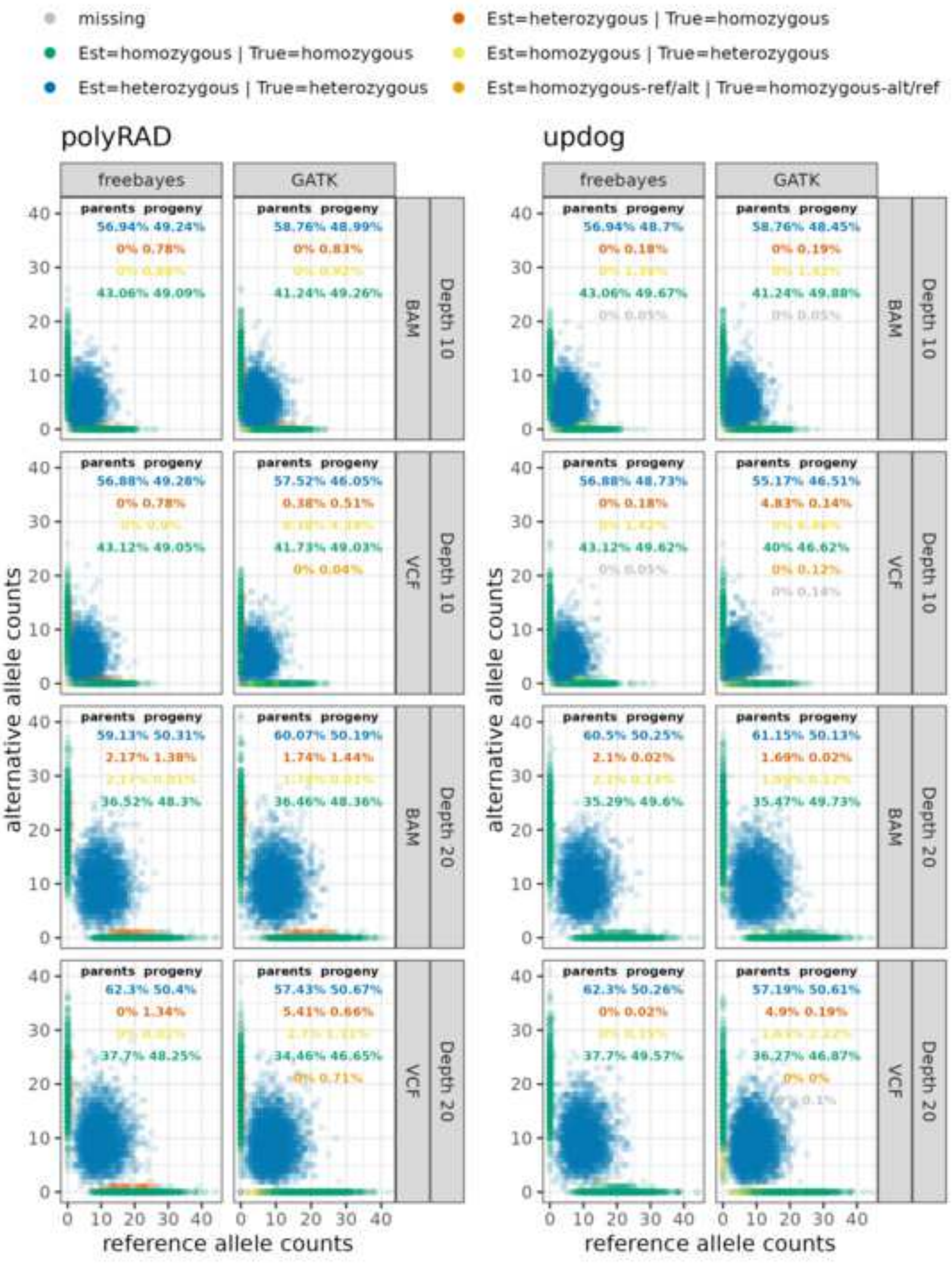

Figure

Click here to access/download;Figure;depth_simu_joint1_dev.png

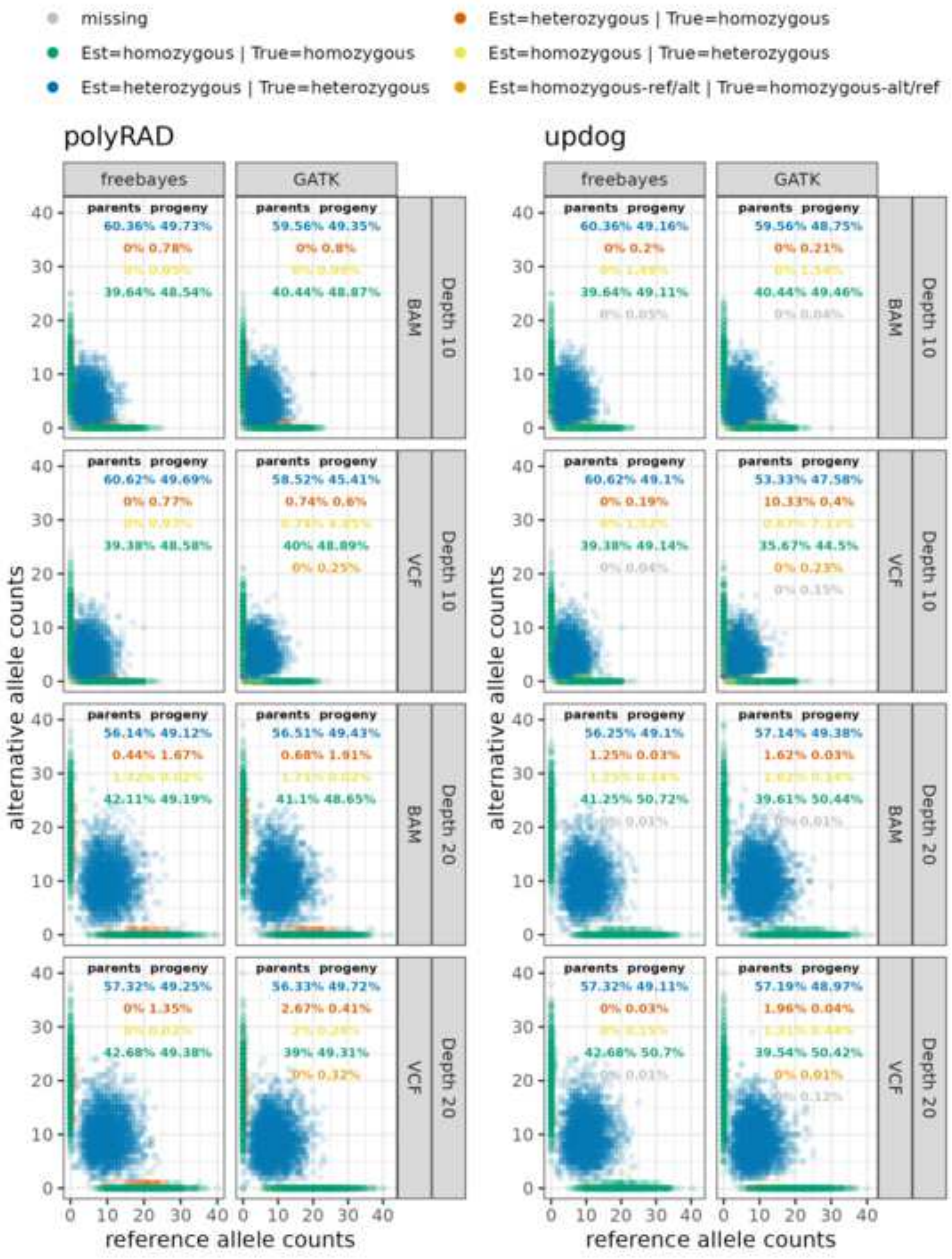

Figure

Click here to access/download;Figure;depth_simu_joint2_dev_edit.png

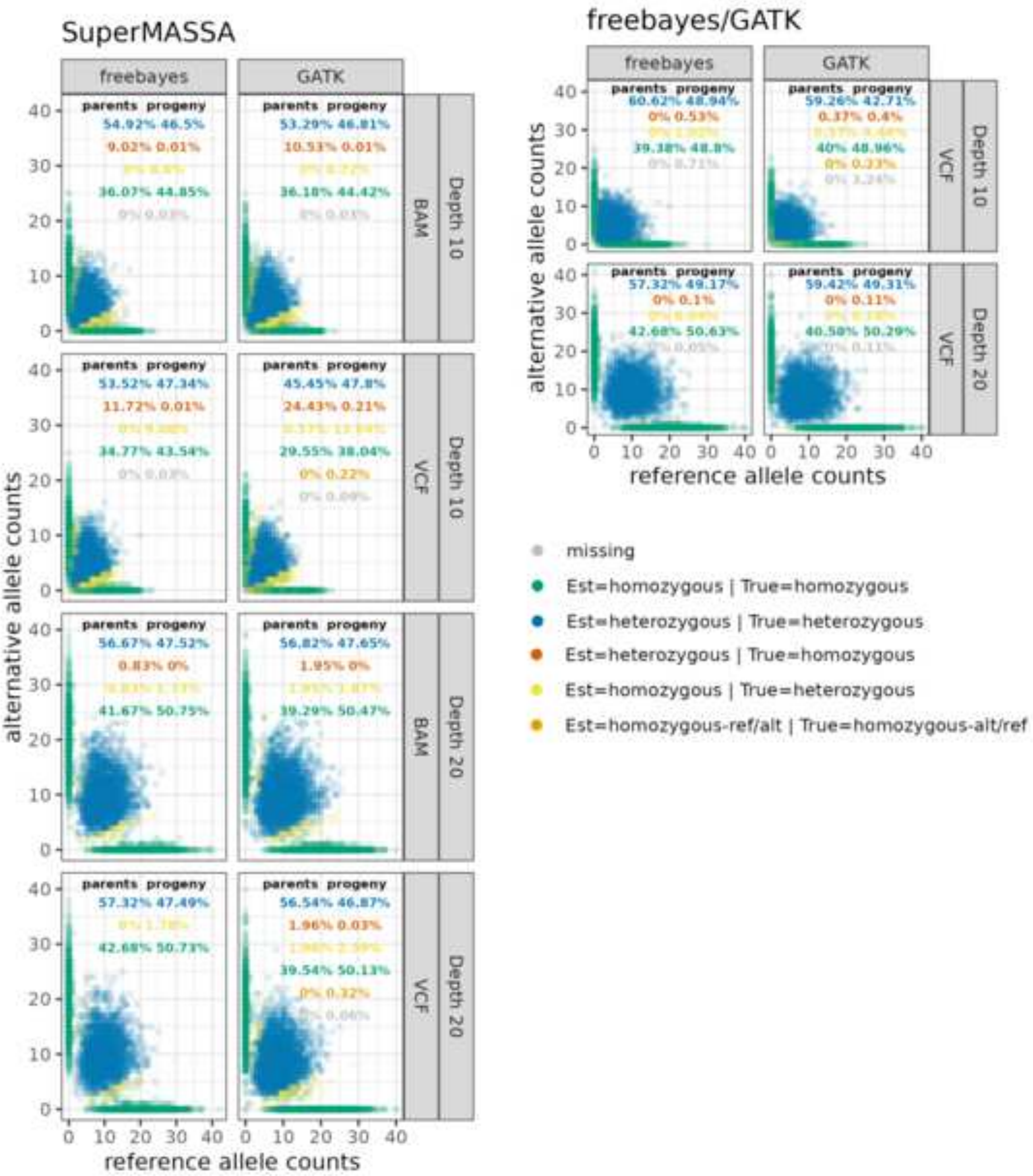

Figure
Click here to access/download;Figure;depths_emp1.png

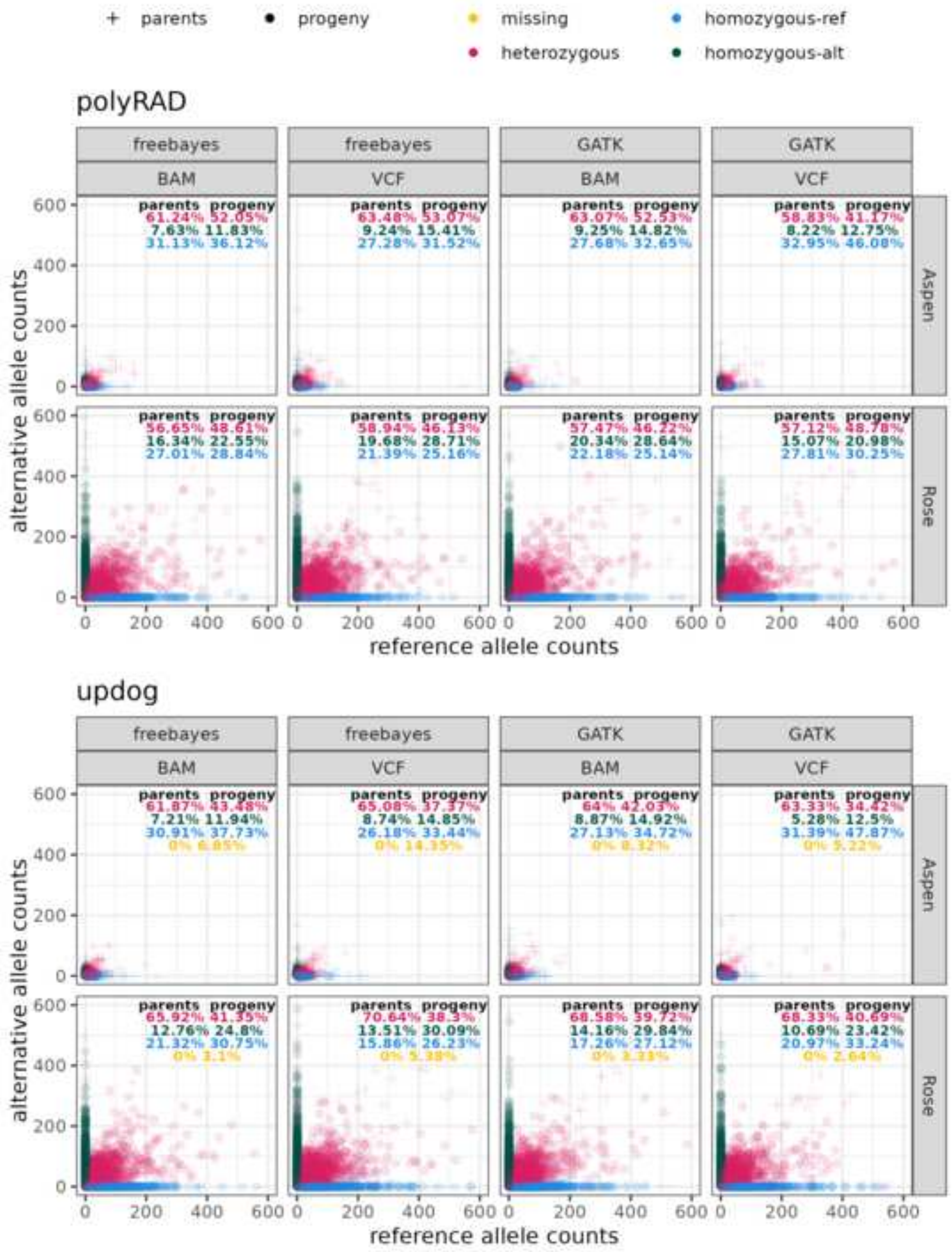

Figure Click here to access/download;Figure;depths_emp2.png

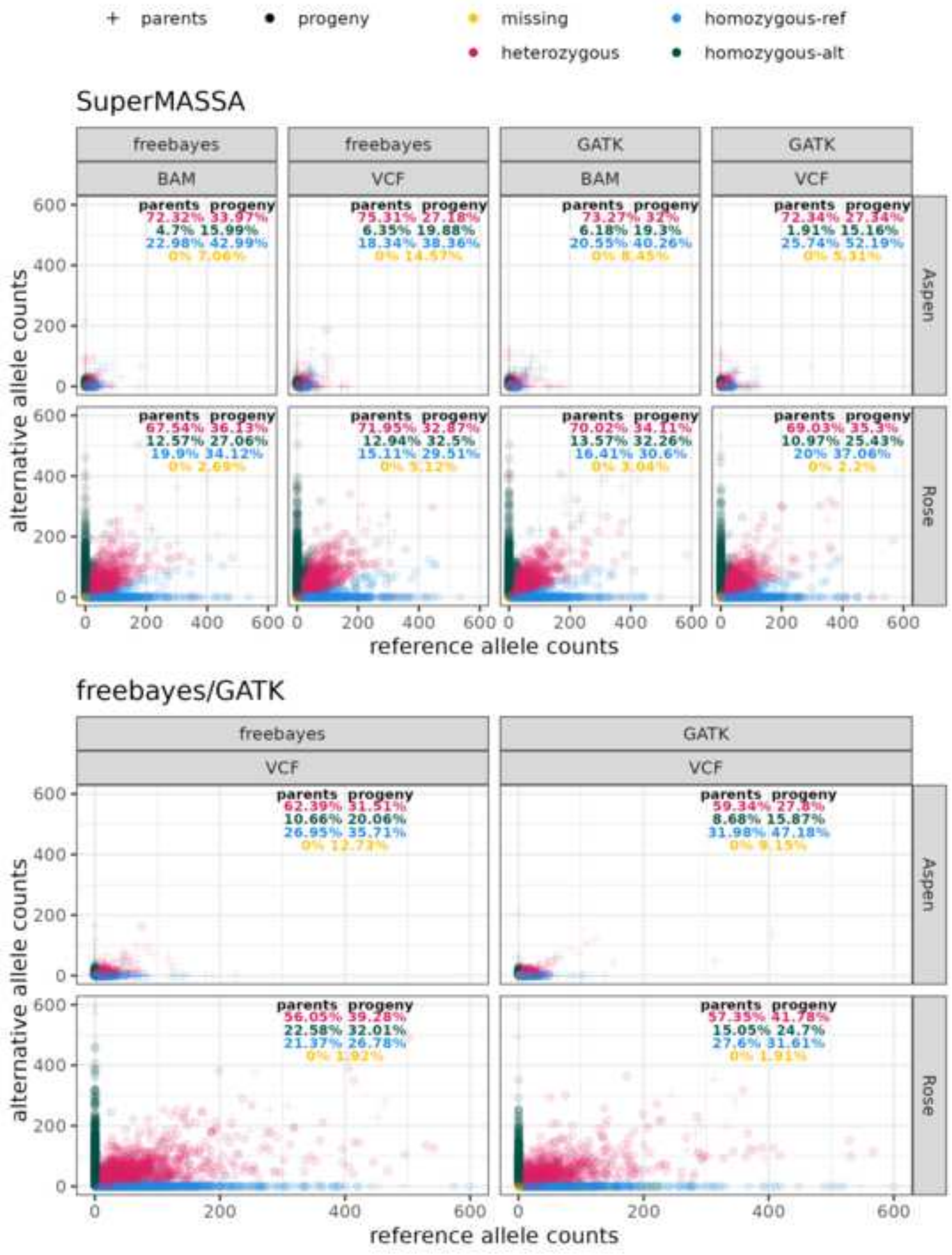

Click here to access/download;Figure;devxnondev_paper.png

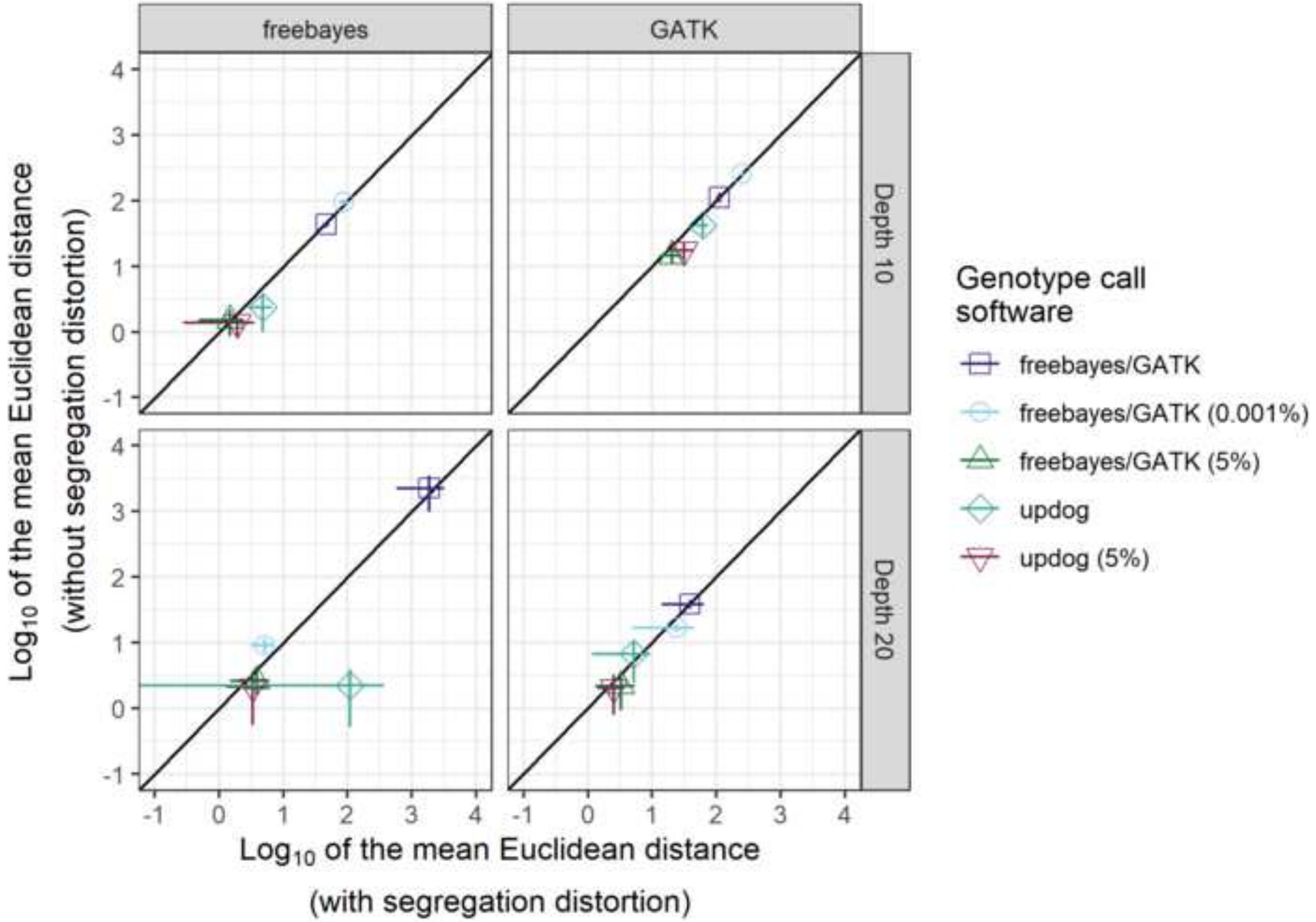

Figure

Click here to access/download;Figure;filters_supple.png

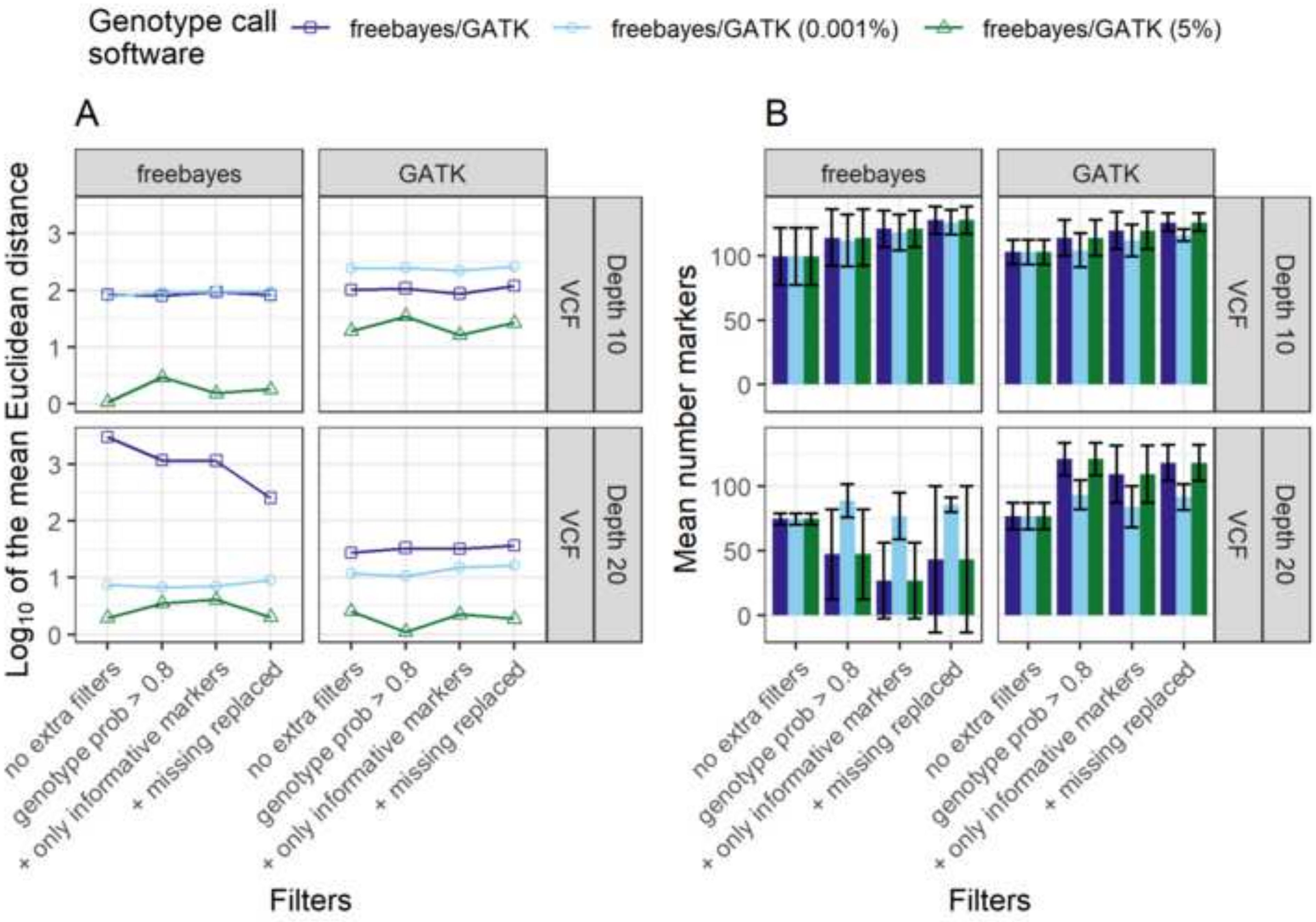

 Click here to access/download;Figure;filters_supple_emp.png

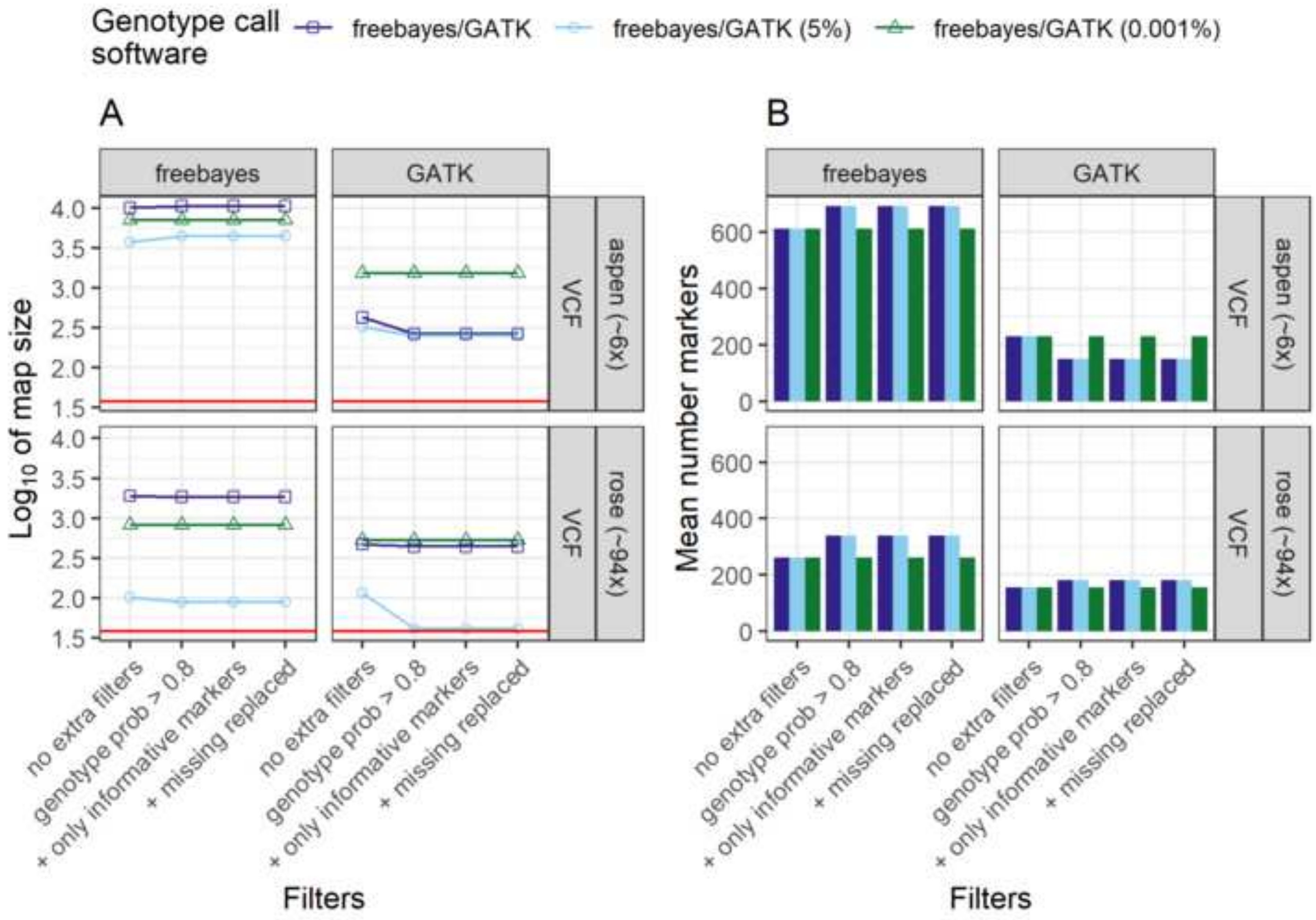

Click here to access/download;Figure;gus_suppl.png

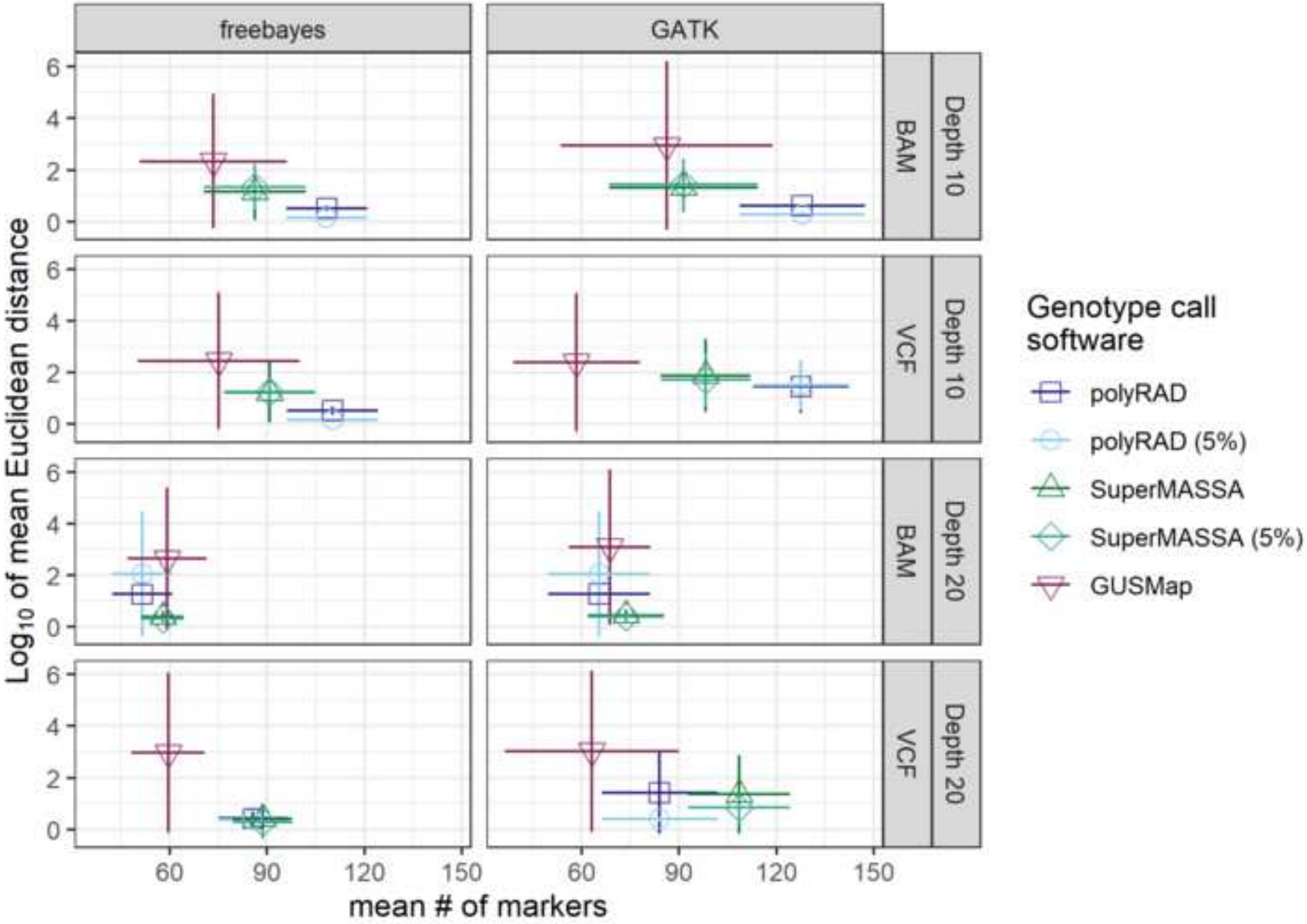

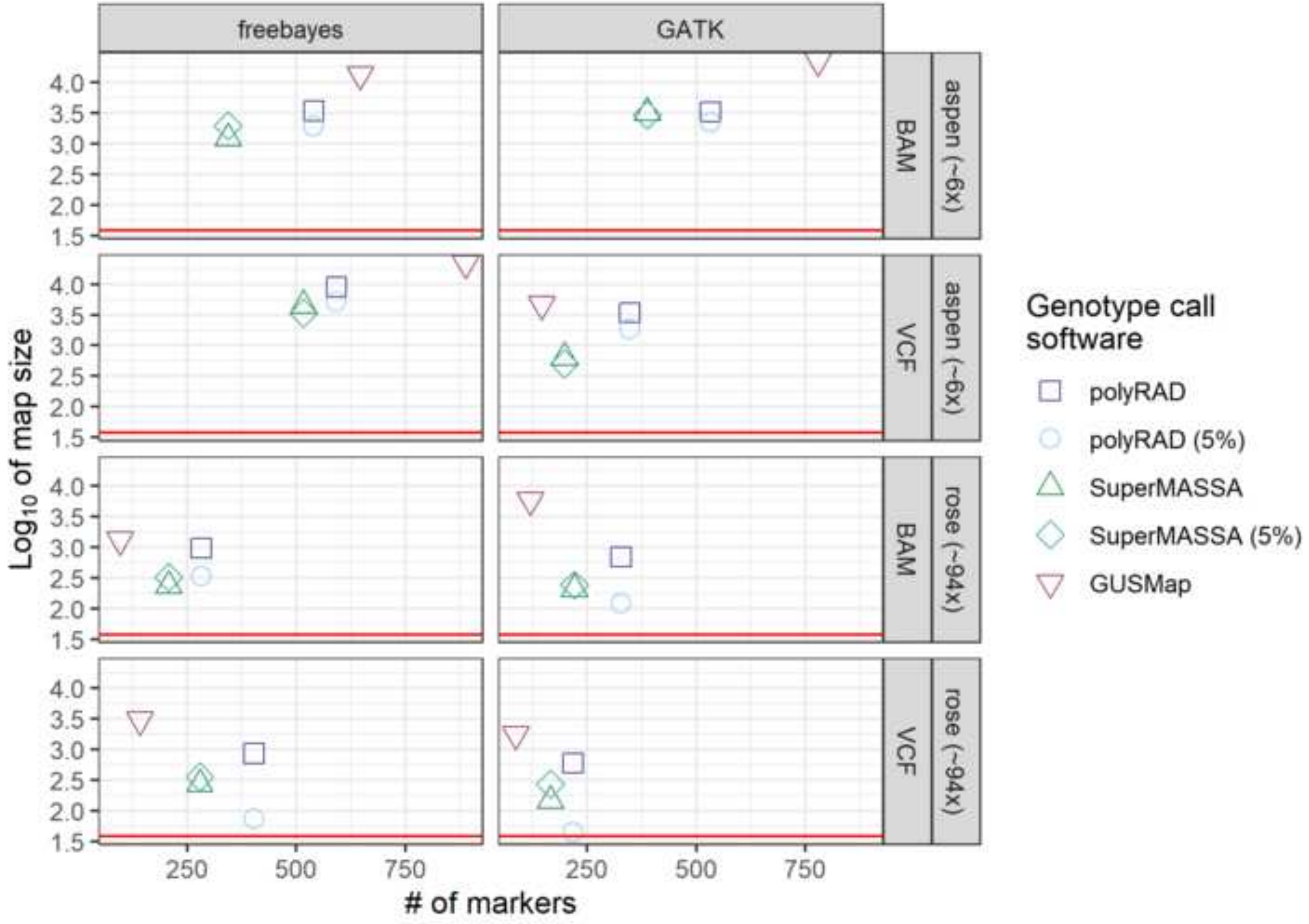
freebayes
GATK
BAM
VCF
aspen (~6x)
rose (~94x)
Log10 of map size
# of markers
4.0
3.5
3.0
2.5
2.0
1.5
250
500
750
Genotype call software
polyRAD
polyRAD (5%)
SuperMASSA
SuperMASSA (5%)
GUSMap

Figure

Click here to access/download;Figure;log10_efficiency_depth10.png

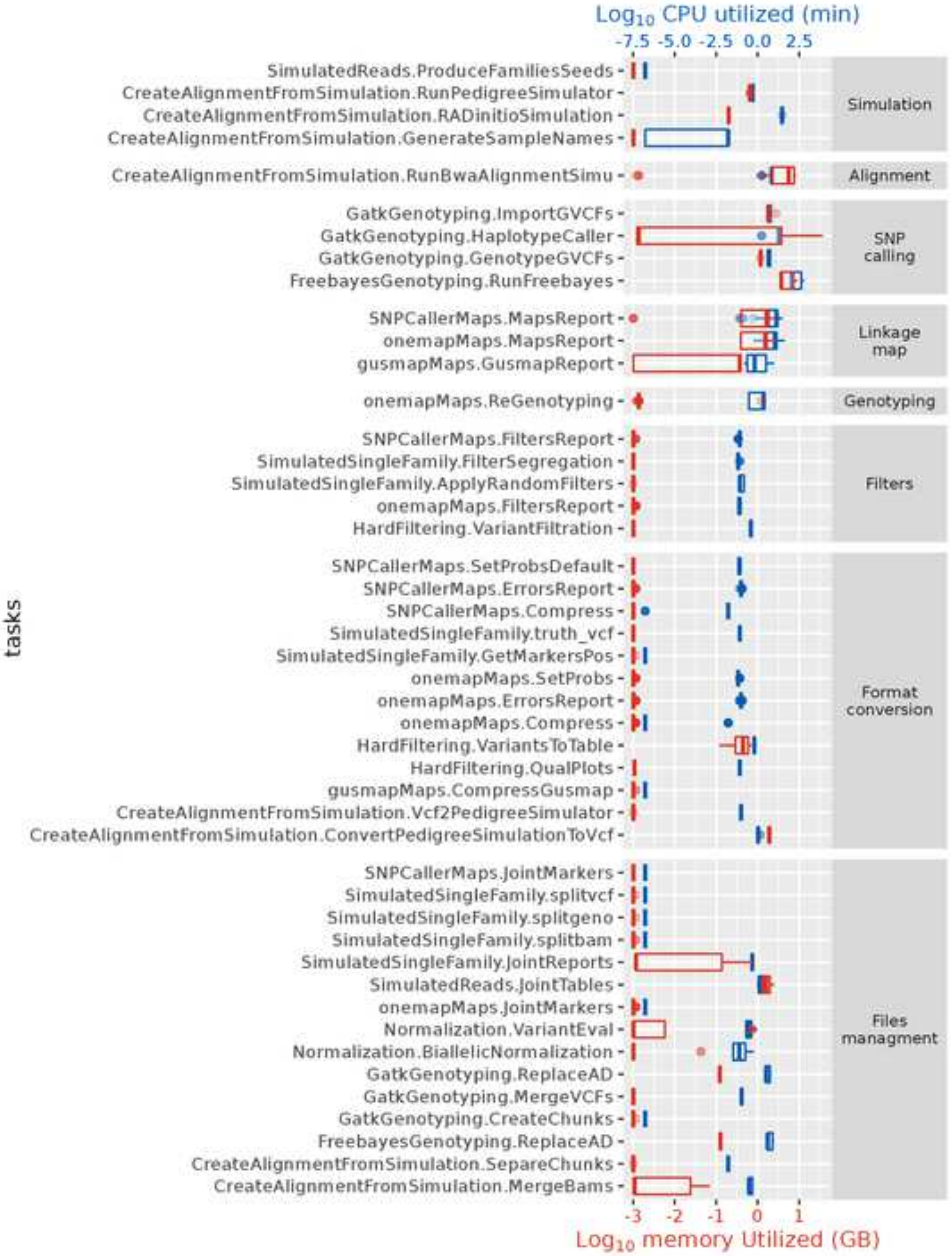

Figure

Click here to access/download;Figure;log10_efficiency_depth20.png

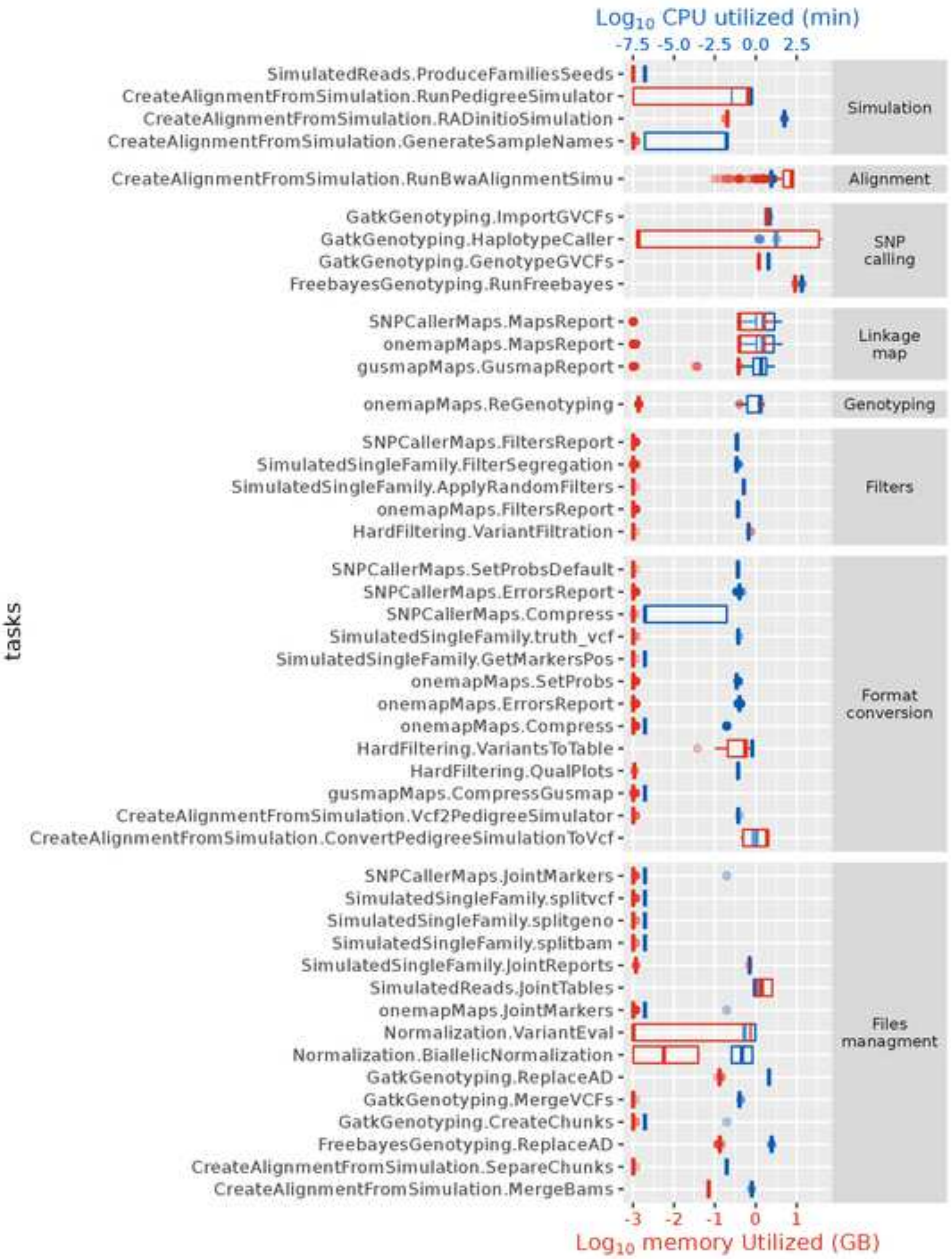

Figure

Click here to access/download;Figure;log10_efficiency_populus.png

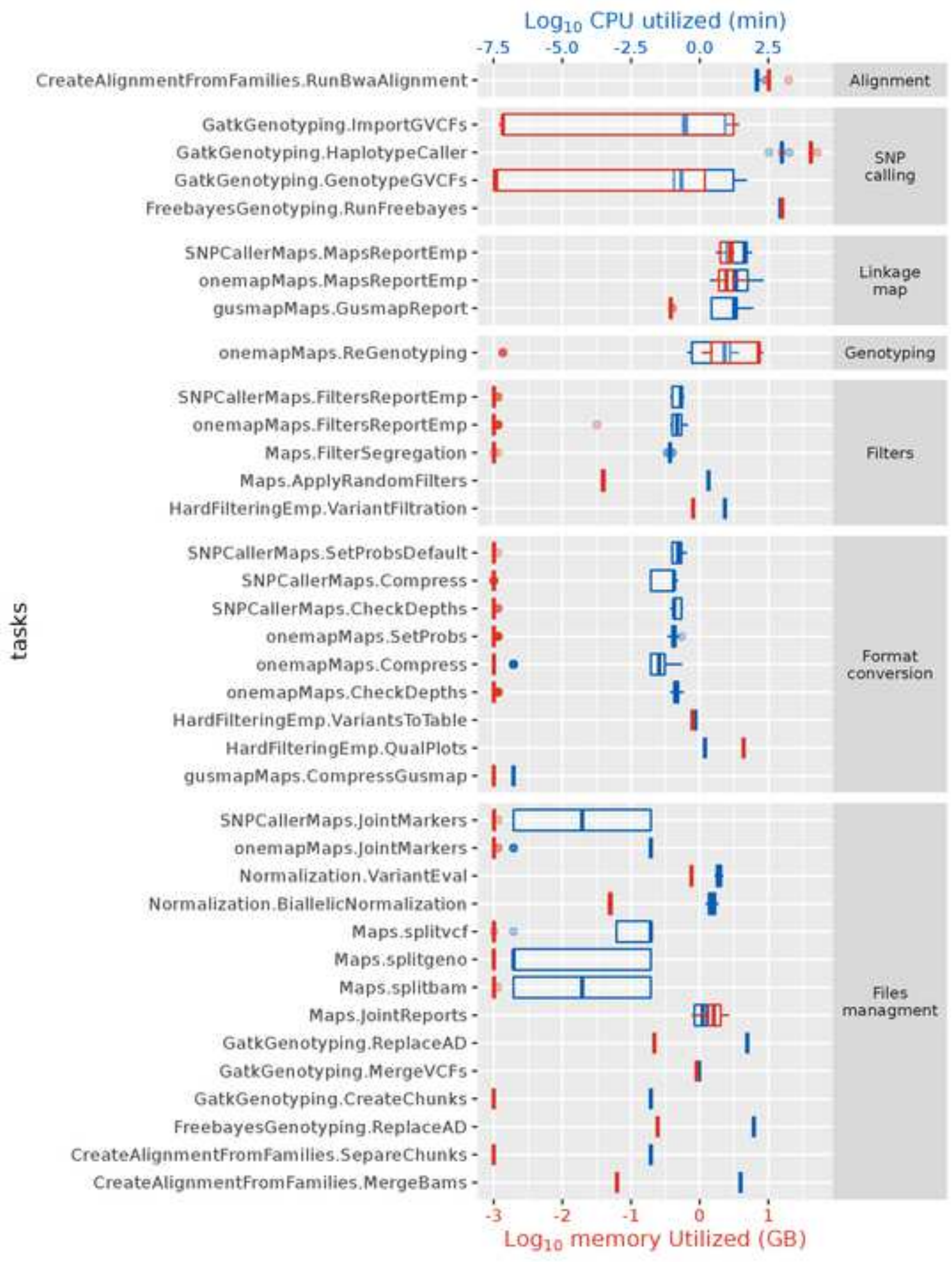

Figure

Click here to access/download;Figure;log10_efficiency_rose.png

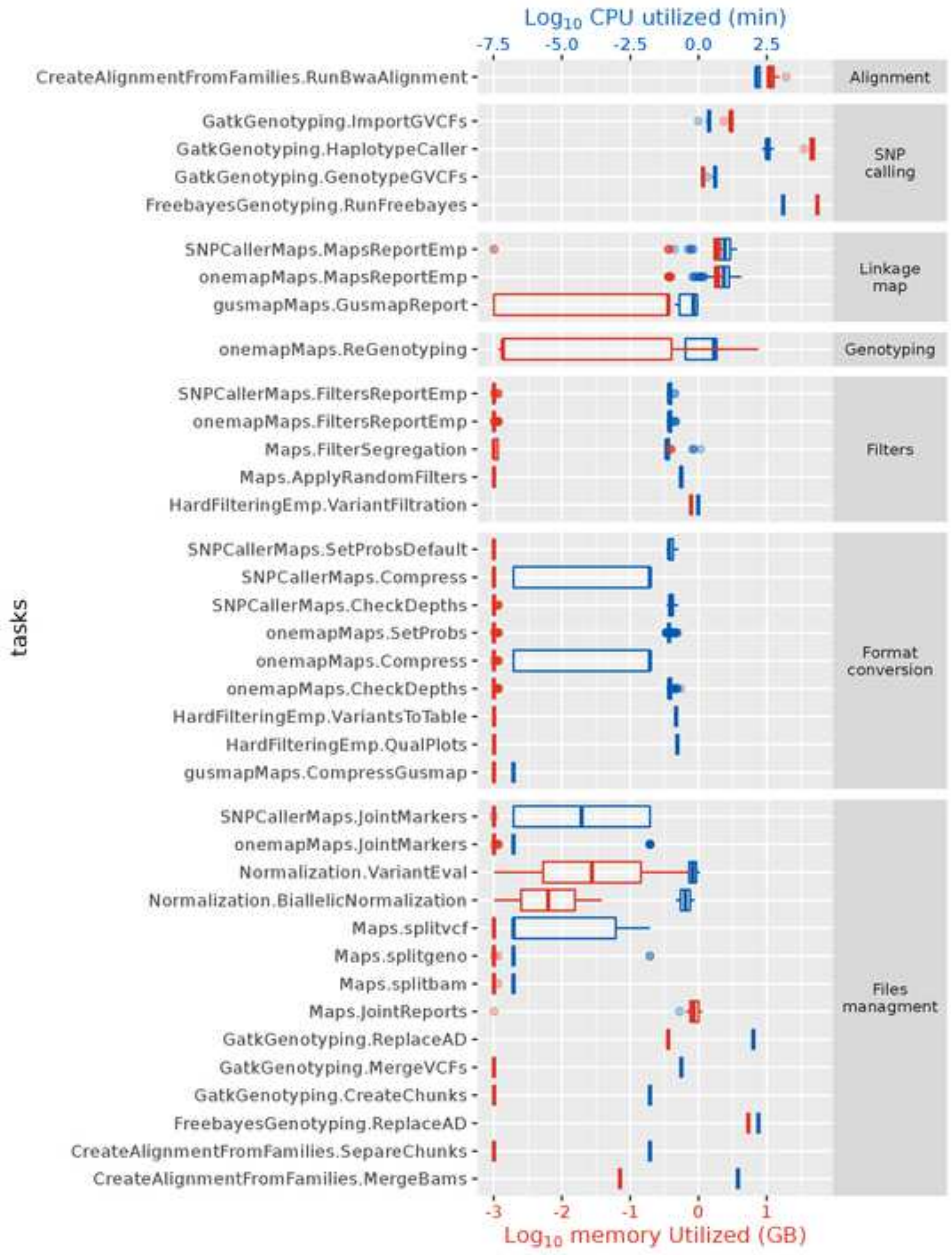

Figure Click here to access/download;Figure;log10_haplo10.png

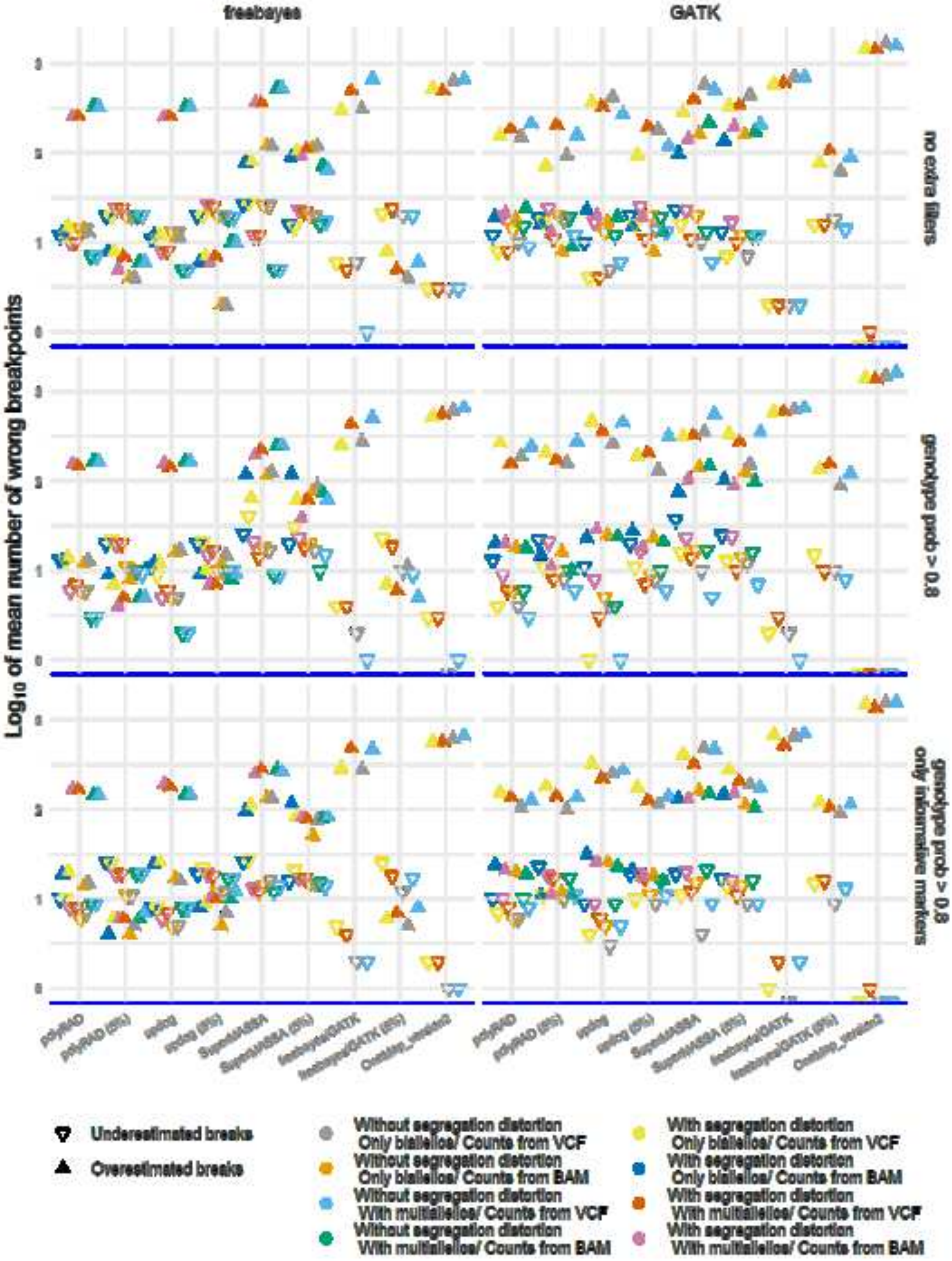

Figure

Click here to access/download;Figure;log10_haplo20.png

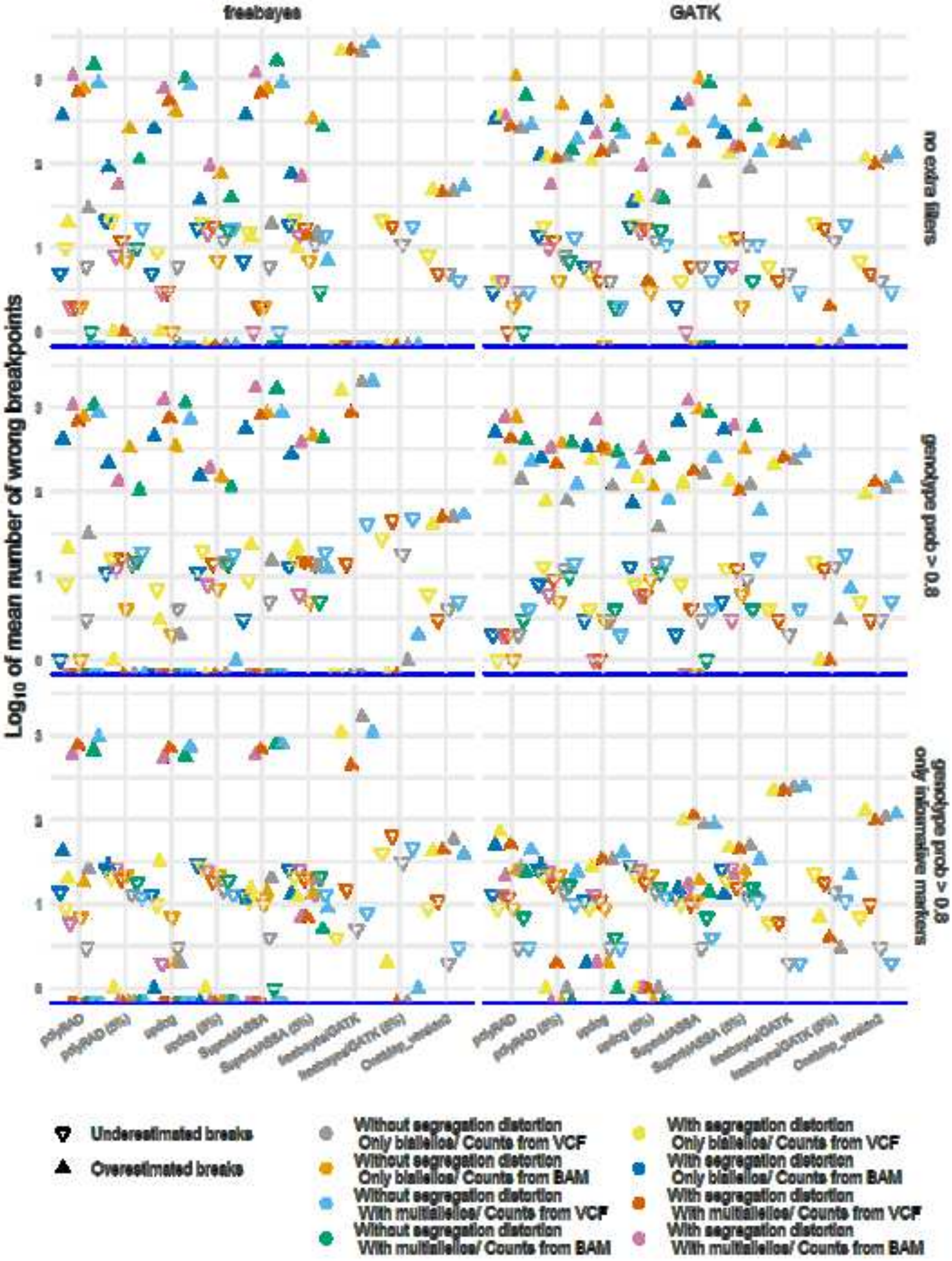

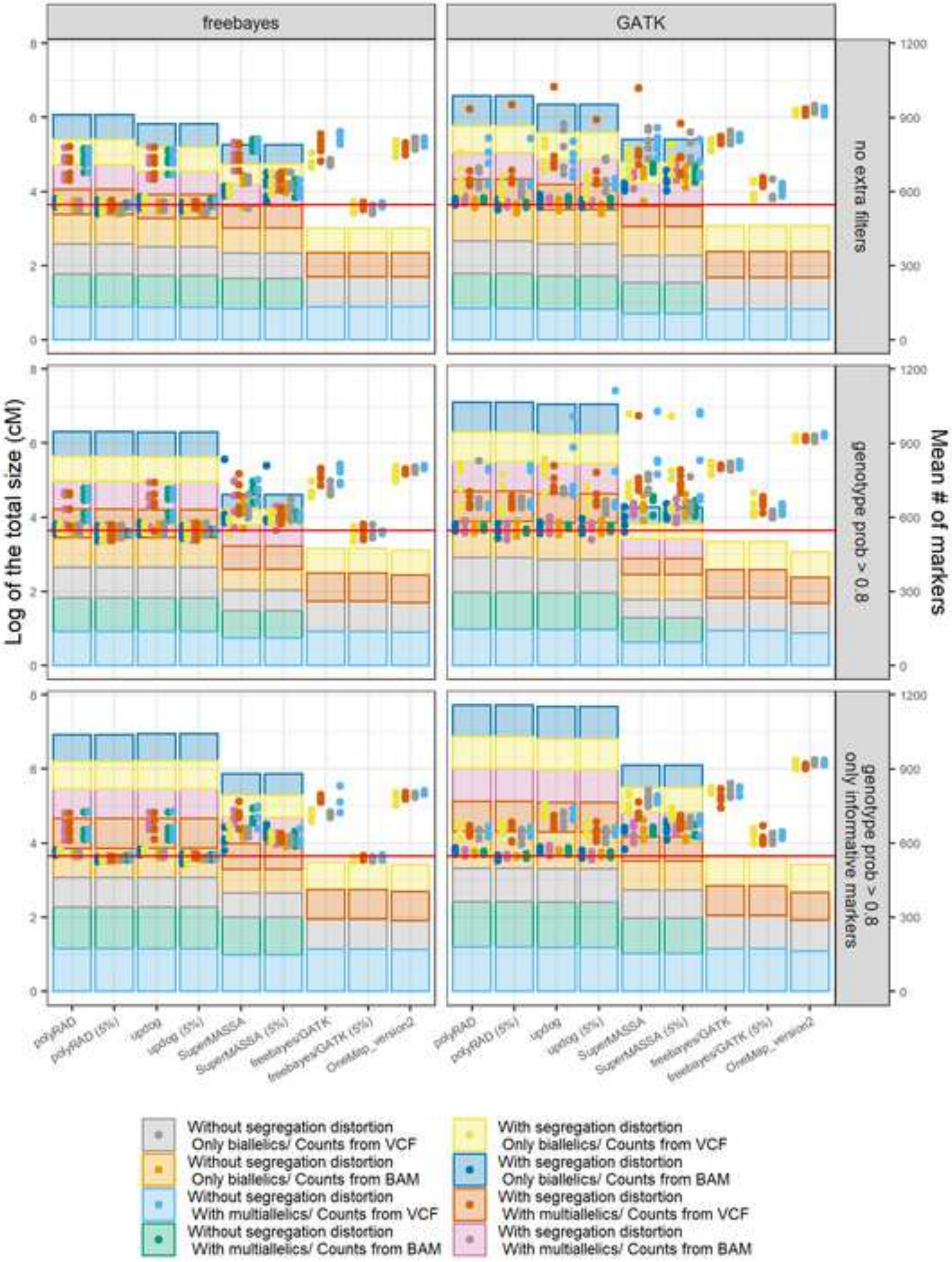
freebayes
GATK
no extra filters
genotype prob > 0.8
genotype prob > 0.8
only informative markers
Log of the total size (cM)
Mean # of markers
0
2
4
6
8
0
300
600
900
1200
polyRAD
polyRAD (5%)
updog
updog (5%)
SuperMASSA
SuperMASSA (5%)
freebayes/GATK
freebayes/GATK (5%)
OneMap_version2
Without segregation distortion
Only biallelics/ Counts from VCF
Without segregation distortion
Only biallelics/ Counts from BAM
Without segregation distortion
With multiallelics/ Counts from VCF
Without segregation distortion
With multiallelics/ Counts from BAM
With segregation distortion
Only biallelics/ Counts from VCF
With segregation distortion
Only biallelics/ Counts from BAM
With segregation distortion
With multiallelics/ Counts from VCF
With segregation distortion
With multiallelics/ Counts from BAM

 Click here to access/download;Figure;log_total_size2.png

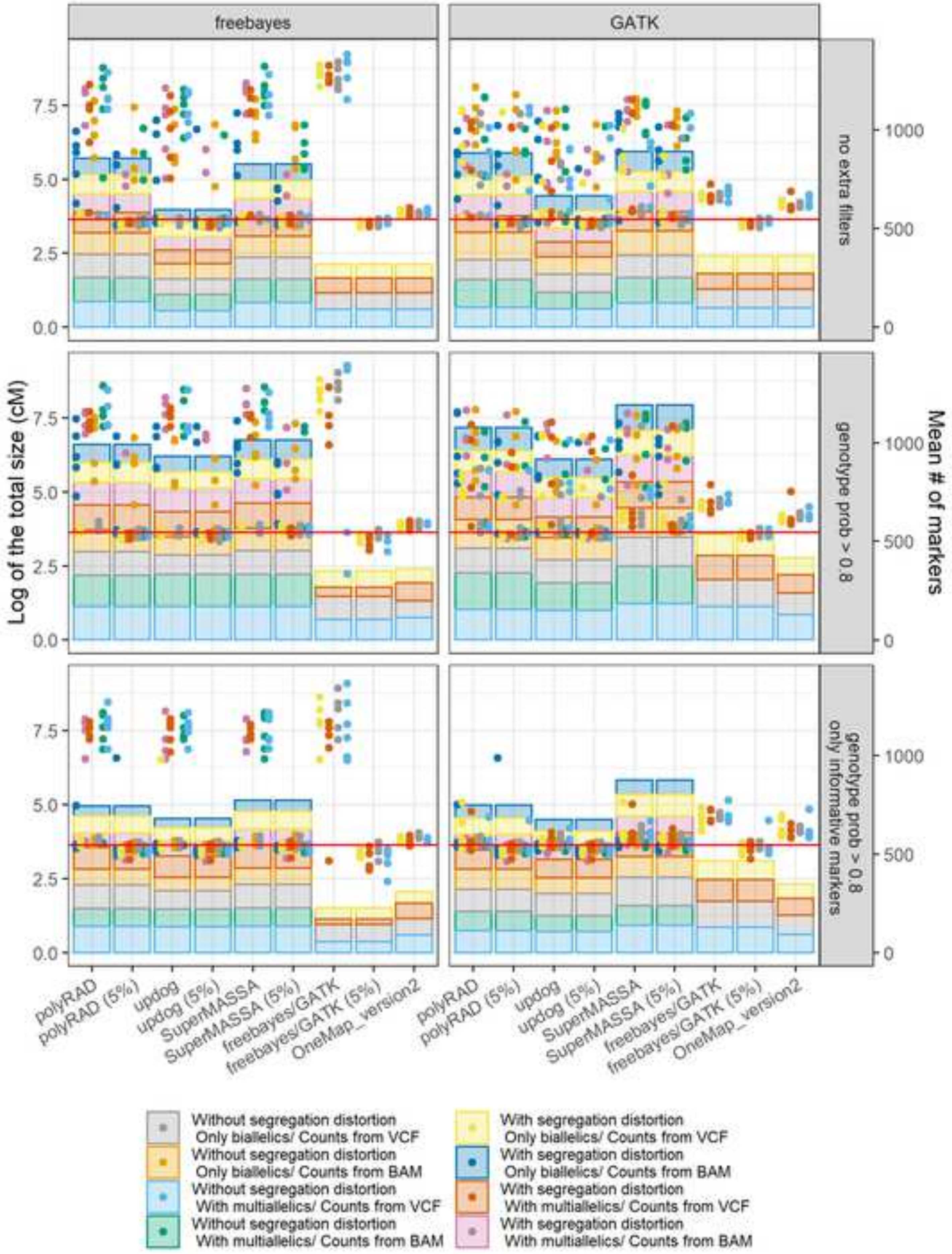

Figure
Click here to access/download;Figure;marker_type_aspen.png

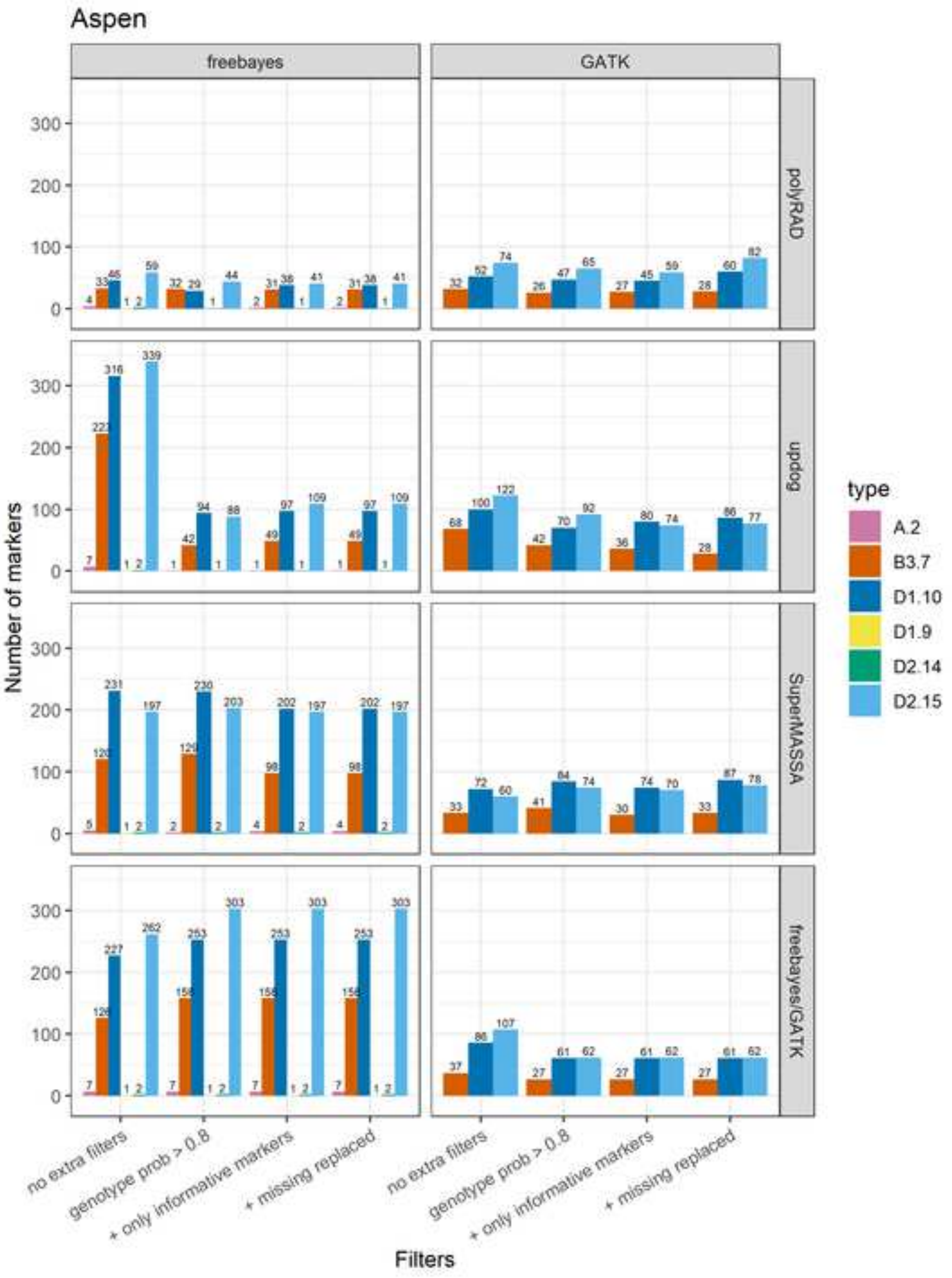

Figure

Click here to access/download;Figure;marker_type_bi_correct_paper.png

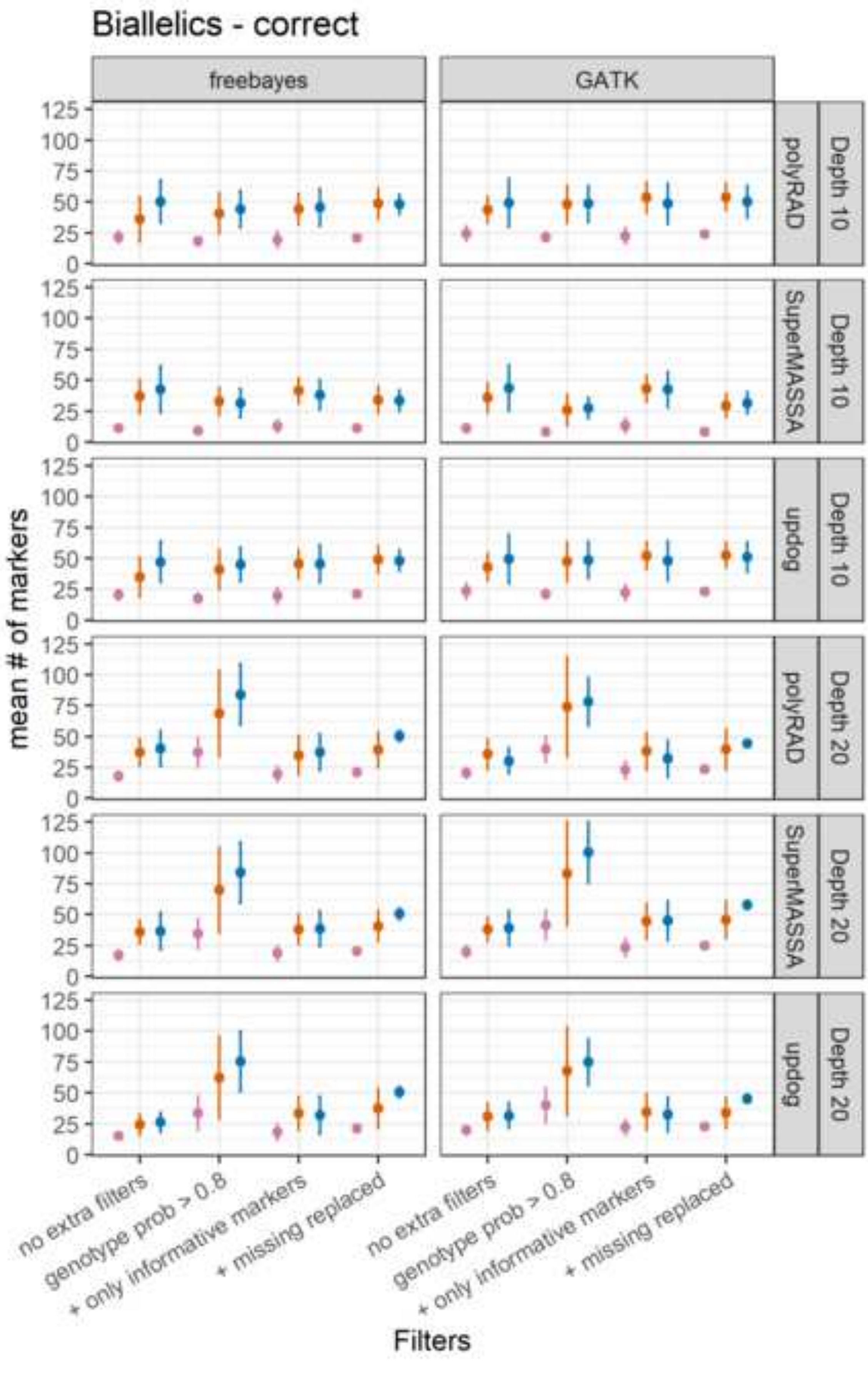

Figure

Click here to access/download;Figure;marker_type_bi_correct_suppl.png

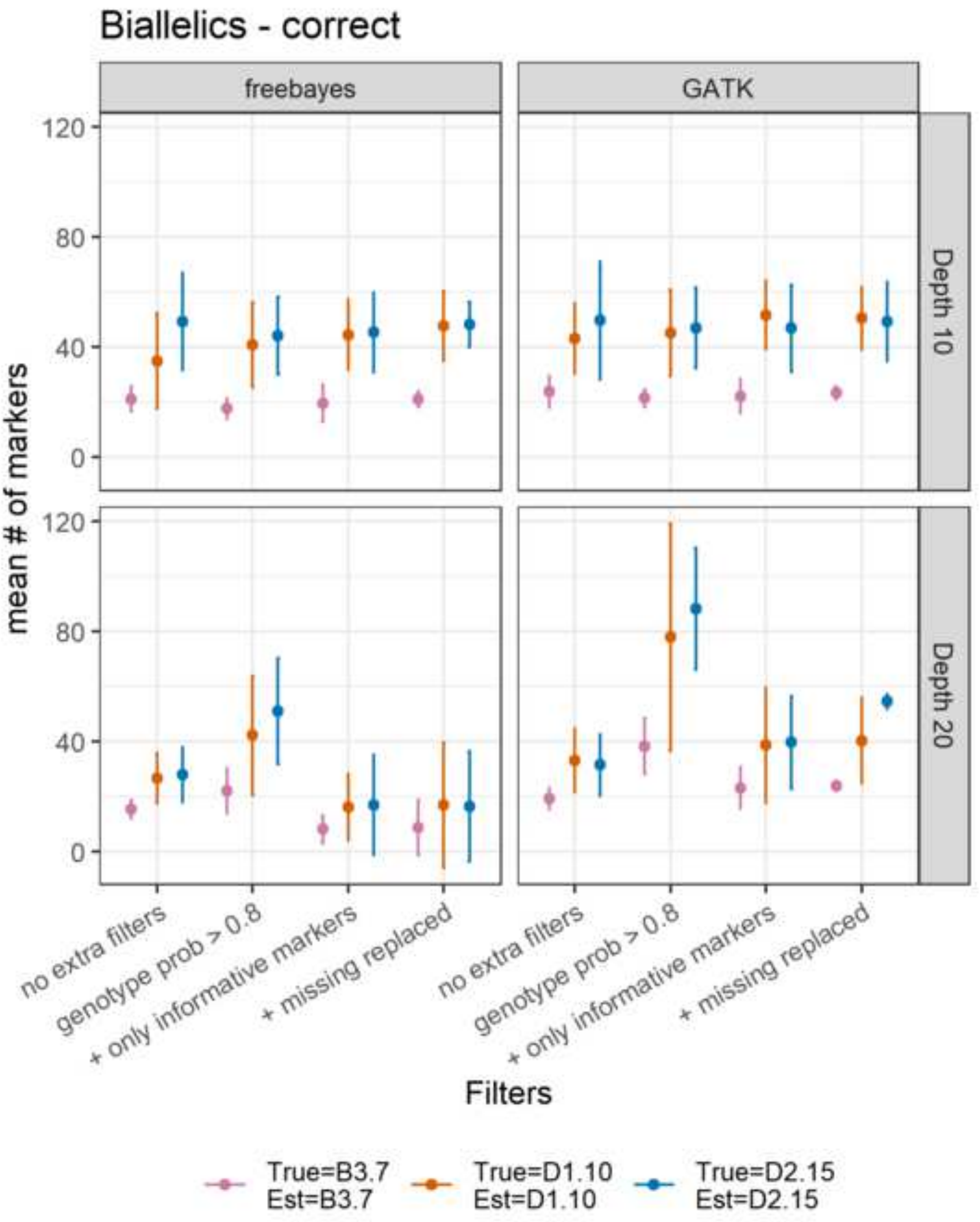

Click here to access/download;Figure;marker_type_bi_wrong_suppl.png

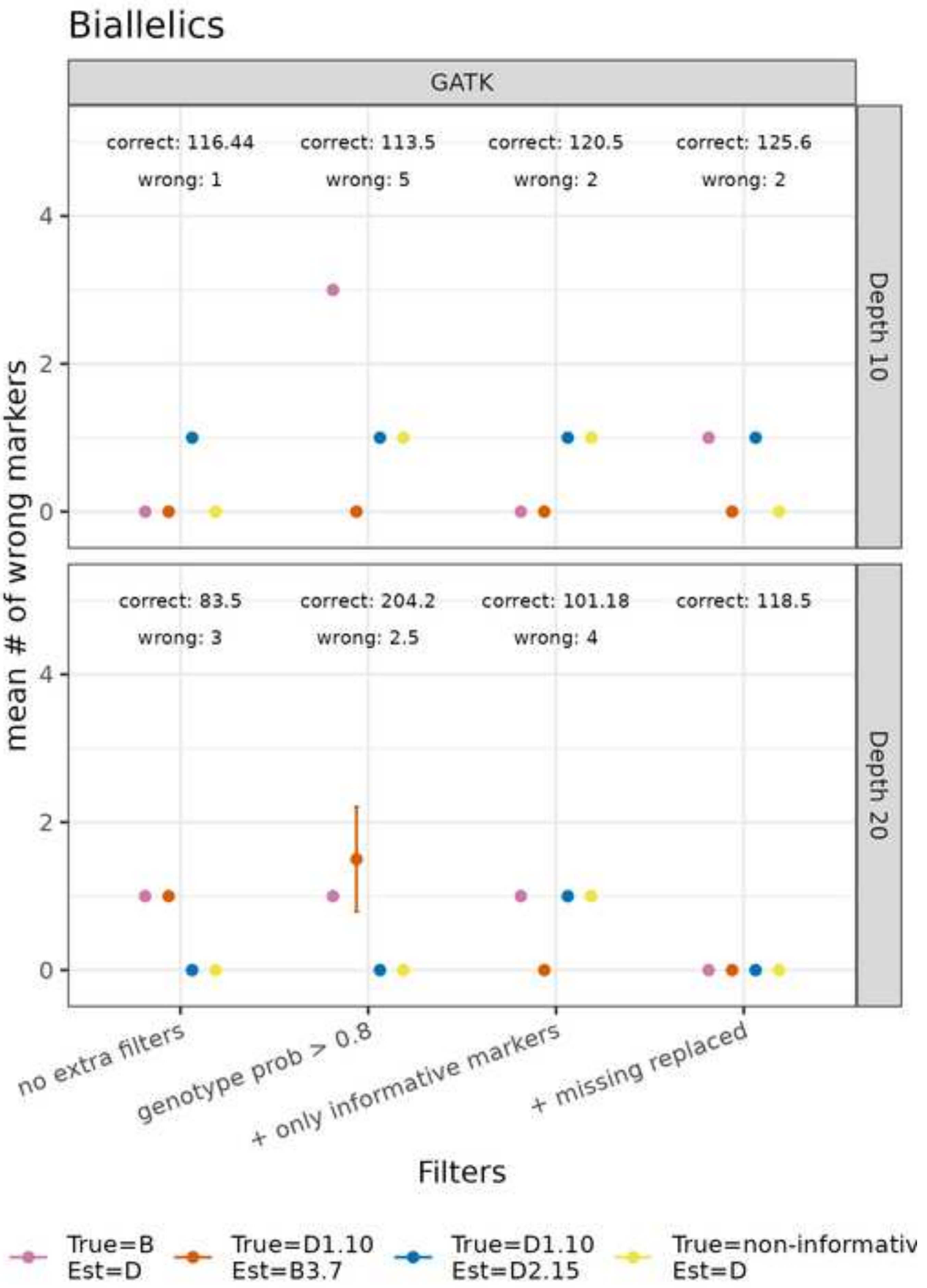

Figure

Click here to access/download;Figure;marker_type_multi_suppl.png

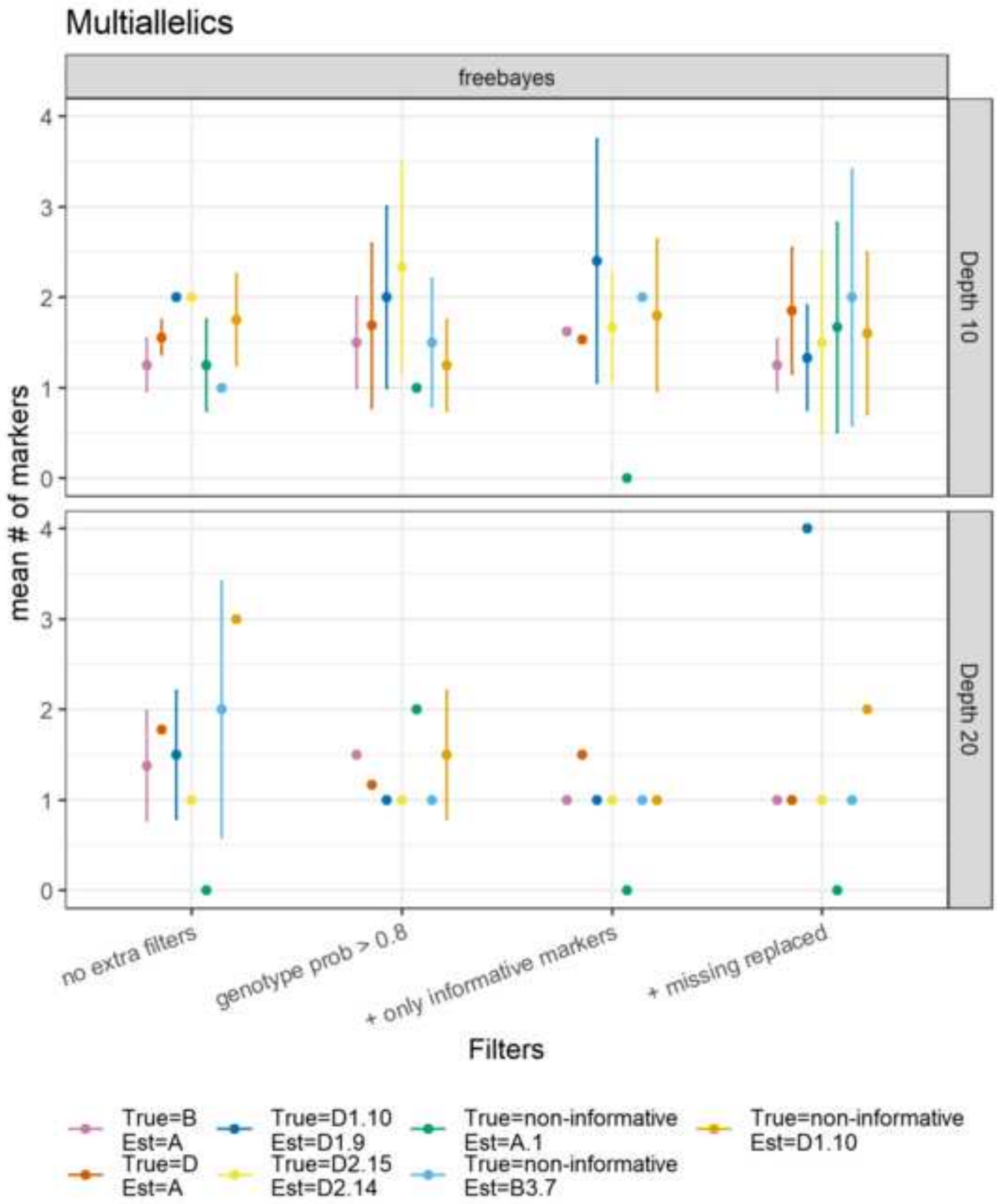

Figure

Click here to access/download;Figure;marker_type_rose.png

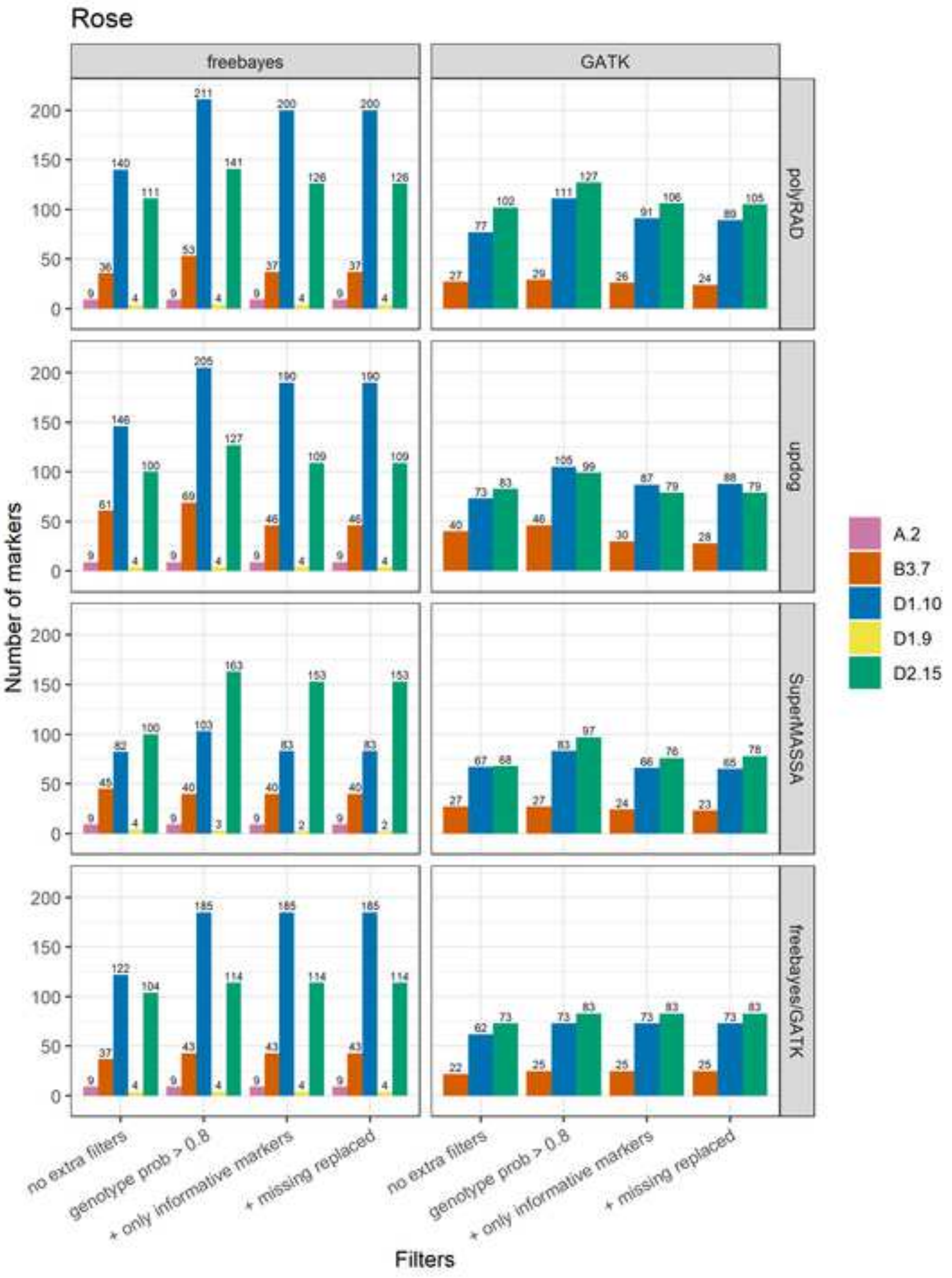

Click here to access/download;Figure;math_meth.png

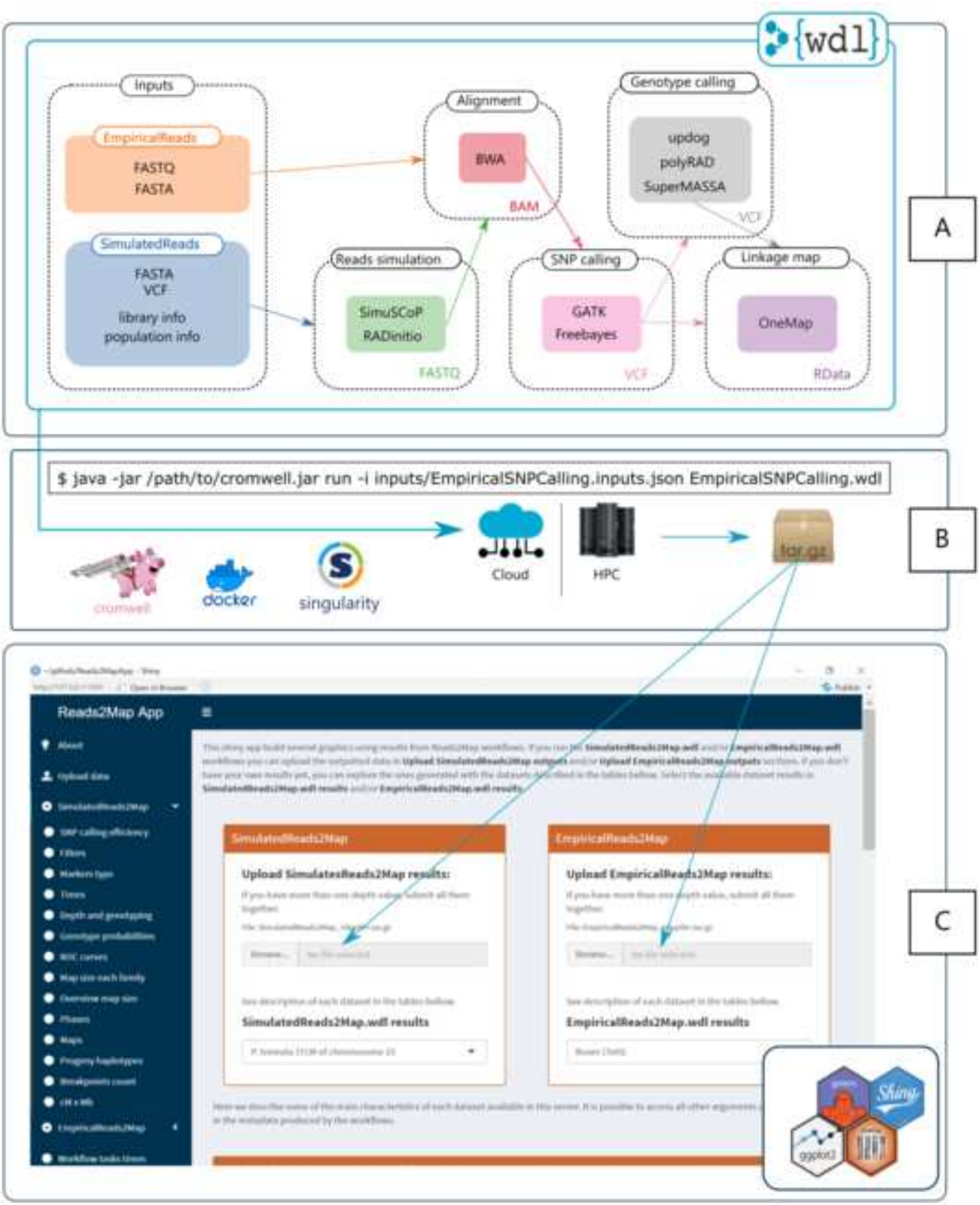

Click here to access/download;Figure;noncontxcont_paper_emp.png

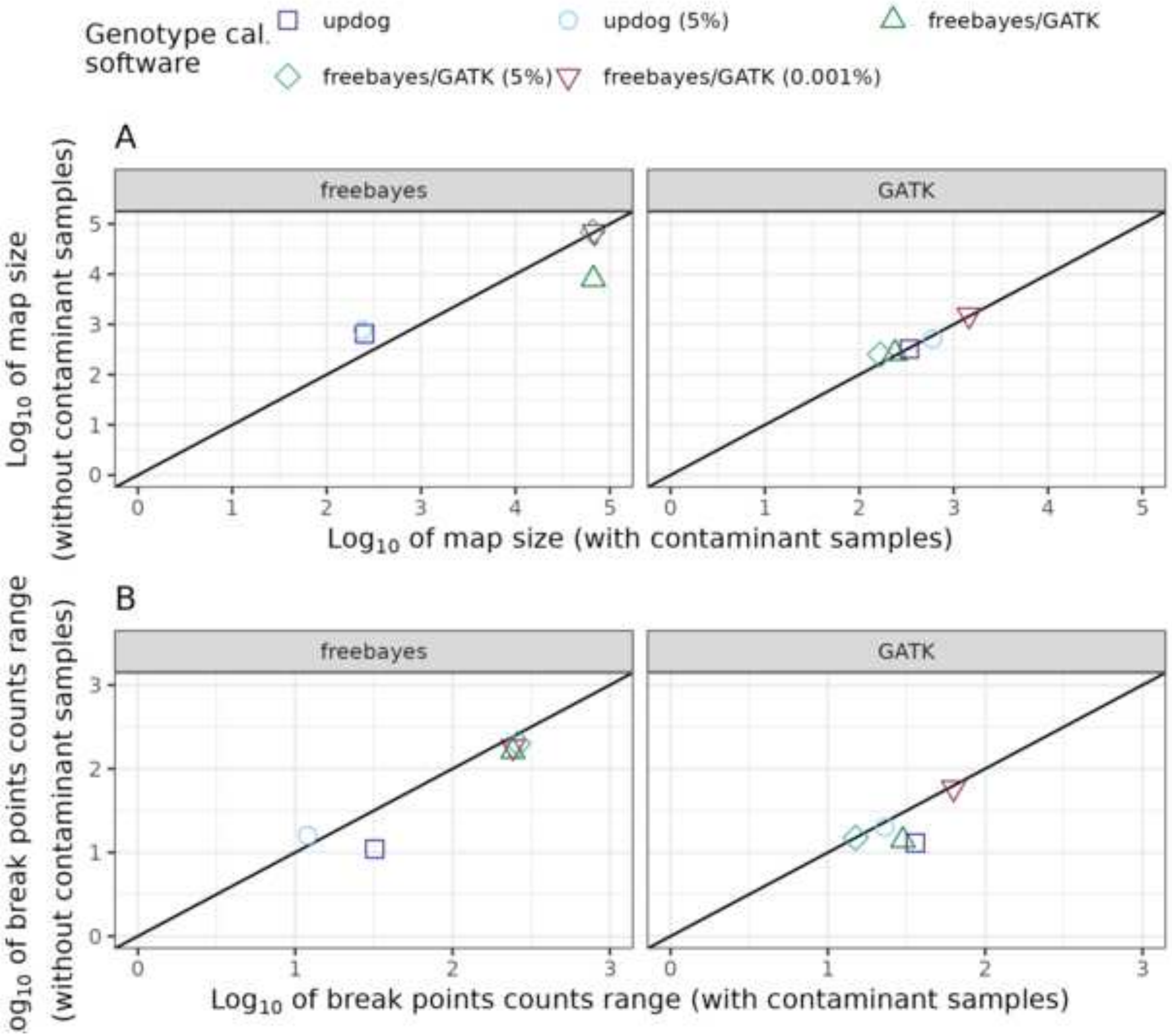

Figure

Click here to access/download;Figure;roc10.png

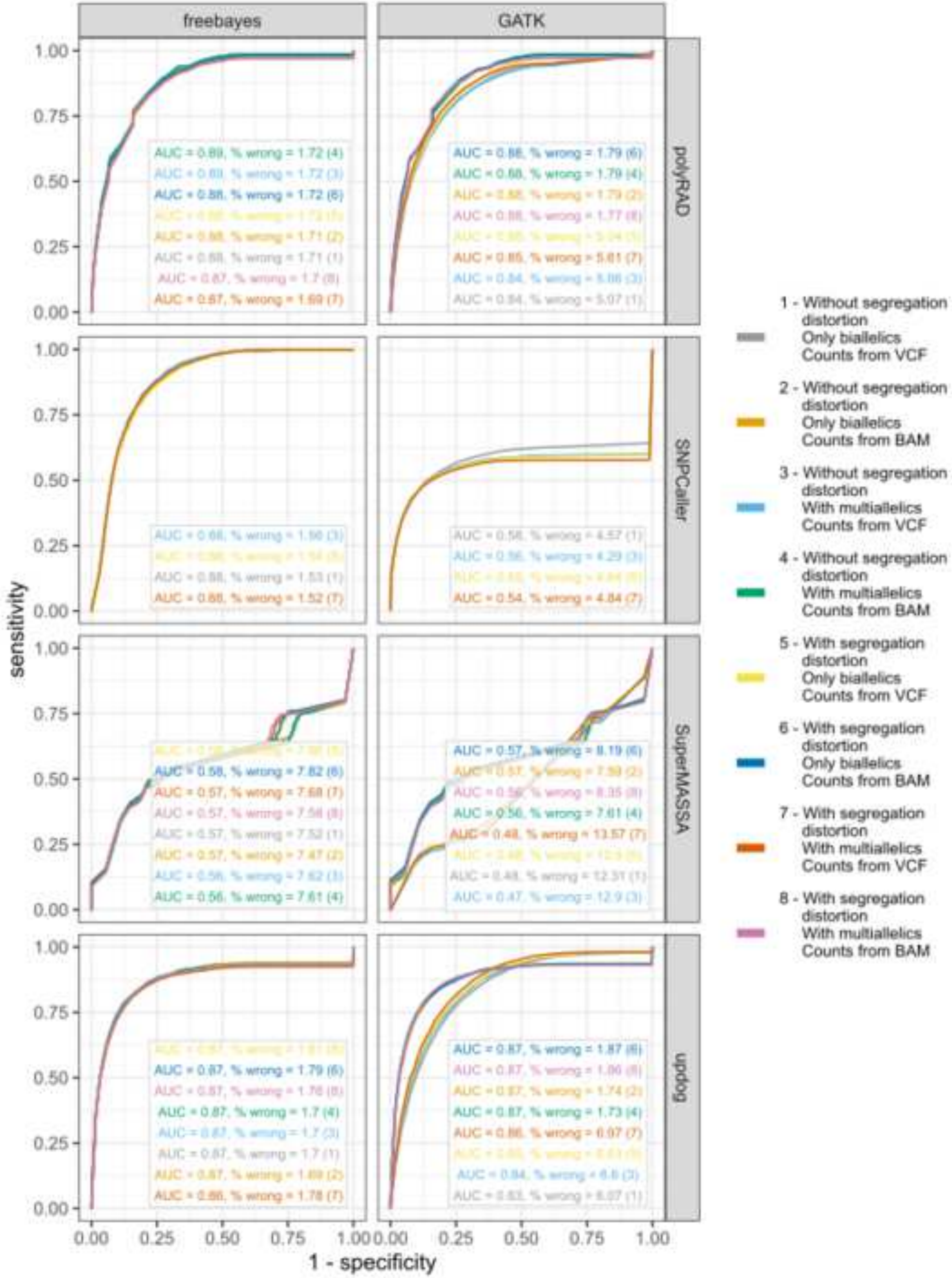

Figure

Click here to access/download;Figure;roc20.png

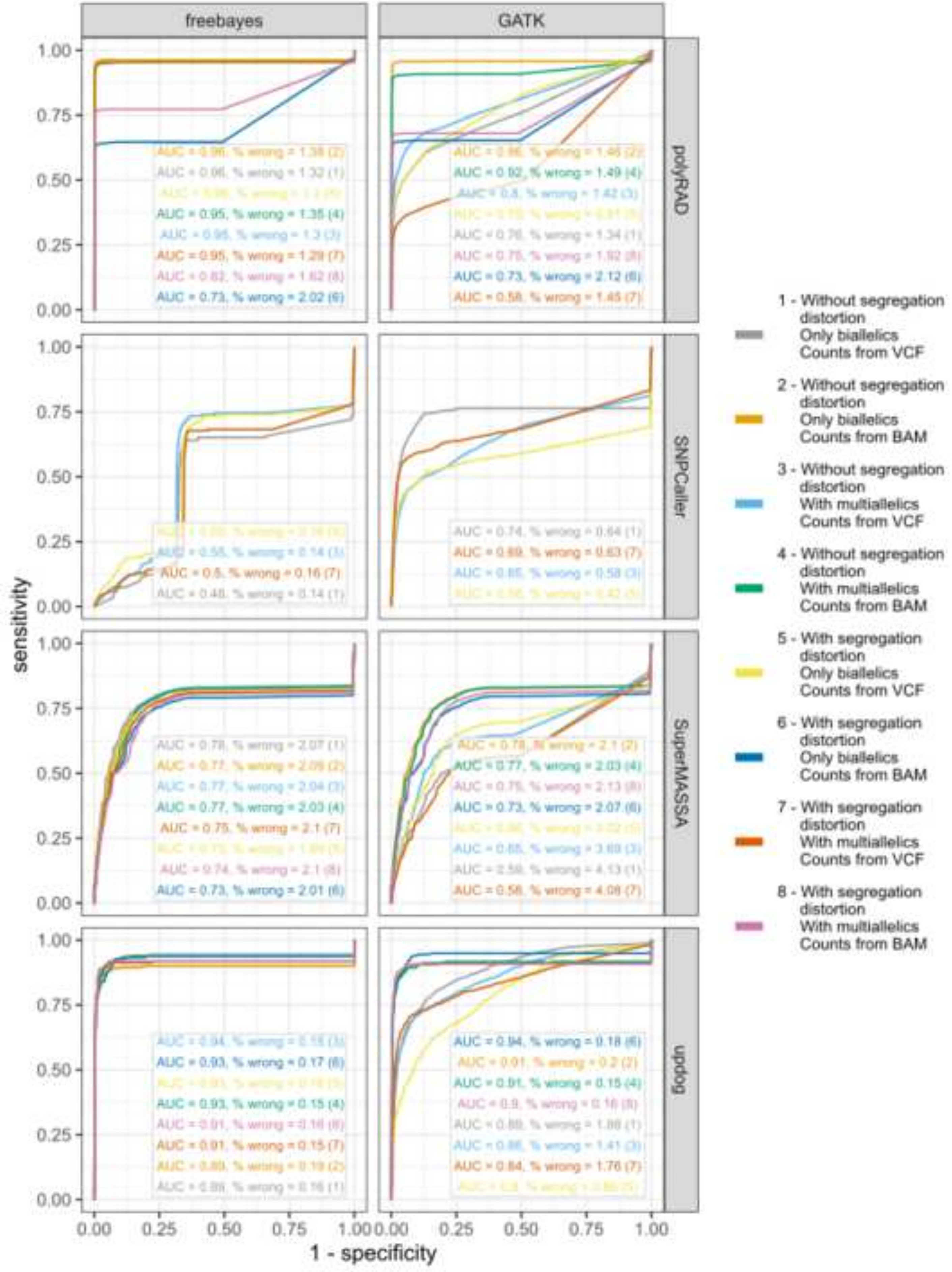

Figure

Click here to access/download;Figure;roses_pipe1.png

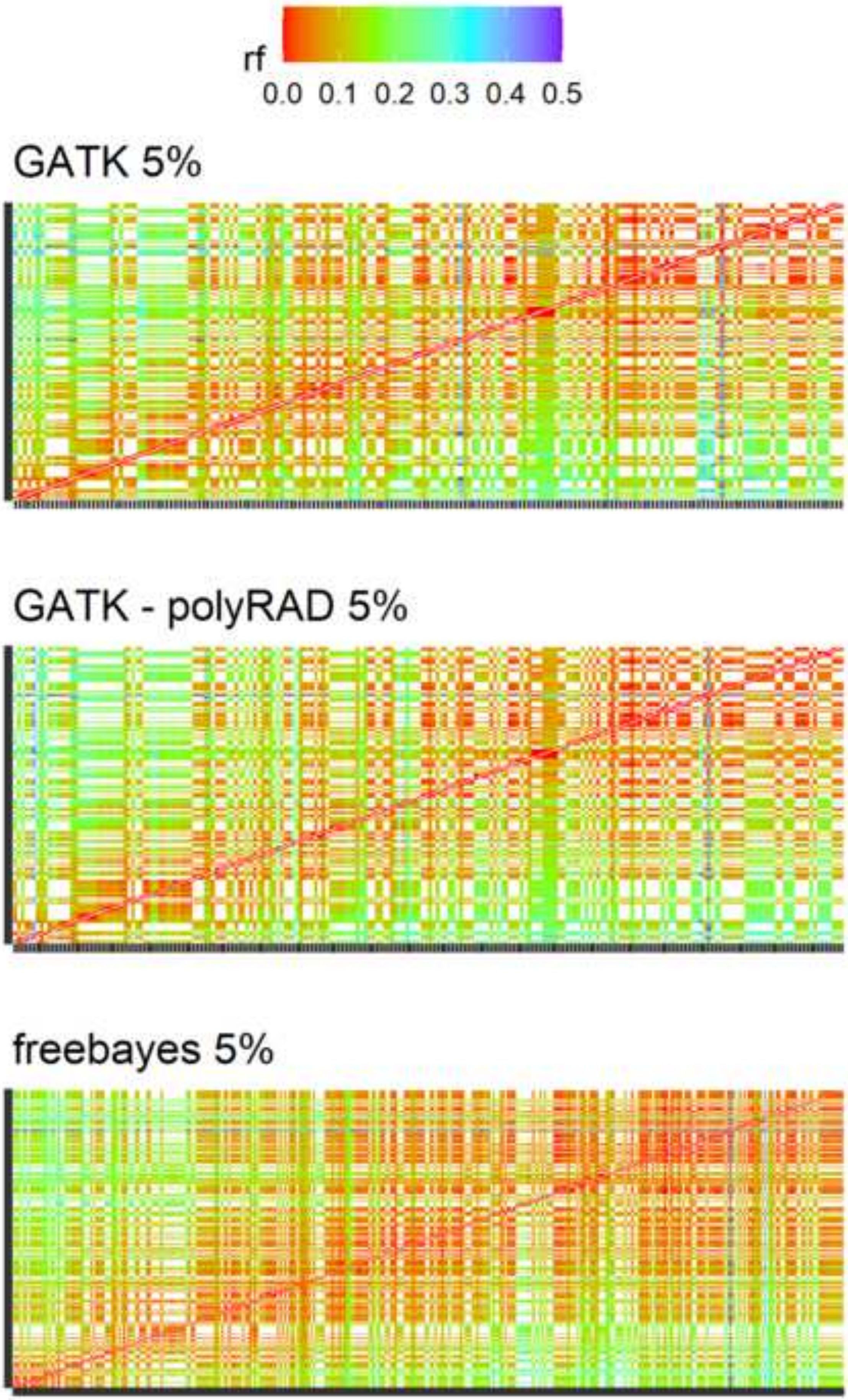

Figure

Click here to access/download;Figure;roses_pipe2.png

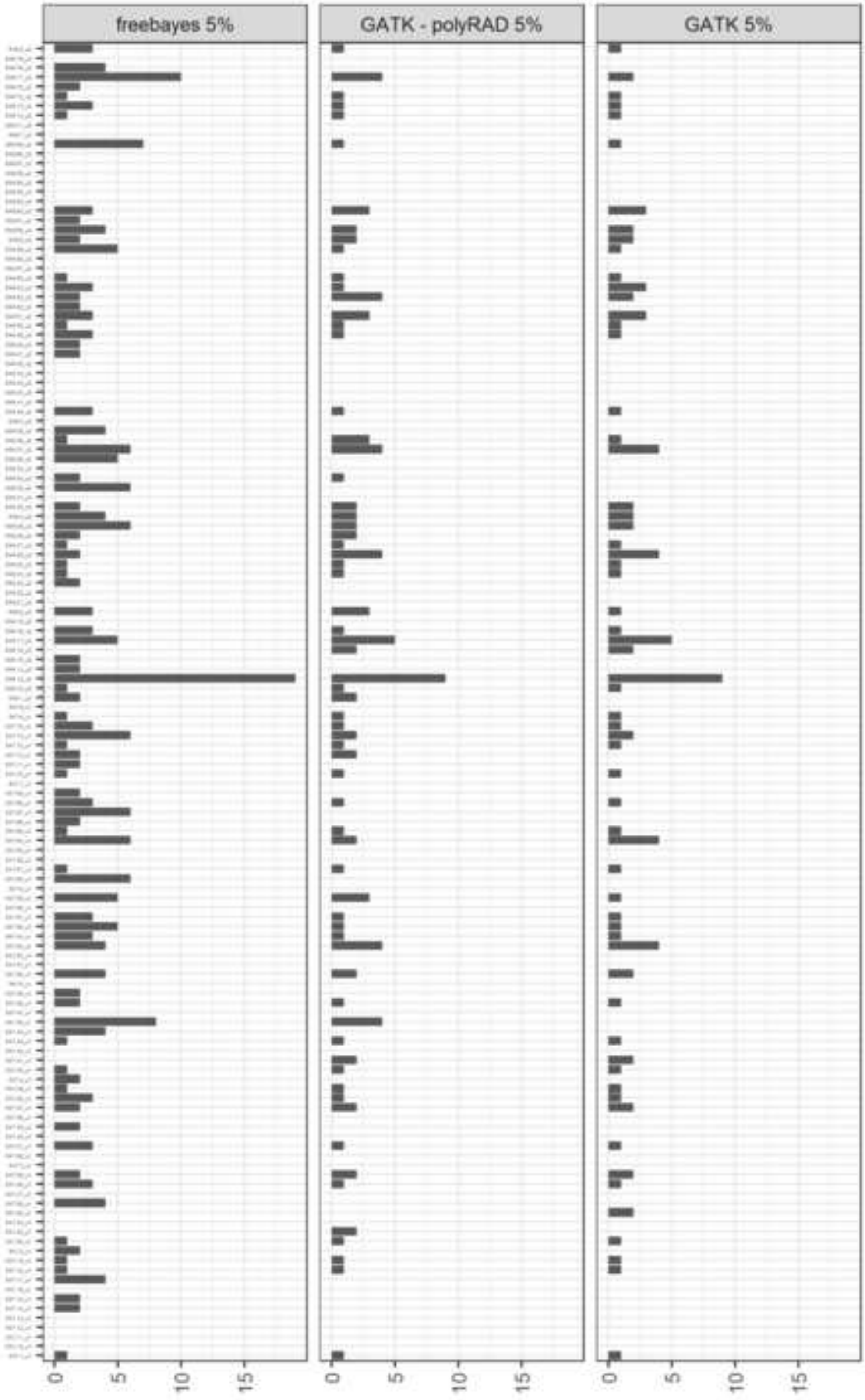

Click here to access/download;Figure;size_paper_multi.png

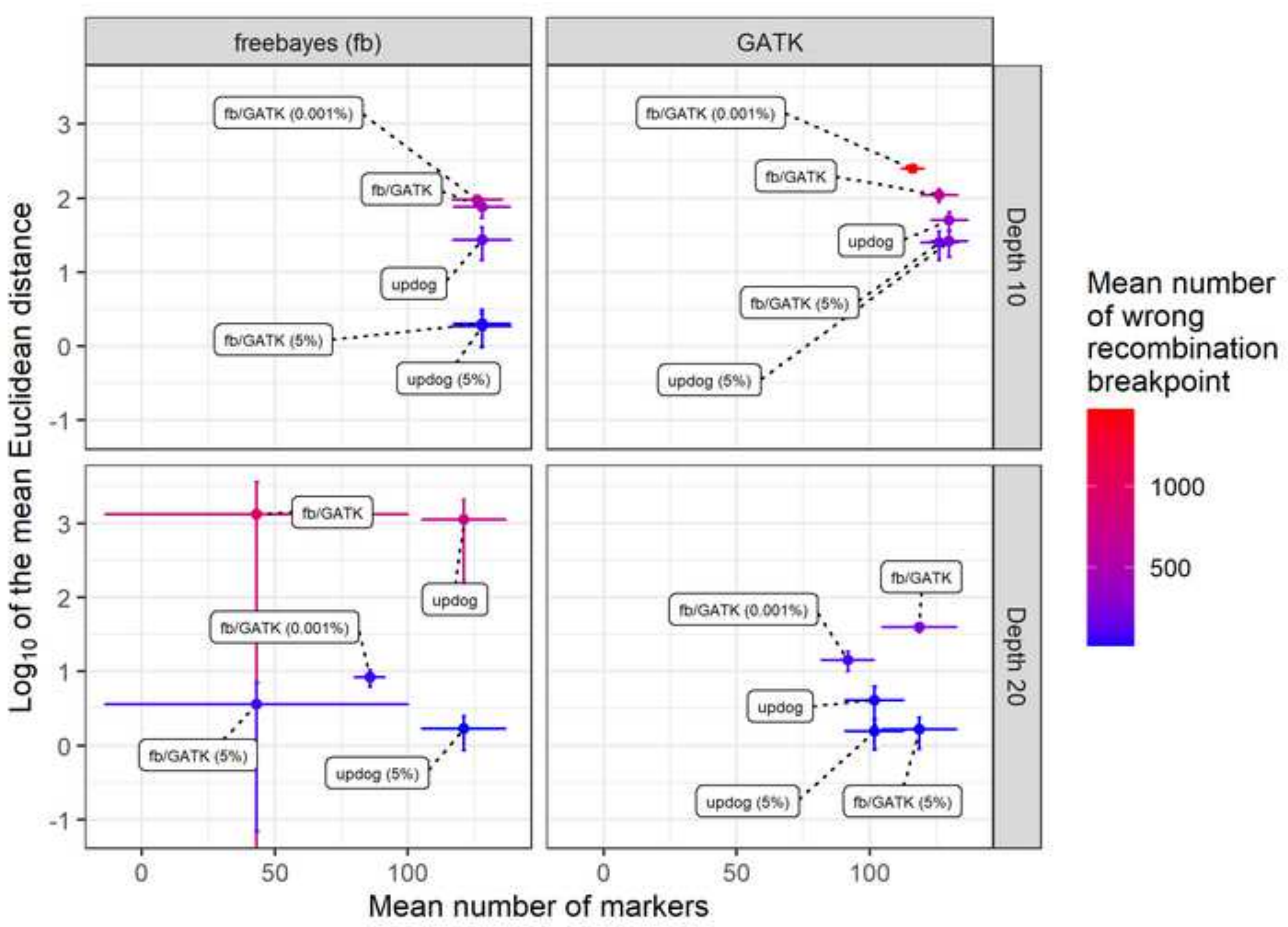

 Click here to access/download;Figure;size_suppl_multi.png

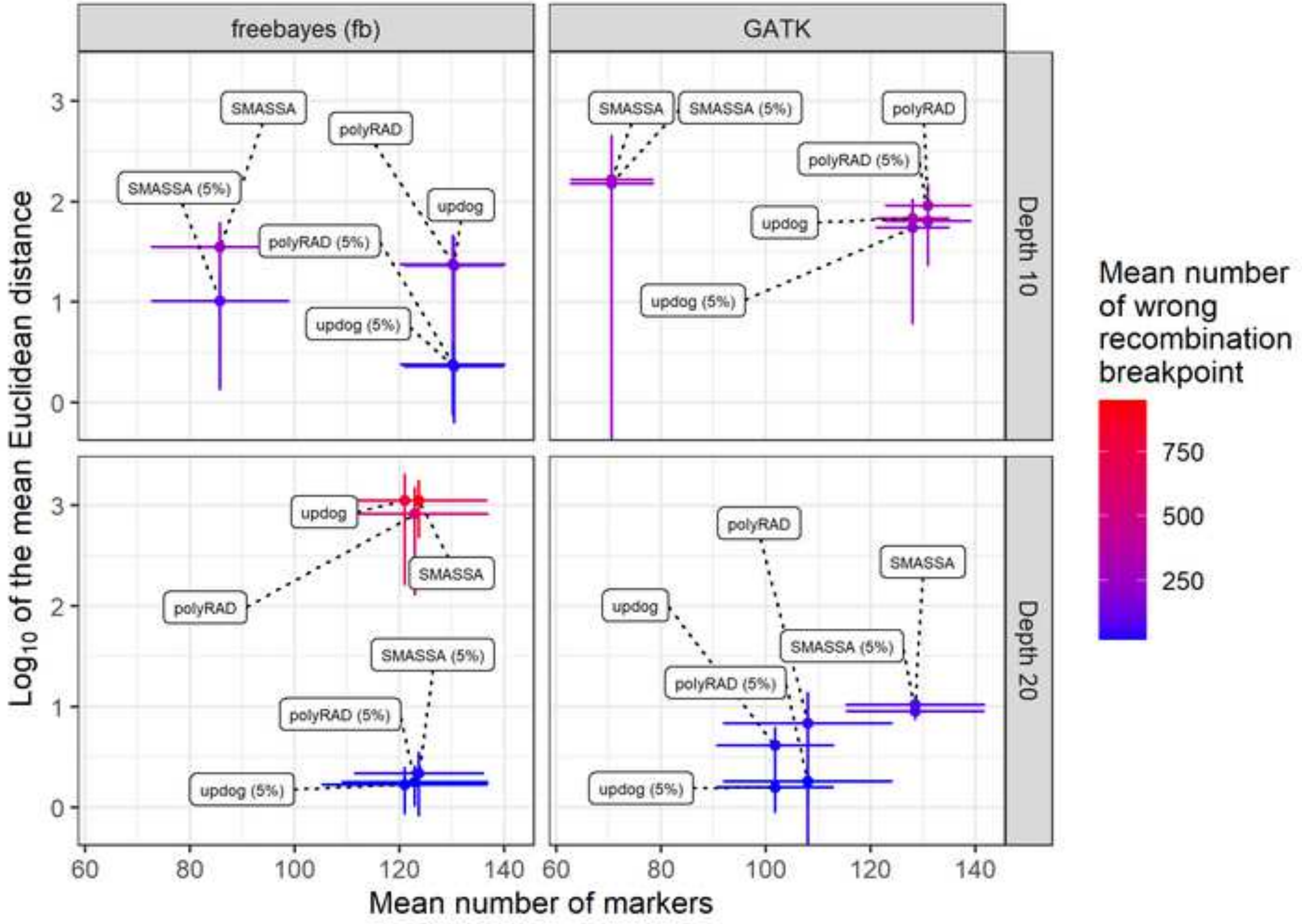

 Click here to access/download;Figure;size_supple_multi_emp.png

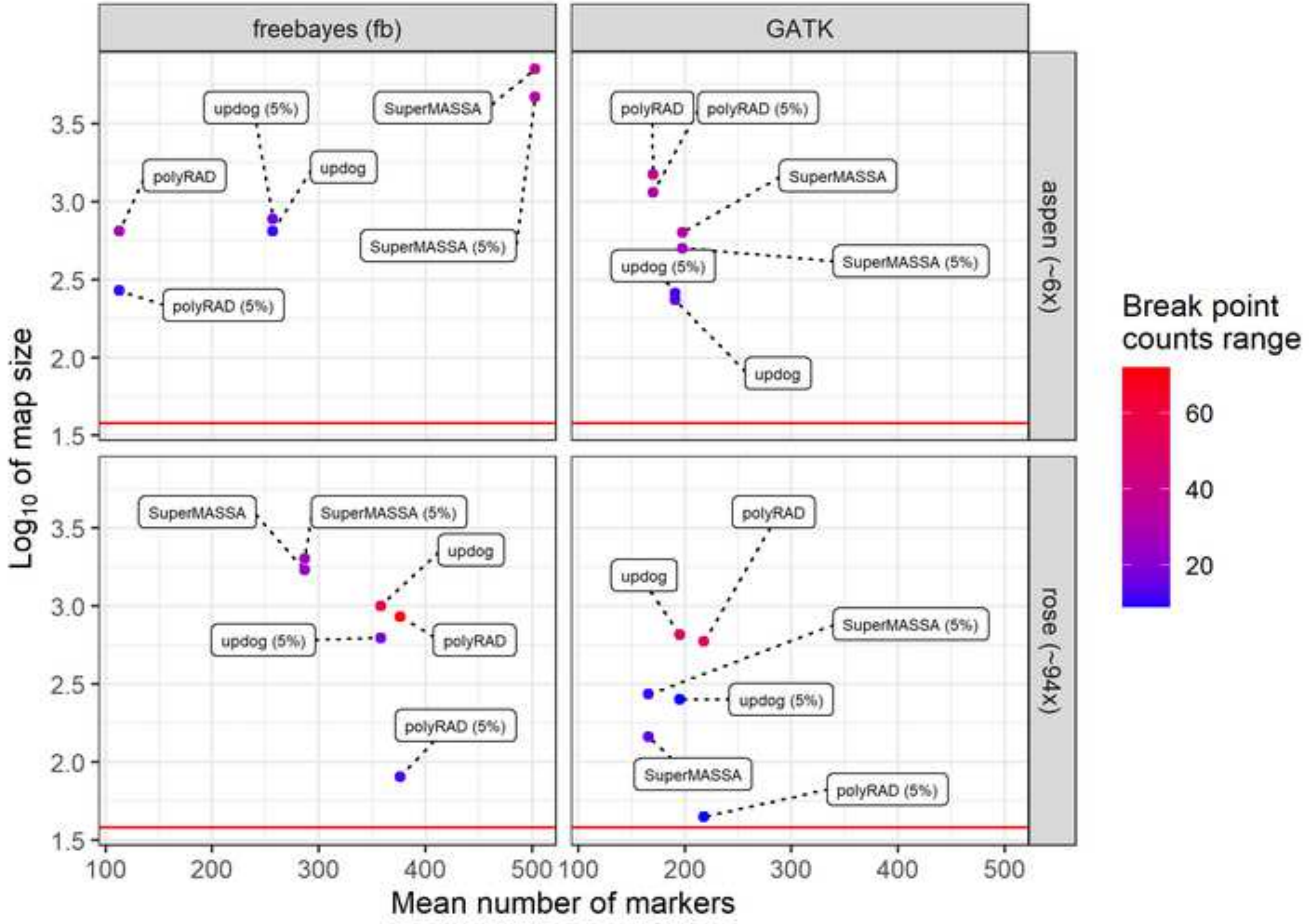

PDF Supplementary Material

Click here to access/download
Supplementary Material
Reads2Map_Supplementary_Material.pdf

Click here to access/download
Supplementary Material
Supplementary_Material.tex
