# Supplementary material for: Developing best practices for genotyping-by-sequencing analysis in the construction of linkage maps
Source: Gigascience. 2023 Oct 27;12:giad092. doi: 10.1093/gigascience/giad092 (PMC10603770; doi:10.1093/gigascience/giad092)
Supplement: giad092_GIGA-D-22-00323_Revision_1 [file giad092_giga-d-22-00323_revision_1.pdf]

GigaScience

Developing best practices for genotyping-by-sequencing analysis using linkage maps as benchmarks

--Manuscript Draft--

**Manuscript Number:**	GIGA-D-22-00323R1
**Full Title:**	Developing best practices for genotyping-by-sequencing analysis using linkage maps as benchmarks
**Article Type:**	Technical Note
**Funding Information:**	National Institute of Food and Agriculture (2020-51181-32156) — Dr David Byrne<br>Bill and Melinda Gates Foundation (OPP1213329) — Dr Marcelo Mollinari<br>HORIZON EUROPE Marie Sklodowska-Curie Actions (801215) — Dr Thiago de Paula Oliveira<br>Conselho Nacional de Desenvolvimento Científico e Tecnológico (313269/2021-1) — Dr Antonio Augusto Franco Garcia

Abstract:

Background: Genotyping-by-Sequencing (GBS) provides affordable methods for genotyping hundreds of individuals using millions of markers. However, this challenges bioinformatic procedures that must overcome possible artifacts such as the bias generated by PCR duplicates and sequencing errors. Genotyping errors lead to data that deviate from what is expected from regular meiosis. This, in turn, leads to difficulties in grouping and ordering markers resulting in inflated and incorrect linkage maps. Therefore, genotyping errors can be easily detected by linkage map quality evaluations.

Results: We developed and used the Reads2Map workflow to build linkage maps with simulated and empirical GBS data of diploid outcrossing populations. The workflows run GATK, Stacks, TASSEL, and Freebayes for SNP calling and updog, polyRAD, and SuperMASSA for genotype calling, and OneMap and GUSMap to build linkage maps. Using simulated data, we observed which genotype call software fails in identifying common errors in GBS sequencing data and proposed specific filters to better handle them. We tested whether it is possible to overcome errors in a linkage map using genotype probabilities from each software or global error rates to estimate genetic distances with an updated version of OneMap. We also evaluated the impact of segregation distortion, contaminant samples, and haplotype-based multiallelic markers in the final linkage maps. Through our evaluations, we observed that some of the approaches produce different results depending on the dataset (dataset-dependent) and others produce consistent advantageous results among them (dataset-independent).

Conclusions: We set as default in the Reads2Map workflows the approaches that showed to be dataset-independent for GBS datasets according to our results. This reduces the number required of tests to identify optimal pipelines and parameters for other empirical datasets. Using Reads2Map, users can select the pipeline and parameters that best fit their data context. The Reads2MapApp shiny app provides a graphical representation of the results to facilitate their interpretation.

**Corresponding Author:**	Cristiane Hayumi Taniguti<br>Texas A&M University<br>College Station, Texas UNITED STATES
**Corresponding Author Secondary Information:**	
**Corresponding Author's Institution:**	Texas A&M University
**Corresponding Author's Secondary Institution:**	
**First Author:**	Cristiane Hayumi Taniguti
**First Author Secondary Information:**	

Powered by Editorial Manager® and ProduXion Manager® from Aries Systems Corporation

Order of Authors:	Cristiane Hayumi Taniguti
	Lucas Mitsuo Taniguti
	Rodrigo Rampazo Amadeu
	Jeekin Lau
	Gabriel de Siqueira Gesteira
	Thiago de Paula Oliveira
	Getulio Caixeta Ferreira
	Guilherme da Silva Pereira
	David Byrne
	Marcelo Mollinari
	Oscar Riera-Lizarazu
	Antonio Augusto Franco Garcia
Order of Authors Secondary Information:	
Response to Reviewers:	Reviewer #1: I read with interest the manuscript on Reads2Map, a really impressive amount of work went into this and I congratulate the authors on it. However, it is precisely this almost excessive amount of results that for me was the major drawback with this paper. I got lost in all the detail, and therefore I have suggested a Major Revision to reflect that I think the paper could be somehow made more stream lined with a clearer central message and fewer figures in the text. Line numbers would have been helpful, I have tried to give the best indication of page number and position, but in future @GigaScience please stick to line numbers for reviewers, it's a pain in the neck without them. Overall I think this is an excellent manuscript of general interest to anyone working in genomics, and definitely worthy of publication.<br><br>Answer: Thanks for your review. I addressed the detailed comment below. To facilitate this next review, I included a version of the manuscript with line numbers.<br><br>General comment: if a user would like to use GBS data for other population types than those amenable for linkage mapping (e.g. GWAS or genomic prediction, so a diversity panel or a breeding panel), how could your tool be useful for them?<br><br>Answer: The first steps of the workflow that include the alignment with BWA and SNP calling with GATK and freebayes (now also with TASSEL and STACKS options) can be applied to any population type. Because the workflows are partitioned into sub-workflows and tasks, these steps can be run independently of the dosage calling and linkage map building which require mapping populations. We separated the EmpiricalReads2Map into EmpiricalSNPCalling and EmpiricalMaps to emphasize this difference. We also added a short explanation in the manuscript (lines 478-493).<br><br>Answer: Another way of applying the tool for non-mapping populations is if the GBS library is producing and sequencing mapping populations and non-mapping populations in the same experiment. In this situation, the results obtained for the mapping populations using Reads2Map can be extrapolated to the other populations without mapping structure.<br><br>Other general comment: the manuscript is long with an exhaustive amount of figures and supplementary materials. Does it really need to be this detailed? It appears like the authors lost the run of themselves a little bit and tried to cram everything in, and in doing so risk losing the point of the endeavour. What is the central message of this manuscript? Regarding the figures, the reader cannot refer to the figures easily as they are now mainly contained on another page. Do you really need Figures 16-18 for example? Figures 13 and 14 could be combined perhaps? I am sure that at most 10 figures and maybe even less are needed in the main text, otherwise figures will always be on different pages and hence lose their impact in the text call-out.<br><br>Answer: We reduced the text and figures.

Powered by Editorial Manager® and ProduXion Manager® from Aries Systems Corporation

Abstract and page 4: "global error rate of 0.05" - How do you motivate the use of a global error rate of 5%? Surely this is dataset-dependent?

Answer: We conduct new tests with different values and added figure number 5 to guide users on how to select a proper value. During this review, we talked to updog developer (David Gerard), who gave us the idea of combining the global error with the software genotype probability. We did that using 1-(1-genotype error probability)x(1-global error), which proved to be a good option too.

Page 4 - how can a user estimate an error per marker per individual? The description of the create_probs function suggests there is an automatic methodology to do this, but I don't see it described. You could perhaps refer to Zheng et al's software polyOrigin, which actually locally optimises the error prior per datapoint. Maybe something for the discussion.

Answer: The error probabilities used are not estimated by OneMap but by the upstream genotype calling software (VCF PL value of HaplotypeCaller, Freebayes, TASSEL, STACKs and genotype probabilities of updog, polyRAD, and SuperMASSA). The idea of doing that is to take into account issues that were found by the upstream bioinformatic process such as low depth, dispersion of the read counts, and alignment quality. Thanks for highlighting the polyOrigin method, if I understood right, it takes into account only the genotypes to estimate this error rate, it is not based on the bioinformatic features for each. We kept linkage map polyploid tools out of the scope of this work to not make it longer than already is, but we are already working to add MAPpoly as a new option to build the maps. MAPpoly contains a similar approach to control the errors as implemented in OneMap. While doing this, we can perform tests to compare with PolyOrigin approach.

Page 6 "recombination fraction giving the genomic order" do you mean "given"?
Answer: Yes. Thanks.

Page 10 section Effects of contaminant samples - if you look at Figure 9 you can see that the presence of contaminant samples seems to have an impact on the genotypes of other, non-contaminant samples, especially using GATK and 5% global error. With the contaminants present, the number of XO points decreases in many other samples. This is very odd behaviour I would have thought. Is it known whether this apparent suppresion of recombination breakpoints in non-contaminant individuals is likely to be "correct"? Perhaps the SNP caller was running under the assumption that all individuals were part of the same F1? If the SNP caller was run without this assumption (eg. specifying only HW equilibrium, or model-free) would we still see the same effect? This is for me a quite worrying result but something that you make no reference to as far as I can tell.

Answer: The GATK was not used applying an F1 assumption, but the linkage map was built considering that. The multipoint approach tries to fit the contaminant sample by redistributing the recombination breaks. This issue is emphasized while using higher values of global error because we decrease the trust in the observed genotype and increase the model assumptions. It is indeed a concerning result. We added lines 623-629 to warn users to remove contaminant samples before the linkage map building.

Page 12 "Effects of segregation distortion" In your study you only considered a single linkage group. One of the primary issues with segregation distortion in mapping is that it can lead to linkage disequilibrium between chromosomes, if selection has occurred on multiple loci. This can then lead to false linkages across linkage groups. Perhaps good to mention this.

Answer: Interesting. Added in lines 710-717 .

Page 12 "have difficulty missing linkage information" - missing word "with"

Page 17 I see no mention of the impact of errors in the multi-allelic markers on the

Powered by Editorial Manager® and ProduXion Manager® from Aries Systems Corporation

efficiency, particularly of order_seq which seems to be very poorly-performing with only bi-allelics (Fig 20). If bi-allelic SNPs have errors then it is not obvious why multi-SNP haplotypes should not also have errors.

Answer: The multiallelic markers do have errors and they have a higher effect on the estimation of the genetic distances. We updated the figure about the effects of the multiallelics now including the HMM error rate and the rose dataset. We also decide to remove order_seq algorithm evaluations because it took a long time to process and the result was not better than MDS. We updated the discussion about it in lines 630-678.

Page 3 Figure 1 - here the workflow shows multiple options for a number of the steps, which can lead to the creation of many map variants (e.g. 816 maps as mentioned on Page 4). Should all users produce 816 variants of their maps? With potentially millions of markers, this is going to take a huge amount of time (most users will want 100% of all chromosomes, not 37% of a single chromosome). Or should this be done for only a subset of markers? What if there is no reference sequence available to select a subset? As there are no clear recommendations, I suspect that the specific combination of pipeline choices will usually be dataset-dependent. You actually mention this in the discussion page 17. And with only 2 real datasets from 2 different species, there is also no way to tell if eg. GATK works best in rose, or updog should be used for monocots but not dicots etc. It would be helpful if the authors were more explicit about how their tool informs "best practices for GBS analysis" for ordinary users. Perhaps it is there, but for me this message gets lost.

Answer: We run many maps in this work to test our ideas about what could be possibly causing bad-quality linkage maps. E.g.: different upstream software, presence, and absence of multiallelic markers, contaminants, segregation distortion, and filters. Some of our conclusions we do not consider dataset dependent such as the lower performance of SuperMASSA and GUSMap (they also apparently are not being updated anymore), the usage of a filter instead of counts from BAM, usage of multiallelic markers and best filters to be applied. These were set as default in the workflows (we clarify this in lines 470-477 and Table 3). Therefore, users do not need to repeat all our tests for every dataset.

Answer: If the user wants to run using a single combination of SNP and genotype calling resulting in a single linkage map it is also possible. This can be set in the workflow input file. The need for subsetting the dataset would depend on the number of tests the user wants to perform and the computational capacity available. It is important to highlight that we did not design the workflow to be a tool to build a final linkage map but to select the bioinformatic pipeline that provides the best quality markers. The SNP and genotype calling are always made for the entire dataset. The subsetting is only required for the linkage map build step, once the HMM approach is a slow process. Once the pipeline is selected, the VCF file with markers for the entire dataset is already available for users to repeat the process in other chromosomes using the R environment and OneMap functions. We describe this suggestion of usage in lines 234-241 and lines 710-717.

Answer: In terms of software used, the results are not only dataset-dependent but also version dependent, as most of the software implemented here is still being actively developed. Although it would require more bioinformatic skills, users can also test their own hypotheses, change software versions, or include new software.

Answer: By now, having a reference genome close enough to the species to determine the markers belonging to each chromosome is required for workflow usage. This requirement was highlighted in lines 649-653.

Page 17 "updates in this version 3.0 to resolve issues with inflated genetic maps" - if I look at Figure 20, it seems that issues with inflated map length have not yet been fully resolved!

Answer: The figure was made to highlight the improvements of multiallelic markers in the ordering process, but we used the OneMap default global error to estimate the distances. We rerun the analysis using the markers resulting from the selected pipeline.

Page 17 "we provide users tools to select the best approaches" - similar comment as before - does this mean users should build > 800 maps with a subset of their dataset first, and then use this single approach for the whole dataset? It is not explicitly stated whether this is the guidance given. What is the eventual aim - to produce a good linkage map, or to use the linkage map to critically compare genotyping tools?

Answer: Reads2Map can be useful in both cases. To build a good linkage map, users need to have good quality markers, and selecting the bioinformatic approach which provides them is essential. As mentioned above, Reads2Map was not designed to directly build a final linkage map but to select the pipeline. The total number of maps generated by it will depend on the tests users wants to make. Currently, using the default parameters, the workflow will generate 12 maps for the user-defined subset.

Answer: With the goal of comparing genotyping tools, Reads2Map is also useful for developers to validate updates, because it facilitates checking the consequences of the changes in the quality of the markers by easily controlling versions, rerunning datasets, and checking the map quality (added in lines 725-731). One example of it is that during this review, updog developer implemented a new method to try to overcome the updog issues identified in this work. We re-run some of our tests to give him feedback on the update impact (see the GitHub issue for details: https://github.com/dcgerard/updog/issues/19).

Reviewer #2: The paper titled "Developing best practices for genotyping-by-sequencing analysis using linkage maps as benchmarks" aims to present an end to end workflow uses GBS genotyping datasets to generate genetic linkage maps. This is a valuable tool for geneticists intending to generate a high confidence linkage map from a mapping population with GBS data as input.

I got confused on reading the MS though, is this a workflow paper or is this a review of the component software for each step of genetic mapping and how parameter/use differences affect the output ? If it's a review, then the choice of software reviewed are not comprehensive enough, esp on SNP calling, and linkage mapping.

Answer: The idea is not to do a review but to provide tools and guidelines for building a good quality linkage map in different situations. We changed the text to streamline our findings according to our tests and we set defaults in the workflow to reduce the number of required tests by users.

There is no clear justification why each component software was used,example the use of GATK and freebayes for SNP calling I am familiar with using TASSEL GBS and STACKS for SNP calling using GBS data, why weren't they included in the SNP calling software.

Answer: We agree. We implemented both software for the SNP calling and perform new tests in empirical datasets. We updated the text to include the results from them.

The MS would benefit greatly from including these SNP calling software in their benchmarking.Onemap and gusmap seems also pre-selected for linkage mapping, without reason for use, or maybe the reason(s) were not highlighted in the text. I've had experience in the venerable MAPMAKER and MSTMap, and would like to see more comparisons of the chosen genetic linkage mapping software with others, if this is the intent of the MS.

Answer: MAPMAKER and MSTMap as well as ASMap are not able to build linkage maps for the highly heterozygous populations (full-sib or outcrossing populations) evaluated in this work. Other software such as Lep-MAP and JoinMap are able to build linkage maps for outcrossing but do not present a method to account for aspects of the genotype calling (e.g. read depth distribution) in their genetic distance estimation such GUSMap and OneMap do. Also, JoinMap is not open-access.

The MS also clearly focuses on genetic linkage mapping using GBS, which should be more explicitly stated in the title. GBS is also extensively used in diversity collections

Powered by Editorial Manager® and ProduXion Manager® from Aries Systems Corporation

and there is scant mention of this in the MS, and whether the workflow could be adapted to such populations.

Answer: Similar to the first answer given to reviewer #1. We added an explanation about how Reads2Map can be applied to other sequencing library types in lines 478-493.

Versions of software used in the workflow are also not explicitly stated within the MS.

Answer: Because there are many used software and libraries versions, we described in the Reads2Map repository README only the docker images used and their versions. Some of the used images are available online and the Dockerfile describing the software and the versions that they contain can be found in their repository. Other images used were built by us and the Dockerfile for them can be found in their DockerHub repository or in the Reads2Map GitHub repository (directory .dockerfiles). The image used by each task of the workflow is indicated in the WDL runtime. We added Table 8 in the Supplementary Material with a list of docker images version used for the results presented.

The shiny app is also not demonstrated well in the MS, it could be presented better with screenshots of the interface, with one or two sample use cases.

Answer: We clarify in the figures caption that they were obtained through the app and we also added screenshots in Supplementary File 2.

Reviewer #3: In this MS, the authors tried to develop a framework for using GBS data for downstream analysis and reduce the impact of sequence errors caused by GBS. However, sequence error is an issue not specific to GBS, it is also for whole genome sequences. Actually, I think the major issue for GBS is the missing data. However, in this MS, the authors did not test the impact of missing data on downstream analysis.

Answer: The work does not focus only on sequence errors but on genotype errors which can be caused also by other sources (e.g. such as low depth, PCR bias) including missing data. The software used to simulate sequence reads (RADinitio) also simulates missing data. The higher the read depth set for the software, the lower will be the rate of missing data once the chance of sequencing common loci between all samples increases. RADinitio does not have a specific parameter for controlling the missing data rate but it is proportional to the read depth parameter. This relation (read depth x missing data) was also observed in the empirical data evaluated. The rose data set has a smaller percentage of missing data compared to the aspen data. In this pipeline, the amount of missing data has a higher effect on the number of markers used to build the linkage map than the genotyping error, once we filter the markers with a maximum of 25% of missing data before starting the linkage map building. The HMM method used to estimate the genetic distances have the capacity to input the missing data, but high percentages of it can demand more time to process. The correct imputation of the missing data will depend on the correct information of the given genotypes.

Answer: We highlight in the text that the PCR bias and the duplicates can generate more genotyping errors in GBS data compared to other library types such as whole genome and exome sequencing. The bias changes the proportion of alleles in heterozygous individuals and can lead to wrong estimations of true heterozygous genotypes as homozygous. Also, differently from other technologies, the GBS data is composed basically of duplicates (sequences that start and end in the same position, the cut sites). This makes it impossible to distinguish optical duplicates and sequencing artifacts. The non-removal of the optical duplicates can lead to the wrong estimation of homozygous genotypes as heterozygous.

Answer: Other library types can also be evaluated in Reads2Map. In the

Powered by Editorial Manager® and ProduXion Manager® from Aries Systems Corporation

EmpiricalSNPCalling sub-workflow, the single difference would be to set the parameter to remove duplicates as TRUE (lines 478-493).

The authors also mentioned that sequencing error may cause distortion segregation in linkage map construction, however, distortion segregation in linkage map construction can also happen for correct genotyping data. The distortion segregation can be caused by individual selection during the construction of the population. So I don't think it is correct to use distortion segregation to correct sequence errors.

Answer: We can think about the effect of segregation distortion in two different steps of the pipeline. The first is in the genotype calling and the second is in the linkage map. The genotype/dosage calling software updog, polyRAD, and SuperMASSA use the population expected segregation as prior to calling the genotypes. Their work highlights the advantages of doing it. With our simulations, we tested how much their estimation would be affected in the presence of true segregation distortion (Supplementary figure 9 and 10), which reveal a slightly lower efficiency.

Answer: In the linkage map step, the presence of true segregation distortion should not affect the linkage map building. However, at first, it is not possible to distinguish between markers with segregation distortion caused by genotyping errors and markers with biologically explained segregation distortion. We adopt the strategy of being restrictive at the beginning and filter all markers presenting segregation distortion to avoid higher map inflation. Once the pipeline is selected and the linkage map main structure is built, we can recover the discarded markers and insert them using the TRY algorithm. At this point, we will be able to check in the recombination fraction matrix plot which distorted markers fit the linkage group (true segregation distortion) and which do not.

The authors need to clear the major question of this MS, in the abstract, the authors highlight the sequence errors, while in the introduction, the authors highlight the package for linkage map construction (the last paragraph). Actually, from the MS, authors were assembling a framework for genotyping-by-sequencing data.

Answer: The same was suggested by the other reviewers. We adapted the text to highlight the goal of Reads2Map as a tool to select bioinformatic pipelines previously to the linkage map building.

Two major reduced-represented sequencing approaches, GBS and RADseq, have specific tools for genotype calling, such as Tassel and Stack. However, the authors used the GATK and Freebayes pipeline for variant calling, authors need to present the reason they were not using TASSEL and Stack.

Answer: We implemented TASSEL and Stacks, made new tests, and updated the text accordingly.

In the genotyping-by-sequencing data, individuals were barcoded and mixed during sequencing, what package/code was used to split the individuals (demultiplex) from the fastq for GATK and Freebayes pipeline?

Answer: We used the STACKs plugin process_radtags for that. This is not included in the main workflows because we think the sequences need to be evaluated through FASTQC and the filtering steps need to be made accordingly before starting the SNP calling. They will variate a lot depending on the library type and technology used. The

	Reads2Map workflows require already filtered and demultiplexed FASTQ files. But we provided a suggestion on how to do that in the Preprocess.wdl workflow which is also available in the GitHub repository.<br><br>The maximum missing data was allowed at 25% for markers data, how about for the individual missing rate?<br><br>Answer: Our strategy keeps all individuals even if some of them have a higher percentage of missing data to account for as many as possible recombination events in the population. As mentioned before, the HMM has the capacity to impute the missing data.<br><br>On page 6, the authors mentioned 'seuqnece size of 350', what that means?<br><br>Answer: This refers to the RADinitio parameter –insert-mean. We changed the text to make it clearer.
**Additional Information:**	
**Question**	**Response**
Are you submitting this manuscript to a special series or article collection?	No
**Experimental design and statistics**<br><br>Full details of the experimental design and statistical methods used should be given in the Methods section, as detailed in our Minimum Standards Reporting Checklist. Information essential to interpreting the data presented should be made available in the figure legends.<br><br>Have you included all the information requested in your manuscript?	Yes
**Resources**<br><br>A description of all resources used, including antibodies, cell lines, animals and software tools, with enough information to allow them to be uniquely identified, should be included in the Methods section. Authors are strongly encouraged to cite Research Resource Identifiers (RRIDs) for antibodies, model organisms and tools, where possible.<br><br>Have you included the information requested as detailed in our Minimum	Yes

Standards Reporting Checklist?	
**Availability of data and materials**<br><br>All datasets and code on which the conclusions of the paper rely must be either included in your submission or deposited in publicly available repositories (where available and ethically appropriate), referencing such data using a unique identifier in the references and in the "Availability of Data and Materials" section of your manuscript.<br><br>Have you have met the above requirement as detailed in our Minimum Standards Reporting Checklist?	Yes

Powered by Editorial Manager® and ProduXion Manager® from Aries Systems Corporation

main in latex template Click here to access/download;Manuscript;main.tex

Placeholder for OUP logo oup.pdf

Placeholder for journal logo gigascience-logo.pdf

GigaScience, 2017, 1–19

doi: xx.xxxx/xxxx
Manuscript in Preparation

Developing best practices for genotyping-by-sequencing analysis using linkage maps as benchmarks

Cristiane Hayumi Taniguti[1,2,*], Lucas Mitsuo Taniguti[1,3], Rodrigo Rampazo Amadeu[1], Jeekin Lau[2], Gabriel de Siqueira Gesteira[1,4], Thiago de Paula Oliveira[5], Getulio Caixeta Ferreira[1], Guilherme da Silva Pereira[7], David Byrne[2], Marcelo Mollinari[4], Oscar Riera-Lizarazu[2] and Antonio Augusto Franco Garcia[1,*]

[1]Department of Genetics, University of São Paulo, Brazil and [2]Department of Horticultural Sciences, Texas A&M University, College Station, TX, USA and [3]Mendelics Genomic Analysis, São Paulo, Brazil and [4]Bioinformatics Research Center, Department of Horticultural Sciences, North Carolina State University, Raleigh, NC, USA and [5]Roslin Institute, University of Edinburgh, Scotland and [7]Department of Agronomy, Federal University of Viçosa, Brazil

[*]chtaniguti@tamu.edu; augusto.garcia@usp.br

Abstract

Background Genotyping-by-Sequencing (GBS) provides affordable methods for genotyping hundreds of individuals using millions of markers. However, this challenges bioinformatic procedures that must overcome possible artifacts such as the bias generated by PCR duplicates and sequencing errors. Genotyping errors lead to data that deviate from what is expected from regular meiosis. This, in turn, leads to difficulties in grouping and ordering markers resulting in inflated and incorrect linkage maps. Therefore, genotyping errors can be easily detected by linkage map quality evaluations.
Results We developed and used the `Reads2Map` workflow to build linkage maps with simulated and empirical GBS data of diploid outcrossing populations. The workflows run `GATK`, `Stacks`, `TASSEL`, and `Freebayes` for SNP calling and `updog`, `polyRAD`, and `SuperMASSA` for genotype calling, and `OneMap` and `GUSMap` to build linkage maps. Using simulated data, we observed which genotype call software fails in identifying common errors in GBS sequencing data and proposed specific filters to better handle them. We tested whether it is possible to overcome errors in a linkage map using genotype probabilities from each software or global error rates to estimate genetic distances with an updated version of `OneMap`. We also evaluated the impact of segregation distortion, contaminant samples, and haplotype-based multiallelic markers in the final linkage maps. Through our evaluations, we observed that some of the approaches produce different results depending on the dataset (dataset-dependent) and others produce consistent advantageous results among them (dataset-independent).
Conclusions We set as default in the `Reads2Map` workflows the approaches that showed to be dataset-independent for GBS datasets according to our results. This reduces the number required of tests to identify optimal pipelines and parameters for other empirical datasets. Using `Reads2Map`, users can select the pipeline and parameters that best fit their data context. The `Reads2MapApp` shiny app provides a graphical representation of the results to facilitate their interpretation.

Key words: genotyping error; haplotype; genetic maker; multiallelic

Compiled on: June 27, 2023.
Draft manuscript prepared by the author.

Introduction

Advances in sequencing technologies and the development of different genome-reduced representation library protocols result in millions of genetic markers from hundreds of samples in a single sequencing run [1, 2, 3, 4]. Increasing the number of markers and individuals genotyped can enhance the capacity of linkage maps to locate recombination events that occur, resulting in higher map resolution and better statistical power for the localization of QTL in further analysis. This large amount of data and genotyping errors common with genotyping-by-sequencing approaches [5] increases the need for computational resources and multiple bioinformatic tools.

Genotyping errors are frequent when high-throughput sequencing technology is applied to reduced representation libraries. There are a variety of protocols to create these types of libraries [4], called Restriction-site Associated DNA sequencing (RADseq) or genotyping-by-sequencing (GBS) [6, 7]. Generally, one or more restriction enzymes are used to digest the sample DNA. The resulting DNA fragments are filtered by size, connected to adaptors and barcodes, amplified by PCR, and sequenced. Consequently, most sequences obtained are PCR duplicates of the regions around the enzyme cut site. By relying on duplicates to increase sequencing depth, such methods introduce errors and a sequencing bias towards one of the alleles due to variabilities in the PCR amplification. These errors are hard to detect by bioinformatic tools [8, 9].

To overcome genotyping errors coming from GBS methods, genotype calling software model sequencing error, allelic bias, overdispersion, outlying observations, and the population Mendelian expected segregation [10]. Building a genetic map with genotypes obtained using these methods can be a powerful tool to validate their efficiency. Wrong decisions or inefficient methods in all steps before linkage map building can be identified in the resulting map as errors that dissociate the map properties from biological processes. For example, genotyping errors generate inflated map sizes that show an excessive number of recombination breakpoints during meiosis [11]. The first genetic map studies by Morgan and Sturtevant [12] discovered that crossing-overs are unlikely to happen too close to each other, a phenomenon named interference. Later studies describing the meiotic molecular mechanisms confirmed the low expected number of recombination breaks in a single event [13].

Recently developed approaches to build linkage maps [14, 15, 16] were implemented in `OneMap` [17] 3.0 package. They use quantitative genotype probability measurements rather than the traditional qualitative genotypic information from SNP and genotype calling methods to account for genotyping errors and provide higher-quality genetic maps. These probabilities can be applied in different ways: using the probability of each possible genotype (PL field in VCF format); using an error probability associated with the called genotype (GQ field in VCF format); or using a global error rate that will be applied to all genotypes. Nevertheless, even using these approaches, building a linkage map will succeed only if the upstream software can identify the errors and provide reliable genotypes or their probabilities.

The biallelic codominant nature of SNPs is another characteristic of high-throughput markers that can affect linkage map building of outcrossing species. Although biallelic markers can distinguish only two haplotypes, the mapping population of outcrossing diploid species inherits two haplotypes with combinations of four different parental haplotypes. With biallelic markers, the observed parental genotypes are limited to types $ab \times ab$, $ab \times aa$, and $aa \times ab$. When one of the parents is homozygous ($ab \times aa$ and $aa \times ab$), it is impossible to observe the crossing-over change for this uninformative parent. So this is taken as missing information (non-measurable crossing-overs) for linkage map building if only two-point information is considered. Therefore, building a linkage map with only biallelic markers requires a multi-point approach that uses loci information with both parents heterozygous ($ab \times ab$) to estimate the recombination of loci where one parent is homozygous, and the recombination information is missing for closely linked loci. The multi-point approach applies likelihood computations involving several loci and has been successfully used since the seminal publication of Lander and Green [18]. The approach makes it possible to identify the four different parental haplotypes by phasing the biallelic information so that the SNPs can be used to identify all the allelic diversity.

Other approaches to overcome the low informativeness of biallelic markers involve combining adjacent biallelic markers in the same disequilibrium block (high LD) into a single multiallelic haplotype. These haplotype-based markers showed higher accuracy in association analysis than individual biallelic SNPs [19, 20, 21, 22, 23, 24, 25]. N'Diaye et al. [21] and Jiang et al. [25] pointed out several advantages of haplotype-based markers, including the higher capacity to identify epistatic interactions, the presence of more information to estimate identical-by-descent alleles and the reduction of the number of statistical tests to perform.

Despite the availability of many software for estimating genotype probabilities [26, 2, 27, 26, 28, 29, 10] and haplotype-based multiallelic markers [26, 30], there are no recommendations about which combination and choice of parameters are the best for building linkage maps. Therefore, this work evaluates the consequences of building maps by applying genotype probabilities and haplotype-based markers from different software and parameters. To achieve these, we implemented new features in `OneMap` [17], a widely-used software for building maps. We also developed the `Reads2Map` workflow, a tool to help users to select a bioinformatic pipeline that provides the best quality markers to build a linkage map for their dataset. Here, we performed tests with simulated and empirical data and were able to make recommendations to users to obtain better linkage maps in several situations, such as low and high-depth sequencing, with and without segregation distortion, contaminant samples, and multiallelic markers, and using different software to perform the SNP and genotype calling.

Material and Methods

We developed `Reads2Map` (RRID SCR_023593), a collection of bioinformatics workflows using Workflow Description Language (WDL) [31]. It enables sequence alignment, SNP and genotype calling analysis, and linkage map construction. With `Reads2Map`, researchers have the flexibility to explore various software options and parameter combinations, enhancing the construction of linkage maps. The workflows are available in GitHub (https://github.com/Cristianetaniguti/Reads2Map) and in workflowhub.eu [32, 33].

The `EmpiricalReads2Map` workflow was designed to evaluate empirical (real) datasets; and the `SimulatedReads2Map` workflow, to simulate and evaluate datasets (figure 1). Both are composed of subworkflows that can be run independently, which increases usage flexibility. There are multiple options available for running WDL workflows. Some of them are Terra.bio platform [34] and Cromwell Execution Engine [31].

Each WDL task in `Reads2Map` is related to a Docker [35], or Singularity [36] container. Some of the container's images used in `Reads2Map` are available in open repositories and others were built using Dockerfiles stored in the `Reads2Map` repository and available in DockerHub. Check a list of all software and image versions used in Supplementary Table 1. We ran the analysis testing workflows on two high-performance computers (Texas A&M University HPRC, University of São Paulo Águia Cluster).

For building linkage maps, we implemented updates in `OneMap` package version 3.0 (https://CRAN.R-project.org/package=onemap) and used this version in the workflows. We also developed the `Reads2MapTools` (https://github.com/Cristianetaniguti/Reads2MapTools) R

package for support functions and Reads2MapApp shiny app (https://github.com/Cristianetaniguti/Reads2MapApp), a visualization tool that receives as input the final workflow output and provides summary statistics about the resulting linkage maps, intermediary steps, and workflow performance.

SNP calling

The first step of the workflows is the SNP calling. To start with GATK [27], Stacks [2], and Freebayes [26] approaches, the demultiplexed FASTQ sequences are first aligned to their respective reference genomes using BWA-MEM [37]. The workflow uses samtools [38] to merge the alignment of replicates, keeping the libraries identification on the BAM header and filtering out reads with MAPQ < 10. After the alignment, BAM files for each sample are used as inputs for sub-workflows with GATK, Stacks, and Freebayes tasks. The gatk_genotyping sub-workflow reproduces GATK joint genotyping via HaplotypeCaller, GenomicsDBImport, and GenotypeGVCFs tools and applies the suggested hard-filtering procedures [8]. The freebayes_genotyping sub-workflow runs Freebayes parallelized by reference genome intervals. The stacks_genotyping sub-workflow includes the option to input the population file. If not included, all individuals are considered from the same population. It runs the gstacks and the populations plugins.

The TASSEL [1] SNP caller is implemented in the tassel_genotyping sub-workflow. It first adds fake barcodes to the demultiplexed fastq sequences. After, it runs the plugins GBSSeqToTagDBPlugin and TagExportToFastqPlugin. The generated tags are aligned to the reference genome using BWA-MEM and the alignment files are input for the SAMToGBSdbPlugin plugin which produces a database. The database was processed by the DiscoverySNPCallerPluginV2, SNPQualityProfilerPlugin, and ProductionSNPCallerPluginV2 plugins.

After obtaining the VCF file using one or more of the SNP calling methods, indel marker positions are left-aligned and normalized with BCFtools [39].

Genotype calling

The VCF files with biallelic markers from Freebayes, TASSEL, Stacks, and GATK are the input for the genotype caller software polyRAD [28], SuperMASSA [29], and updog [10]. These three software are implemented in the sub-workflows genotyping_empirical and genotyping_simulated.

To use the polyRAD approach, the VCF files are imported using VCF2RADdata without applying any filters or considering phase information. The polyRAD model is run with PipelineMapping2Parents default arguments which assume an F_1 bi-parental population. The function Export_MAPpoly is used to export the genotype probabilities. The vcfR package [40] and custom R (function polyRAD_genotype_vcf in Reads2MapTools package) code is used to store outputted genotypes and their probabilities in a new VCF file. We also adapted SuperMASSA scripts to output the genotype probabilities information. The modified version is available in Reads2MapTools package. A wrapper function called supermassa_genotype, available in the package, can run the model in parallel and export the results to a new VCF file. The F_1 SuperMASSA model is run with the parameter naive_posterior_reporting_threshold set to zero to not filter any genotype. The updog F_1 model is used in parallel using the function multidog through the Reads2MapTools wrapper function updog_genotype which outputs the results in a new VCF file.

The software GUSMap performs the genotype calling and linkage map building with a single model. We use VCFtoRA function to convert the outputted VCF files from GATK, TASSEL, Stacks, and Freebayes approaches into GUSMap format. A pedigree of the population and a list of filters (MAF = 0.05, MISS=0.25, BIN=0, DETPH=0, and PVALUE=0.05) is provided to the readRA function. The function makeFS is used to create the full-sib population information. Functions infer_OPGP_FS and rf_est_FS are used to estimate the phase and recombination fraction given the genomic order of the markers. In some situations, the function rf_est_FS outputs infinite values of the recombination fraction. In these situations, our pipeline removes the respective marker and runs the function again. This workaround code can increase the time required to run GUSMap.

Updates in OneMap 3.0 for building linkage maps

OneMap is an open-source R package that has been serving the research community since its initial release in 2007. It offers a comprehensive suite of functions designed to facilitate marker filtering, grouping, ordering, and genetic distance estimation in both inbred and outbred populations. The genetic distances estimation is made using a Hidden Markov Model (HMM) multipoint approach. The forward-backward algorithm [41] is implemented to compute the HMM combined with the expectation-maximization algorithm (EM).

The OneMap latest version (3.0) is implemented in Reads2Map workflows. In this new version, we have introduced a new feature to enhance the flexibility of the HMM in scenarios where genotyping errors are expected in the dataset. This update includes the create_probs function and modifications to the HMM algorithm. With this option, users can provide OneMap with prior information regarding the reliability of each input genotype, thereby increasing the HMM's adaptability. The create_probs function allows users to input three types of values: a global error value (global_error); an error probability for each inferred genotype (genotypes_error); or genotype probabilities for each possible genotype in individuals (genotypes_probs). This flexibility empowers users to tailor the analysis to their specific dataset characteristics and improve the accuracy of the results. This update is described in detail in Supplementary File 1.

The OneMap software previous to version 3.0 considered the HMM error probability as a single value of 10^{-5} for every genotype. In version 3.0, this value is kept as default to keep the code reproducible. But it is noteworthy that this probability can be unreliable in several situations when the genotypes are more prone to errors, especially for new genotyping technology (e.g. GBS data).

OneMap 3.0 updates also include the possibility to parallelize the HMM using the approach described by [42]. It parallelizes the procedure into a maximum of four cores. We used this new OneMap feature to estimate the genetic distances. We also implemented new functions for linkage maps quality diagnostics such as interactive plots for recombination fraction matrices, progeny haplotypes representation, and counts of the recombination breakpoints in progeny.

Despite using the parallelized HMM, the genetic distances estimations in OneMap can take time to run with a high number of markers, chromosomes, and tested combinations of software. Therefore, the EmpiricalReads2Map workflow runs the HMM in just a subset of markers which can be a single chromosome or a fragment of a chromosome. The alignment, the SNP, and genotype calling steps are performed with the entire dataset. After running the workflow and deciding the pipeline that provided the best results, the respective VCF output can be used to build the linkage map for all chromosomes in the R environment with OneMap functions.

The OneMap function onemap_read_vcfR is used to convert the VCFs to the OneMap R object format. The markers are filtered again by a maximum number of missing data of 25% because the VCF files include unexpected genotypes according to the segregation of a given locus (e.g. in a cross "AA x AB", genotype "BB" cannot exist). OneMap makes this genotype calls missing. Markers are also filtered if the segregation distortion is under a global significance level of 0.05 with Bonferroni correction and if they are redundant.

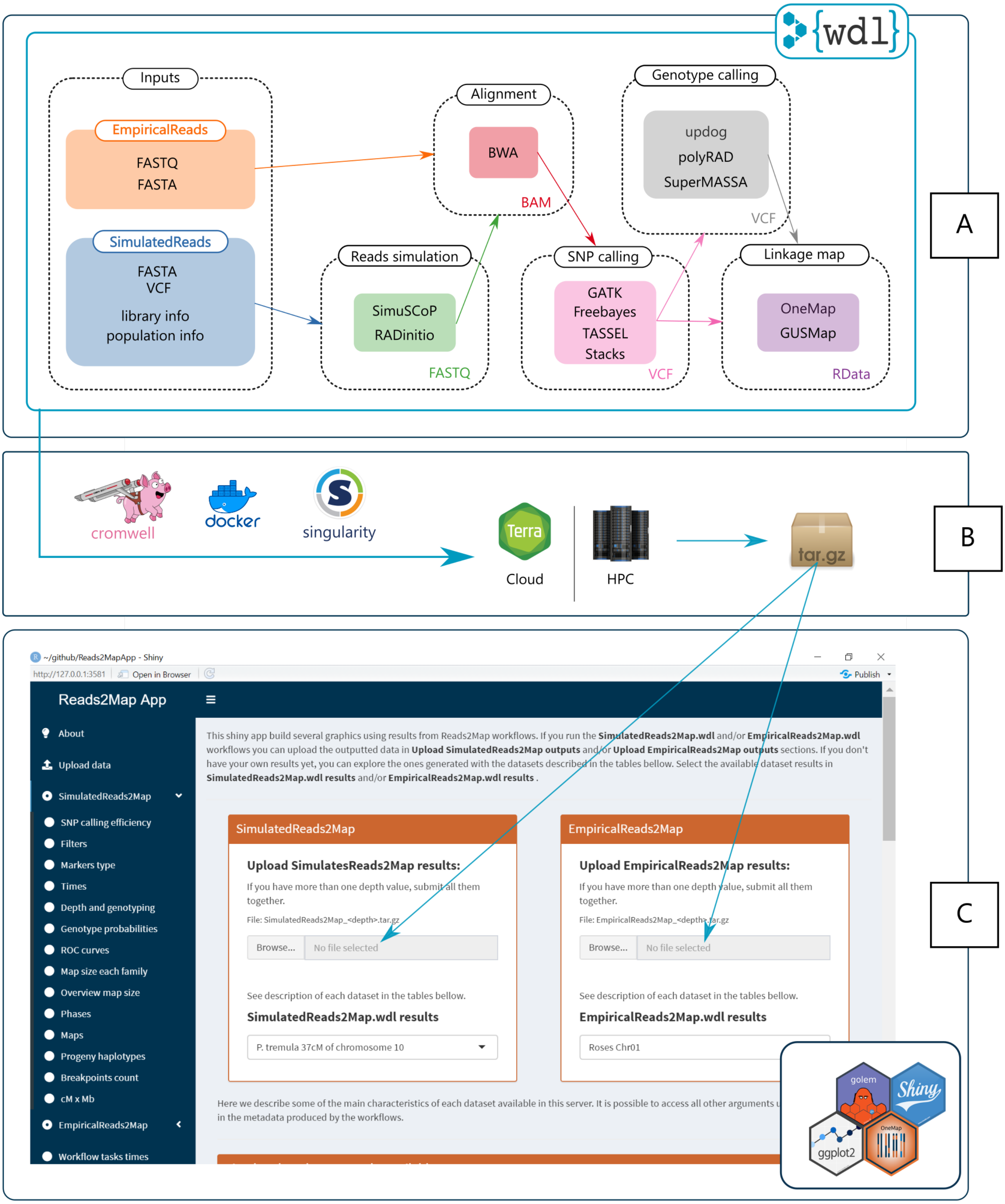

Figure 1. A: Tasks of the two main `Reads2Map` workflows: `EmpiricalReads2Map` and `SimulatedReads2Map`. B: Tools to run the workflows on the Cloud (`https://app.terra.bio/` platform) or in High-Performance Computing (HPC) environments. C: The `Reads2Map` shiny app has as input the outputs of the workflows. It builds several descriptive graphics to evaluate the best upstream software combination for linkage map construction.

Markers are ordered according to the reference genome position.

The `Reads2Map` workflows give flexibility to the user to define the probabilities to be used in the `OneMap` HMM for the estimation of the genetic distances. Users can provide more than one value to be tested as global errors (global_error input); can choose to use the upstream genotype caller error probability (genoprob_error input); and can provide global error values to be considered together with the software probabilities (genoprob_global_error input) according to the following: $1 - (1 - \textit{global error})x(1 - \textit{software error probability})$.

For `GATK`, `TASSEL`, `Stacks`, and `Freebayes` callers, the workflow uses in the HMM the Phred score genotype error (GQ FORMAT value) converted to probabilities. For the software `polyRAD`, `SuperMASSA`, and `updog` it uses $1 - \textit{output genotype probability}$ as a genotype error. For these last, the population's structure (F_1) is used as *a priori* information to increase the accuracy of the estimated genotypes.

The simulations do not consider interference in the recombination events. Therefore the Haldane map function was used to estimate the genetic distances in `SimulatedReads2Map`. Kosambi's map function was applied to estimate the genetic distances in the `EmpiricalReads2Map`.

Read2Map Workflows App

The shiny app `Reads2MapApp` was built to display results from the workflow analysis. It includes graphics and statistics about SNP calling efficiency, the number of markers discarded by filtering steps, marker types, computer resources and time spent by each step of the workflow, allele depth by genotype, genotype probabilities, map size, map phases, recombination fraction matrix, progeny haplotypes, breakpoints count, and the correlation between linkage map and reference genome markers positions. `Reads2MapApp` is a modular R package using the golem framework [43] that can be rendered and displayed locally or on a server. It can be installed from its GitHub repository and run with a single command (run_app). Once the `Reads2Map` output file is uploaded into the app, all graphics will be automatically generated.

Empirical datasets

We used the structure of `Reads2Map` to test the effects in the linkage map built using different combinations of software, and parameters in datasets with different characteristics. For our tests with empirical data, we used two datasets from previous works. They are GBS datasets from a bi-parental diploid F_1 full-sib mapping populations of aspen (*Populus tremula L.*) [44] (BioProject PRJNA395596), and rose (*Rosa spp.*) [45]. The aspen dataset comes from an intraspecific cross of two *Populus tremula* genotypes. The GBS libraries were built using *HindIII* and *NalIII* enzymes and sequenced as 150 base pair single-end reads on an Illumina HiSeq2500. Eight library replicates were built and sequenced for the parents and only one for each of the 116 F1 offspring. The dataset includes six samples erroneously sequenced as part of the progeny and later identified as contaminants. An average read depth of approximately 6x for progeny and 58x for parental samples were observed from the sequencing process. The *Populus trichocarpa* genome version 3.0 [46] was used as a reference for the sequence's alignment. It has about 397 Mb in size.

The diploid roses dataset comprises 138 individuals from the cross between a Texas A&M breeding line J06-20-14-3 (J14-3) and cultivar Papa Hemeray (PH). GBS libraries were built with *NgoMIV* enzyme and sequenced as a 113 base pair single-end read on a HiSeq2500. The parent J14-3 was repeated twice, and the PH sample three times. An average read depth of approximately 94x for progeny and 528x for parental samples were observed from the sequencing process. The *Rosa chinensis* v1.0 genome assembly [47] was used as a reference genome to align the sequences. It has about 527 Mb in size.

Table 1. Marker types according to parental genotype combinations and progeny segregation. The letters "a", "b", "c" and "d" represent different alleles and the letter "o" represents null alleles. Adapted from [50].

Marker type			Parents: Cross	Progeny: Observed genotypes	Progeny: Expected segregation
A		1	ab x cd	ac,ad,bc,bd	1:1:1:1
		2	ab x ac	a,ac,ba,bc	1:1:1:1
		3	ab x co	ac,a,bc,b	1:1:1:1
		4	ao x bo	ab,a,b,o	1:1:1:1
B	B_1	5	ab x ao	ab,2a,b	1:2:1
	B_2	6	ao x ab	ab,2a,b	1:2:1
	B_3	7	ab x ab	a,2ab,b	1:2:1
C		8	ao x ao	3a,o	3:1
D	D_1	9	ab x cc	ac,bc	1:1
		10	ab x aa	a,ab	1:1
		11	ab x oo	a,b	1:1
		12	bo x aa	ab,a	1:1
		13	ao x oo	a,o	1:1
	D_2	14	cc x ab	ac,bc	1:1
		15	aa x ab	a,ab	1:1
		16	oo x ab	a,b	1:1
		17	aa x bo	ab,a	1:1
		18	oo x ao	a,o	1:1

The sequencing reads of the two empirical datasets were filtered using the `Stacks` plugin process_radtags [2] to filter sequences by the presence of the restriction site and sequencing quality. The reads were discarded if the average quality score of 50% of its length was below the Phred score of 10 (or 90% probability of being correct). The software `cutadapt` [48] was used to remove adapters and filter by a minimum read length of 64 bp. The sequences were then evaluated in our `EmpiricalReads2Map` workflow.

Simulated GBS data

The first step of the `SimulatedReads2Map` workflow is to perform simulations of a mapping population, GBS libraries, and sequences. The simulation is based on a given reference genome chromosome sequence. If a reference linkage map and a VCF file are provided, the workflow simulates the marker genetic distances and parental genotype frequencies based on them. A cubic spline interpolation with the Hyman method [49] is applied to simulate the centimorgan position for each marker's physical position based on this same relation on the reference linkage map provided.

We based our simulation analysis on the first 37% of the chromosome 10 sequence of *Populus trichocarpa* version 3.0, which includes a sequence with 8.426 Mb from a total chromosome size of about 23 Mb. This sequence comprises 38 cM (21%) of the linkage group 10 built using the aspen empirical data [44]. Due to the computational resources needed to build such a high number of maps, we used only a subset of the data to finish the analysis in a reasonable time. Chromosome 10 was randomly chosen.

We simulated markers with different expected segregation patterns according to parental genotypes in each locus. Table 2 shows the notation for each possible marker type in an outcrossing diploid population. The `SimulatedReads2Map` workflow simulates parental haplotypes using the same proportion of marker types identified in the empirical VCF file. This approach overcomes the missing data present in the empirical dataset. The final VCF file used as a reference to the simulations contains 810 markers (126 B3.7, 263 D1.10, 278 D2.15, and 143 non-informative markers with both parents homozygous), which results from the aspen empirical data `GATK` SNP calling, filtered by a maximum of 25% of missing data and MAF of 5%.

`PedigreeSim` v2.1 software [51] is implemented in the workflow

to simulate the meiosis events and generate an F_1 progeny based on the provided genetic map and simulated parental haplotypes. We did not consider interference in meiotic events (Haldane [52] mapping function). PedigreeSim output files were converted to VCF files using Reads2MapTools R package function pedsim2vcf.

While converting the files, the pedsim2vcf function can also simulate segregation distortion by applying a selection strength. For that, a high number of individuals in the progeny have to be simulated with the PedigreeSim software and one or more loci to be under a given selection intensity. In our study, we targeted a final population size of 200 individuals. For that, we simulated 50 $\times$ 200 individuals and applied a selection intensity of 50% in the 30th marker, eliminating 50% of the genotypes containing one of the alleles. Then, 200 individuals of the resulting population are randomly selected to compose the mapping population. We used this feature to compare software performance in segregation distortion.

The VCF file output by pedsim2vcf and the reference genome file are inputs for the RADinitio [9] software. RADinitio adds the VCF polymorphisms in the reference genome sequence and simulates the GBS sequences. It uses the inherited efficiency model [53] to simulate a PCR-amplified pool of molecules. The model includes the heterogeneity of the PCR amplification and the polymerase substitution errors. Next, RADinitio applies the user-defined ratio between DNA original molecules to be sequenced and PCR duplicates to create a distribution that will define the number of times the pool of loci is sampled, the number of duplicate molecules that are generated from a RAD locus template, and the distribution of PCR errors in the resulting reads. We defined the default parameter with a proportion of 4:1. Besides the PCR errors inserted during the pool sampling, the software also includes a commonly observed error pattern, where the 3' end of the read accumulates more errors than the 5' [54]. We tested different values of PCR cycles (5, 9, and 14) and mean depth (5, 10, and 20) to simulate the FASTA files. We set the other RADinitio simulation parameters to obtain 150 bases of read length, sequence size of 350 (parameter "–insert-mean"), and restriction enzymes *HindIII* and *NalIII*. The mean read depth parameter for the parental samples was eight times higher than the progeny. The combination of RADinitio parameters that produced results closer to those observed in empirical data was selected to perform simulations with and without segregation distortion, five repetitions (five families), and two average sequencing depths (10 and 20) and 5 PCR cycles.

RADinitio does not output the sequence quality scores, so we converted the FASTA file format to FASTQ format, including a Phred score of 40 for every base simulated using seqtk [55] software. After obtaining the FASTQ files, the SimulatedReads2Map workflow followed the same tasks as the EmpiricalReads2Map, with alignment, SNP and genotype calling, and linkage map build. The SimulatedReads2Map workflow makes comparisons between real and estimated results within each step. The comparisons made during the workflow can be visualized in the shiny app Reads2MapApp.

Tested scenarios

We ran all implemented software for SNP calling and genotype calling (GATK, Freebayes, TASSEL, Stacks, updog, SuperMASSA, and polyRAD) on the empirical and simulated datasets. In addition, we explored the substitution of VCF allele counts with counts from the alignment (BAM) files to mitigate potential biases introduced by SNP caller software when analyzing low-coverage sequence data. GATK inserts the bias when reads are filtered in the local re-assembly step to avoid sequencing errors [56]. BCFtools is used to find the read depths information for each allele in BAM files and update the allele depths information in the AD (allele depth) field of the VCF file. For the Aspen dataset, we also executed the workflows for every scenario in the presence of the contaminant samples.

The markers identified by the SNP callers (GATK, TASSEL, Stacks, Freebayes) were filtered by minor allele frequency (MAF) of 5% and maximum missing data allowed of 25% before proceeding to the genotype callers (updog, polyRAD, and SuperMASSA). At this step, we also tested two other filters. One of them was removing non-informative markers from the VCF file. We considered non-informative markers homozygous in both parents or if at least one of the parental genotypes was missing. The second filter was to replace the allele depth (AD) field in the VCF file format by missing data when the genotype is missing. This avoids that updog, polyRAD, and SuperMASSA use the allele depth when GATK filtered out the genotype due to bad quality.

After the genotype call, we reduce the analysis to a subset of markers (the first 8.426 Mb or 37%) of *Populus trichocarpa* chromosome 10 and the first 25 Mb (37%) of *Rosa chinensis* chromosome 1 reference genomes. This made it possible to build maps for all tests in a feasible time. The markers were filtered by the maximum missing data allowed of 25%, redundancy, and segregation distortion. In addition, we tested filtering the genotypes by a minimum genotype probability of 0.8.

We tested the consequences of building maps applying different genotype probabilities in the OneMap 3.0 HMM coming from seven different genotype caller software: GATK, Freebayes, TASSEL, Stacks, polyRAD [28], SuperMASSA [29] and updog [10]; a global error rate of 0.01, 0.05, 0.1, and the OneMap 2.0 default value of 10^{-5}. We also tested the combination of the two distributions. We compared OneMap 3.0 capacity of estimating accurate genetic distances with the GUSMap package [14] estimations since it also uses an HMM to account for errors present in sequencing data.

We also tested the consequences of the presence and absence of the and Stacks haplotype-based multiallelic markers in the linkage map. To test the influence of the presence of the multiallelic markers in the ordering procedure, we built a map for the entire chromosome 1 and 10 from the roses and aspen datasets, respectively, using the selected pipeline. We ordered the markers using MDSMap [57] (wrapper function implemented in OneMap 3.0) ordering algorithm with and without multiallelic markers.

In the testing of scenarios in which we considered multiallelic markers, the VCFs containing them are merged into the VCF files from polyRAD, SuperMASSA, and updog. The merged VCF is the input for linkage map building in OneMap version 3.0.

Table 2 shows an overview of the notations used to refer to each evaluated scenario.

Performance comparison

We conducted performance comparisons of each tested dataset and scenario based on the built linkage map quality. To consider good quality we evaluate the following linkage map characteristics:

- Marker type:
  In outcrossing populations, it is important to have markers that have recombination information for both parents. We avoided approaches that provide only ab x aa (D1.10) or aa x ab (D2.15) in a single chromosome. The Reads2MapApp "Marker type" section describes the amount of each marker type in the linkage maps built by Reads2Map workflows.
- Marker coverage:
  It refers to how equally distributed markers are in the genome. We avoided approaches that do not detect markers in a large portion of the genomic selected area. The graphics in Reads2MapApp section "cMxMb" section correlate the linkage map position with the genomic positions. This is an excellent tool to evaluate marker coverage.
- Marker density:
  It refers to how equally distributed markers are on the linkage map. We avoided big gaps (higher than about 10 cM) in

Table 2. Notation used to refer to each evaluation scenario in empirical and simulated datasets.

Workflow step	Notation	Description
Reads simulations	Depth 10<br>Depth 20	Mean read depth used<br>to simulate the dataset
	segregation distortion	Dataset simulated with<br>segregation distortion
SNP calling	`Freebayes`<br>`GATK`<br>`TASSEL`<br>`Stacks`	Software used to<br>identify the variants
Counts source	BAM<br>VCF	Source files of allele depth information
Filters	only informative markers	Filter non-informative markers<br>(both parents homozygous<br>or at least one missing)
	missing replaced	Replace AD field for missing<br>data when GT is missing
Genotype calling	`polyRAD`<br>`SuperMASSA`<br>`updog`	Software used to perform the<br>estimation of genotype for a<br>given allele depth information
	SNPCaller	Software used to genotype calling is<br>the same that performed the SNP calling
Filters	genotype prob >0.8	Filter by minimum genotype<br>probabilities of 0.8
Marker type	biallelics	Keep only biallelic markers
	biallelics + multiallelics	Keep biallelic and multiallelic markers
Map building	<Genotype caller name>	Maps built with genotype<br>probabilities from<br><Genotype caller name>
	<Genotype caller name><br>(<global error rate>%)	Map built with genotypes from<br><Genotype caller name>and<br>global error of <global error probability>
	<Genotype caller name>x<br>(<global error rate>%)	Map build with genotypes probabilities<br>from <Genotype caller name>and global<br>error of <global error probability>

the linkage maps. Some of the gaps observed in the maps are due to outlier markers (a single marker with gaps in both edges). Outlier markers can be removed manually in further steps. We search for approaches that provided fewer outlier markers, which would require less manipulation later. The linkage map draw and graphics about the genetic distances among markers present in the section "Map size" of `Reads2MapApp` are good tools to evaluate marker density.

- Marker order:
  The efficiency of ordering algorithms can be significantly influenced by the presence of marker types that provide recombination information for both parents. In the `Reads2Map` workflows, to ensure accurate comparisons and to be possible to distinguish if linkage map inflation is due to different orders or genotyping errors, we have standardized the marker order across the workflow comparisons. Therefore, the order of the markers is always based on the reference genome. This means that it is crucial to carefully select, for the workflows, tests chromosome regions in the datasets that do not exhibit inversions or translocations when compared to the reference genome.
  However, in order to assess the impact of highly informative haplotype-based multiallelic markers, we conduct separate experiments outside of the workflows. In these experiments, we exclude outlier markers and evaluate the efficiency of the MDS ordering algorithms with and without the inclusion of multiallelic markers. This allows us to investigate these markers' influence on the algorithm's performance. We evaluated the orders provided by the different ordering algorithms by computing the absolute value of Spearman's rank correlation between orders.
- Marker quality:
  In cases where all markers are correctly ordered (following the standardization in `Reads2Map` comparisons), and there is sufficient coverage and density, an inflated size of the linkage map can be attributed to a high error rate in the genotypes. Our objective is to find an approach that minimizes this inflation and brings the linkage map size closer to the expected value (e.g., 38 cM in our tested subsets).
  To identify the causes of inflated maps, the linkage map draw and recombination fraction matrix heatmap generated by `Reads2MapApp` prove valuable. It enables us to distinguish whether the inflation is a result of outlier markers creating gaps or due to genotyping errors.
- Estimated haplotypes:
  Together with the linkage map, the `OneMap` HMM multipoint approach also estimates the parents and progeny haplotypes. In a scenario without contaminant samples, we expect a low (around 1 or 2) and equally distributed number of recombination breaks across all samples. In scenarios where there are contaminant samples, we expect that their haplotypes contain a high number of estimated breaks because wrong assumptions were made leading to the wrong estimated number for these samples. `Reads2MapApp` contains a section for visualizing the progeny haplotypes and also for counting the estimated number of recombination breaks.

Results and Discussion

We use the structure of the `Reads2Map` workflows, the simulated, and the empirical datasets to test each software and some different parameters and markers filters. Our goal was to identify the approach that provides the best quality linkage map.

We have categorized the approaches used in our analysis into two groups: dataset-independent and dataset-dependent. The

Table 3. Reads2Map workflows default option set based on tests with empirical em simulated data.

Process	Workflow options	Default
SNP calling	run GATK	TRUE
	run Freebayes	FALSE
	run Stacks	TRUE
	run TASSEL	FALSE
	remove duplicates	FALSE
	replace AD by BAM counts	FALSE
	GATK hard filters	TRUE
genotype calling	replace AD by missing when GT is missing	TRUE
	probability threshold	0.8
	run updog	TRUE
	run polyRAD	TRUE
	run SuperMASSA	FALSE
	run GUSMap	FALSE
linkage map	filter non-informative	TRUE
	add multiallelics	TRUE (if available)
	global errors	0.05
	genotype caller probabilities	FALSE
	genotype caller probabilities + global errors	0.05

dataset-independent approaches consistently produce reliable results across all datasets, while the dataset-dependent approaches exhibit varying efficiency depending on the dataset characteristics. To streamline the user experience, we have selected the dataset-independent approaches that improve linkage map quality as the default options in the Reads2Map workflows (table 3). This simplifies the process for users by reducing the number of tests required, as these default approaches consistently yield favorable results across different datasets.

We focused our tests and set the default options based on F_1 diploid populations and GBS markers. However, because the Reads2Map workflow is modularized, the EmpiricalSNPCalling sub-workflow can be used separately and applied to other population structures, ploidy, and sequencing libraries. In the case of working with sequencing libraries other than RADseq, such as Whole-Genome-Sequencing (WGS) or Exome sequencing, it is important to set the option "remove duplicates" to TRUE. The PCR duplicates in RADseq data constitute the majority of the data and they are included in the allele count while calling the genotypes, but in other types of libraries, they are considered artifacts and are removed to avoid errors [58].

The genotype call and linkage map building in the EmpiricalMap sub-workflow have the F_1 population structure as an assumption. In this current version, they can be applied to another type of sequencing library but not to another type of population structure. For these steps, it is just important that the VCF file format is standardized and can be processed by BCFtools. They do not need to be necessarily from the SNP call software implemented. They can be also a combination of VCFs from different software such as the common markers between the implemented SNP call software results ("intersect" in Figure 2).

We had to perform extra manipulations in TASSEL VCF output to be able to run the downstream analysis because they presented missing header information. Also, processing Freebayes showed to consume an unexpectedly high amount of RAM memory in some situations, which made it impossible to automatize the amount of memory required from the HPC and Cloud by the workflow task.

The number of markers identified by each software is related to the species, library preparation, and sequencing aspects such as genome size, restriction enzyme used, and sequencing depth. In figure 2, we can observe that more markers were identified in Aspen dataset compared to the Roses due to the higher frequency of enzymes cut sites. There is no consistency between the two datasets about which of the software identifies the higher number of markers.

After all the filtering steps and linkage map building, it is consistent that Freebayes keeps more markers. However, the resulting maps built with Freebayes markers, genotypes, and genotypes probabilities presented higher genetic distances inflation compared to the other approaches. Using TASSEL software markers also resulted in higher inflation in Aspen dataset maps which have lower sequencing depth ($\sim$ 6x) compared to the Roses ($\sim$ 94). The other approaches also presented outlier markers that inflate the total map size, but, because they are individual markers, they can be easily removed in further steps. The maps built with only common markers among all four software (intersection in figure 2) contained fewer markers and have markers distances similar to GATK and Stacks results.

Evaluating the results of our simulations for GATK, we identified a format characteristic of VCFs from this software that leads to genotyping errors in estimations by updog, polyRAD, and SuperMASSA. In such cases, the genotype is considered missing in the GATK output VCF GT format field, while the total read depth is always reported in the reference allele field of the AD format field (e.g., Estimated = GT:AD ./.;22,0 | True = GT:AD 1/1;0,22).

We present examples of the consequences of this format in genotypes called by updog, polyRAD, and SuperMASSA in figures 3 and 4. In figure 3 A, allele dropouts are observed in the genotype of parent P2 and some of the progeny individuals. In empirical data, allele dropout can occur due to various reasons, such as polymorphisms in the cut site or the non-amplification of one allele during the PCR step [9]. Our simulations also consider allele dropout, but in the observed scenario, the source of allele dropout is due to the format characteristic of the GATK VCF file.

The occurrence of genotyping errors while using GATK VCF allele counts was previously observed by [56], who suggested using counts from BAM alignment files to address the issue (Figure 3 B). However, when testing the usage of BAM allele counts, we lose the advantage of the robust filtering applied by the GATK pipeline to retain only high-quality read counts in its VCF allele depth field. To maintain the accuracy of the GATK allele depth while overcoming the common error observed when the genotype is missing, we replaced the VCF allele count (AD and DP fields) with zero when the genotype information is missing before utilizing it for genotyping with polyRAD, SuperMASSA, and updog. This more precise way of solving the issue was only possible due to our simulations studies once they provide a clear comparison between simulated (true) and estimated data which highlighted the sources of the genotyping errors.

We also observed situations in updog, polyRAD and SuperMASSA results where the parental genotypes are wrongly estimated because of the low quality of the progeny genotypes that distort the expected segregation. These genotype call software consider the ex-

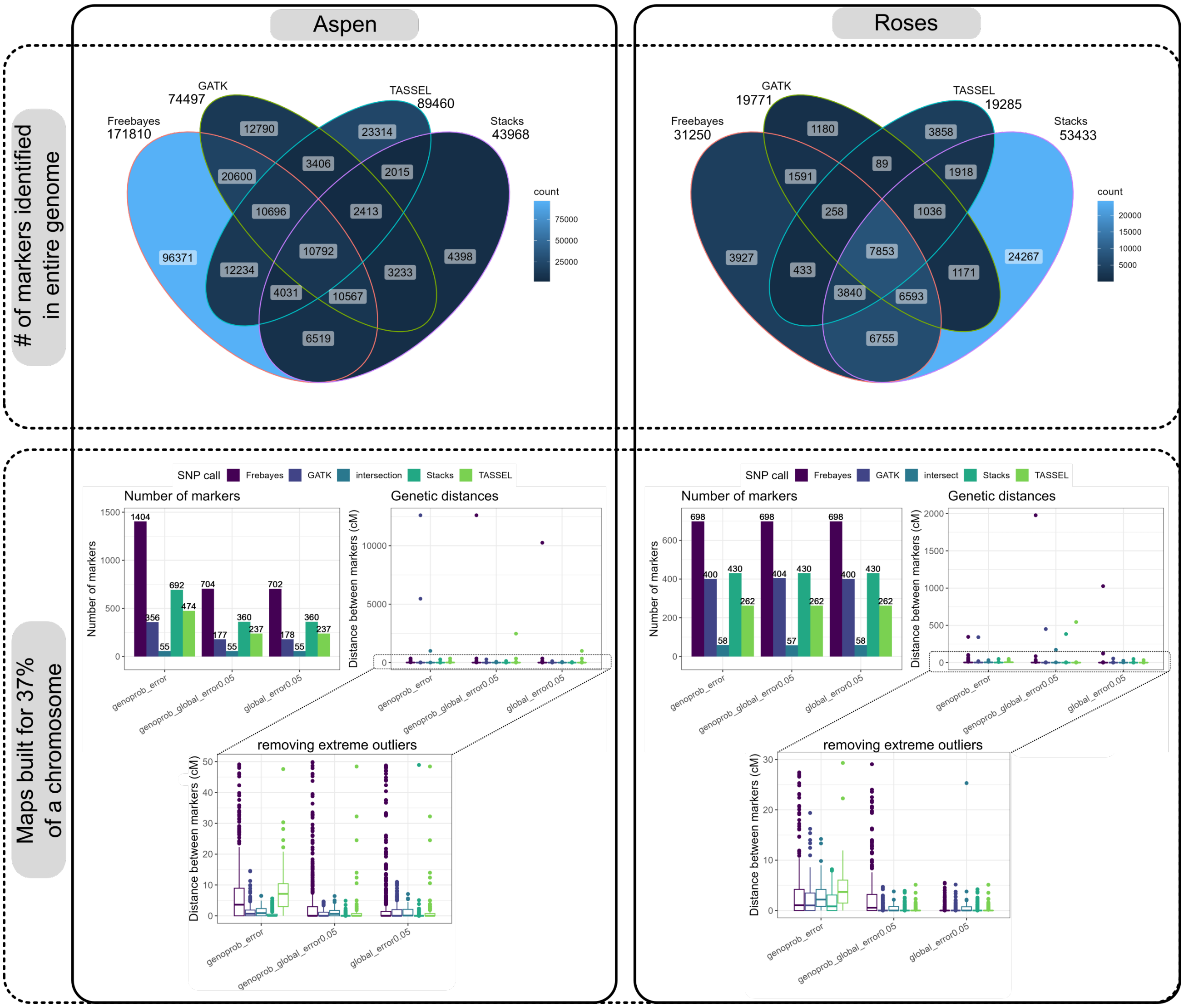

Figure 2. The top two figures show the number of markers identified by each SNP call software (number above each software name) and Venn diagrams showing the number of markers with common positions among all software results for the Aspen and Roses complete datasets. The markers were previously filtered by maximum missing data of 25% and MAF of 5%/. The compatibility of positions among markers from different software was only possible after using "BCFtools norm" to left-align the indels positions. The bottom two figures show the number of markers (bar plot) and distances between markers (boxplot) after building the linkage maps for a subset of 37% of chromosome 10 in the Aspen dataset and 1 in the Roses dataset with the markers from `Freebayes`, `GATK`, `TASSEL`, and `Stacks`. It was considered in the `OneMap` HMM the genotypes and a global error of 5% (global_error0.05); genotypes probabilities (genoprob_error); and the combination of genotype probabilities and a global error of 5% (genoprob_global_error0.5). These figures can be generated for user-defined empirical datasets in the `Reads2MapApp` sections "SNP calling efficiency" and "Map size" after running the EmpiricalMaps workflow.

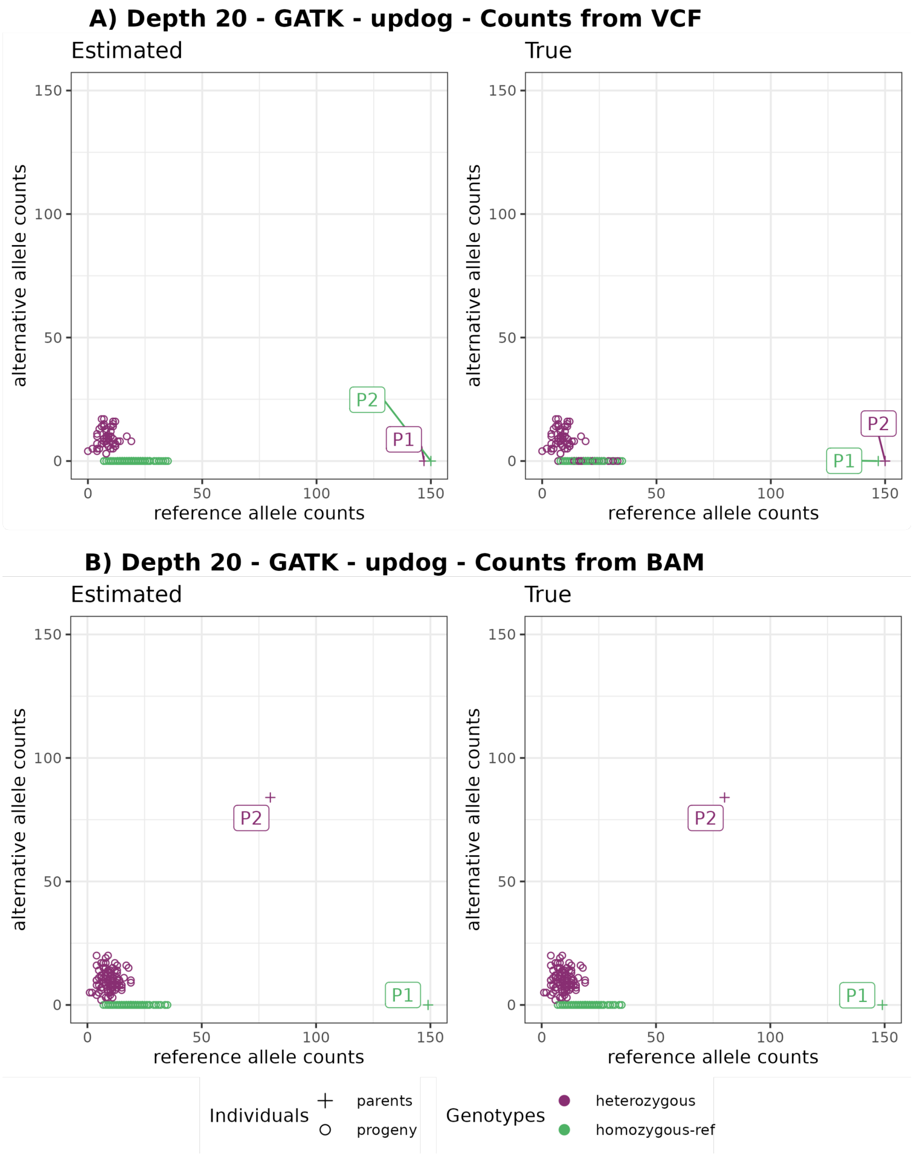

Figure 3. Example of error (Est: homozygous | True: heterozygous and Est: heterozygous | True: homozygous) in parental genotypes leading to a wrong marker type (Est: D1.10 | True: D2.15). Estimated reference (x-axis) and alternative (y-axis) allele count. Graphics on the left have colors according to estimated genotypes, and on the right to the true genotypes. A) show counts from GATK VCF file and B) from BAM file. In the VCF file outputted by GATK the P1 genotype is missing (GT ./.) because the reads did not pass the quality filters, but it reports the counts in the reference AD field (149,0). The `updog` software use progeny segregation (1:1) to estimate the parents, but it makes a mistake identifying which one is heterozygous. Using counts from BAM file (B) fix this issue despite losing the GATK quality filters that can be important in other situations.

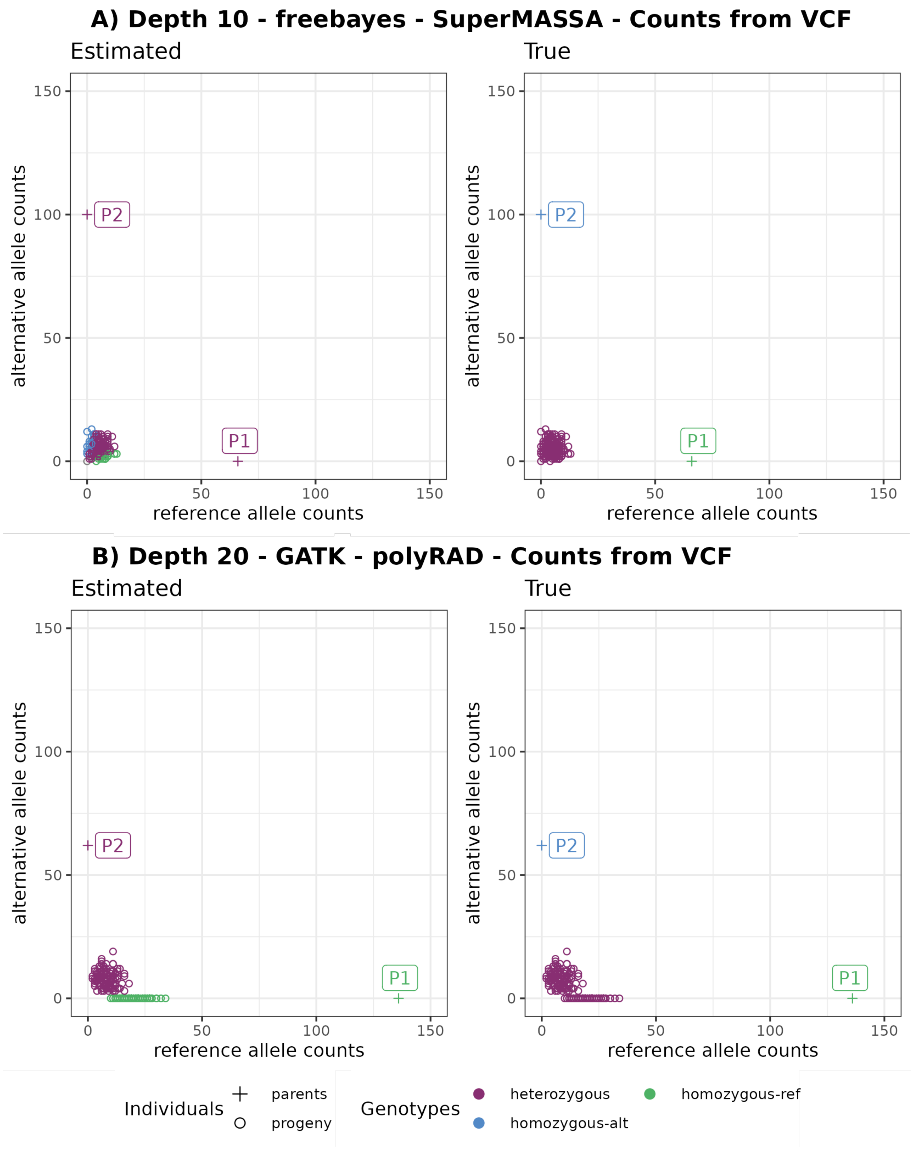

Figure 4. Example of error (Est: homozygous | True: heterozygous) in progeny genotypes leading to wrong marker types in A) Est: B3.7 | True: non-informative and in B) Est: D1.10 | True: non-informative. Graphics on the left have colors according to estimated genotypes, and on the right to the true genotypes.

pected segregation in their models therefore errors in the progeny leads to errors in the parents. Figure 4 shows examples where the marker would be considered non-informative for an outcrossing population, as both parents are homozygous. However, due to genotyping errors in the population, SuperMASSA and polyRAD incorrectly estimate the parents as heterozygous. To tackle this problem, we implement a filtering step to exclude non-informative markers before applying the genotype callers.

Solving these issues was particularly important because erroneous parent genotypes have a higher impact on linkage map quality than progeny genotype errors. OneMap 3.0 does not consider the parental genotype probabilities in its HMM multi-point approach. Thus, it is important to plan the sequencing experiment with high-quality parental genotypes because, if there are errors, they will not be corrected in downstream processing, and it will cause distortions in the resulting distances and haplotypes. To avoid map size inflation, erroneous parental genotypes must be removed before the linkage map analysis.

In general, the evaluations of RADinitio simulations profile shows that we can expect fewer markers and genotyping errors in the simulated compared to the empirical data (Supplementary Figure 7). A smaller number of markers should not reduce the built linkage map quality because the analysis was made in F_1 populations, which have large disequilibrium blocks. However, the smaller number of genotyping errors overestimates the SNP and genotype calling software efficiency. This overestimation is commonly observed in simulation results once the data cannot capture all biases and errors in the empirical data. Thus, we used the simulations to understand specific software limitations and errors source but not ultimately define the best performance [59].

We observed the same or improved quality of linkage maps in the empirical datasets evaluations (Supplementary Figure 8) when we applied these two described filtering steps: removing non-informative data before genotype calling, and replacing allele counts with missing data when the genotype is missing in the GATK calls. After the genotype calling, we applied a threshold of 0.8 to filter low-quality genotypes, which also was beneficial in all scenarios. It is important to notice that these filters are applied before the segregation test filter, which reduces the number of tests and increases the permissibility of the threshold corrected by multiple tests (Bonferroni correction). Thus, the built map can have more markers in some scenarios even if more filters are applied.

The simulations were also useful to validate all code developed for the analysis and to measure the effects of segregation distortion. The results showed that the segregation distortion does not affect the frequency of correct estimated genotypes in most scenarios, despite affecting the reliability of the genotype probabilities provided by updog, SuperMASSA, and polyRAD (Supplementary Figures 9 and 10). This can be one of the reasons why using genotype probabilities in the HMM did not present consistent results across tested datasets.

Despite we considered the HMM error rate dataset-dependent values, we identified that some of the possible values can be discarded. Using the OneMap default value of 10^{-5} global error rate produced bad-quality maps in all situations. The same happened while using all the genotype call software relative error. Using higher values of global error rate and genotypes from GATK, Freebayes, TASSEL, Stacks, updog, and polyRAD, or the combination of the genotype probability and a global error rate from software GATK, updog, Stacks, and polyRAD produced the most reliable linkage maps, with linkage map sizes closer to the expected.

As observed in figure 5, many of the approaches produced linkage maps with distances between all adjacent markers smaller than 10 cM. We chose the method that results in less inflated linkage maps and outlier markers even when applying the small values of the global error rate (0.01). Once the method was selected, we tried an intermediary global error rate (0.075) for the roses dataset values to adjust to the expected total size. We also checked the recombination fraction heatmap, the markers coverage, density, and the number of estimated recombination breakpoints in progeny through Reads2MapApp figures (see the app interface demonstration in Supplementary File 2).

Before using the map size as a metric for map quality, we checked if a map with the expected size always means good quality. A map can have the expected size but a poor quality if the number of overestimated and underestimated recombination breakpoints in the progeny haplotypes is the same; in other words, if they cancel out. To test if this happens in our simulated dataset, we compared the Euclidean relation of estimated and true genetic distances with the total number of wrong (overestimated + underestimated) recombination breakpoints in the progeny haplotypes (Figure 6). For identifying a break as overestimated or underestimated, we do not consider the expected break position but the total breaks expected for the evaluated haplotype. For example, if one haplotype for a specific progeny was simulated with one break and estimated with zero, then we count it as one underestimated break.

The comparison shows that overestimated breakpoints are generally more frequent than underestimated ones. We observe that when a map is inflated, it also has many wrong recombination breakpoints. However, in some cases, the map has the expected map size, but a high number of wrong haplotypes due to both overestimated and underestimated breaks. A high number of underestimated breaks can be observed in situations where the Euclidean distance is close to, or less than 1 ($log_{10}0$) and the number of wrong recombination events is between 10 and 100 ($log_{10}1$ and $log_{10}2$). These situations are more frequent when a global error rate of 5% is used.

In the empirical data results, we observed maps with expected size and excess recombination breakpoints in just a few individuals in the progeny. This variation can be related to contaminant samples. The study of Zhigunov et al. [44] identified six contaminants in the Aspen dataset. When we ran the workflows, including the contaminant samples, the maps built with Freebayes markers and updog, SuperMASSA, and polyRAD were smaller in size than without the contaminant (Supplementary Figure 11). This would (wrongly) suggest better quality if map size is the only metric used. Nevertheless, the maps presented higher differences in the number of recombination breakpoints among individuals when using the genotype probabilities relative to each genotype call software. Some contaminant samples presented more estimated recombination events than the rest of the progeny. Using higher values of global error reduces this difference and can mask the presence of contamination.

These results show that it is important to exclude contaminant samples before the linkage map building once the multi-point HMM approach tends to fix the genotypes according to the biological assumption that they are all F_1 individuals. There are several methods available for identifying contaminant samples in previous steps. The ADMIXTURE [60] software analysis as made by Zhigunov et al. [44] is one possibility. Another is to calculate a marker-based relationship matrix using the R package AGHmatrix [61].

So far, all the evaluations we have discussed have focused exclusively on biallelic markers. We also evaluate the impact on the genetic distances when haplotype-based multiallelic markers are included. In most of the tested scenarios, incorporating these markers leads to map inflation. This is primarily due to the fact that inaccurately estimated multiallelic markers or genotyping errors associated with them can significantly affect the quality of the linkage map. The impact is particularly pronounced because multiallelic markers provide richer information, including recombination and phase information for both parents, compared to biallelic markers. However, the advantages of including the multiallelic markers appear in the marker ordering step.

Algorithms that use two-point recombination fractions estimations have issues ordering only biallelic markers because of the

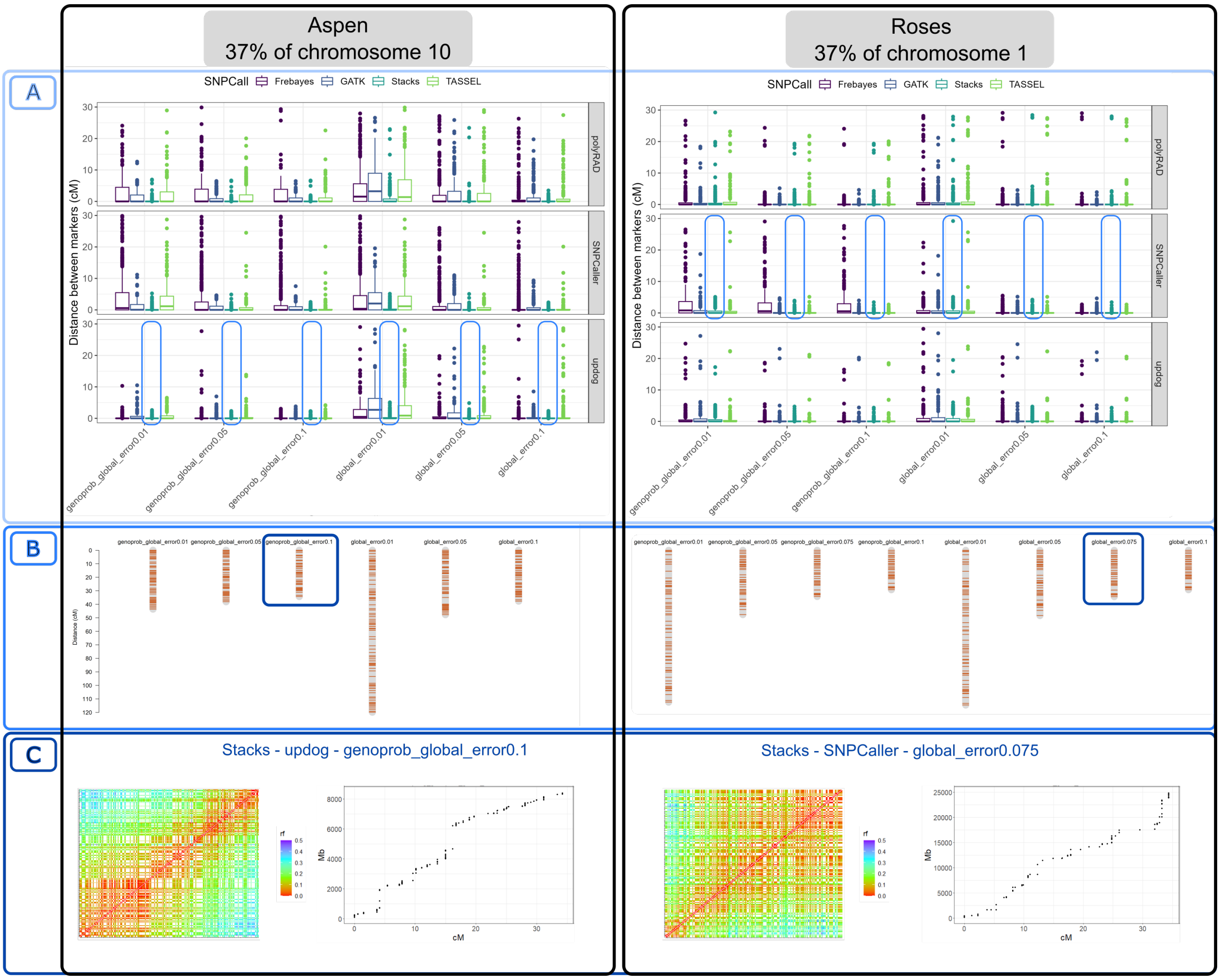

Figure 5. Process of selecting best pipeline: A) Comparing the effect of different error probabilities in the `OneMap` 3.0 HMM in the distances between adjacent markers; B) Comparing the effect of different error probabilities in the linkage maps total size built with a single SNP call software; C) Checking the recombination fraction (rf) heatmap and markers coverage in the genome using the selected pipeline. These figures were extracted from `Reads2MapApp`.

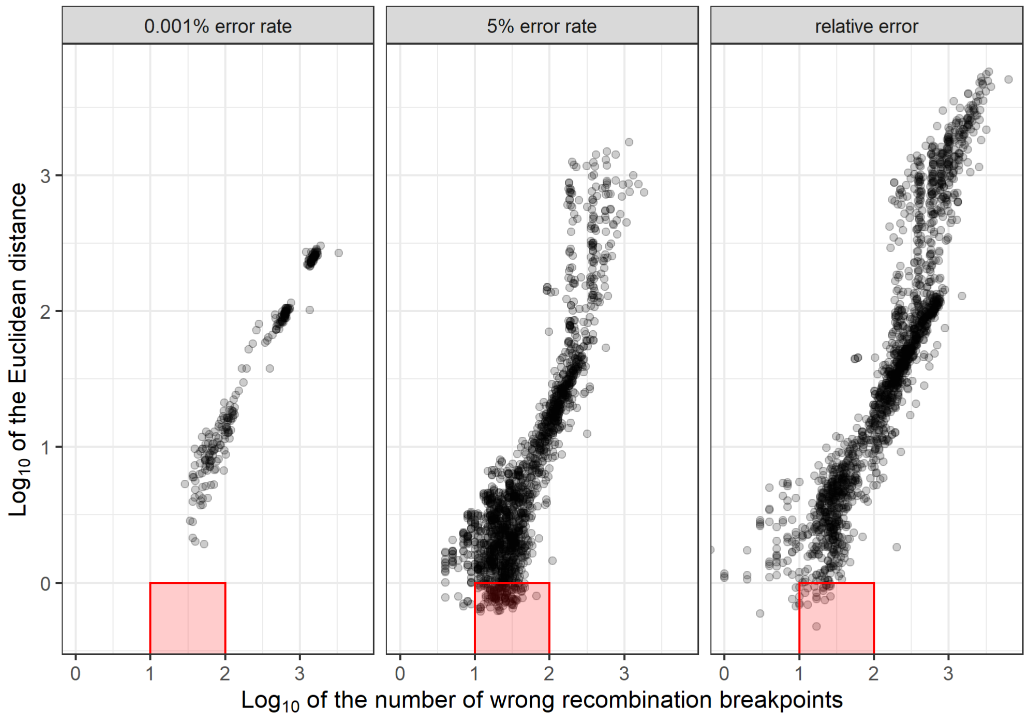

Figure 6. Relation between Euclidean distance (y-axis) and the number of recombination breakpoints (x-axis) in maps built with global error rates (0.001% and 5%), and with probabilities outputted by the genotype call software (relative error). Each dot represents a map built with simulated data based on the first 37% of aspen chromosome 10. The red squares highlight maps that do not present inflated size (1 or less Euclidean distance) but have from 10 to 100 wrong recombination breakpoints.

missing linkage information between markers D1 and D2 (homozygous x heterozygous or vice-versa). These markers can only be related to each other in the presence of more informative markers, such as B3.7 (heterozygous x heterozygous) or multiallelic states. Yet, having few B7.3 markers compared to D1 and D2 can still be an issue for linkage map building. In fact, this characteristic was the reason behind the initial development of separate maps for each parent in the first methods used for building genetic maps in such populations [62]. These non-integrated genetic maps subsequently limited further analysis of multiallelic traits in terms QTL mapping [63].

The markers ordering efficiency is not considered by `Reads2Map` workflows once it uses the genomic order to position the markers in the linkage maps. The reference genome is a required input by the workflows to standardize the positions of the markers across all tested methods. This avoids the confounding interpretation of bad-quality linkage maps due to wrong ordering and not genotyping errors.

To test the effect of multiallelic markers in the ordering, we built a linkage map for the entire chromosome 1 and 10 of the roses and aspen datasets, respectively, using the selected methods and adding the haplotype-based multiallelic markers provided by `Stacks population` plugin. We used the `OneMap` wrapper function mds_onemap to order the markers with MDS [57]. The genetic distances were estimated by HMM multipoint approach. Figure 7 shows the effects of including the multiallelic markers in the two-points-based MDS algorithm.

The impact of multiallelic markers differed between the aspen and roses datasets. In the aspen dataset, characterized by a lower depth and a higher rate of genotyping errors in the markers, most of the B3.7 biallelic markers were filtered out during previous steps, resulting in an unsatisfactory performance of the MDS algorithm in ordering the markers. However, incorporating the multiallelic markers, although slightly inflating the genetic distances, significantly improved the ordering accuracy using MDS. It should be noted that MDS itself can contribute to genetic distance inflation as it may erroneously invert markers in close proximity. In scenarios where a reference genome is unavailable, the inclusion of multiallelic markers can prove valuable for effective marker ordering in these types of datasets.

The rose dataset is characterized by higher-quality markers, and the genomic ordering can be almost entirely reproduced using only biallelic markers. In this scenario, the inclusion of multiallelic markers also leads to a slight inflation of the map size while improving the ordering accuracy through MDS. Unlike the aspen dataset, the MDS algorithm in the rose dataset tends to reduce the genetic distances, resulting in an underestimation of recombination breakpoints. However, considering that there are no significant inversions or translocations (see dot plots in figure 7), we can have more confidence in the genomic order, even if the map is larger. Any discrepancies between the MDS-based order and the genomic order are likely attributed to local changes, which are likely to be errors introduced by MDS.

Final considerations

The `Reads2Map` workflows have a robust structure to generate production-level results with simple inputs and optimized usage of computational resources. The structure allowed us to test the quality of genetic maps built with the following scenarios: i) using different SNP calling software (`GATK`, `TASSEL`, `Stacks`, and `Freebayes`); ii) using different genotype calling software (`GATK`, `Freebayes`, `TASSEL`, `Stacks`, `updog`, `polyRAD`, `SuperMASSA`); iii) using different linkage map building software (`OneMap` 3.0 and `GUSMap`); iv) establishing different error probabilities (relative to genotype call software, 10%, 1%, 5%, and 0.001% global error, and the combination of the global error rate with the genotype call probabilities); v) applying different marker filtering; vi) with or without multiallelic markers; vi) in empirical and simulated data; vii) with and without segregation distortion; viii) with and without contaminant samples; ix) with different GBS library preparation aspects; and x) with differ-

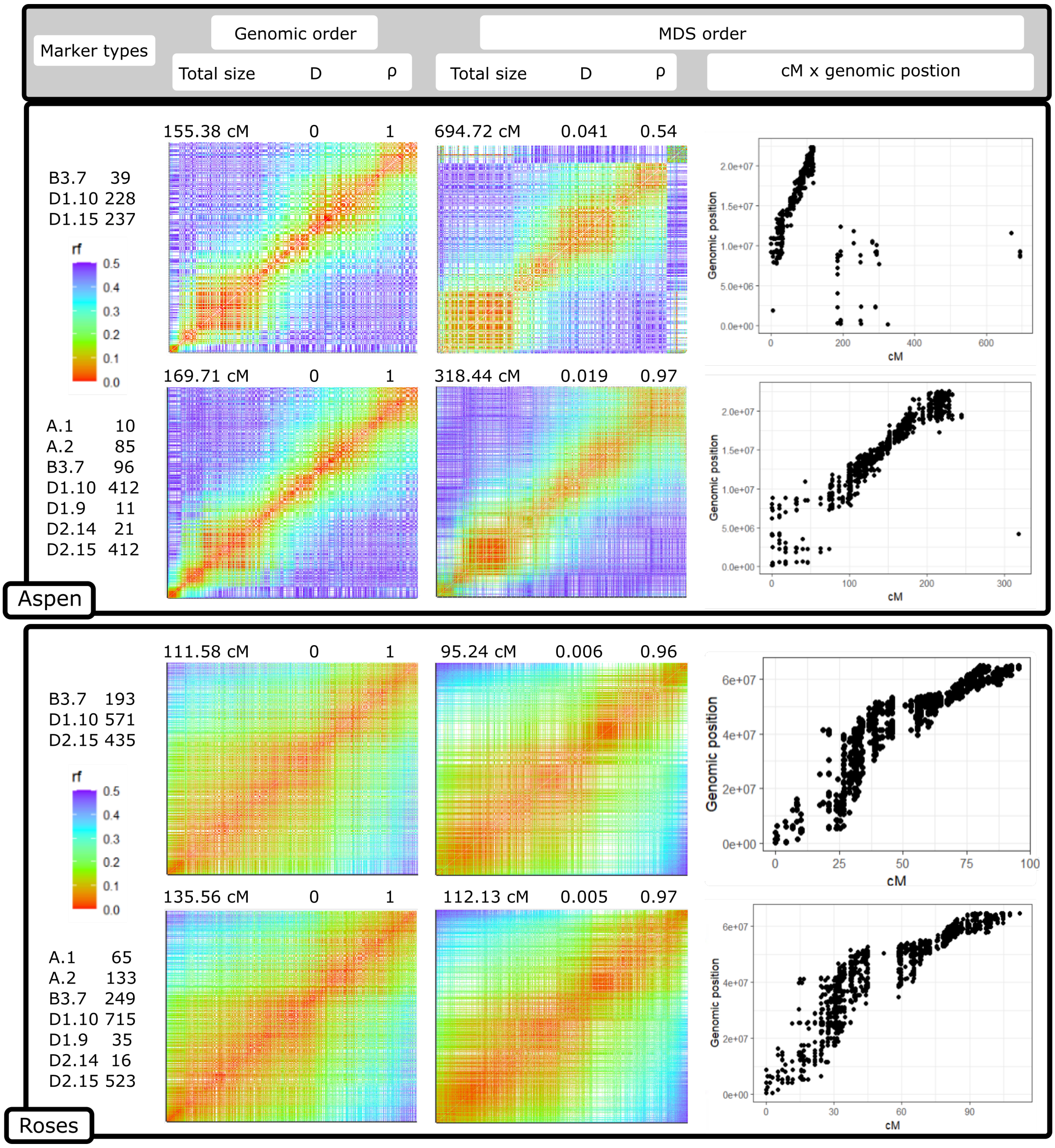

Figure 7. Comparison between MDS ordering algorithm performance in the aspen and rose dataset entire linkage group 10 and 1, respectively with only biallelic markers, and with biallelic and haplotype-based multiallelic markers estimated by `Stacks`. The heatmaps represent the recombination fraction (rf) matrix between markers positioned at both axes. In well-ordered linkage groups, we expect a gradient from hot colors in the diagonal (adjacent markers) to cold colors in the upper left and lower right corners. The figure also presents the Spearman rank correlation (ρ) and the Euclidean distances (D) between the estimated map using MDS and the map built with markers ordered by the genomic positions (used as reference). The dot plots relate the positions of markers estimated by MDS with the genomic position.

ent sequencing depths. These scenarios are commonly found by researchers trying to produce high-quality linkage maps using sequencing technologies. The Reads2Map and Reads2MapApp are the first tools to guide best practices for building linkage maps with sequencing data pointing software, parameters, and marker filters to be used in diverse scenarios.

We elaborated and limited the scenarios explored according to our experiences as developers of OneMap. OneMap first version was released in 2007, and since then it has been used to build linkage maps in a diversity of species. Its strategies and structure also served as a base for more complex software such as MAPpoly [15] for building linkage maps in polyploid species. With time, new methods for genetic marker identification using sequencing data emerged, changing the context where OneMap was used. We included updates in this version 3.0 to resolve issues with inflated genetic maps and marker ordering. Two major changes allow users to read and build genetic maps with the genotype probabilities and haplotype-based multiallelic markers information from the input files (OneMap format or VCF file). However, the success of genetic map building will be proportional to the quality of the information provided by upstream procedures such as library preparation, SNP and genotype calling, genotype probabilities estimation, and the combination of SNPs into haplotype-based markers. With Reads2Map and Reads2MapApp, we provide users tools to select the best approaches before using OneMap 3.0 to guarantee that it will result in the best quality genetic map possible with the data available.

It is important to highlight that we did not design the workflows to be a tool to build a final linkage map but to select the bioinformatic pipeline that provides the best quality genetic markers. Once the pipeline is selected, the respective VCF file and OneMap functions can be used in the R environment to build the final map. Building the complete linkage map will require evaluations and edits that are highly specific and cannot be fully automated within the workflows. These tasks include addressing the presence of translocations and inversions, identifying outlier markers, and linkage between markers located in different chromosomes.

The diversity in the results of the pipeline suggested for both empirical datasets highlights that pipelines perform differently with datasets with different properties. This means that the pipelines presented here as the best cannot be considered the best for every dataset. We could reduce the number of required tests by users identifying the dataset-independent approaches and setting them as default in Reads2Map. However, we suggest users reproduce the tests presented here for the dataset-dependent approaches using the Reads2Map workflows with their empirical dataset and select the best pipelines for their specific conditions.

The workflows were built using WDL and containers to ensure high reproducibility. This guarantees that different results running different datasets is due to the dataset's properties and not to bioinformatic pipeline changes. Also, updates can be easily made in the workflows as the software implemented are improved once the versions are controlled by Docker images. This makes Reads2Map also a useful tool for software developers to validate updates because it facilitates checking the consequences of the changes in the quality of the markers by easily controlling versions, rerunning datasets, and checking the map quality.

Every Reads2Map workflow run returns a large amount of information. Every step of the workflow, from the reads' alignment to the completed linkage map, provides quality measurements for users to evaluate each scenario. The Reads2MapApp shiny app receives all this information compressed in a single workflow output file and converts it into comprehensive interactive graphics. Through the app interface, users can evaluate the performance of each combination of software and parameters in each step. If results show issues in any of them, users can re-run the workflow with adapted parameters or include new filters that make sense in their context. Once established the upstream steps based on the app graphics for the built linkage map subset, users can reproduce it for the complete dataset, inputting the VCF files from Reads2Map into OneMap.

Availability of source code and requirements

- Project name: Reads2Map
- Project home page: https://github.com/Cristianetaniguti/Reads2Map
- Main workflows: EmpiricalReads2Map [32] and SimulatedReads2Map [33]
- Operating system(s): Platform independent
- Programming language: WDL
- Other requirements: docker or singularity
- License: GNU GPL

Additional files

Supplementary File 1. Emission function for outcrossing.

Supplementary File 2. Reads2MapApp interface demonstration.

Supplementary Table S8. List of third-party software and images versions used

Supplementary Figure S7. Venn diagrams show the number of markers identified by freebayes, GATK, and simulated (true). The intersection between the data sets represents markers with the same position in the reference genome *Populus trichocarpa* version 3.0. The Empirical data sets include markers spread across the entire reference genome. The simulations only include markers in the first 8.426 Mb of chromosome 10 (2.1% of the genome). The mean and standard deviation of number markers are shown for the simulated data set once the simulation and SNP calling are repeated 60 times. Markers were filtered by 25% maximum missing data and MAF 5% in empirical and simulated data. * Number of markers common to all 60 repetitions.

Supplementary Figure S8. The relation between filters applied (x-axis), the map size (A y-axis), and the number of markers (B y-axis) for genotype calling software used in the empirical data sets. The data sets shown in the figure contain only biallelic markers. The horizontal red line indicates the expected map size (38 cM) for the subset of the genomes used.

Supplementary Figure S9. ROC curves with the true and estimated genotypes from the five families simulated with mean depth 10 and 20 and the first 8.426 Mb of the chromosome 10 (37% or 38 cM). Here only biallelic markers are considered. The specificity and sensitivity profiles consider different thresholds in the genotype probabilities for each scenario. The higher the area under the curve, the higher the genotype's probability reliability. Genotype probabilities thresholds closer to the left superior corner have a higher capacity to differentiate right and wrong genotypes.

Supplementary Figure S10. Supplementary Figure S9 continued.

Supplementary Figure S11. Effect of contaminant samples in the map size (A) and in the number of estimated recombination breakpoints range (B) among progeny individuals. The empirical aspen data sets presented in this figure contain multiallelic markers, the allele counts from the VCF file, and is filtered by genotype probability higher than 0.8 to keep only informative markers.

Abbreviations

GBS: Genotyping-by-Sequencing; PCR: polymerase chain reaction; RADSeq: Restriction-site associated; DNA sequencing; VCF: variant call format; GQ: genotyping quality; GT: genotype; GWAS: genome-wide association; SNP: single nucleotide polymorphism; LD: linkage disequilibrium; QTL: quantitative trait loci; WDL: workflow description language; HPRC: high-performance research comput-

ing; CPU: central processing unit; HMM: hidden Markov model; EM: expectation-maximization; MAF: minor allele frequency; NGS: Next Generation Sequencing.

Competing Interests

The authors declare that they have no competing interests.

Funding

This work was partially supported by the National Council for Scientific and Technological Development (CNPq - 313269/2021-1); by USDA, National Institute of Food and Agriculture (NIFA), Specialty Crop Research Initiative (SCRI) project "Tools for Genomics-Assisted Breeding in Polyploids: Development of a Community Resource" (Award No. 2020-51181-32156); and by the Bill and Melinda Gates Foundation (OPP1213329) project SweetGAINS. TPO acknowledges funding from the European Union's Horizon 2020 research and innovation program under the Marie Sklodowska-Curie grant agreement No. 801215 and the University of Edinburgh Data-Driven Innovation program as part of the Edinburgh and South East Scotland City Region Deal.

Author's Contributions

CHT, MM, RRA, AAFG, GSP and GCF contributed to `OneMap` package updates. CHT, LMT, GSG, GSP, AAGF, MM, ORL, and JL contributed with ideas to design `Reads2Map`. CHT and LMT developed and optimized the `Reads2Map` code. CHT developed `Reads2MapApp`. CHT, TPO, AAFG, ORL, DB, and RRA contributed to elaborate the tested scenarios. CHT, TPO, and RRA contributed to analyzing the results. CHT wrote the first version of the manuscript. All authors provided helpful discussions for the work and reviewed the manuscript.

Acknowledgements

Portions of this research were conducted with the advanced computing resources provided by Texas A&M High-Performance Research Computing and by the University of São Paulo Aguia High-Performance Computing. We also thank David Gerard for the idea of using genotype probabilities from `updog` combined with a global error rate.

References

1. Glaubitz JC, Casstevens TM, Lu F, Harriman J, Elshire RJ, Sun Q, et al. TASSEL-GBS: a high capacity genotyping by sequencing analysis pipeline. PLoS ONE 2014 2;9:1–11.
2. Catchen J, Hohenlohe PA, Bassham S, Amores A, Cresko WA. Stacks: an analysis tool set for population genomics. Molecular Ecology 2013;22:3124–40.
3. Anderson CB, Franzmayr BK, Hong SW, Larking AC, Stijn TC, Tan R, et al. Protocol: A versatile, inexpensive, high-throughput plant genomic DNA extraction method suitable for genotyping-by-sequencing. Plant Methods 2018 8;14.
4. Andrews KR, Good JM, Miller MR, Luikart G, Hohenlohe PA. Harnessing the power of RADseq for ecological and evolutionary genomics. Nature Reviews Genetics 2016 1;17:81–92.
5. Bresadola L, Link V, Buerkle CA, Lexer C, Wegmann D. Estimating and accounting for genotyping errors in RAD-seq experiments. Molecular Ecology Resources 2020;20:856–870.
6. Baird NA, Etter PD, Atwood TS, Currey MC, Shiver AL, Lewis ZA, et al. Rapid SNP Discovery and Genetic Mapping Using Sequenced RAD Markers. PLoS ONE 2008;3:e3376.
7. Elshire RJ, Glaubitz JC, Sun Q, Poland JA, Kawamoto K, Buckler ES, et al. A robust, simple genotyping-by-sequencing (GBS) approach for high diversity species. PLoS ONE 2011 5;6:e19379.
8. der Auwera GV, O'Connor B. Genomics in the Cloud: Using Docker, GATK, and WDL in Terra. O'Reilly Media, Incorporated; 2020.
9. Rivera-Colón AG, Rochette NC, Catchen JM. Simulation with RADinitio improves RADseq experimental design and sheds light on sources of missing data. Molecular Ecology Resources 2020;p. 1–16.
10. Gerard D, Ferrão LFV, Garcia AAF, Stephens M. Genotyping Polyploids from Messy Sequencing Data. Genetics 2018 11;210:789–807.
11. a Hackett C, Broadfoot LB. Effects of genotyping errors, missing values and segregation distortion in molecular marker data on the construction of linkage maps. Heredity 2003 1;90:33–38.
12. Sturtevant AH. The behavior of the chromosomes as studied through linkage. Zeitschrift für induktive Abstammungs- und Vererbungslehre 1915;13:234–287.
13. Smith GR, Nambiar M. New Solutions to Old Problems: Molecular Mechanisms of Meiotic Crossover Control. Trends in Genetics 2020;36:337–346.
14. Bilton TP, Schofield MR, Black MA, Chagné D, Wilcox PL, Dodds KG. Accounting for Errors in Low Coverage High-Throughput Sequencing Data When Constructing Genetic Maps Using Biparental Outcrossed Populations. Genetics 2018 5;209:65–76.
15. Mollinari M, Garcia AAF. Linkage Analysis and Haplotype Phasing in Experimental Autopolyploid Populations with High Ploidy Level Using Hidden Markov Models. G3: Genes|Genomes|Genetics 2019 10;9:3297–3314.
16. Liao Y, Voorrips RE, Bourke PM, Tumino G, Arens P, Visser RGF, et al. Using probabilistic genotypes in linkage analysis of polyploids. Theoretical and Applied Genetics 2021 8;134:2443–2457.
17. Margarido GRA, Souza AP, Garcia AAF. OneMap: software for genetic mapping in outcrossing species. Hereditas 2007 7;144:78–9.
18. Lander ES, Green P. Construction of multilocus genetic linkage maps in humans. Proc Natl Acad Sci USA 1987;84:2363–2367.
19. Lorenz AJ, Hamblin MT, Jannink JL. Performance of single nucleotide polymorphisms versus haplotypes for genome-wide association analysis in barley. PLoS ONE 2010;5:1–11.
20. Gawenda I, Thorwarth P, Günther T, Ordon F, Schmid KJ. Genome-wide association studies in elite varieties of German winter barley using single-marker and haplotype-based methods. Plant Breeding 2015 2;134:28–39.
21. N'Diaye A, Haile JK, Fowler DB, Ammar K, Pozniak CJ. Effect of Co-segregating Markers on High-Density Genetic Maps and Prediction of Map Expansion Using Machine Learning Algorithms. Frontiers in Plant Science 2017 8;8.
22. Sehgal D, Dreisigacker S. Haplotypes-based genetic analysis: Benefits and challenges. Vavilovskii Zhurnal Genetiki i Selektsii 2019;23:803–808.
23. Abed A, Belzile F. Comparing Single-SNP, Multi-SNP, and Haplotype-Based Approaches in Association Studies for Major Traits in Barley. The Plant Genome 2019;12:190036.
24. Liu N, Zhang K, in Genetics Zhao HBTA. Haplotype-Association Analysis. Genetic Dissection of Complex Traits 2008;60:335–405.
25. Jiang Y, Schmidt RH, Reif JC. Haplotype-Based Genome-Wide Prediction Models Exploit Local Epistatic Interactions Among Markers. G3: Genes|Genomes|Genetics 2018;49:g3.300548.2017.
26. Garrison E, Marth G. Haplotype-based variant detection from short-read sequencing. ArXiv e-prints 2012;p. 9.
27. McKenna A, Hanna M, Banks E, Sivachenko A, Cibulskis K, Kernytsky A, et al. The Genome Analysis Toolkit: A MapReduce framework for analyzing next-generation DNA sequencing data. Genome Research 2010 9;20:1297–1303.
28. Clark LV, Lipka AE, Sacks EJ. polyRAD: Genotype Calling with Uncertainty from Sequencing Data in Polyploids and Diploids. G3: Genes|Genomes|Genetics 2019;9:g3.200913.2018.
29. Serang O, Mollinari M, Garcia AAF. Efficient exact maximum a posteriori computation for bayesian SNP genotyping in polyploids. PLoS ONE 2012;7:1–13.
30. Patterson M, Marschall T, Pisanti N, van Iersel L, Stougie L, Klau GW, et al. WhatsHap: Weighted Haplotype Assembly for Future-Generation Sequencing Reads. Journal of Computational Biology 2015;22:498–509.
31. Voss K, Gentry J, Auwera GVD. Full-stack genomics pipelining with GATK4+ WDL+ Cromwell [version 1; not peer reviewed]. F1000Research 2017;p. 4.
32. Taniguti CH. EmpiricalReads2Map. WorkflowHub 2022;https://doi.org/10.48546/WORKFLOWHUB.WORKFLOW.409.1.
33. Taniguti CH. SimulatedReads2Map. WorkflowHub 2022;https://doi.org/10.48546/WORKFLOWHUB.WORKFLOW.410.1.
34. bio T. Terra: Focus on your science. Available online at: https://appterrabio/ 2020;.
35. Merkel D. Docker : Lightweight Linux Containers for Consistent Development and Deployment Docker : a Little Background Under the Hood. Linux Journal 2014;2014:2–7.
36. Kurtzer GM, Sochat V, Bauer MW. Singularity: Scientific containers for mobility of compute. PLOS ONE 2017 5;12:e0177459.
37. Li H. Aligning sequence reads, clone sequences and assembly contigs with BWA-MEM. ArXiv 2013;1303.
38. Li H, Handsaker B, Wysoker A, Fennell T, Ruan J, Homer N, et al. The sequence alignment/map format and SAMtools. Bioinformatics 2009;25:2078–2079.
39. Danecek P, Bonfield JK, Liddle J, Marshall J, Ohan V, Pollard MO, et al. Twelve years of SAMtools and BCFtools. GigaScience 2021 1;10.
40. Knaus BJ, Grünwald NJ. vcfR: a package to manipulate and visualize variant call format data in R. Molecular Ecology Resources 2017 1;17:44–53.
41. Baum E, Petrie T, G S, N W. A Maximization Technique Occurring in the Statistical Analysis of Probabilistic Functions of Markov Chains. The Annals of Mathematical Statistics 1970;41:164–171.
42. Schiffthaler B, Bernhardsson C, Ingvarsson PK, Street NR. BatchMap: A parallel implementation of the OneMap R package for fast computation of F1 linkage maps in outcrossing species. PLoS ONE 2017;12:1–12.
43. Guyader V, Fay C, Rochette S, Girard C. golem: A Framework for Robust Shiny Applications. Golem GitHub repository 2022;https://github.com/ThinkR-open/golem.

44. Zhigunov AV, Ulianich PS, Lebedeva MV, Chang PL, Nuzhdin SV, Potokina EK. Development of F1 hybrid population and the high-density linkage map for European aspen (Populus tremula L.) using RADseq technology. BMC Plant Biology 2017;17.
45. Young EL, Lau J, Bentley NB, Rawandoozi Z, Collins S, Windham MT, et al. Identification of QTLs for Reduced Susceptibility to Rose Rosette Disease in Diploid Roses. Pathogens 2022 6;11:660.
46. Tuskan GA, DiFazio S, Jansson S, Bohlmann J, Grigoriev I, Hellsten U, et al. The Genome of Black Cottonwood, Populus trichocarpa. Science 2006 9;313:1596–1604.
47. Saint-Oyant LH, Ruttink T, Hamama L, Kirov I, Lakhwani D, Zhou NN, et al. A high-quality genome sequence of Rosa chinensis to elucidate ornamental traits. Nature Plants 2018 7;4:473–484.
48. Martin M. Cutadapt removes adapter sequences from high-throughput sequencing reads. EMBnetjournal 2011 5;17:10.
49. Hyman JM. Accurate Monotonicity Preserving Cubic Interpolation. SIAM Journal on Scientific and Statistical Computing 1983 12;4:645–654.
50. Wu R, Ma CX, Wu SS, Zeng ZB. Linkage mapping of sex-specific differences. Genetical research 2002;79:85–96.
51. Voorrips RE, Maliepaard CA. The simulation of meiosis in diploid and tetraploid organisms using various genetic models. BMC Bioinformatics 2012 12;13:248.
52. Haldane JBS. The combination of linkage values, and the calculation of distance between linked factors. Journal of Genetics 1919;8:299–309.
53. Best K, Oakes T, Heather JM, Shawe-Taylor J, Chain B. Computational analysis of stochastic heterogeneity in PCR amplification efficiency revealed by single molecule barcoding. Scientific reports 2015 10;5:14629.
54. Glenn TC. Field guide to next-generation DNA sequencers. Molecular Ecology Resources 2011;11:759–769.
55. Li H. seqtk: Toolkit for processing sequences in FASTA/Q formats. seqtk GitHub repository 2020;https://github.com/lh3/seqtk.
56. Ros-Freixedes R, Gonen S, Gorjanc G, Hickey JM. A method for allocating low-coverage sequencing resources by targeting haplotypes rather than individuals. Genetics Selection Evolution 2017;49:1–17.
57. Preedy KF, Hackett CA. A rapid marker ordering approach for high-density genetic linkage maps in experimental autotetraploid populations using multidimensional scaling. Theoretical and Applied Genetics 2016;.
58. DePristo MA, Banks E, Poplin R, Garimella KV, Maguire JR, Hartl C, et al. A framework for variation discovery and genotyping using next-generation DNA sequencing data. Nature Genetics 2011;43:491–8.
59. Duncavage EJ, Coleman JF, de Baca ME, Kadri S, Leon A, Routbort M, et al. Recommendations for the Use of In silico Approaches for Next Generation Sequencing Bioinformatic Pipeline Validation: A Joint Report of the Association for Molecular Pathology, Association for Pathology Informatics, and College of American Pathologists. The Journal of molecular diagnostics : JMD 2022 10;.
60. Alexander DH, Novembre J, Lange K. Fast model-based estimation of ancestry in unrelated individuals. Genome Research 2009 9;19:1655–1664.
61. Amadeu RR, Cellon C, Olmstead JW, Garcia AAF, Resende MFR, Muñoz PR. AGHmatrix: R Package to Construct Relationship Matrices for Autotetraploid and Diploid Species: A Blueberry Example. The Plant Genome 2016 11;9.
62. Grattapaglia D, Sederoff R. Genetic linkage maps of Eucalyptus grandis and Eucalyptus urophylla using a pseudo-testcross: mapping strategy and RAPD markers. Genetics 1994 8;137:1121–37.
63. Gazaffi R, Margarido GRA, Pastina MM, Mollinari M, Garcia AAF. A model for quantitative trait loci mapping, linkage phase, and segregation pattern estimation for a full-sib progeny. Tree Genetics and Genomes 2014;10:791–801.

Figure
Click here to access/download;Figure;SNPcalling_eff.png

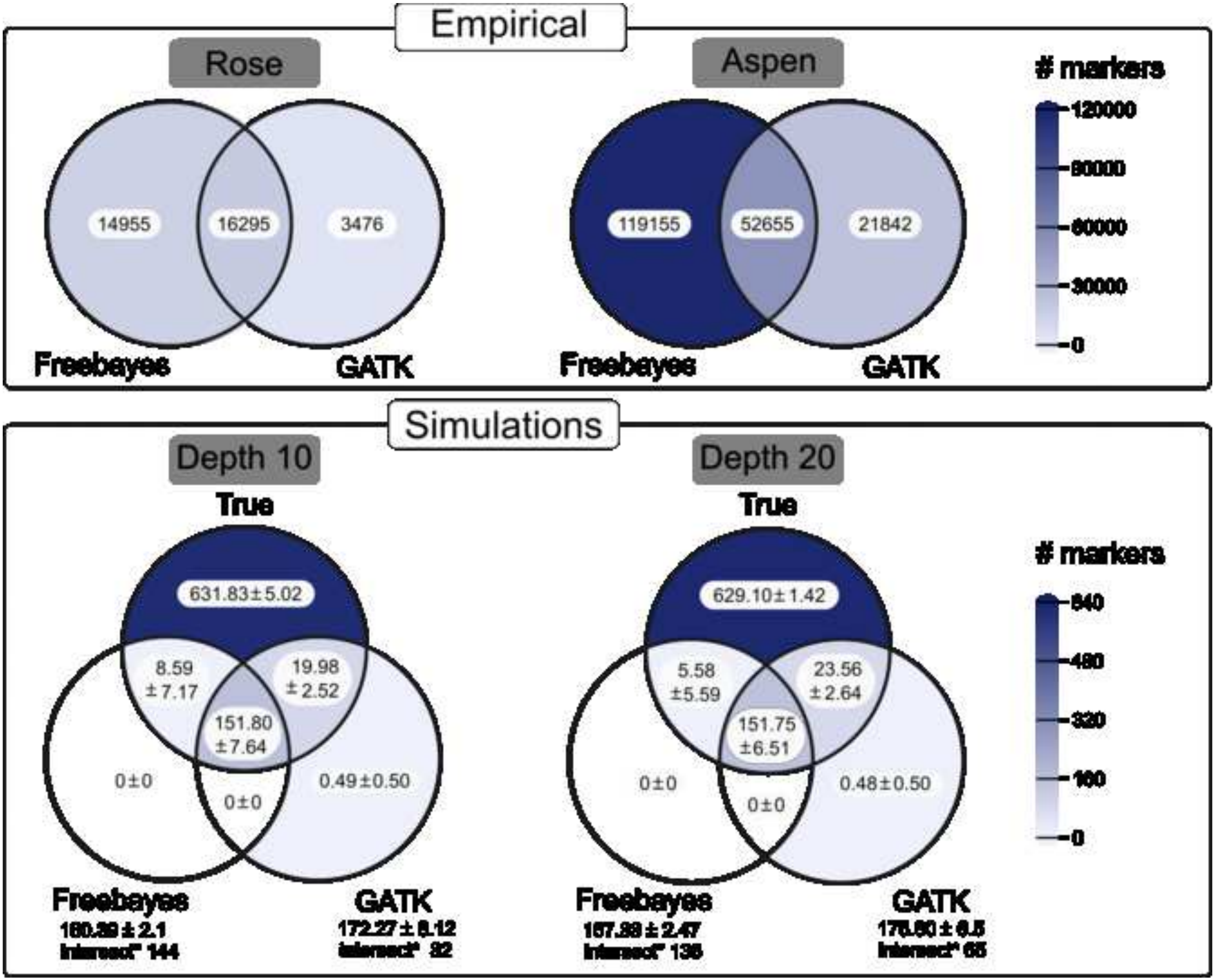

Figure

Click here to access/download;Figure;filters_paper_emp.png

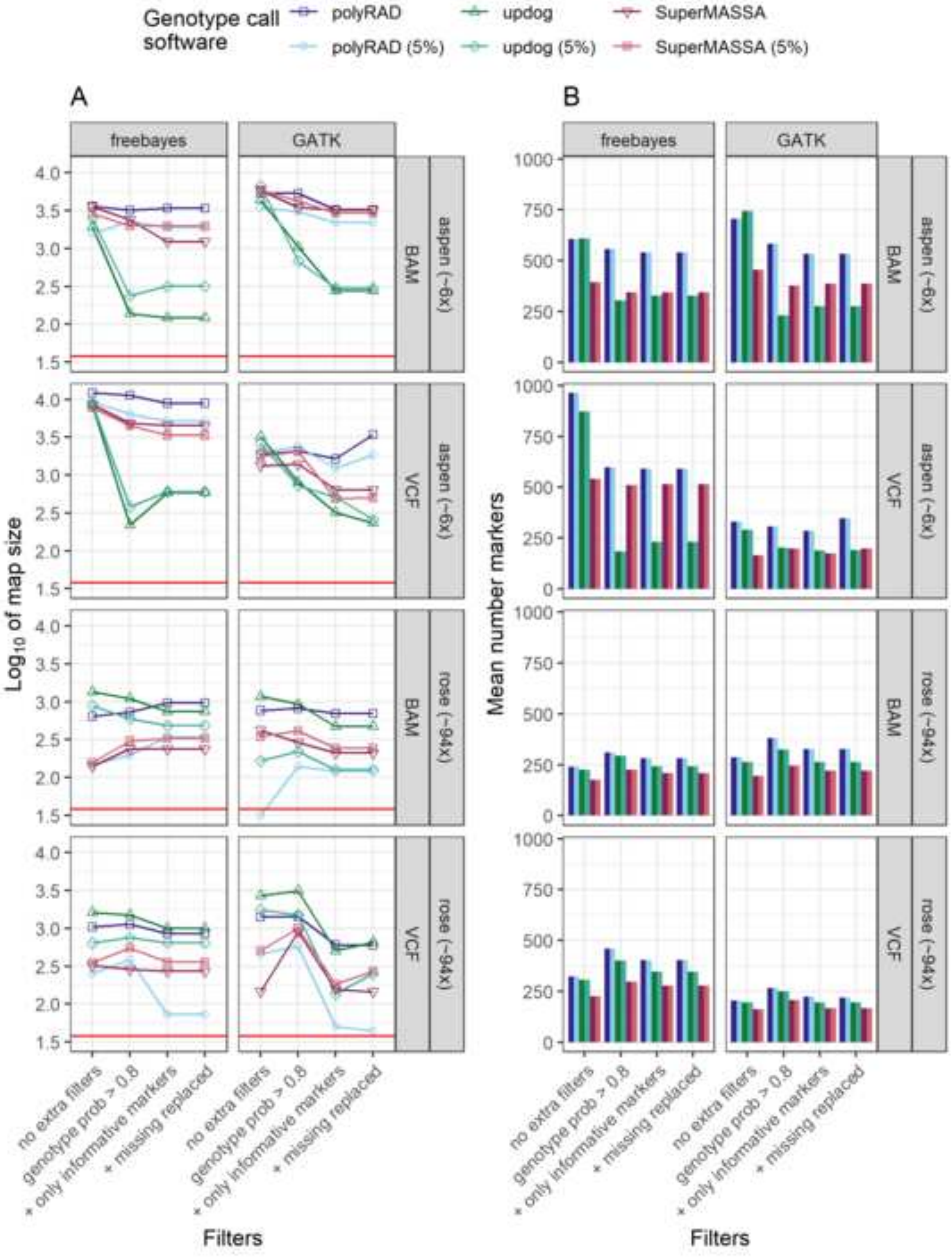

Figure

Click here to access/download;Figure;noncontxcont_paper_emp.png

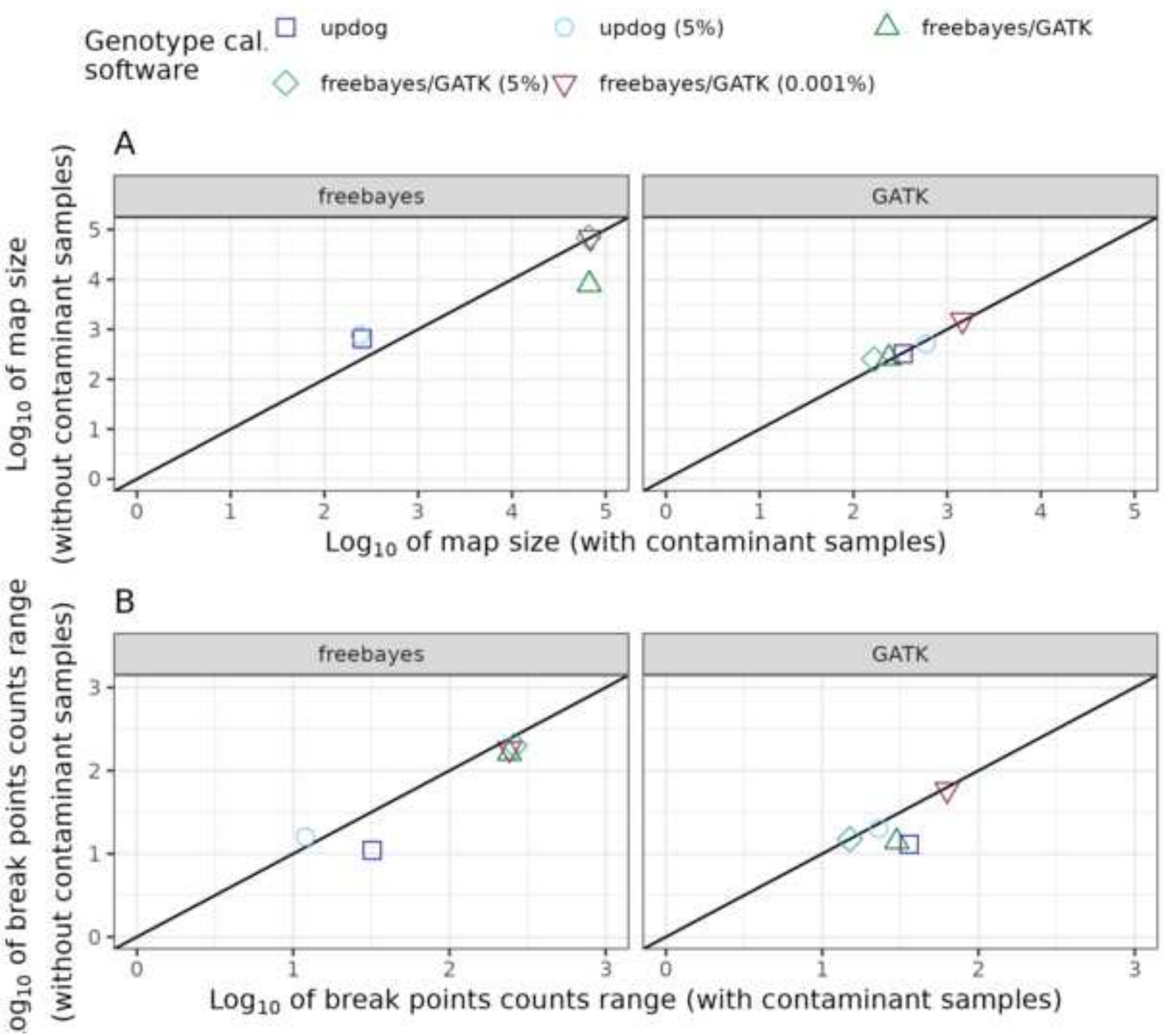

Figure

Click here to access/download;Figure;roc10.png

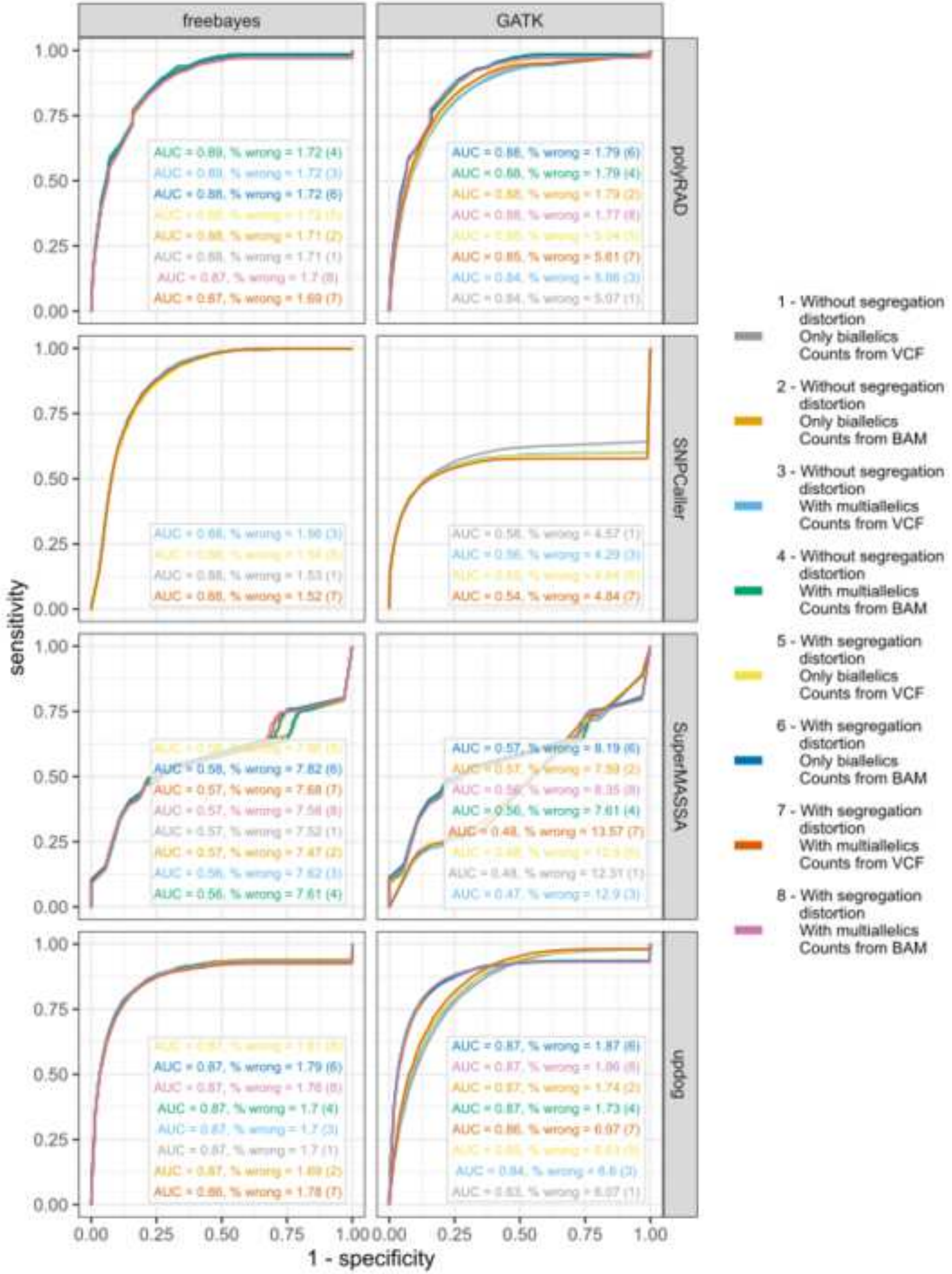

Figure

Click here to access/download;Figure;roc20.png

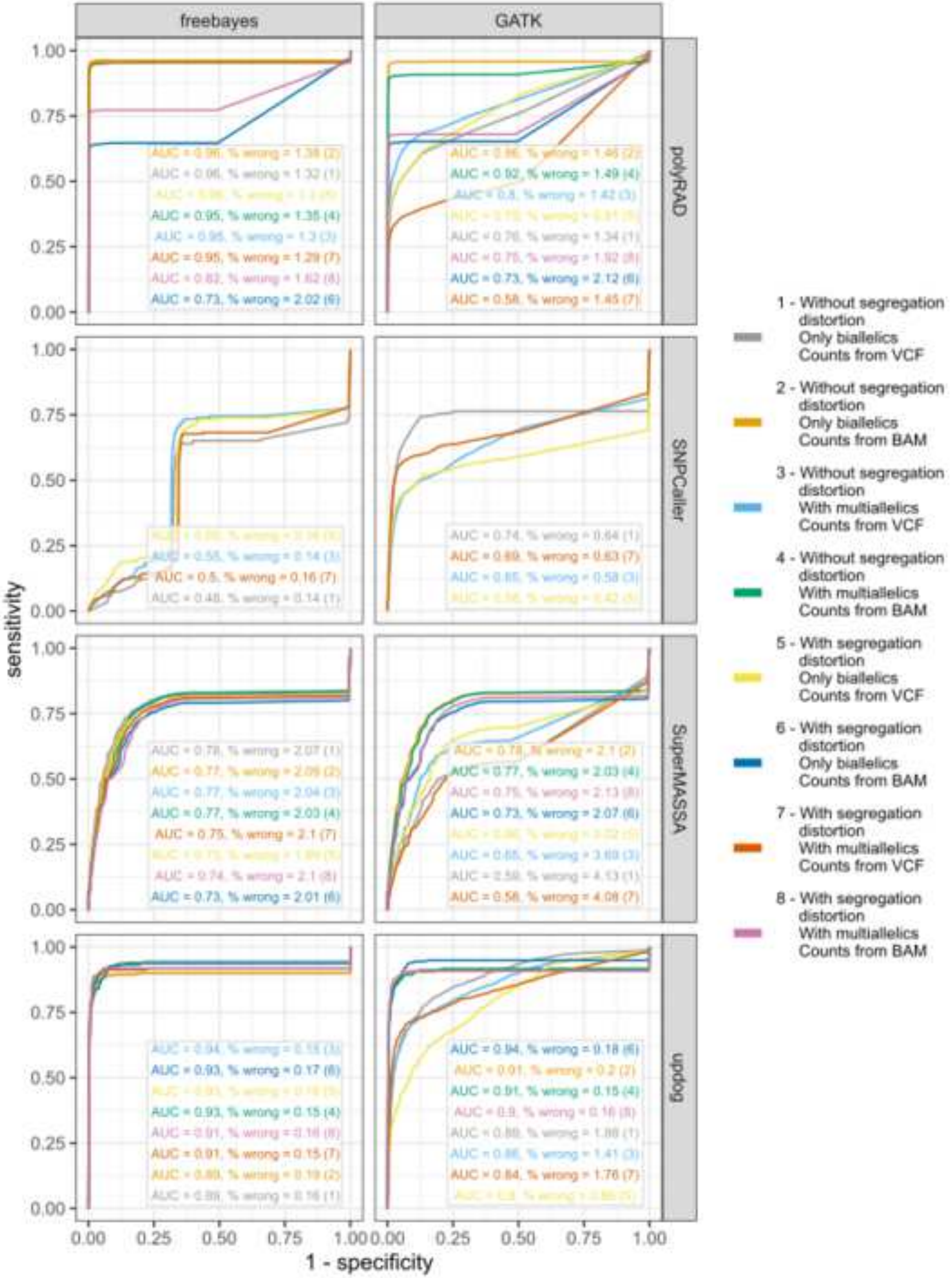

Click here to access/download;Figure;app2.png

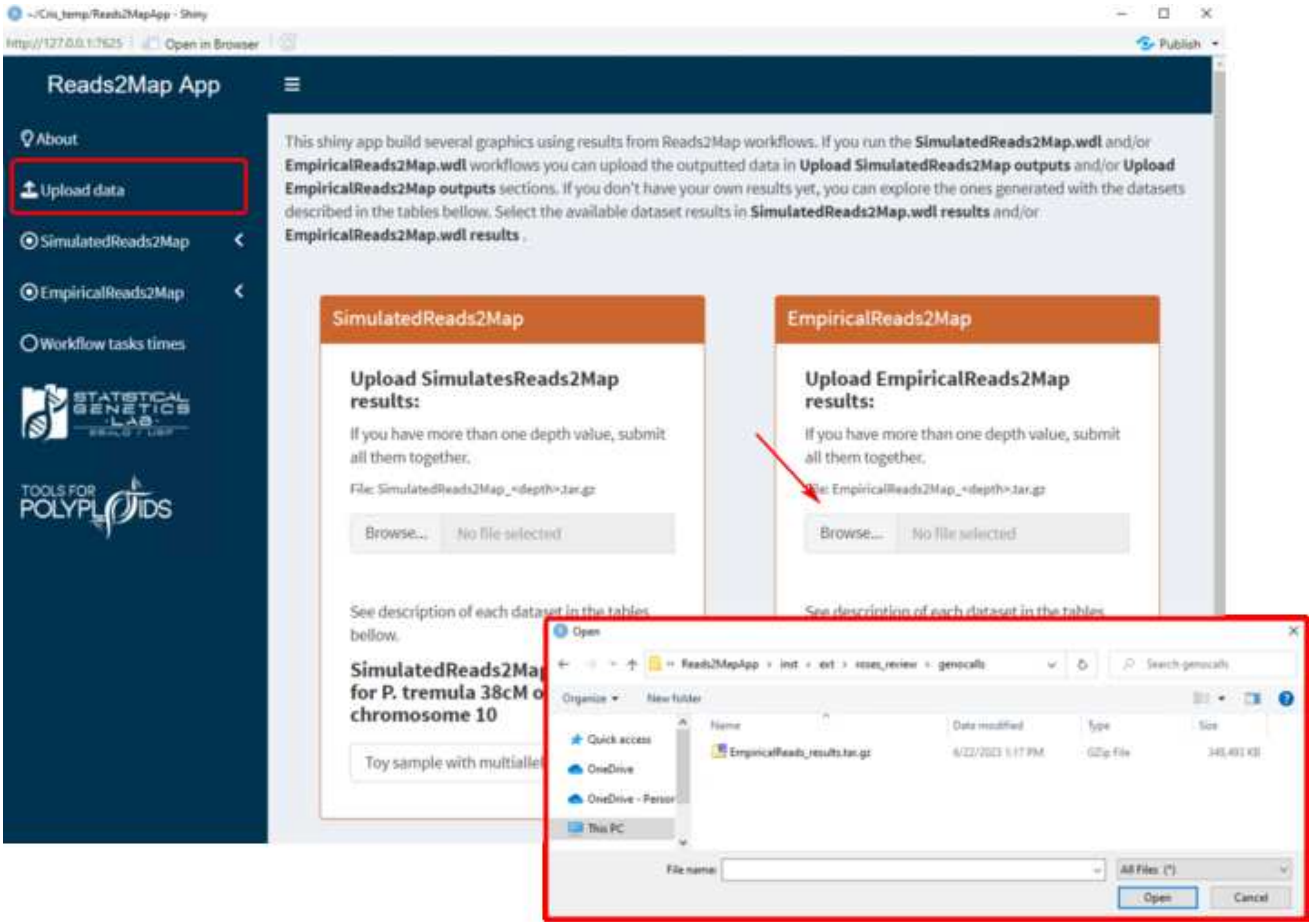

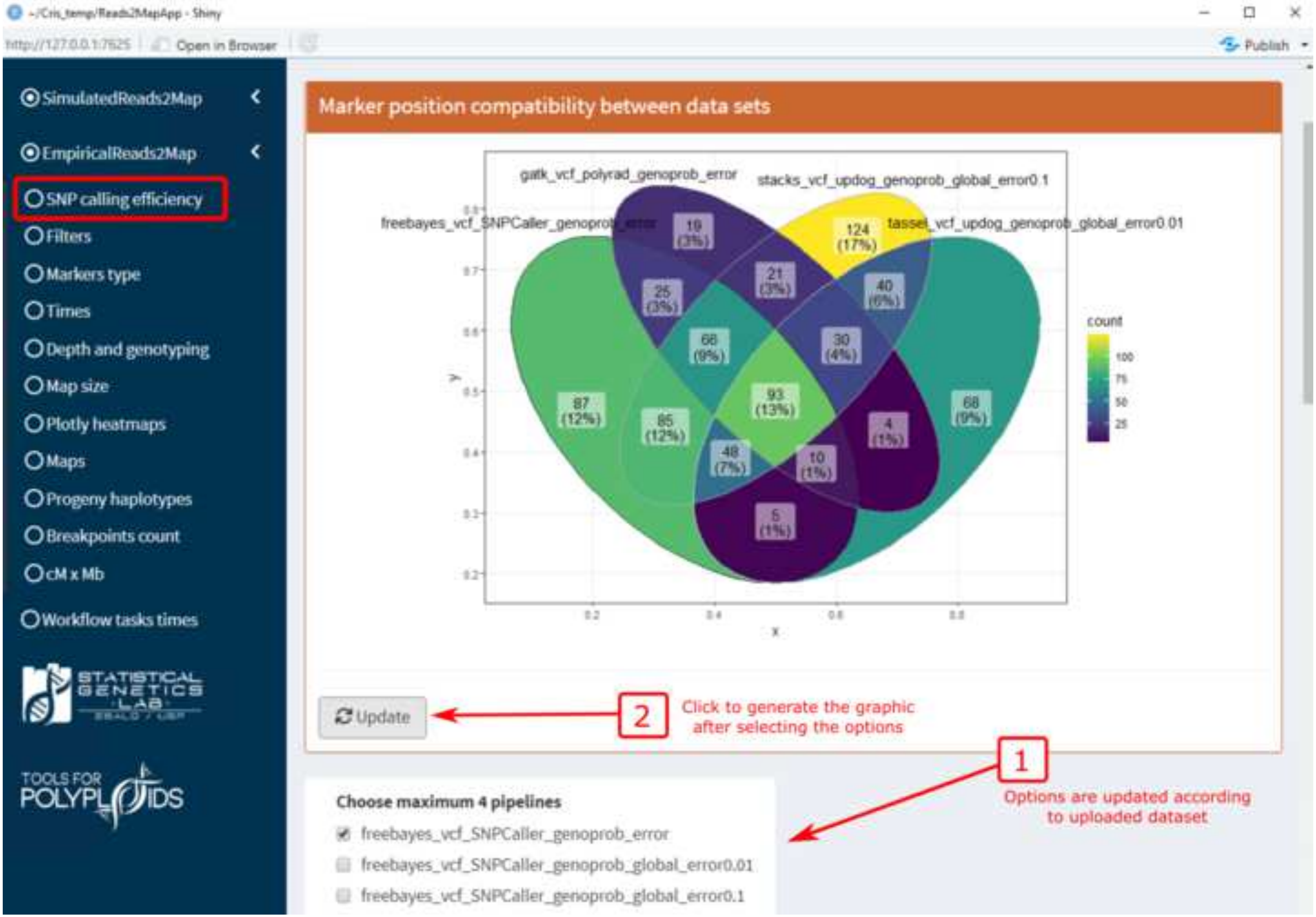
SimulatedReads2Map
EmpiricalReads2Map
SNP calling efficiency
Filters
Markers type
Times
Depth and genotyping
Map size
Plotly heatmaps
Maps
Progeny haplotypes
Breakpoints count
cM x Mb
Workflow tasks times
Marker position compatibility between data sets
gatk_vcf_polyrad_genoprob_error
stacks_vcf_updog_genoprob_global_error0.1
freebayes_vcf_SNPCaller_genoprob_error
tassel_vcf_updog_genoprob_global_error0.01
count
Update
2
Click to generate the graphic after selecting the options
1
Options are updated according to uploaded dataset
Choose maximum 4 pipelines
freebayes_vcf_SNPCaller_genoprob_error
freebayes_vcf_SNPCaller_genoprob_global_error0.01
freebayes_vcf_SNPCaller_genoprob_global_error0.1

Figure

Click here to access/download;Figure;app4.png

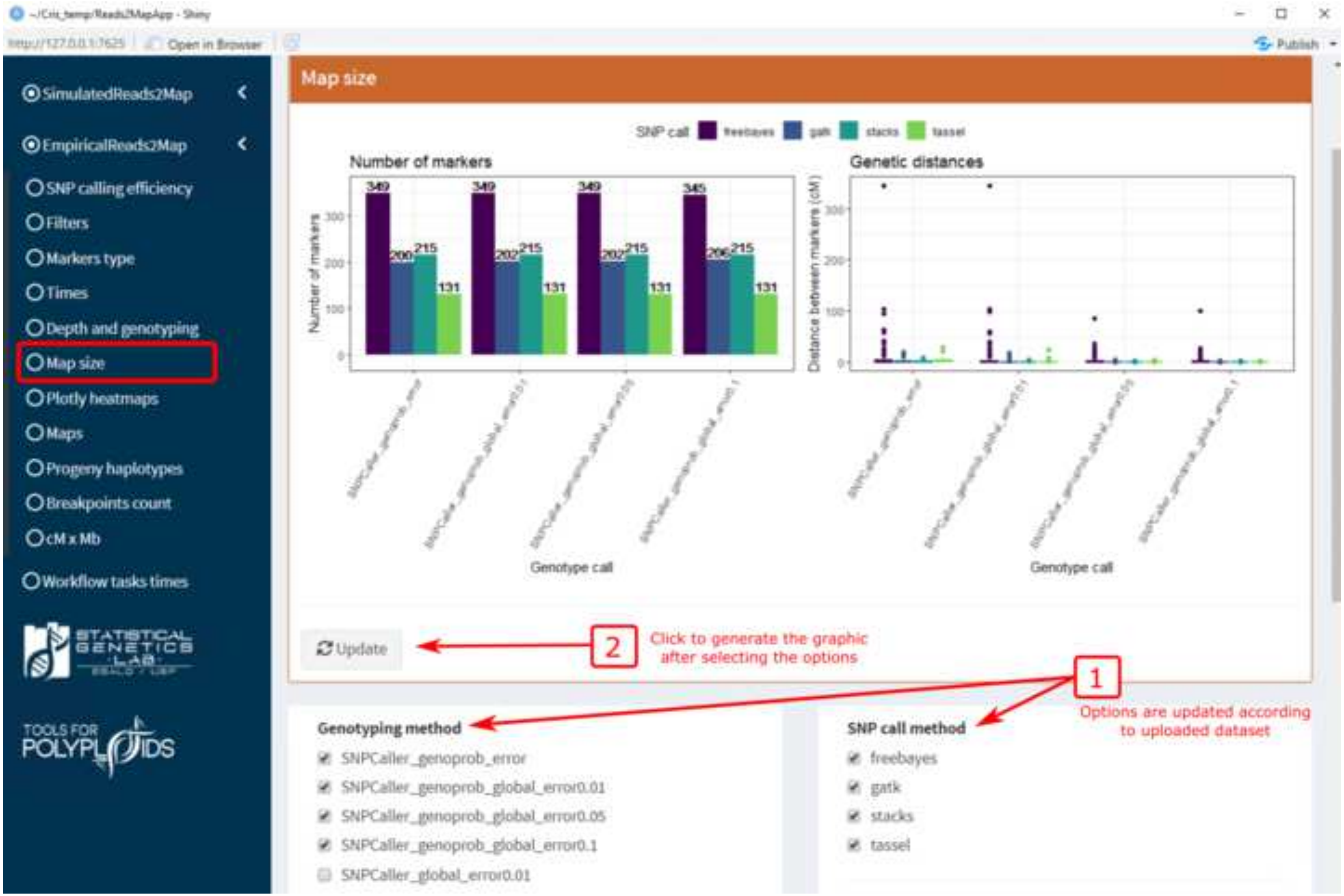

Click here to access/download;Figure;app5.png

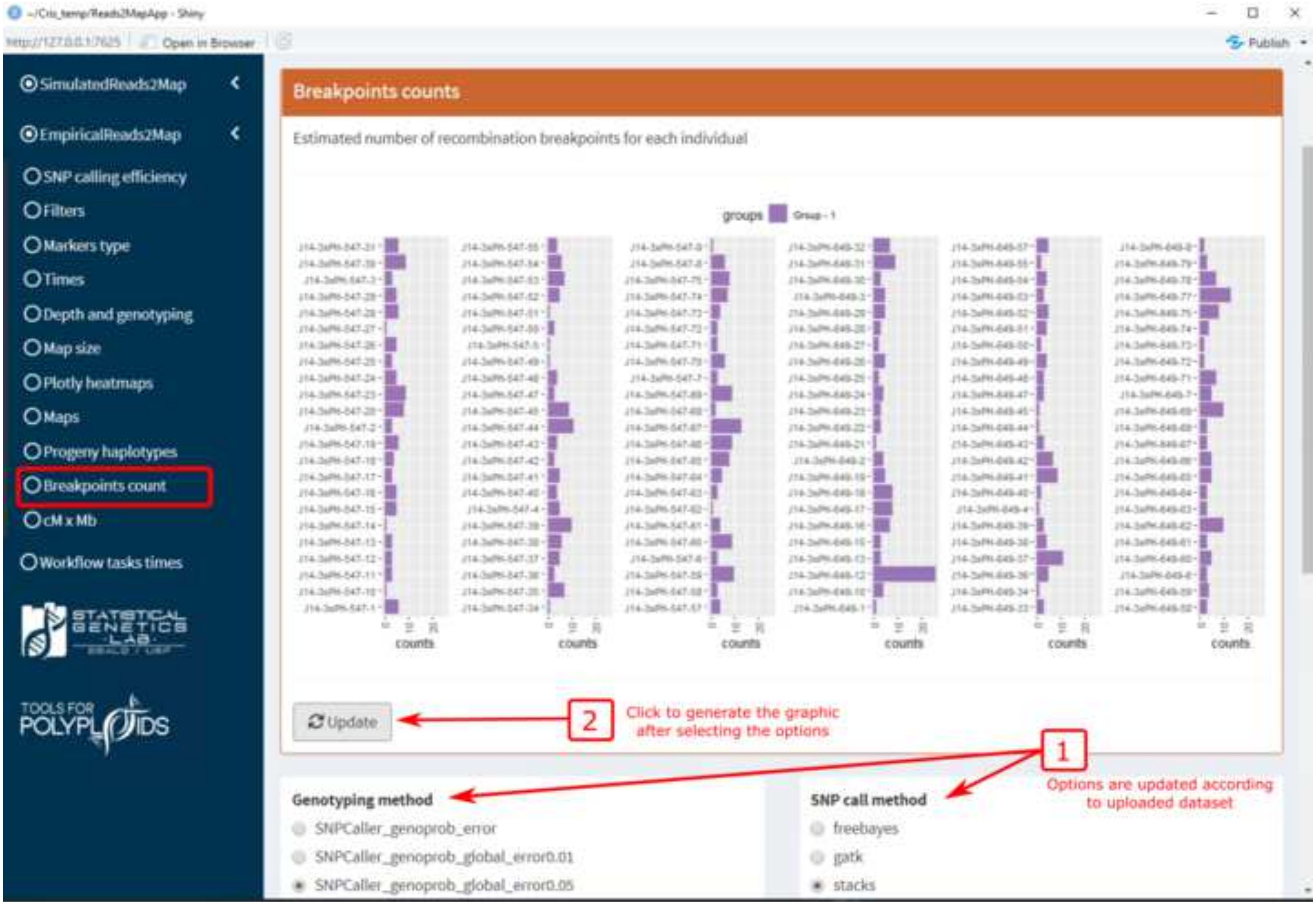

Figure

Click here to access/download;Figure;app6.png

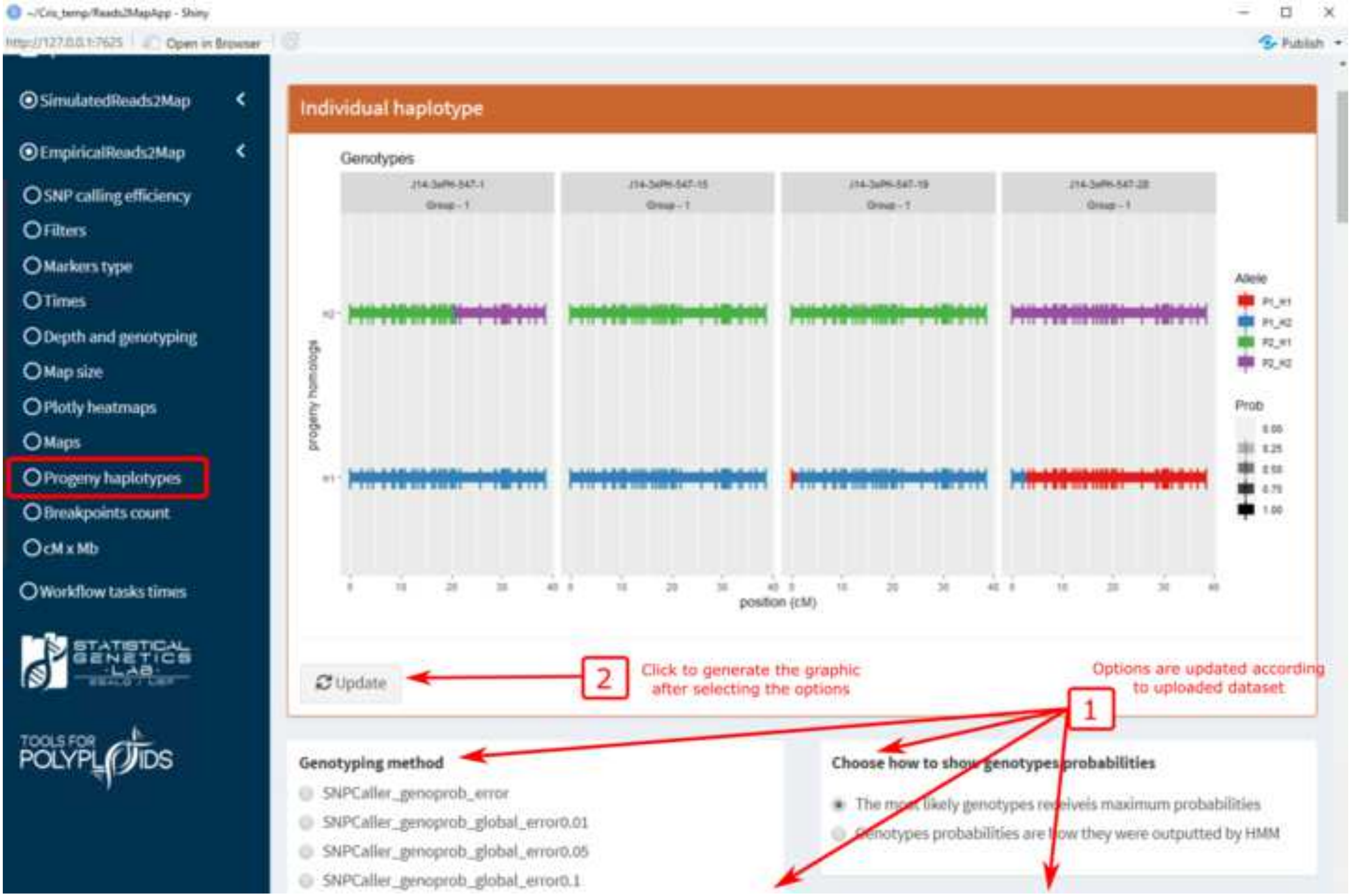

Figure

Click here to access/download;Figure;app1.png

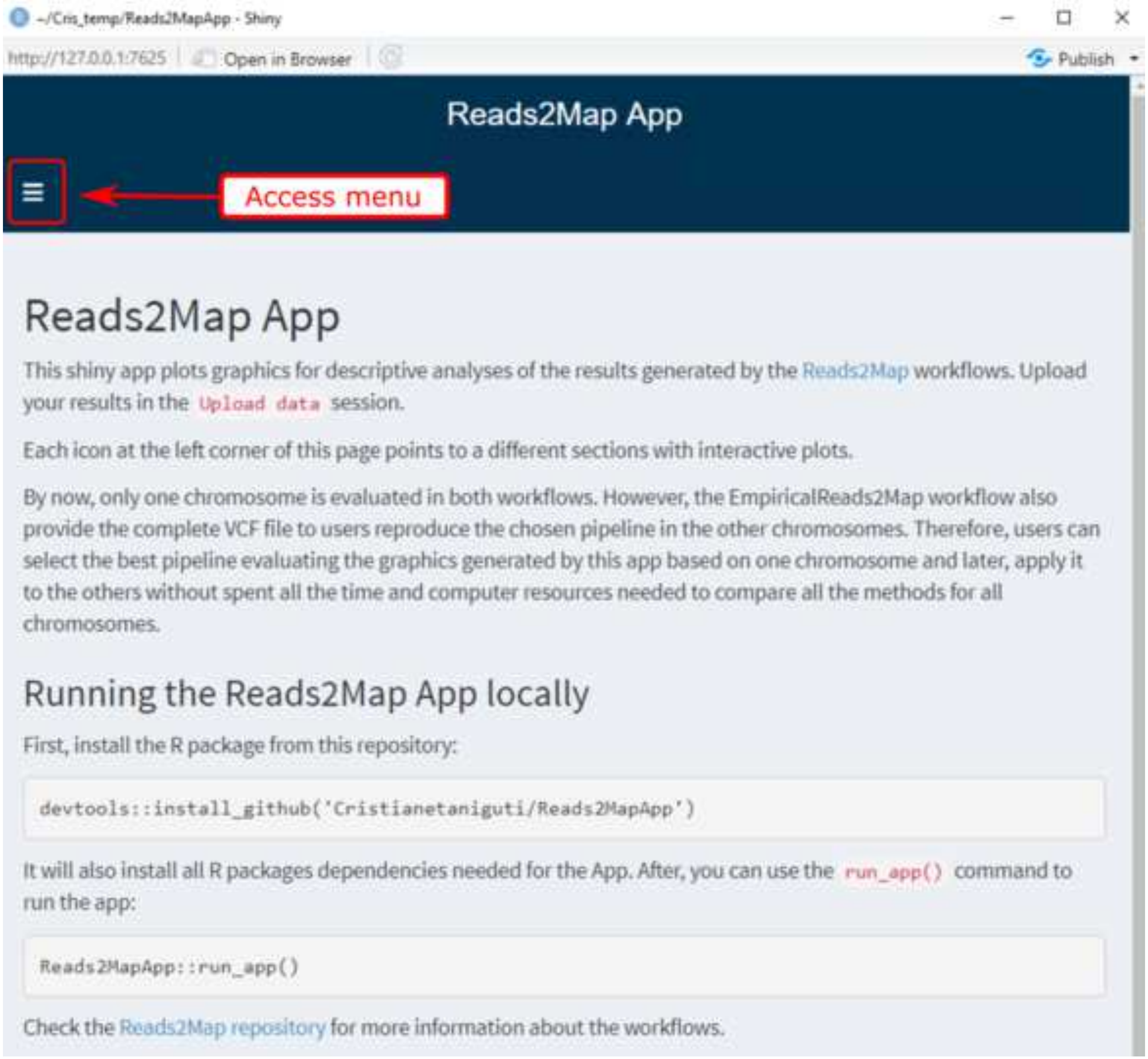

Manuscript_with_lineNumbers

Click here to access/download
Supplementary Material
main_lines.pdf

Click here to access/download
Supplementary Material
Supplementary_material.pdf
