# Supplementary material for: Developing best practices for genotyping-by-sequencing analysis in the construction of linkage maps
Source: Gigascience. 2023 Oct 27;12:giad092. doi: 10.1093/gigascience/giad092 (PMC10603770; doi:10.1093/gigascience/giad092)
Supplement: giad092_Response_to_Reviewer_Comments_Original_Submission [file giad092_response_to_reviewer_comments_original_submission.pdf]

# Author's Response To Reviewer Comments

Reviewer #1: I read with interest the manuscript on Reads2Map, a really impressive amount of work went into this and I congratulate the authors on it. However, it is precisely this almost excessive amount of results that for me was the major drawback with this paper. I got lost in all the detail, and therefore I have suggested a Major Revision to reflect that I think the paper could be somehow made more stream lined with a clearer central message and fewer figures in the text. Line numbers would have been helpful, I have tried to give the best indication of page number and position, but in future @GigaScience please stick to line numbers for reviewers, it's a pain in the neck without them. Overall I think this is an excellent manuscript of general interest to anyone working in genomics, and definitely worthy of publication.

Answer: Thanks for your review. I addressed the detailed comment below. To facilitate this next review, I included a version of the manuscript with line numbers.

General comment: if a user would like to use GBS data for other population types than those amenable for linkage mapping (e.g. GWAS or genomic prediction, so a diversity panel or a breeding panel), how could your tool be useful for them?

Answer: The first steps of the workflow that include the alignment with BWA and SNP calling with GATK and freebayes (now also with TASSEL and STACKS options) can be applied to any population type. Because the workflows are partitioned into sub-workflows and tasks, these steps can be run independently of the dosage calling and linkage map building which require mapping populations. We separated the EmpiricalReads2Map into EmpiricalSNPCalling and EmpiricalMaps to emphasize this difference. We also added a short explanation in the manuscript (lines 478-493).

Answer: Another way of applying the tool for non-mapping populations is if the GBS library is producing and sequencing mapping populations and non-mapping populations in the same experiment. In this situation, the results obtained for the mapping populations using Reads2Map can be extrapolated to the other populations without mapping structure.

Other general comment: the manuscript is long with an exhaustive amount of figures and supplementary materials. Does it really need to be this detailed? It appears like the authors lost the run of themselves a little bit and tried to cram everything in, and in doing so risk losing the point of the endeavour. What is the central message of this manuscript? Regarding the figures, the reader cannot refer to the figures easily as they are now mainly contained on another page. Do you really need Figures 16-18 for example? Figures 13 and 14 could be combined perhaps? I am sure that at most 10 figures and maybe even less are needed in the main text, otherwise figures will always be on different pages and hence lose their impact in the text call-out.

Answer: We reduced the text and figures.

Abstract and page 4: "global error rate of 0.05" - How do you motivate the use of a global error rate of 5%? Surely this is dataset-dependent?

Answer: We conduct new tests with different values and added figure number 5 to guide users on how to select a proper value. During this review, we talked to updog developer (David Gerard), who gave us the idea of combining the global error with the software genotype probability. We did that using 1-(1-genotype error probability)x(1-global error), which proved to be a good option too.

Page 4 - how can a user estimate an error per marker per individual? The description of the create_probs function suggests there is an automatic methodology to do this, but I don't see it described. You could perhaps refer to Zheng et al's software polyOrigin, which actually locally optimises the error prior per datapoint. Maybe something for the discussion.

Answer: The error probabilities used are not estimated by OneMap but by the upstream genotype calling

software (VCF PL value of HaplotypeCaller, Freebayes, TASSEL, STACKs and genotype probabilities of updog, polyRAD, and SuperMASSA). The idea of doing that is to take into account issues that were found by the upstream bioinformatic process such as low depth, dispersion of the read counts, and alignment quality. Thanks for highlighting the polyOrigin method, if I understood right, it takes into account only the genotypes to estimate this error rate, it is not based on the bioinformatic features for each. We kept linkage map polyploid tools out of the scope of this work to not make it longer than already is, but we are already working to add MAPpoly as a new option to build the maps. MAPpoly contains a similar approach to control the errors as implemented in OneMap. While doing this, we can perform tests to compare with PolyOrigin approach.

Page 6 "recombination fraction giving the genomic order" do you mean "given"?
Answer: Yes. Thanks.

Page 10 section Effects of contaminant samples - if you look at Figure 9 you can see that the presence of contaminant samples seems to have an impact on the genotypes of other, non-contaminant samples, especially using GATK and 5% global error. With the contaminants present, the number of XO points decreases in many other samples. This is very odd behaviour I would have thought. Is it known whether this apparent suppresion of recombination breakpoints in non-contaminant individuals is likely to be "correct"? Perhaps the SNP caller was running under the assumption that all individuals were part of the same F1? If the SNP caller was run without this assumption (eg. specifying only HW equilibrium, or model-free) would we still see the same effect? This is for me a quite worrying result but something that you make no reference to as far as I can tell.

Answer: The GATK was not used applying an F1 assumption, but the linkage map was built considering that. The multipoint approach tries to fit the contaminant sample by redistributing the recombination breaks. This issue is emphasized while using higher values of global error because we decrease the trust in the observed genotype and increase the model assumptions. It is indeed a concerning result. We added lines 623-629 to warn users to remove contaminant samples before the linkage map building.

Page 12 "Effects of segregation distortion" In your study you only considered a single linkage group. One of the primary issues with segregation distortion in mapping is that it can lead to linkage disequilibrium between chromosomes, if selection has occurred on multiple loci. This can then lead to false linkages across linkage groups. Perhaps good to mention this.

Answer: Interesting. Added in lines 710-717 .

Page 12 "have difficulty missing linkage information" - missing word "with"

Page 17 I see no mention of the impact of errors in the multi-allelic markers on the efficiency, particularly of order_seq which seems to be very poorly-performing with only bi-allelics (Fig 20). If bi-allelic SNPs have errors then it is not obvious why multi-SNP haplotypes should not also have errors.

Answer: The multiallelic markers do have errors and they have a higher effect on the estimation of the genetic distances. We updated the figure about the effects of the multiallelics now including the HMM error rate and the rose dataset. We also decide to remove order_seq algorithm evaluations because it took a long time to process and the result was not better than MDS. We updated the discussion about it in lines 630-678.

Page 3 Figure 1 - here the workflow shows multiple options for a number of the steps, which can lead to the creation of many map variants (e.g. 816 maps as mentioned on Page 4). Should all users produce 816 variants of their maps? With potentially millions of markers, this is going to take a huge amount of time (most users will want 100% of all chromosomes, not 37% of a single chromosome). Or should this be done for only a subset of markers? What if there is no reference sequence available to select a subset? As there are no clear recommendations, I suspect that the specific combination of pipeline choices will usually be dataset-dependent. You actually mention this in the discussion page 17. And with only 2 real datasets from 2 different species, there is also no way to tell if eg. GATK works best in rose, or updog should be used for

monocots but not dicots etc. It would be helpful if the authors were more explicit about how their tool informs "best practices for GBS analysis" for ordinary users. Perhaps it is there, but for me this message gets lost.

Answer: We run many maps in this work to test our ideas about what could be possibly causing bad-quality linkage maps. E.g.: different upstream software, presence, and absence of multiallelic markers, contaminants, segregation distortion, and filters. Some of our conclusions we do not consider dataset dependent such as the lower performance of SuperMASSA and GUSMap (they also apparently are not being updated anymore), the usage of a filter instead of counts from BAM, usage of multiallelic markers and best filters to be applied. These were set as default in the workflows (we clarify this in lines 470-477 and Table 3). Therefore, users do not need to repeat all our tests for every dataset.

Answer: If the user wants to run using a single combination of SNP and genotype calling resulting in a single linkage map it is also possible. This can be set in the workflow input file. The need for subsetting the dataset would depend on the number of tests the user wants to perform and the computational capacity available. It is important to highlight that we did not design the workflow to be a tool to build a final linkage map but to select the bioinformatic pipeline that provides the best quality markers. The SNP and genotype calling are always made for the entire dataset. The subsetting is only required for the linkage map build step, once the HMM approach is a slow process. Once the pipeline is selected, the VCF file with markers for the entire dataset is already available for users to repeat the process in other chromosomes using the R environment and OneMap functions. We describe this suggestion of usage in lines 234-241 and lines 710-717.

Answer: In terms of software used, the results are not only dataset-dependent but also version dependent, as most of the software implemented here is still being actively developed. Although it would require more bioinformatic skills, users can also test their own hypotheses, change software versions, or include new software.

Answer: By now, having a reference genome close enough to the species to determine the markers belonging to each chromosome is required for workflow usage. This requirement was highlighted in lines 649-653.

Page 17 "updates in this version 3.0 to resolve issues with inflated genetic maps" - if I look at Figure 20, it seems that issues with inflated map length have not yet been fully resolved!

Answer: The figure was made to highlight the improvements of multiallelic markers in the ordering process, but we used the OneMap default global error to estimate the distances. We rerun the analysis using the markers resulting from the selected pipeline.

Page 17 "we provide users tools to select the best approaches" - similar comment as before - does this mean users should build > 800 maps with a subset of their dataset first, and then use this single approach for the whole dataset? It is not explicitly stated whether this is the guidance given. What is the eventual aim - to produce a good linkage map, or to use the linkage map to critically compare genotyping tools?

Answer: Reads2Map can be useful in both cases. To build a good linkage map, users need to have good quality markers, and selecting the bioinformatic approach which provides them is essential. As mentioned above, Reads2Map was not designed to directly build a final linkage map but to select the pipeline. The total number of maps generated by it will depend on the tests users wants to make. Currently, using the default parameters, the workflow will generate 12 maps for the user-defined subset.

Answer: With the goal of comparing genotyping tools, Reads2Map is also useful for developers to validate updates, because it facilitates checking the consequences of the changes in the quality of the markers by easily controlling versions, rerunning datasets, and checking the map quality (added in lines 725-731). One example of it is that during this review, updog developer implemented a new method to try to overcome the updog issues identified in this work. We re-run some of our tests to give him feedback on the update impact (see the GitHub issue for details: https://github.com/dcgerard/updog/issues/19).

Reviewer #2: The paper titled "Developing best practices for genotyping-by-sequencing analysis using linkage maps as benchmarks" aims to present an end to end workflow uses GBS genotyping datasets to generate genetic linkage maps. This is a valuable tool for geneticists intending to generate a high confidence linkage map from a mapping population with GBS data as input.

I got confused on reading the MS though, is this a workflow paper or is this a review of the component software for each step of genetic mapping and how parameter/use differences affect the output ? If it's a review, then the choice of software reviewed are not comprehensive enough, esp on SNP calling, and linkage mapping.

Answer: The idea is not to do a review but to provide tools and guidelines for building a good quality linkage map in different situations. We changed the text to streamline our findings according to our tests and we set defaults in the workflow to reduce the number of required tests by users.

There is no clear justification why each component software was used,example the use of GATK and freebayes for SNP calling I am familiar with using TASSEL GBS and STACKS for SNP calling using GBS data, why weren't they included in the SNP calling software.

Answer: We agree. We implemented both software for the SNP calling and perform new tests in empirical datasets. We updated the text to include the results from them.

The MS would benefit greatly from including these SNP calling software in their benchmarking.Onemap and gusmap seems also pre-selected for linkage mapping, without reason for use, or maybe the reason(s) were not highlighted in the text. I've had experience in the venerable MAPMAKER and MSTMap, and would like to see more comparisons of the chosen genetic linkage mapping software with others, if this is the intent of the MS.

Answer: MAPMAKER and MSTMap as well as ASMap are not able to build linkage maps for the highly heterozygous populations (full-sib or outcrossing populations) evaluated in this work. Other software such as Lep-MAP and JoinMap are able to build linkage maps for outcrossing but do not present a method to account for aspects of the genotype calling (e.g. read depth distribution) in their genetic distance estimation such GUSMap and OneMap do. Also, JoinMap is not open-access.

The MS also clearly focuses on genetic linkage mapping using GBS, which should be more explicitly stated in the title. GBS is also extensively used in diversity collections and there is scant mention of this in the MS, and whether the workflow could be adapted to such populations.

Answer: Similar to the first answer given to reviewer #1. We added an explanation about how Reads2Map can be applied to other sequencing library types in lines 478-493.

Versions of software used in the workflow are also not explicitly stated within the MS.

Answer: Because there are many used software and libraries versions, we described in the Reads2Map repository README only the docker images used and their versions. Some of the used images are available online and the Dockerfile describing the software and the versions that they contain can be found in their repository. Other images used were built by us and the Dockerfile for them can be found in their DockerHub repository or in the Reads2Map GitHub repository (directory .dockerfiles). The image used by each task of the workflow is indicated in the WDL runtime. We added Table 8 in the Supplementary Material with a list of docker images version used for the results presented.

The shiny app is also not demonstrated well in the MS, it could be presented better with screenshots of the interface, with one or two sample use cases.

Answer: We clarify in the figures caption that they were obtained through the app and we also added

screenshots in Supplementary File 2.

Reviewer #3: In this MS, the authors tried to develop a framework for using GBS data for downstream analysis and reduce the impact of sequence errors caused by GBS. However, sequence error is an issue not specific to GBS, it is also for whole genome sequences. Actually, I think the major issue for GBS is the missing data. However, in this MS, the authors did not test the impact of missing data on downstream analysis.

Answer: The work does not focus only on sequence errors but on genotype errors which can be caused also by other sources (e.g. such as low depth, PCR bias) including missing data. The software used to simulate sequence reads (RADinitio) also simulates missing data. The higher the read depth set for the software, the lower will be the rate of missing data once the chance of sequencing common loci between all samples increases. RADinitio does not have a specific parameter for controlling the missing data rate but it is proportional to the read depth parameter. This relation (read depth x missing data) was also observed in the empirical data evaluated. The rose data set has a smaller percentage of missing data compared to the aspen data. In this pipeline, the amount of missing data has a higher effect on the number of markers used to build the linkage map than the genotyping error, once we filter the markers with a maximum of 25% of missing data before starting the linkage map building. The HMM method used to estimate the genetic distances have the capacity to input the missing data, but high percentages of it can demand more time to process. The correct imputation of the missing data will depend on the correct information of the given genotypes.

Answer: We highlight in the text that the PCR bias and the duplicates can generate more genotyping errors in GBS data compared to other library types such as whole genome and exome sequencing. The bias changes the proportion of alleles in heterozygous individuals and can lead to wrong estimations of true heterozygous genotypes as homozygous. Also, differently from other technologies, the GBS data is composed basically of duplicates (sequences that start and end in the same position, the cut sites). This makes it impossible to distinguish optical duplicates and sequencing artifacts. The non-removal of the optical duplicates can lead to the wrong estimation of homozygous genotypes as heterozygous.

Answer: Other library types can also be evaluated in Reads2Map. In the EmpiricalSNPCalling sub-workflow, the single difference would be to set the parameter to remove duplicates as TRUE (lines 478-493).

The authors also mentioned that sequencing error may cause distortion segregation in linkage map construction, however, distortion segregation in linkage map construction can also happen for correct genotyping data. The distortion segregation can be caused by individual selection during the construction of the population. So I don't think it is correct to use distortion segregation to correct sequence errors.

Answer: We can think about the effect of segregation distortion in two different steps of the pipeline. The first is in the genotype calling and the second is in the linkage map. The genotype/dosage calling software updog, polyRAD, and SuperMASSA use the population expected segregation as prior to calling the genotypes. Their work highlights the advantages of doing it. With our simulations, we tested how much their estimation would be affected in the presence of true segregation distortion (Supplementary figure 9 and 10), which reveal a slightly lower efficiency.

Answer: In the linkage map step, the presence of true segregation distortion should not affect the linkage map building. However, at first, it is not possible to distinguish between markers with segregation distortion caused by genotyping errors and markers with biologically explained segregation distortion. We adopt the strategy of being restrictive at the beginning and filter all markers presenting segregation distortion to avoid higher map inflation. Once the pipeline is selected and the linkage map main structure is built, we can recover the discarded markers and insert them using the TRY algorithm. At this point, we will

be able to check in the recombination fraction matrix plot which distorted markers fit the linkage group (true segregation distortion) and which do not.

The authors need to clear the major question of this MS, in the abstract, the authors highlight the sequence errors, while in the introduction, the authors highlight the package for linkage map construction (the last paragraph). Actually, from the MS, authors were assembling a framework for genotyping-by-sequencing data.

Answer: The same was suggested by the other reviewers. We adapted the text to highlight the goal of Reads2Map as a tool to select bioinformatic pipelines previously to the linkage map building.

Two major reduced-represented sequencing approaches, GBS and RADseq, have specific tools for genotype calling, such as Tassel and Stack. However, the authors used the GATK and Freebayes pipeline for variant calling, authors need to present the reason they were not using TASSEL and Stack.

Answer: We implemented TASSEL and Stacks, made new tests, and updated the text accordingly.

In the genotyping-by-sequencing data, individuals were barcoded and mixed during sequencing, what package/code was used to split the individuals (demultiplex) from the fastq for GATK and Freebayes pipeline?

Answer: We used the STACKs plugin process_radtags for that. This is not included in the main workflows because we think the sequences need to be evaluated through FASTQC and the filtering steps need to be made accordingly before starting the SNP calling. They will variate a lot depending on the library type and technology used. The Reads2Map workflows require already filtered and demultiplexed FASTQ files. But we provided a suggestion on how to do that in the Preprocess.wdl workflow which is also available in the GitHub repository.

The maximum missing data was allowed at 25% for markers data, how about for the individual missing rate?

Answer: Our strategy keeps all individuals even if some of them have a higher percentage of missing data to account for as many as possible recombination events in the population. As mentioned before, the HMM has the capacity to impute the missing data.

On page 6, the authors mentioned 'seuqnece size of 350', what that means?

Answer: This refers to the RADinitio parameter –insert-mean. We changed the text to make it clearer.
