# Supplementary material for: Developing best practices for genotyping-by-sequencing analysis in the construction of linkage maps
Source: Gigascience. 2023 Oct 27;12:giad092. doi: 10.1093/gigascience/giad092 (PMC10603770; doi:10.1093/gigascience/giad092)
Supplement: giad092_Response_to_Reviewer_Comments_Revision_1 [file giad092_response_to_reviewer_comments_revision_1.pdf]

Author's Response To Reviewer Comments

Dear editor and reviewers,

We changed the title as suggested by reviewer #2 and made the other required modifications.

Thank you for your great work on this.
