# Supplementary material for: Developing best practices for genotyping-by-sequencing analysis in the construction of linkage maps
Source: Gigascience. 2023 Oct 27;12:giad092. doi: 10.1093/gigascience/giad092 (PMC10603770; doi:10.1093/gigascience/giad092)
Supplement: giad092_Response_to_Reviewer_Comments_Revision_2 [file giad092_response_to_reviewer_comments_revision_2.pdf]

Author's Response To Reviewer Comments

Hello,

We included a new symbol to denote the shared senior authorship and kept only two corresponding authors.

Best regards
