# Supplementary material for: Developing best practices for genotyping-by-sequencing analysis in the construction of linkage maps
Source: Gigascience. 2023 Oct 27;12:giad092. doi: 10.1093/gigascience/giad092 (PMC10603770; doi:10.1093/gigascience/giad092)
Supplement: giad092_Reviewer_1_Report_Original_Submission — Peter M. Bourke, Ph.D. -- 3/23/2023 Reviewed [file giad092_reviewer_1_report_original_submission.pdf]

Reviewer Report

Title: Developing best practices for genotyping-by-sequencing analysis in the construction of linkage maps

Version: Original Submission **Date:** 3/23/2023

Reviewer name: Peter M. Bourke, Ph.D.

Reviewer Comments to Author:

I read with interest the manuscript on Reads2Map, a really impressive amount of work went into this and I congratulate the authors on it. However, it is precisely this almost excessive amount of results that for me was the major drawback with this paper. I got lost in all the detail, and therefore I have suggested a Major Revision to reflect that I think the paper could be somehow made more stream lined with a clearer central message and fewer figures in the text. Line numbers would have been helpful, I have tried to give the best indication of page number and position, but in future @GigaScience please stick to line numbers for reviewers, it's a pain in the neck without them. Overall I think this is an excellent manuscript of general interest to anyone working in genomics, and definitely worthy of publication.Here are my more detailed comments:General comment: if a user would like to use GBS data for other population types than those amenable for linkage mapping (e.g. GWAS or genomic prediction, so a diversity panel or a breeding panel), how could your tool be useful for them?Other general comment: the manuscript is long with an exhaustive amount of figures and supplementary materials. Does it really need to be this detailed? It appears like the authors lost the run of themselves a little bit and tried to cram everything in, and in doing so risk losing the point of the endeavour. What is the central message of this manuscript? Regarding the figures, the reader cannot refer to the figures easily as they are now mainly contained on another page. Do you really need Figures 16-18 for example? Figures 13 and 14 could be combined perhaps? I am sure that at most 10 figures and maybe even less are needed in the main text, otherwise figures will always be on different pages and hence lose their impact in the text call-out.Abstract and page 4: "global error rate of 0.05" - How do you motivate the use of a global error rate of 5%? Surely this is dataset-dependent?Page 4 - how can a user estimate an error per marker per individual? The description of the create_probs function suggests there is an automatic methodology to do this, but I don't see it described. You could perhaps refer to Zheng et al's software polyOrigin, which actually locally optimises the error prior per datapoint. Maybe something for the discussion.Page 6 "recombination fraction giving the genomic order" do you mean "given"?Page 10 section Effects of contaminant samples - if you look at Figure 9 you can see that the presence of contaminant samples seems to have an impact on the genotypes of other, non-contaminant samples, especially using GATK and 5% global error. With the contaminants present, the number of XO points decreases in many other samples. This is very odd behaviour I would have thought. Is it known whether this apparent suppresion of recombination breakpoints in non-contaminant individuals is likely to be "correct"? Perhaps the SNP caller was running under the assumption that all individuals were part of the same F1? If the SNP caller was run without this assumption (eg. specifying only HW equilibrium, or model-free) would we still see the same effect? This is for me a quite worrying result but something that

you make no reference to as far as I can tell.Page 12 "Effects of segregation distortion" In your study you only considered a single linkage group. One of the primary issues with segregation distortion in mapping is that it can lead to linkage disequilibrium between chromosomes, if selection has occurred on multiple loci. This can then lead to false linkages across linkage groups. Perhaps good to mention this.Page 12 "have difficulty missing linkage information" - missing word "with"Page 17 I see no mention of the impact of errors in the multi-allelic markers on the efficiency, particularly of order_seq which seems to be very poorly-performing with only bi-allelics (Fig 20). If bi-allelic SNPs have errors then it is not obvious why multi-SNP haplotypes should not also have errors.Page 3 Figure 1 - here the workflow shows multiple options for a number of the steps, which can lead to the creation of many map variants (e.g. 816 maps as mentioned on Page 4). Should all users produce 816 variants of their maps? With potentially millions of markers, this is going to take a huge amount of time (most users will want 100% of all chromosomes, not 37% of a single chromosome). Or should this be done for only a subset of markers? What if there is no reference sequence available to select a subset? As there are no clear recommendations, I suspect that the specific combination of pipeline choices will usually be dataset-dependent. You actually mention this in the discussion page 17. And with only 2 real datasets from 2 different species, there is also no way to tell if eg. GATK works best in rose, or updog should be used for monocots but not dicots etc. It would be helpful if the authors were more explicit about how their tool informs "best practices for GBS analysis" for ordinary users. Perhaps it is there, but for me this message gets lost.Page 17 "updates in this version 3.0 to resolve issues with inflated genetic maps" - if I look at Figure 20, it seems that issues with inflated map length have not yet been fully resolved!Page 17 "we provide users tools to select the best approaches" - similar comment as before - does this mean users should build > 800 maps with a subset of their dataset first, and then use this single approach for the whole dataset? It is not explicitly stated whether this is the guidance given. What is the eventual aim - to produce a good linkage map, or to use the linkage map to critically compare genotyping tools?

Level of Interest

Please indicate how interesting you found the manuscript: Choose an item.

Quality of Written English

Please indicate the quality of language in the manuscript: Choose an item.

Declaration of Competing Interests

Please complete a declaration of competing interests, considering the following questions:

- Have you in the past five years received reimbursements, fees, funding, or salary from an organisation that may in any way gain or lose financially from the publication of this manuscript, either now or in the future?
- Do you hold any stocks or shares in an organisation that may in any way gain or lose financially from the publication of this manuscript, either now or in the future?

- Do you hold or are you currently applying for any patents relating to the content of the manuscript?
- Have you received reimbursements, fees, funding, or salary from an organization that holds or has applied for patents relating to the content of the manuscript?
- Do you have any other financial competing interests?
- Do you have any non-financial competing interests in relation to this paper?

If you can answer no to all of the above, write 'I declare that I have no competing interests' below. If your reply is yes to any, please give details below.

'I declare that I have no competing interests'

I agree to the open peer review policy of the journal. I understand that my name will be included on my report to the authors and, if the manuscript is accepted for publication, my named report including any attachments I upload will be posted on the website along with the authors' responses. I agree for my report to be made available under an Open Access Creative Commons CC-BY license (http://creativecommons.org/licenses/by/4.0/). I understand that any comments which I do not wish to be included in my named report can be included as confidential comments to the editors, which will not be published.

Choose an item.

To further support our reviewers, we have joined with Publons, where you can gain additional credit to further highlight your hard work (see: https://publons.com/journal/530/gigascience). On publication of this paper, your review will be automatically added to Publons, you can then choose whether or not to claim your Publons credit. I understand this statement.

Yes Choose an item.
