# Supplementary material for: Developing best practices for genotyping-by-sequencing analysis in the construction of linkage maps
Source: Gigascience. 2023 Oct 27;12:giad092. doi: 10.1093/gigascience/giad092 (PMC10603770; doi:10.1093/gigascience/giad092)
Supplement: giad092_Reviewer_2_Report_Original_Submission — Ramil Mauleon -- 3/23/2023 Reviewed [file giad092_reviewer_2_report_original_submission.pdf]

Reviewer Report

Title: Developing best practices for genotyping-by-sequencing analysis in the construction of linkage maps

Version: Original Submission **Date:** 3/23/2023

Reviewer name: Ramil Mauleon

Reviewer Comments to Author:

The paper titled "Developing best practices for genotyping-by-sequencing analysis using linkage maps as benchmarks" aims to present an end to end workflow uses GBS genotyping datasets to generate genetic linkage maps. This is a valuable tool for geneticists intending to generate a high confidence linkage map from a mapping population with GBS data as input.I got confused on reading the MS though, is this a workflow paper or is this a review of the component software for each step of genetic mapping and how parameter/use differences affect the output ? If it's a review, then the choice of software reviewed are not comprehensive enough, esp on SNP calling, and linkage mapping.There is no clear justification why each component software was used,example the use of GATK and freebayes for SNP calling I am familiar with using TASSEL GBS and STACKS for SNP calling using GBS data, why weren't they included in the SNP calling software. The MS would benefit greatly from including these SNP calling software in their benchmarking.Onemap and gusmap seems also pre-selected for linkage mapping, without reason for use, or maybe the reason(s) were not highlighted in the text. I've had experience in the venerable MAPMAKER and MSTMap, and would like to see more comparisons of the chosen genetic linkage mapping software with others, if this is the intent of the MS.The MS also clearly focuses on genetic linkage mapping using GBS, which should be more explicitly stated in the title. GBS is also extensively used in diversity collections and there is scant mention of this in the MS, and whether the workflow could be adapted to such populations.Versions of sofware used in the workflow are also not explicitly stated within the MS.The shiny app is also not demonstrated well in the MS, it could be presented better with screenshots of the interface , with one or two sample use cases.

Level of Interest

Please indicate how interesting you found the manuscript: Choose an item.

Quality of Written English

Please indicate the quality of language in the manuscript: Choose an item.

Declaration of Competing Interests

Please complete a declaration of competing interests, considering the following questions:

- Have you in the past five years received reimbursements, fees, funding, or salary from an organisation that may in any way gain or lose financially from the publication of this manuscript, either now or in the future?
- Do you hold any stocks or shares in an organisation that may in any way gain or lose financially from the publication of this manuscript, either now or in the future?
- Do you hold or are you currently applying for any patents relating to the content of the manuscript?
- Have you received reimbursements, fees, funding, or salary from an organization that holds or has applied for patents relating to the content of the manuscript?
- Do you have any other financial competing interests?
- Do you have any non-financial competing interests in relation to this paper?

If you can answer no to all of the above, write 'I declare that I have no competing interests' below. If your reply is yes to any, please give details below.

I declare that I have no competing interests.

I agree to the open peer review policy of the journal. I understand that my name will be included on my report to the authors and, if the manuscript is accepted for publication, my named report including any attachments I upload will be posted on the website along with the authors' responses. I agree for my report to be made available under an Open Access Creative Commons CC-BY license (http://creativecommons.org/licenses/by/4.0/). I understand that any comments which I do not wish to be included in my named report can be included as confidential comments to the editors, which will not be published.

Choose an item.

To further support our reviewers, we have joined with Publons, where you can gain additional credit to further highlight your hard work (see: https://publons.com/journal/530/gigascience). On publication of this paper, your review will be automatically added to Publons, you can then choose whether or not to claim your Publons credit. I understand this statement.

Yes Choose an item.
