# Supplementary material for: Developing best practices for genotyping-by-sequencing analysis in the construction of linkage maps
Source: Gigascience. 2023 Oct 27;12:giad092. doi: 10.1093/gigascience/giad092 (PMC10603770; doi:10.1093/gigascience/giad092)
Supplement: giad092_Reviewer_2_Report_Revision_1 — Ramil Mauleon -- 8/9/2023 Reviewed [file giad092_reviewer_2_report_revision_1.pdf]

Reviewer Report

Title: Developing best practices for genotyping-by-sequencing analysis in the construction of linkage maps

Version: Revision 1 **Date:** 8/9/2023

Reviewer name: Ramil Mauleon

Reviewer Comments to Author:

I'm satisfied with your responses to my comments, thanks for addressing this and congratulations to your team for building this workflow. The major re-write you did on focusing on the software components of the workflow made it an easier read for me, as a bioinformatician.My minor comment is still on the title of your paper, "Developing best practices for genotyping-by-sequencing analysis using linkage maps as benchmarks". Your paper primarily presents the application of Reads2map in the construction of genetic linkage map(s). As I've mentioned, I come from a team where GBS is also extensively applied for population diversity analysis, which means constructing genetic linkage maps is not an endpoint target, and the only benefit from Reads2map for population diversity analysis is the automation of SNP calling using several methods (thanks for adding the other methods, by the way), which you mentioned very briefly when you addressed my comment on this.The title of your paper is also a bit confusing since the use of GBS in population genetic applications cannot be benchmarked by linkage map construction, for obvious reasons. I'd suggest a straightforward title like "Developing best practices for genotyping-by-sequencing analysis in the construction of linkage maps" or something like this..

Level of Interest

Please indicate how interesting you found the manuscript: Choose an item.

Quality of Written English

Please indicate the quality of language in the manuscript: Choose an item.

Declaration of Competing Interests

Please complete a declaration of competing interests, considering the following questions:

- Have you in the past five years received reimbursements, fees, funding, or salary from an organisation that may in any way gain or lose financially from the publication of this manuscript, either now or in the future?
- Do you hold any stocks or shares in an organisation that may in any way gain or lose financially from the publication of this manuscript, either now or in the future?

- Do you hold or are you currently applying for any patents relating to the content of the manuscript?
- Have you received reimbursements, fees, funding, or salary from an organization that holds or has applied for patents relating to the content of the manuscript?
- Do you have any other financial competing interests?
- Do you have any non-financial competing interests in relation to this paper?

If you can answer no to all of the above, write 'I declare that I have no competing interests' below. If your reply is yes to any, please give details below.

I declare that I have no competing interests

I agree to the open peer review policy of the journal. I understand that my name will be included on my report to the authors and, if the manuscript is accepted for publication, my named report including any attachments I upload will be posted on the website along with the authors' responses. I agree for my report to be made available under an Open Access Creative Commons CC-BY license (http://creativecommons.org/licenses/by/4.0/). I understand that any comments which I do not wish to be included in my named report can be included as confidential comments to the editors, which will not be published.

Choose an item.

To further support our reviewers, we have joined with Publons, where you can gain additional credit to further highlight your hard work (see: https://publons.com/journal/530/gigascience). On publication of this paper, your review will be automatically added to Publons, you can then choose whether or not to claim your Publons credit. I understand this statement.

Yes Choose an item.
