# Supplementary material for: Developing best practices for genotyping-by-sequencing analysis in the construction of linkage maps
Source: Gigascience. 2023 Oct 27;12:giad092. doi: 10.1093/gigascience/giad092 (PMC10603770; doi:10.1093/gigascience/giad092)
Supplement: giad092_Reviewer_3_Report_Original_Submission — Zhenbin Hu -- 3/27/2023 Reviewed [file giad092_reviewer_3_report_original_submission.pdf]

Reviewer Report

Title: Developing best practices for genotyping-by-sequencing analysis in the construction of linkage maps

Version: Original Submission **Date:** 3/27/2023

Reviewer name: Zhenbin Hu

Reviewer Comments to Author:

In this MS, the authors tried to develop a framework for using GBS data for downstream analysis and reduce the impact of sequence errors caused by GBS. However, sequence error is an issue not specific to GBS, it is also for whole genome sequences. Actually, I think the major issue for GBS is the missing data. However, in this MS, the authors did not test the impact of missing data on downstream analysis.The authors also mentioned that sequencing error may cause distortion segregation in linkage map construction, however, distortion segregation in linkage map construction can also happen for correct genotyping data. The distortion segregation can be caused by individual selection during the construction of the population. So I don't think it is correct to use distortion segregation to correct sequence errors.The authors need to clear the major question of this MS, in the abstract, the authors highlight the sequence errors, while in the introduction, the authors highlight the package for linkage map construction (the last paragraph). Actually, from the MS, authors were assembling a framework for genotyping-by-sequencing data.Two major reduced-represented sequencing approaches, GBS and RADseq, have specific tools for genotype calling, such as Tassel and Stack. However, the authors used the GATK and Freebayes pipeline for variant calling, authors need to present the reason they were not using TASSEL and Stack.In the genotyping-by-sequencing data, individuals were barcoded and mixed during sequencing, what package/code was used to split the individuals (demultiplex) from the fastq for GATK and Freebayes pipeline?The maximum missing data was allowed at 25% for markers data, how about for the individual missing rate?On page 6, the authors mentioned 'seuqnece size of 350', what that means?

Level of Interest

Please indicate how interesting you found the manuscript: Choose an item.

Quality of Written English

Please indicate the quality of language in the manuscript: Choose an item.

Declaration of Competing Interests

Please complete a declaration of competing interests, considering the following questions:

- Have you in the past five years received reimbursements, fees, funding, or salary from an organisation that may in any way gain or lose financially from the publication of this manuscript, either now or in the future?
- Do you hold any stocks or shares in an organisation that may in any way gain or lose financially from the publication of this manuscript, either now or in the future?
- Do you hold or are you currently applying for any patents relating to the content of the manuscript?
- Have you received reimbursements, fees, funding, or salary from an organization that holds or has applied for patents relating to the content of the manuscript?
- Do you have any other financial competing interests?
- Do you have any non-financial competing interests in relation to this paper?

If you can answer no to all of the above, write 'I declare that I have no competing interests' below. If your reply is yes to any, please give details below.

I declare that I have no competing interests

I agree to the open peer review policy of the journal. I understand that my name will be included on my report to the authors and, if the manuscript is accepted for publication, my named report including any attachments I upload will be posted on the website along with the authors' responses. I agree for my report to be made available under an Open Access Creative Commons CC-BY license (http://creativecommons.org/licenses/by/4.0/). I understand that any comments which I do not wish to be included in my named report can be included as confidential comments to the editors, which will not be published.

Choose an item.

To further support our reviewers, we have joined with Publons, where you can gain additional credit to further highlight your hard work (see: https://publons.com/journal/530/gigascience). On publication of this paper, your review will be automatically added to Publons, you can then choose whether or not to claim your Publons credit. I understand this statement.

Yes Choose an item.
